# Computational Kinetic Study on the Intramolecular H-Migration of Hydroperoxyalkylperoxy Radicals (•OOQOOH) in Normal-Alkyl Cyclohexanes

**DOI:** 10.3390/molecules30132805

**Published:** 2025-06-29

**Authors:** Xiaoxia Yao, Juanqin Li, Zerong Li

**Affiliations:** 1 College of Chemistry, Sichuan University, Chengdu 610064, China; yaoxxkgd@163.com; 2 College of Chemical Engineering, Sichuan University, Chengdu 610065, China; lijuanqin@scu.edu.cn; 3 Engineering Research Center of Combustion and Cooling for Aerospace Power, Ministry of Education, Sichuan University, Chengdu 610065, China

**Keywords:** dihydroperoxyalkyl radical, intramolecular H-migration, normal-alkyl cycloalkanes, high-pressure limit rate rules

## Abstract

Hydroperoxyalkylperoxy radicals (•OOQOOH) are important intermediates in the low-temperature oxidation chemistry of conventional fuels. In these species, a hydrogen atom may migrate from a non-adjacent carbon to the peroxy group, forming a dihydroperoxyalkyl radical (•P(OOH)_2_). This research delves into the theoretical kinetics of a set of 110 H-migration reactions in normal-alkyl cyclohexanes, calculating high-pressure limit rate constants for these reactions. The reactions are further classified into 15 subclasses based on distinctions in the reaction center and its environment, with rate rules derived by averaging the rate constants within each subclass. A comparison of our calculated rate constants for specific H-migration reactions of •OOQOOH with existing mechanisms and similar reactions in non-cyclic alkanes reveals significant disparities, emphasizing the necessity for precise rate constants tailored to normal-alkyl cyclohexanes. Ethyl cyclohexane mechanisms and n-propyl cyclohexane mechanisms sourced from studies have been improved with high-pressure limit rate constants from this study. Simulations of the low-temperature combustion of ethyl cyclohexane and n-propyl cyclohexane show that the predictions from the updated mechanisms align more closely with the experimental data under specific conditions compared to the original mechanism.

## 1. Introduction

Cycloalkanes are important components of transportation fuels, accounting for about 35% of diesel fuels, 20% of aviation fuel, and 10% of gasoline [1]. Cycloalkanes were also found to be important in the formation of aromatic pollutants [2] and polycyclic aromatic soot precursors [3]. Considering their high concentration in practical fuels and thus their great relevance in combustion processes, cycloalkanes have been widely used as surrogate components for practical fuels. Therefore, adequate knowledge of cycloalkane chemistry and kinetics is necessary in order to improve transport fuel combustion efficiencies and reduce emission profiles. The cycle structure of cycloalkanes introduces some unique reaction channels, such as cycle-opening reactions, which increase the complexity of the reaction scheme and the reaction mechanism compared with alkanes. The auto-oxidation chemistry of hydrocarbons plays an important role in fuel combustion and atmospheric processes [4].

The reaction sequence of hydrocarbon combustion at low temperatures has been described in detail previously [5,6]. The initial step of hydrocarbon combustion at low temperatures is the H-abstraction from the fuels to form radicals, which then react with O_2_ addition to form peroxyalkyl radicals (ROO•) [7,8], while the intramolecular H-migration of the ROO• radicals can produce hydroperoxy alkyl radicals (•QOOH), to which the second O_2_ is added to the carbon radical site of •QOOH to form hydroperoxyalkylperoxy radicals (•OOQOOH), where the first intramolecular H-migration distance in ROO• determines the distance between the two OO-bonded carbons in •OOQOOH, i.e., the isomers of •OOQOOH. •OOQOOH can also undergo intramolecular H-migration from a carbon atom to the end atom of the -OO moiety, and if this carbon atom is a non-α carbon to the -OO moiety, the intramolecular H-migration of •OOQOOH will lead to a dihydroperoxyalkyl radical (•P(OOH)_2_). This work, as noted by Klippenstein in his seminal review on reaction dynamics and chemical modeling in combustion [9], is a pioneering study on second O_2_ kinetics, which is responsible for •OOQOOH formation. Recent kinetic studies [10,11,12,13,14,15,16,17] have demonstrated the significance of these channels for the intramolecular H-migration of •OOQOOH. Analysis of the oxidation reaction sequence of hydrocarbon fuels at low temperatures reveals the importance of the intramolecular H-migration reactions of both ROO• and •OOQOOH. Their subsequent reactions play a crucial role in chain branching, which in turn accelerates combustion at low temperatures. Kinetic calculations for the intramolecular H-migration of ROO• have been extensively conducted for alkanes [10,15,16,18,19,20] and cyclohexanes [21,22,23,24,25,26,27,28,29,30]. Similarly, there is a wealth of kinetic calculations for alkanes [9,10,15,16,17] in the class of the intramolecular H-migration of •OOQOOH. However, there is a notable scarcity of kinetic studies on the intramolecular H-migration of •OOQOOH for cyclohexanes in low temperature oxidation.

Currently, numerous modeling studies have investigated the combustion process of normal-alkyl cyclohexanes. Nonetheless, the absence of precise rate constants for the H-migration of the •OOQOOH class in normal-alkyl cyclohexane has resulted in limited consideration of this reaction class in many combustion mechanisms. Moreover, in combustion mechanisms that do include this reaction class, the kinetic data are often estimated from analogous H-migration of the •OOQOOH class in non-cyclic alkanes or from analogous H-migration reactions of ROO• in normal-alkyl cyclohexane. Zou et al. [31] have developed a low-temperature combustion mechanism for ethyl cyclohexane, utilizing rate constants for the H-migration of •OOQOOH derived from analogous intramolecular H-migration reactions of ROO• in low-temperature combustion mechanisms of cyclohexane [24], methyl cyclohexane [25,26], and ethyl cyclohexane in previous studies [28,29], as well as similar reactions of alkanes [15]. Recently, Liu et al. [32] have developed a comprehensive chemical kinetic model for n-propyl cyclohexane combustion, utilizing a core mechanism from AramcoMech 3.0. The model integrates 34 reaction classes, incorporating detailed rate constants for intramolecular H-migration reactions of •OOQOOH to produce •P(OOH)_2_. These rate constants were approximated using similar reactions of intramolecular H-migration of ROO• observed in a prior investigation of n-propyl cyclohexane [29]. In our previous study [29], a comparison of the rate constants of analogous reactions between normal-alkyl cycloalkanes and alkanes revealed significant differences. As a result, the kinetic data of the reactions in alkanes cannot be applied to the corresponding analogous reactions in normal-alkyl cycloalkane mechanisms. Therefore, precise kinetic parameters for the intramolecular H-migration reactions of •OOQOOH to form •P(OOH)_2_ in normal-alkyl cyclohexanes are crucial for developing accurate low-temperature combustion models.

To our knowledge, there are no kinetic calculation reports about the intramolecular H-migration reactions of •OOQOOH in normal-alkyl cyclohexanes. As the alkyl side chains get longer, more fuel-derived radicals have to be considered and the complexity of H-migration reactions is increased dramatically. This will greatly increase the numbers of reactions of intramolecular H-migration reactions of •OOQOOH. Additionally, the molecular structure of the reactants in normal-alkyl cyclohexane is further complicated by the presence of the cycle and four oxygen atoms, particularly with regard to •OOQOOH. The incorporation of four oxygen atoms in •OOQOOH can result in intramolecular hydrogen bonding (HB), thereby posing a challenge in calculating the rate constants and developing the kinetic mechanisms. Previous studies have examined the impact of HB on the decomposition and isomerization reactions of the intermediates involved in the oxidation of fuels [15,33,34,35,36,37]. Hence, comprehensive kinetic investigations into the intramolecular H-migration reactions of •OOQOOH are of considerable importance.

The aim of this work is to study the kinetics for the intramolecular H-migration reactions of •OOQOOH to form •P(OOH)_2_ at different temperatures, and develop the high-pressure limit rate rules for them, which are the prerequisites for automatic generation of low-temperature oxidation mechanisms for normal-alkyl cycloalkanes.

## 2. Results

### 2.1. Potential Energy Surfaces

•OOQOOH are generated by the addition of O_2_ to the radical sites of •QOOH, which are produced by intramolecular H-migration of ROO•. Therefore, the configuration of the •OOQOOH is determined by the configuration of •QOOH. Theoretical and modeling studies on normal-alkyl cyclohexanes by Knepp et al. [22], Xing et al. [25,26], Serinyel et al. [38], Vranckx et al. [39], and Bissoonauth et al. [40] have shown that 1,5 H-migration dominates the intramolecular H-migration of ROO•. Hence, Zou et al. [31] considered the •QOOH generated through the 1,5 H-migration of ROO• as a primary intermediate for subsequent oxygenation in their development of a low-temperature combustion mechanism for ethyl cyclohexane.

**Scheme 1:**
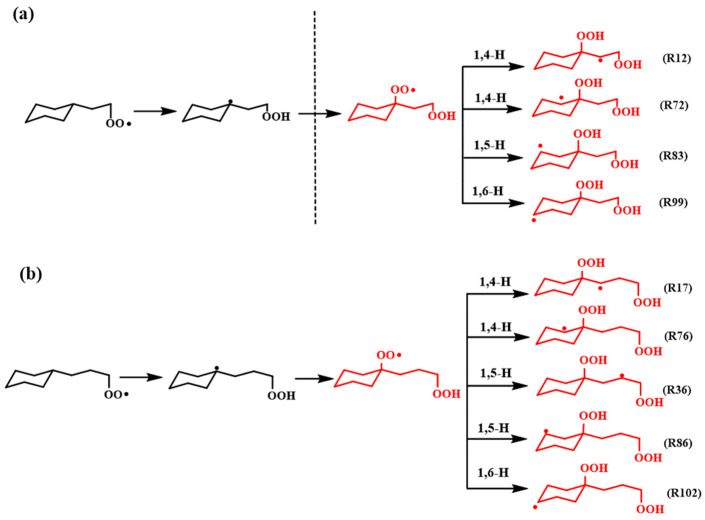
Scheme for the reactions from ROO → QOOH → OOQOOH → products: (**a**) corresponding to 1,5 H-migration in ROO•; (**b**) corresponding to 1,6 H-migration in ROO•. (The reactions in red are the targets of this work.)

In our previous study of the H-migration reactions that occur after the first oxygenation of normal-alkyl cyclohexane, it was shown that the formation of •QOOH by 1,5 H-migration of ROO• is the main intermediates for further second oxygenation reactions. In some cases, the energy barriers of •QOOH formed by 1,6 H-migration reactions of ROO• were lower than those of 1,5 H-migration reactions, suggesting that •QOOH formed by 1,6 H-migration reactions of ROO• may also be the main intermediates for the second step of oxygenation. The reactants •OOQOOH in this study were obtained by the addition of •QOOH and O_2_, so it is necessary to consider the channels of 1,5 H-migration and 1,6 H-migration of ROO•. Examples of the scheme are shown in Scheme 1a,b, and other schemes are shown in Schemes S1–S4 of the Supplemental Material—File S1.

In this study, the composition of a transition state within a reaction class is divided into two separate parts: the reaction center and the substituents. The shared aspect of the reaction structure within a given reaction class (encompassing the reactive atoms and their interconnected bonds) is identified as the reaction center. Substituents, such as hydrogen atoms or alkyls, can be designated as part of this categorization. The presence of different substituents may introduce disparities among reactions within the same reaction class. In the majority of automatic mechanism generation programs, kinetic data is typically produced using the simple rate rule method [41]. This approach involves assigning the same rate constants to all reactions within a reaction class. However, the considerable discrepancies between different reactions within a reaction class can result in significant uncertainty when using the same rate constants. To address this issue, it is common practice to divide reactions in a class into subclasses according to the substituent groups involved. A rate rule for each subclass is then developed.

This study further classifies the intramolecular H-migration reactions of •OOQOOH into more subclasses based on the following: (1) the number of atoms connected in the TS cycles, denoted as 1,4-H migration reactions (TS cycle connects five atoms), 1,5-H migration reactions (TS cycle connects six atoms) and 1,6-H migration reactions (TS cycle connects seven atoms); (2) the types of carbons bonded to the migrated H atom, denoted as primary (p), secondary (s), and tertiary (t) carbon atoms; (3) the reaction centers being located either on the alkyl side chain or on the cycle; (4) when the reaction center involves only cycle atoms, the position of the -OOH moiety will also have a certain effect on the energy barrier of the reaction, so the location of the -OOH moiety on the alkyl side chain or the cycle is also classified; and (5) the •OOQOOH produced by the addition of O_2_ to the product of the 1,5 H-migration of ROO• or 1,6 H-migration of ROO•. The representative reactions in this study focus solely on cyclohexanes with methyl, ethyl, n-propyl, and n-butyl alkyl side chains, omitting cyclohexanes with longer alkyl side chains. As a result of this limitation, certain hydrogen migration reactions are precluded. Consequently, some subclasses may have an abundance of representative responses, while others may only have a few. For a comprehensive overview of the subclasses studied, refer to Table 1.

### 2.2. Lowest Energy Conformations

In this work, the conformations of all species are approximately obtained by taking the combination of the most stable six-membered cycle conformation and the most stable alkyl side chain conformation [29]. Specifically, for a six-membered cycle, the chair conformation [42,43] has the lowest energy, while for alkanes, the most extended conformation has the most stable energy advantage. However, in the case of the reactants of the intramolecular H-migration of •OOQOOH, the lowest energy structure is not simply a result of the combination of a six-membered cycle and the chain conformation. In this work, the chair conformation for the cyclohexane cycle was chosen to be kept unchanged. To verify the energy difference between the boat and chair conformations, the representative reactions R5 and R57 were selected, and single-point energy calculations were performed for these conformations of the reactants, transition states, and products involved in the aforementioned reactions. The results are presented in Table 2. It can be observed from Table 2 that the single-point energies of the boat conformations of the reactants, transition states, and products in reactions R5 and R51 are all higher than those of the chair conformations. Therefore, in order to streamline the number of systems studied, only the most stable chair conformation is considered in this study.

In the case of the reactants of the intramolecular H-migration of •OOQOOH, the lowest energy structure is not simply a result of the combination of a six-membered cycle and the chain conformation. This is due to the presence of -OOH moiety and -OO moiety, which may introduce intramolecular hydrogen bonds. Consequently, the effect of intramolecular hydrogen bonds on the conformation is also taken into account in this study. Taking into account the relative stability of the single bonds within the cycle conformation, we conducted a loose scan of the dihedral angles where all the single bonds on the side chain are situated, at an increment of 10 degrees, using the B3LYP/CBSB7 level. This approach allowed us to explore the lowest energy potential energy surface and determine the most stable normal-alkyl cyclohexane conformation. The Cartesian coordinates for all species in the 110 studied reactions in this study are provided in the Supplemental Material—File S2.

The reaction R57 was chosen in this study was to demonstrate the impact of hydrogen bonds in both reactants and transition states on the energy barrier, as illustrated in Table 3. To differentiate between cycle-shaped structures formed by intramolecular hydrogen bonds and the reaction centers in the conformations of the transition states, the latter are indicated by green dashed lines. When no intramolecular hydrogen bond is formed in the reactant and transition state, the energy barrier of reaction (1) is 31.7 kcal mol^−1^. The energy barrier of reaction (2) is 32.2 kcal mol^−1^ when there is an intramolecular hydrogen bond in the reactant conformation but no intramolecular hydrogen bond in the transition state conformation. When no intramolecular hydrogen bond is formed in the reactant conformation, but the intramolecular hydrogen bond is formed in the transition state conformation, the energy barrier of reaction (3) is 29.9 kcal mol^−1^. When intramolecular hydrogen bonds are formed in both the reactant and transition state conformations, the energy barrier of reaction (4) is 30.5 kcal mol^−1^.

Upon analyzing the intrinsic reaction coordinate (IRC) for the four reactions listed in Table 3, it becomes evident that only reactant conformations featuring intramolecular hydrogen bonds can transition to transition state conformations with intramolecular hydrogen bonds. Conversely, reactant conformations lacking intramolecular hydrogen bonds can only transition to transition state conformations that also lack intramolecular hydrogen bonds. Consequently, only reactions (1) and (4) from Table 3 are feasible. To summarize, the conformations of intramolecular hydrogen bonds in both the reactants and the transition state structures are chosen as the focus of this study due to their ability to substantially lower the energy barrier of the reaction.

**Figure 1:**
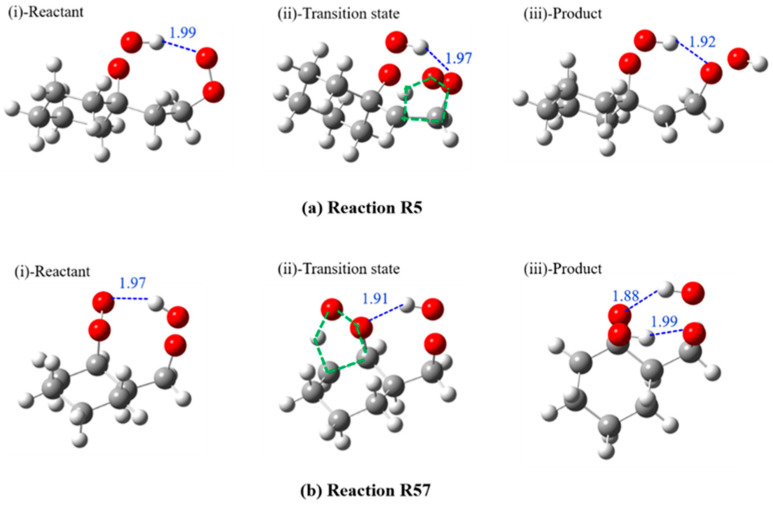
The hydrogen bonds of the species involved in reactions R5 and R57 (The red balls in the figure represent oxygen atoms, and the TS reaction centers are marked in green). Values represent distances in Angstroms.

Since there are four oxygen atoms in the species of the reactions studied in this work, when oxygen atoms form intramolecular hydrogen bonds with hydrogen atoms, different molecular conformations will be generated, taking the reaction R5 and R57 as examples, as shown in Figure 1. In Figure 1, in order to distinguish the difference between cycle-shaped structures caused by intramolecular hydrogen bonds in the transition state and the reaction center in the transition state, we marked the reaction center in the transition states with green dashed lines. As can be seen from Figure 1, the lengths of hydrogen bonds vary in different species of the reactions.

### 2.3. Energy Barriers for Subclasses

In most automated mechanism generators, the standard practice involves employing the generic rate rule method [41], where identical rate constants (denoted as (A, n, E)) are applied to all reactions within a specific reaction class. In this study, to minimize the uncertainty associated with the simple rate rule method, the reaction classes are subdivided into subclasses, and the simple rate rule method is applied to each subclass separately. To assess the validity of the developed rate rules, the maximum energy barrier deviation among each subclass is calculated. Our calculation results indicate that the location of the reaction center also affects the energy barrier for the reaction producing P(OOH)_2_ products; therefore, we have categorized these distinctions.

#### 2.3.1. Subclasses of Reaction Centers Containing Alkyl Side Chains

The energy barriers for the reactions of reaction centers containing alkyl side chains are listed in Table 4. In this work, for the reactions of reaction centers containing alkyl side chains, there would be a large uncertainty if all these reactions were classified into a class and a rate rule was constructed for it. Therefore, these reactions were further divided into nine different subclasses based on the number of atoms connected in the TS cycles, and the types of carbons to which the migrating H atom is attached, with the results listed in Table 4. From Table 4, it is evident that the maximum deviations in energy barriers among these subclasses do not exceed 4.5 kcal mol^−1^, leading to rate constants at 500 K, 800 K, and 1500 K of 90, 20, and 5 times, respectively. Moreover, it is important to note that the combustion temperature of hydrocarbons typically exceeds 500 K. In this context, it is observed that the uncertainties of the rate constants decrease as the temperatures increase. This trend reinforces the acceptability and reasonableness of the developed rate rules presented in this work.

#### 2.3.2. Subclasses of Reaction Centers Involving Only Cycle Atoms

The energy barriers for the reactions of reaction centers involving only cycle atoms are listed in Table 5. In this work, for the reactions of reaction centers involving only cycle atoms, these reactions are further divided into six different subclasses based on the number of atoms connected in the TS cycles, the types of carbons to which the migrating H atom is attached, and the locations of the -OOH moiety, with the results listed in Table 5. It is evident from Table 5 that the maximum deviations of energy barriers of these subclasses do not exceed 5.0 kcal mol^−1^, leading to rate constants at 500 K, 800 K, and 1500 K of about 150, 20, and 5 times, respectively. It is shown that the uncertainties of the rate constants decrease with increasing temperatures, and generally the combustion temperature of hydrocarbons exceeds 500 K, so it is reasonable to assign the same rate constant to different reactions in each subclass at higher temperature ranges.

In summary, it is necessary that the intramolecular H-migration reactions of •OOQOOH are classified into subclasses according to the difference in the reaction center and the environment around the reaction center, and our calculated results indicate that using the classification schemes, the maximum deviation of the energy barrier in each subclass is almost below 5.0 kcal mol^−1^. This result indicates the acceptability and reasonableness of the developed rate rules presented in this work.

### 2.4. High-Pressure Limit Rate Constants and Rate Rules

In this work, rate constants calculated over the temperature range of 500 K to 1500 K are fitted to the modified Arrhenius equation $k(T)=AT^{n}exp[-E_{a}/RT]$. The fitted parameters (A, n, E) are given in Table 6 and Table 7. From the rate constants for reactions in a subclass, the average rate constants at each temperature are obtained by the equation $k_{avg}^{T}=\frac{1}{N}\sum_{i=1}^{i=N}k_{i}^{T}$, where *N* represents the number of reactions, $k_{i}^{T}$ represents the rate constant at *T* for reaction *i*, and $k_{avg}^{T}$ is the average rate constant at temperature *T*. Then, these averaged rate constants are fitted to the modified Arrhenius equation, yielding a three-parameter representation (*A*, *n*, *E*) that represents the rate rule for each subclass.

To measure the uncertainty and the applicability of the rate rule for a subclass, an uncertainty factor f of a reaction subclass is defined as f = kmax/kmin, where kmax and kmin are the maximum and the minimum rate constant in a subclass, respectively. k/kave, which is defined as the ratio of the rate constant of a reaction to the average rate constant of the reactions in a subclass, is also calculated at a given temperature (800 K) to measure the suitability of the rate rules. It can be seen from Table 6 and Table 7 that uncertainty factors and the ratio factors fall within an order of magnitude, suggesting limited variation in the high-pressure limit rate constants for the reactions within.

#### 2.4.1. High-Pressure Limit Rate Rules for Reactions with •P(OOH)_2_ Products

**(1) Subclasses of reaction centers containing alkyl side chains.** The three-parameter (A, n, E) fitting forms of the high-pressure limit rate constants for each subclass of the reaction centers containing the alkyl side chains at the temperature range 500–1500 K are listed in Table 6. 

**Figure 2:**
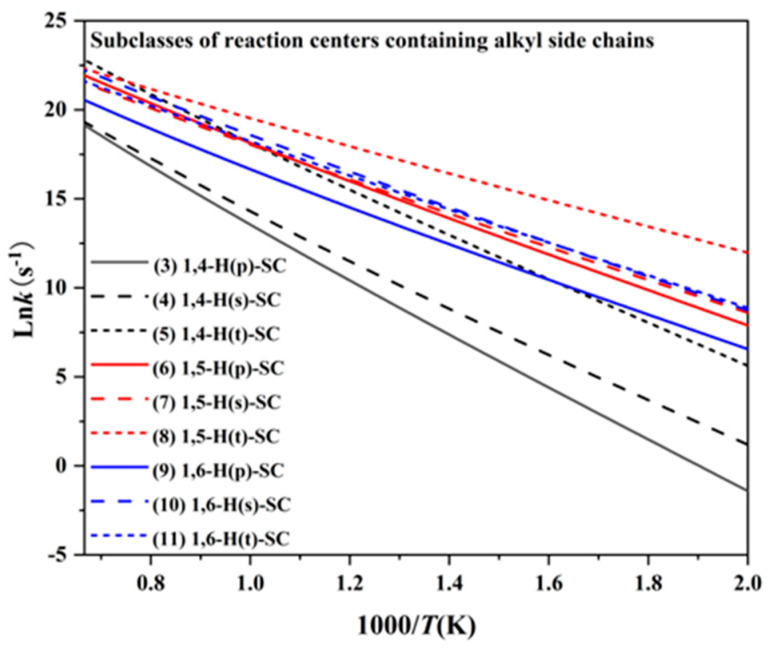
Comparison of the high−pressure limit rate rules between subclasses: reaction centers contain the alkyl side chain.

The comparisons of the high-pressure limit rate constants for different subclasses are shown in Figure 2. It can be seen from Figure 2 that when the reaction centers of subclasses contain the alkyl side chain, the average high-pressure limit rate constants for these subclasses exhibit the following trends: 1,4-H(t)-SC > 1,4-H(s)-SC > 1,4-H(p)-SC, 1,5-H(t)-SC > 1,5-H(s)-SC > 1,5-H(p)-SC, and 1,6-H(t)-SC > 1,6-H(s)-SC > 1,6-H(p)-SC. This indicates that, under the same number of atoms connected in the TS cycles, the rate constants depend on the types of carbon from which the H is migrated in the following order: tertiary carbon > secondary carbon > primary carbon. It also can be seen from Figure 2 that the average high-pressure limit rate constants for these subclasses exhibit the following trends: 1,5-H(p)-SC > 1,6-H(p)-SC > 1,4-H(p)-SC, 1,5-H(s)-SC > 1,6-H(s)-SC > 1,4-H(s)-SC, and 1,5-H(t)-SC > 1,6-H(t)-SC > 1,4-H(t)-SC. This suggests that within the same carbon types from which hydrogen is being migrated, the rate constants vary in relation to the number of atoms connected in the TS cycles in the following sequence: 1,5-H migration > 1,6-H migration > 1,4-H migration.

**(2) Subclasses of reaction centers involving only cycle atoms.** The three-parameter (A, n, E) fitting forms of the high-pressure limit rate constants for each subclass of the reaction centers involving only cycle atoms at the temperature range 500–1500 K are listed in Table 7.

**Figure 3:**
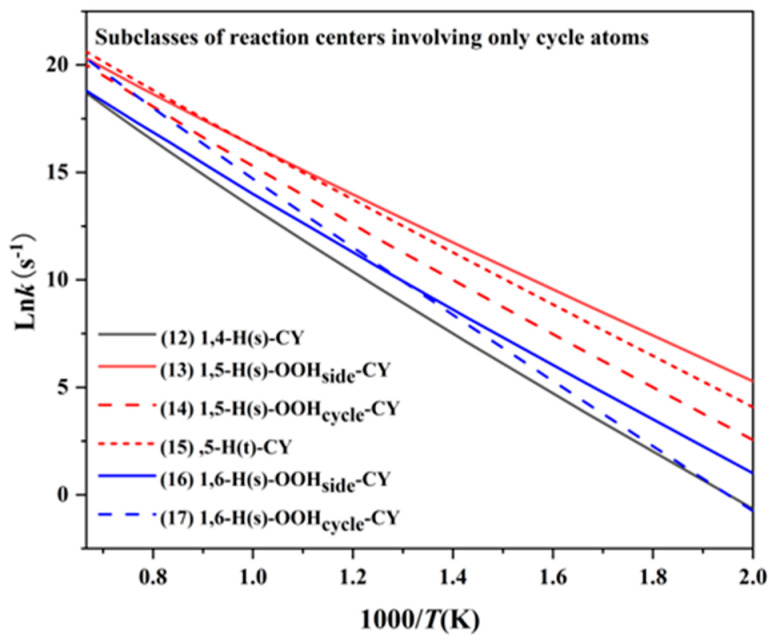
Comparison of the high−pressure limit rate rules between subclasses: reaction centers involve only cycle atoms.

When the reaction centers of subclasses involve only cycle atoms, the comparisons of the high-pressure limit rate constants for different subclasses are shown in Figure 3. It can be seen from Figure 3 that the average high-pressure limit rate constants for these subclasses exhibit the following trends: 1,5-H(s)-OOH_side_-CY > 1,5-H(s)-OOH_cycle_-CY > 1,6-H(s)-OOH_side_-CY > 1,6-H(s)-OOH_cycle_-CY > 1,4-H(s)-CY, indicating that for identical carbon types from which the hydrogen migrates, the rate constants vary depending on the number of atoms connected in the TS cycles, following this order: 1,5-H migration > 1,6-H migration > 1,4-H migration. In addition, from the trend of the rate constants of the subclasses 1,5-H(s)-OOH_side_-CY > 1,5-H(s)-OOH_cycle_-CY and 1,6-H(s)-OOH_side_-CY > 1,6-H(s)-OOH_cycle_-CY, it can be seen that when the numbers of the atom connected in the TS cycles is the same, the intramolecular H-migration reactions of •OOQOOH are more likely to occur on the alkyl side chain.

#### 2.4.2. Comparing the High-Pressure Limit Rate Constants with Reported Published Mechanisms

(1) **Subclasses of reaction centers containing alkyl side chains.**

**Figure 4:**
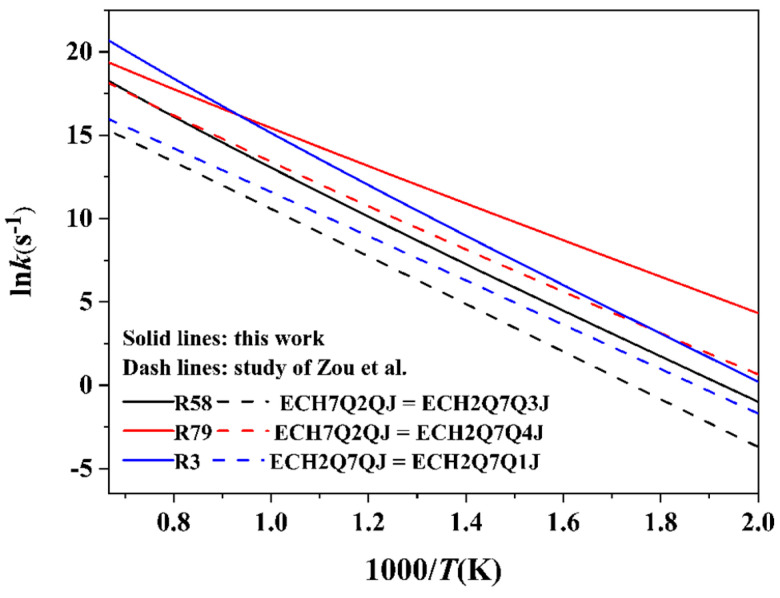
Comparison of the high−pressure limit rate constants for reactions R58, R79, and R3 in our study with the corresponding reactions ECH7Q2QJ = ECH2Q7Q3J, ECH7Q2QJ = ECH2Q7Q4J, and ECH2Q7QJ = ECH2Q7Q1J, respectively, as outlined in the ethyl cyclohexane mechanism developed by Zou et al. [31].

Figure 4 illustrates the comparisons of high-pressure limit rate constants calculated in our study with values reported in published combustion mechanisms. It is evident from the figures that our calculated values differ from the data provided by the published mechanisms for normal-alkyl cyclohexanes. Figure 4 illustrates the comparison of the high-pressure limit rate constants for the reactions R58, R79, and R3 investigated in this study with the corresponding reactions in the work by Zou et al. [31] across the temperature range of 500–1500 K. The resulting ratios are 10.7–19.3, 3.4–39.9, and 6.7–112.4, respectively.

#### 2.4.3. Comparison of High-Pressure Limit Rate Rules Between Normal-Alkyl Cyclohexanes and Non-Cyclic Alkane Systems

To investigate the substantial disparity between the high-pressure rate constants of the intramolecular H-migration reactions in the •OOQOOH class, specifically those transpiring in the side chain, and the corresponding values observed in non-cyclic systems, an analysis of the energy barriers and pre-exponential factors is conducted. This examination aims to shed light on the underlying reasons behind such pronounced discrepancies. Yao et al. [17] studied the intramolecular H-migration reactions of •OOQOOH in non-cyclic alkanes at the level of CBS-QB3. The intramolecular H-migration reactions of non-cyclic alkanes and normal-alkyl cycloalkanes with similar reaction types are selected and their energy barriers are compared. In this study, the energy barrier of R5 (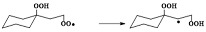) is 30.1 kcal mol^−1^, which is lower than the energy barrier of 31.5 kcal mol^−1^ calculated by Yao et al. for the analogous reaction in alkane (O2CCOO• → O2C•COO). The energy barrier of R42 (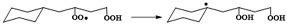) is 16.1 kcal mol^−1^, which is also lower than the energy barrier of 18.5 kcal mol^−1^ calculated by Yao et al. for the analogous reaction in alkane (O2C(C)CCOO• → O2C•(C)CCOO). Similarly, the energy barrier of R49 (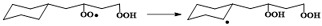) is 18.8 kcal mol^−1^, which is also lower than the energy barrier of 21.6kcal mol^−1^ calculated by Yao et al. for the analogous reaction in alkane (O2CCC(C)C(C)OO• → O2C•CC(C)C(C)OO).

However, different energy barriers will lead to a large difference in the rate constants of the intramolecular H-migration reactions of normal-alkyl cycloalkanes and non-cyclic alkanes with analogous reaction types, thus distinguishing the low temperature chemical reaction kinetics of normal-alkyl cyclohexane and non-cyclic alkanes. Moreover, the pre-exponential factors for non-cyclic alkanes and normal-alkyl cyclohexanes exhibit significant differences. This variance can be attributed to several factors, such as Eckart’s tunneling correction coefficients, reaction path degeneracy, and partition function values of transition states and reactants that account for translational, rotational, and vibrational movements.

### 2.5. Kinetic Modeling

At present, there are very few combustion mechanisms that involve both the H-migration of •OOQOOH with •P(OOH)_2_ product class and the H-migration of •OOQOOH with KHP and an OH radical product class for normal-alkyl cyclohexanes. In the low-temperature combustion mechanisms of ethyl cyclohexane constructed by Zou et al. [31] and n-propyl cyclohexane constructed by Liu et al. [32], the kinetic parameters of these two classes in their mechanisms are all approximately estimated from the analogous reactions in non-cyclic alkanes or from the analogous intramolecular H-migration reactions of ROO• in alkyl cyclohexanes; thus, the predictive powers of ethyl cyclohexane and n-propyl cyclohexane oxidation may be improved by replacing the rate constants of these two kinds of reactions with the calculated the high-pressure limit rate constants in this work. Therefore, we modified the mechanisms by replacing the rate constants for the H-migration reactions of •OOQOOH to the •P(OOH)_2_ product at temperatures above 500 K with our calculated results. Additionally, the thermodynamic data for the species involved in these reactions have been updated with our calculated results. The high-pressure limit rate constants for these reactions in both the updated and original mechanisms are listed in Tables S3 and S4 of Supplemental Material—File S1.

The simulation of ethyl cyclohexane oxidation in a jet-stirred reactor (JSR) involves employing the perfectly stirred reactor model within the Chemkin-Pro (15092) [44] software. The simulation of ignition delay times (IDTs) for n-propyl cyclohexane oxidation in a high-pressure shock tube (HPST) is carried out by employing the closed homogeneous model within the Chemkin-Pro [44] software.

**Figure 5:**
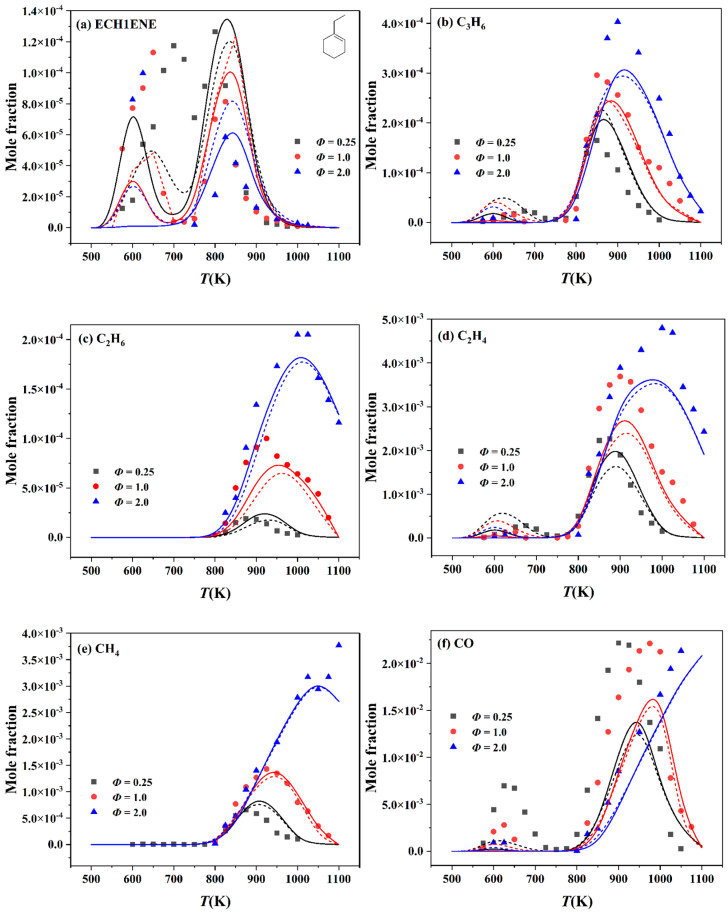
Comparison of the concentration profiles for major species in ethyl cyclohexane oxidation with equivalence ratios: (■) *ϕ* = 0.25, our modified mechanism (black lines), original mechanism constructed by Zou et al. [31] (black dash lines), and experiments (symbols) by Husson et al. [45]; (●) *ϕ* = 1.0, our modified mechanism (red lines), original mechanism constructed by Zou et al. [31] (red dash lines), and experiments (symbols) by Husson et al. [45]; (▲) *ϕ* = 2.0; our modified mechanism (blue lines), mechanism constructed by Zou et al. (blue dash lines), and experiments (symbols) by Husson et al.

Concentration profiles of certain species are crucial for understanding heat release and pollutant emission during ethyl cyclohexane oxidation. Husson et al. [45] measured the concentration profiles of key species in a jet-stirred reactor experiment over a temperature range of 500–1100 K, with equivalence ratios (the ratio of the actual mixture ratio of ethyl cyclohexane and oxidant to the stoichiometric ratio required for complete combustion) of 0.25, 1.0, and 2.0, at 800 Torr and a residence time of 2.0 s. To replicate these conditions, the concentrations of these important species were simulated using the perfectly stirred reactor model under isothermal and isobaric assumptions, matching the experimental setup. Figure 5 illustrates the comparisons between simulated results for certain predominant species obtained from both the original and updated mechanisms, alongside experimental data, across equivalence ratios of 0.25, 1.0, and 2.0.

Based on the analysis of the figures, several observations can be made regarding the performance of the original model compared to the updated model. Figure 5a–d show that the updated model slightly improves the predictions compared to the original model for equivalence ratios of 0.25, 1.0 and 2.0. Figure 5e,f show that the updated model outperforms the original model for both equivalent ratios of 0.25 and 1.0, while there is minimal discrepancy between the two models for an equivalence ratio of 2.0. It is important to note that updating a combustion mechanism with newly accurately calculated rate constants for certain reactions does not necessarily guarantee improved modeling results. This is due to the complexity of combustion, which encompasses numerous reactions and intricate interdependencies. Merely updating the rate constants of a few reactions may fail to account for the detailed interactions and feedback effects within the combustion system. Furthermore, combustion involves a multitude of simultaneous reactions, and focusing solely on a subset of rate constants may not adequately capture the overall behavior of the system.

**Figure 6:**
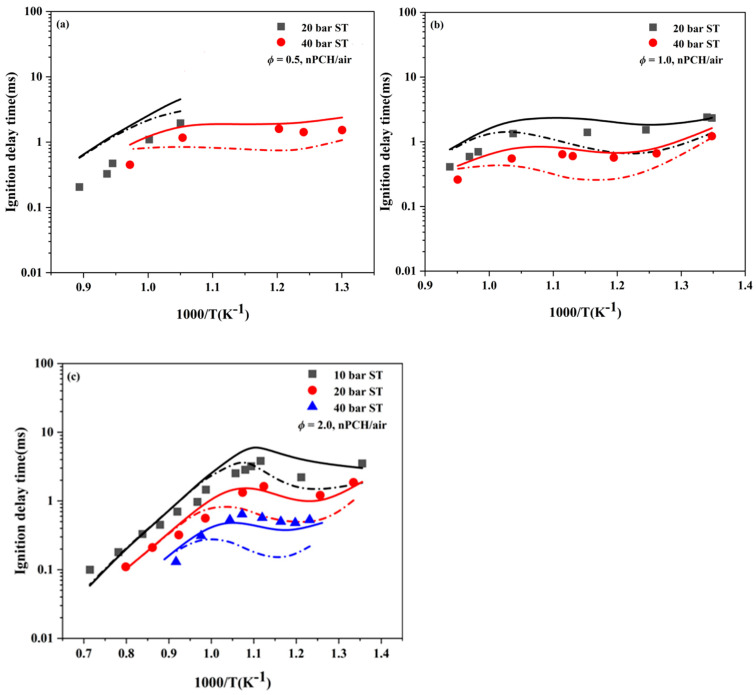
Comparison of nPCH/air ignition delay times predicted from both the original and the current updated mechanisms, measured in the HPST experiments of the work by Liu et al. [32], and reported HPST data by Ahmed et al. [46]. (**a**) *ϕ* = 0.5 [46]; (**b**) *ϕ* = 1.0 [46]; (**c**) *ϕ* = 2.0 [32]. (Solid lines: the current updated mechanism predictions; dash lines: the original mechanism predictions.)

To comprehensively understand the combustion properties and chemical kinetics of n-propyl cyclohexane (nPCH), IDT measurements of nPCH/air mixtures were conducted in a high-pressure shock tube (HPST) under fuel-rich conditions (ϕ = 2.0), with pressures ranging from 10 to 40 bar and temperatures between 738 and 1400 K, as reported by Liu et al. [32]. Previous experimental investigations on n-propyl cyclohexane, including IDTs at equivalence ratios of ϕ = 0.5 and 1.0, can also be found in the work by Ahmed et al. [46]. To assess whether the intramolecular H-migration reactions of •OOQOOH studied in this work affect the overall fuel characteristics due to chain branching, we performed simulations of IDTs for both the updated mechanism and the original mechanism under the experimental conditions established by Liu et al. [32] and Ahmed et al. [46]. Figure 6 presents comparisons of the simulated IDT results for nPCH/air mixtures predicted by both the original and updated mechanisms, alongside the experimental data across equivalence ratios of 0.5, 1.0, and 2.0. Figure 6a–c demonstrate that the updated mechanism provides improved prediction results compared to the original mechanism under most conditions for equivalence ratios of 0.5, 1.0, and 2.0. This indicates that incorporating the newly calculated rate constants for the intramolecular H-migration reactions of •OOQOOH can enhance the modeling results to some extent.

## 3. Materials and Methods

A set of 110 representative reactions (R1–R110) for the studied system were selected, and the reaction list is shown in Table S1 of the Supplemental Material—File S1. In the realm of chemical kinetics, particularly for reactions involving energized intermediates like those formed in unimolecular decompositions or recombination reactions, the observed rate constant often exhibits a crucial dependence on the total pressure of the system. This pressure dependence arises fundamentally from the competition between the chemical transformation of the energized intermediate (e.g., isomerization or dissociation) and its collisional deactivation (stabilization) by the surrounding bath gas molecules. High-pressure limit rate constants are defined as the pressure-independent limits of the reaction rate constant. They are achieved under conditions where the pressure (and consequently, the concentration of the bath gas) is sufficiently high. Under these high-pressure conditions, collisional deactivation of the energized intermediates by the bath gas becomes extremely rapid and efficient. This rapid deactivation has a profound consequence: it maintains the population of the energized species in a state of thermal equilibrium with the surrounding environment. This concept is fundamental to unimolecular reaction theory [47]. In this study, high-pressure limit rate constants for reactions with transition states are calculated with transition state theory (TST). Electronic structures are calculated using the computationally inexpensive CBS-QB3 [48] composite method using Gaussian 09 software [49]. The CBS-QB3 method is a composite method, in which geometries and vibrational frequency (scaled by a factor of 0.99) are calculated at B3LYP/CBSB7 level of theory. In addition, the intrinsic reaction coordinates (IRCs) are used for the transition state conformation at the same B3LYP/CBSB7 level to determine the optimized conformation of the intermediate transition state connecting the reactants to the products. The CBS-QB3 method was the first comprehensive fundamentally based study of the isomerization and association of second O_2_ chemistry for real fuel models as large as n-pentane by Asatryan and Bozzelli [50]. It is worth noting that while the CBS-QB3 method generally provides reliable results, it may have limitations in accurately calculating energy barriers in certain cases [51]. Specifically, reference [51] demonstrates these limitations become particularly apparent for (1) reactions involving strained cyclic transition states (as shown for cyclic ether formation from hydroperoxyalkyl radicals), (2) systems with significant multi-reference character, and (3) reactions where post-CCSD(T) correlation effects are important. However, it is worth noting that the CBS-QB3 method offers significant advantages in terms of computational efficiency compared to more precise methods like CCSD(T). It allows for the calculation of relatively large reaction systems in a reasonable amount of time. In the field of combustion kinetics, where numerous reactions need to be analyzed, striking the right balance between accuracy and computational speed is crucial. This is why the CBS-QB3 method is widely adopted as a go-to approach. However, it is important to validate its results and assess its applicability when employing it in practical applications. This method has been widely used for the calculations of electronic energies [52,53,54,55] and it is shown that the energy uncertainty is less than 1.5 kcal mol^−1^. It has been proven that the CBS-QB3 combination method can correctly calculate the kinetics of the reactions in cycloalkanes [23,28,29,56,57,58]. The rotational and vibrational partition functions are determined using the rigid-rotor harmonic oscillator (RRHO) approximation. In this study, low-frequency vibrations corresponding to torsions about a single bond for all reactants and transition states are treated with one-dimensional (1-D) hindered internal rotors model, following the Pitzer and Gwinn method [59], where the potentials of each internal rotation are scanned at the B3LYP/CBSB7 level of theory with an interval of 10 of the dihedral angle. For the transition states, internal rotor scans can perform by freezing the atoms involved in the reaction centers and by taking into account the remaining torsions. These corrections account for the deviations from the idealized harmonic motion in the molecule. At the B3LYP/CBSB7 level, the hindrance potentials are obtained by relaxed scans of the dihedral angle with an increment of 10 degrees (more details can be found in Figure S1a–c of the Supplemental Material—File S1).

The TST for calculating high-pressure limit rate constants has been derived using classical mechanics; however, for many reactions, quantum effects—particularly zero-point energy and tunneling—cannot be ignored [60]. To incorporate the effects of zero-point energy in TST calculations, one can replace the classical partition function with a quantum mechanical partition function that incorporates the discrete energy levels. Quantum mechanical tunneling enables reactions to occur even when the reactants lack sufficient energy to overcome the transition state barrier. While the TST assumes classical behavior and neglects tunneling effects, incorporating a tunneling correction is essential for accurately predicting rate constants in cases involving large kinetic isotope effects or lighter reactants, such as H-migration reactions, especially at low temperatures [61]. Several methods for tunneling correction can be applied to calculate rate constants, including the Wigner correction, the Eckart correction, and the small-curvature tunneling correction. Each method has its own mathematical formulation and assumptions about the reaction system. In this study, we employed the asymmetric Eckart method to account for the tunneling effect [62]. The Eckart correction is based on the assumption that the tunneling process is governed by a two-dimensional anharmonic potential. The calculated barrier width, denoted as L, for all the reactions examined in our research, is presented in Table S2 of the Supplemental Material—File S1 for reference and comprehensive evaluation. This methodological inclusion allows us to appropriately account for the influence of tunneling on the computed reaction rates.

Transition state theory (TST) is usually sufficient for reactions with an apparent transition state. These reactions involve a defined energy barrier that separates the reactants from the products, allowing TST to be used to calculate reaction rates based on the properties of the transition state. In this study, all studied intramolecular H-migration reactions of •OOQOOH possessed apparent transition states and hence traditional TST was employed.

In this study, all rate constants were calculated using the ChemRate software (version 1.5.8) [63]. The (*A*, *n*, *E*) parameters within the modified Arrhenius equation were employed to characterize the temperature dependence of the rate constants. These parameters can also be utilized in practical modeling software like Chemkin-Pro [44]. The calculated enthalpies, entropies, and heat capacities for all species in this work are provided in NASA format with 14 parameters, detailed in Supplemental Material—File S3. Finally, the rate constants calculated between 500 K and 1500 K were fitted to the three-parameter Arrhenius expression [64],

$$k(T)=AT^{n}exp(-\frac{E}{RT})$$

where *A* is the pre-exponential factor, *n* is the temperature exponent, and *Ea* is the activation energy.

## 4. Conclusions

In this study, energy barriers and high-pressure limit rate constants for intramolecular H-migration reactions of •OOQOOH across temperatures ranging from 500 to 1500 K are calculated. Our findings indicate significant variation in these values among different reactions. Hence, it is essential to classify these reactions into distinct subclasses and formulate specific rate rules for each subclass. The studied reactions are divided into 15 subclasses according to the reaction center and high-pressure limit rate rules for all subclasses are developed. This work has conducted a thorough analysis of the uncertainty factor values for the rate rules of normal-alkyl cyclohexanes. The results indicate that these values are generally within one order of magnitude for most subclasses, suggesting that the classification and rate rules proposed in this work are logical and acceptable. Consequently, these findings lay the groundwork for the automatic generation of mechanisms for normal-alkyl cyclohexanes.

To validate the calculated rate constants, a comparison is made with kinetic data from detailed mechanisms found in the literature. These mechanisms typically approximate non-cyclic alkanes or normal-alkyl cyclohexanes with short alkyl side chains, such as cyclohexane, methylcyclohexane, or ethyl cyclohexane. Furthermore, the calculated rate constants of specific reactions are compared with kinetic data obtained from analogous classes in non-cyclic alkane systems. The striking observation from these comparisons is the significant differences, often reaching several orders of magnitude, between the calculated rate constants in this work and those reported in the literature. This discrepancy highlights the critical need for more accurate kinetic calculations specifically tailored for the H-migration of •OOQOOH reactions in normal-alkyl cyclohexanes.

Furthermore, this work presents newly calculated kinetic data for selected reactions, which are incorporated into the comprehensive oxidation model for ethyl cyclohexane and n-propyl cyclohexane. Utilizing information from previous studies, we simulated the concentration profiles of key species during the low temperature combustion of ethyl cyclohexane in a jet-stirred reactor, as well as the ignition delay times for n-propyl cyclohexane oxidation in a high-pressure shock tube, using Chemkin-Pro software. The simulation results indicate that, compared to the original mechanisms, the predictions obtained from the updated mechanisms align more closely with the experimental data under certain conditions.

## Figures and Tables

**Table 1:** A summary of the reaction subclasses in the H-migration of •OOQOOH.

Reactions with •P(OOH)_2_ Products	
Reaction Center Contains Side Chain	Reaction Center Involves Only Cycle Atoms
(1) 1,4 ^a^-H(p) ^b^-SC ^c^	(10) 1,4-H(s)-CY ^d^
(2) 1,4-H(s)-SC	(11) 1,5-H(s)-OOH_side_^e^-CY
(3) 1,4-H(t)-SC	(12) 1,5-H(s)-OOH_cycle_^f^-CY
(4) 1,5-H(p)-SC	(13) 1,5-H(t)-CY
(5) 1,5-H(s)-SC	(14) 1,6-H(s)-OOH_side_-CY
(6) 1,5-H(t)-SC	(15) 1,6-H(s)-OOH_cycle_-CY
(7) 1,6-H(p)-SC	
(8) 1,6-H(s)-SC	
(9) 1,6-H(t)-SC	

^a^ Representation of the number of atoms connected in the TS cycles. ^b^ Representation of the types of carbons to which the hydrogen atom is migrated in •OOQOOH. ^c^ Representation of the reaction center containing a side chain. ^d^ Representation of the reaction center involving only cycle atoms. ^e^ Representation of the positions of the -OOH moiety in •OOQOOH resides within the side chain. ^f^ Representation of the positions of the -OOH moiety in •OOQOOH resides within the cycle.

**Table 2:** Comparison of energies between chair and boat conformations for species involved in reactions R5 and R57. (The unit of E is kcal mol^−1^.)

Species	Electronic Energy		
	*E*_chair_	*E*_boat_	Δ(*E*_boat_ − *E*_chair_)
R5	−385,024.3	−385,013.2	11.1
TS5	−384,994.2	−384,984.1	10.1
P5	−385,012.3	−385,001.3	11.0
R57	−360,393.6	−360,405.1	11.5
TS57	−360,366.8	−360,374.6	7.8
P57	−360,387.3	−360,392.0	4.6

**Table 3:** Comparison of the energy barriers for reaction R57 with and without hydrogen bond conformation (the TS reaction centers are marked in green) (unit: kcal mol^−1^).

	Reactants		Transition States		Energy Barriers
(1)	No HB	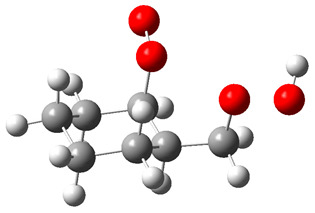	No HB	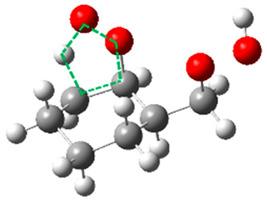	31.7
(2)	HB	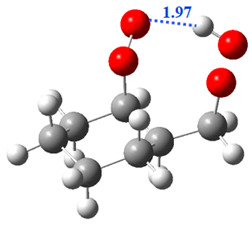	No HB	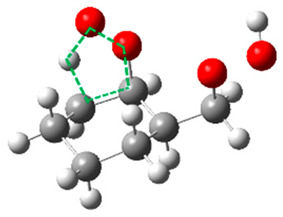	32.2
(3)	No HB	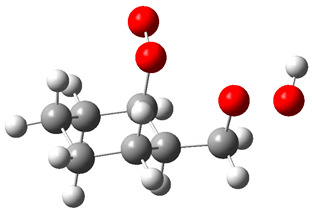	HB	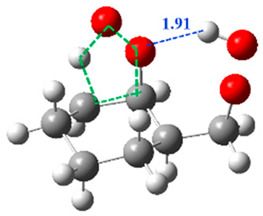	29.9
(4)	HB	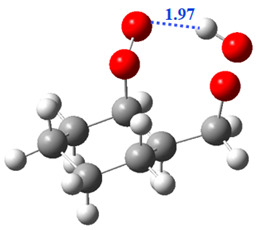	HB	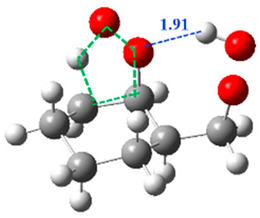	30.5

**Table 4:** Energy barriers for the reactions with •P(OOH)_2_ product when the reaction center containing the alkyl side chain (kcal mol^−1^).

Subclasses	No.	Reactions	Energy Barriers
**(1) 1,4-H(p)-SC**	R1	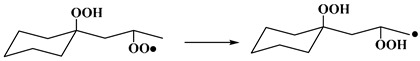	34.8
	R2	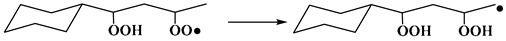	34.8
	R3	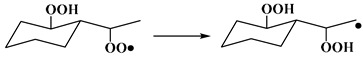	32.3
	R4	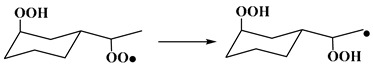	35.2
			**34.3 ^a^ (2.9 ^b^)**
**(2) 1,4-H(s)-SC**	R5	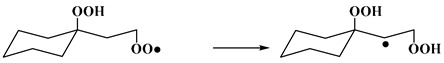	30.1
	R6	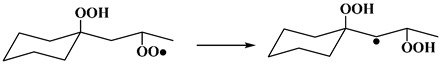	30.6
	R7	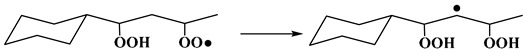	30.7
	R8	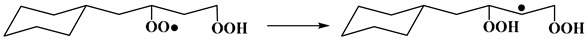	30.9
	R9	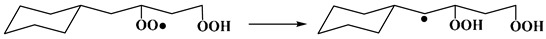	30.9
	R10	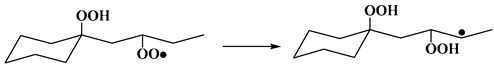	31.2
	R11	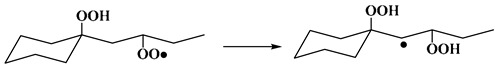	31.8
	R12	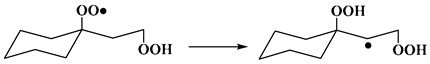	31.3
	R13	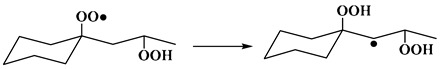	31.3
	R14	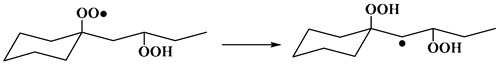	31.2
	R15	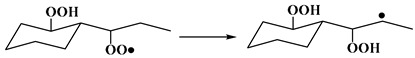	27.7
	R16	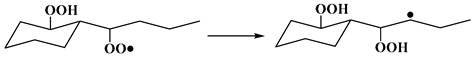	28.5
	R17	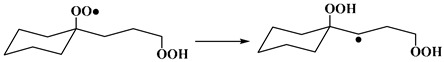	27.7
	R18	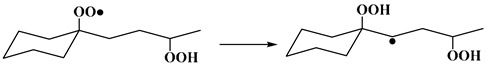	28.3
	R19	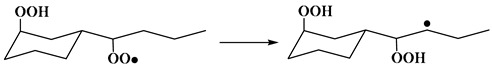	31.0
			**30.2 (4.1)**
**(3) 1,4-H(t)-SC**	R20	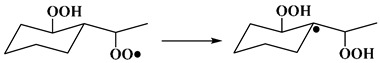	27.1
	R21	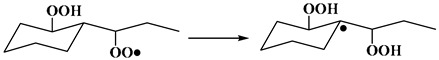	26.9
	R22	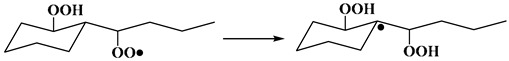	27.7
	R23	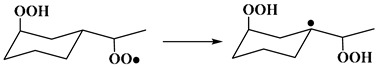	25.9
	R24	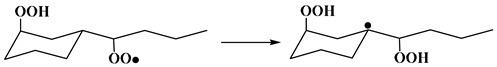	25.7
			**26.7 (2.0)**
**(4) 1,5-H(p)-SC**	R25	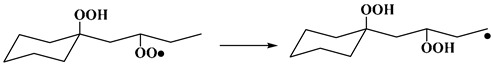	24.0
	R26	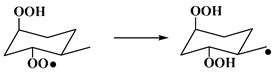	23.6
	R27	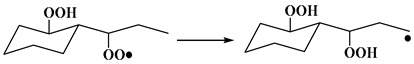	20.4
			**22.7 (3.6)**
**(5) 1,5-H(s)-SC**	R28	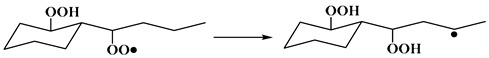	19.7
	R29	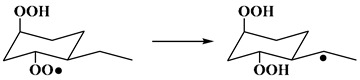	20.1
	R30	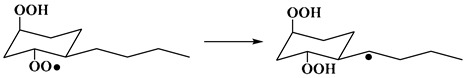	20.4
	R31	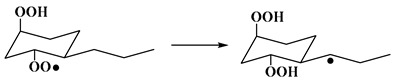	19.6
	R32	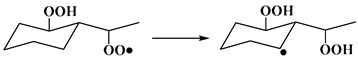	20.9
	R33	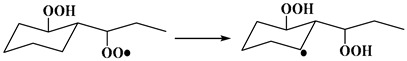	21.2
	R34	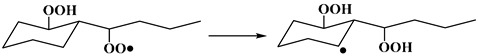	21.9
	R35	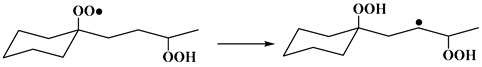	20.4
	R36	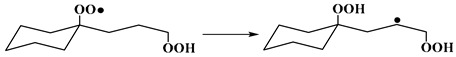	20.2
	R37	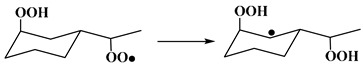	23.4
	R38	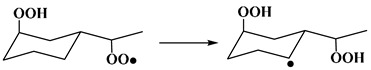	23.3
	R39	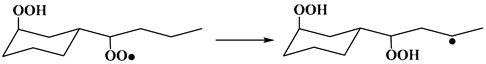	23.2
	R40	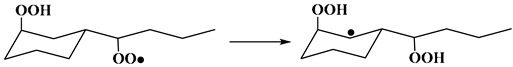	23.0
	R41	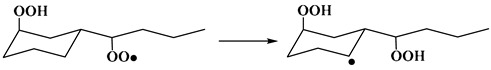	23.1
			**21.5 (3.8)**
**(6) 1,5-H(t)-SC**	R42	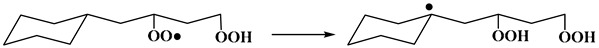	16.1
			**16.1 (0.0)**
**(7) 1,6-H(p)-SC**	R43	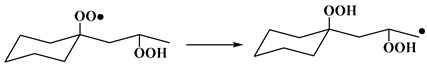	25.4
	R44	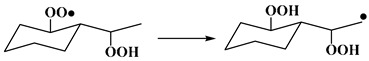	22.4
	R45	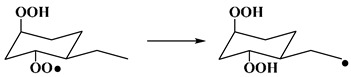	23.3
	R46	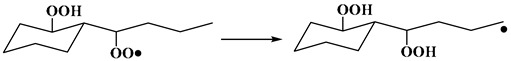	20.9
	R47	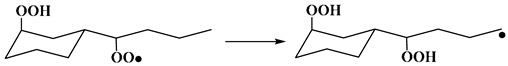	23.5
			**23.1 (4.5)**
**(8) 1,6-H(s)-SC**	R48	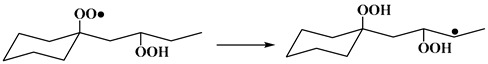	21.3
	R49	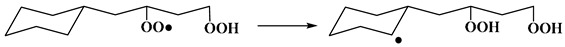	18.8
	R50	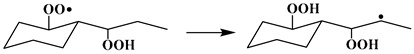	18.7
	R51	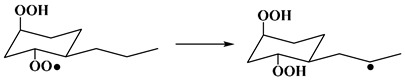	19.3
	R52	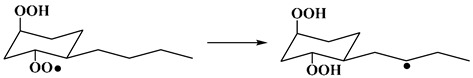	19.8
	R53	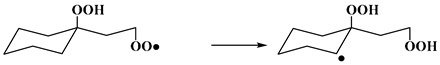	21.2
	R54	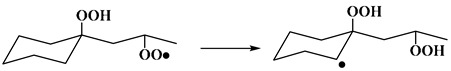	22.0
	R55	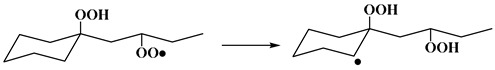	21.9
			**20.4 (3.3)**
**(9) 1,6-H(t)-SC**	R56	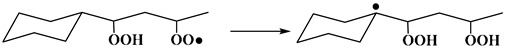	19.8
			**19.8 (0.0)**

^a^ The average energy barrier of reactions in a subclass. ^b^ The maximum deviation of energy barriers in a subclass.

**Table 5:** Energy barriers for the reactions with •P(OOH)_2_ product when the reaction center involves only cycle atoms (kcal mol^−1^).

Subclasses	No.	Reactions	Energy Barriers
**(10) 1,4-H(s)-CY**	R57	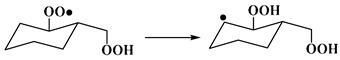	30.5
	R58	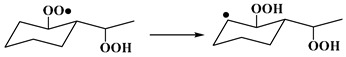	30.5
	R59	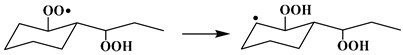	29.9
	R60	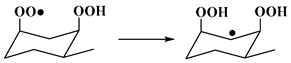	32.9
	R61	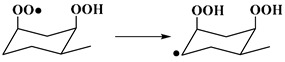	32.9
	R62	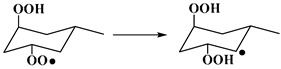	33.0
	R63	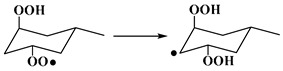	33.1
	R64	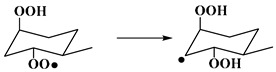	32.9
	R65	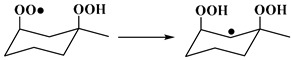	33.1
	R66	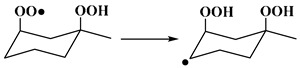	32.6
	R67	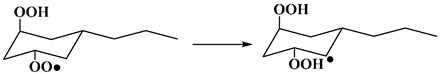	32.3
	R68	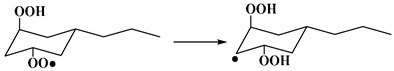	33.1
	R69	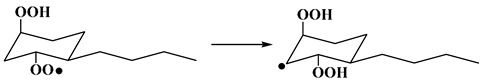	33.7
	R70	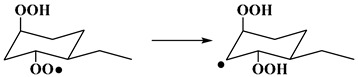	32.8
	R71	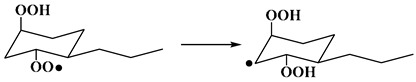	32.9
	R72	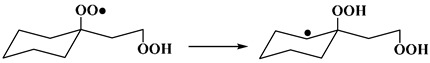	32.9
	R73	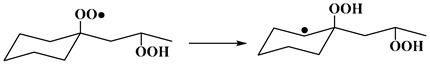	32.6
	R74	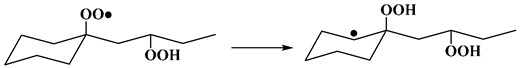	31.2
	R75	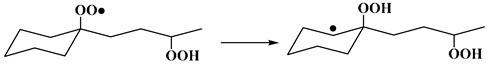	28.7
	R76	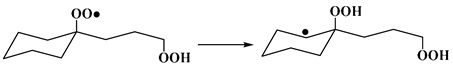	28.7
			**32.1 ^a^ (5.0 ^b^)**
**(11) 1,5-H(s)-OOH_side_-CY**	R77	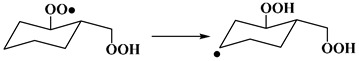	23.9
	R78	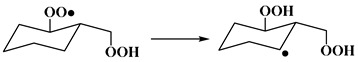	23.4
	R79	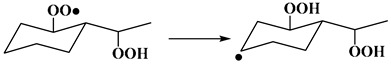	23.7
	R80	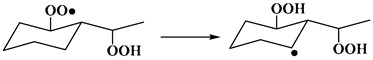	22.9
	R81	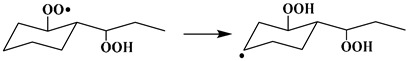	21.5
	R82	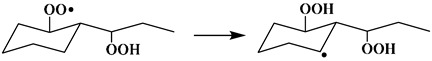	21.6
	R83	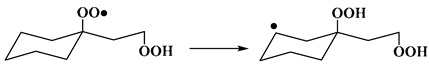	26.1
	R84	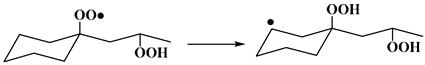	25.0
	R85	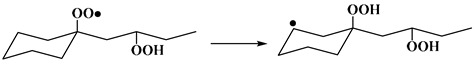	26.3
	R86	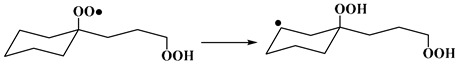	23.1
	R87	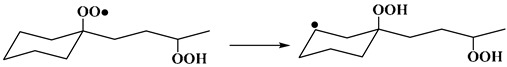	24.8
			**23.8 (4.8)**
**(12) 1,5-H(s)-OOH_cycle_-CY**	R88	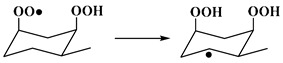	28.9
	R89	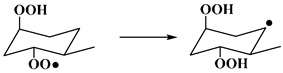	27.5
	R90	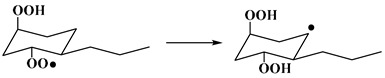	27.0
	R91	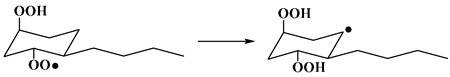	27.8
	R92	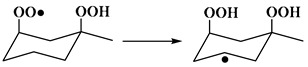	28.4
	R93	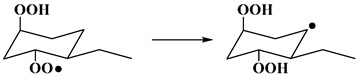	27.2
			**27.8 (1.9)**
**(13) 1,5-H(t)-CY**	R94	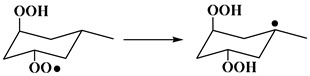	26.1
	R95	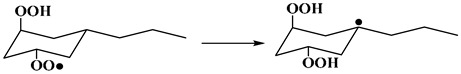	25.7
			**25.9 (0.4)**
**(14) 1,6-H(s)-OOH_side_-CY**	R96	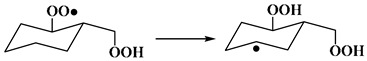	27.3
	R97	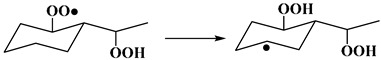	26.7
	R98	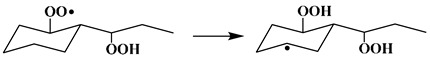	27.2
	R99	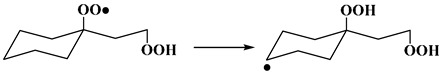	30.5
	R100	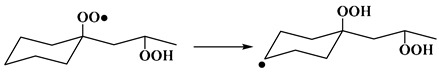	30.2
	R101	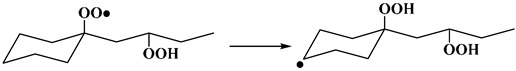	30.2
	R102	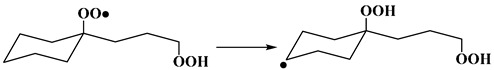	26.9
	R103	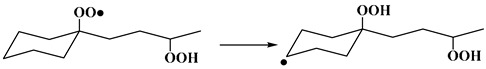	27.4
			**28.3 (3.8)**
**(15) 1,6-H(s)-OOH_cycle_-CY**	R104	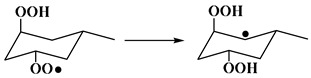	32.9
	R105	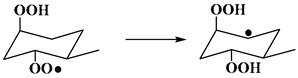	31.7
	R106	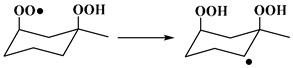	32.8
	R107	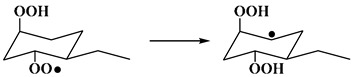	31.6
	R108	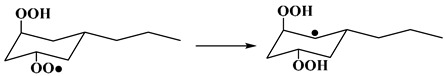	32.4
	R109	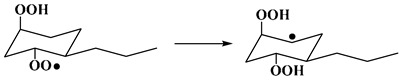	31.6
	R110	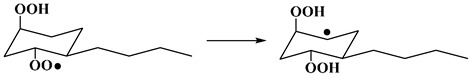	31.5
			**32.1 (1.4)**

^a^ The average energy barrier of reactions in a subclass. ^b^ The maximum deviation of energy barriers in a subclass.

**Table 6:** Calculated rate constants and derived rate rules in each subclass for the reactions with the •P(OOH)_2_ product when the reaction center contains the alkyl side chain at high pressure.

	Modified Arrhenius Parameters			*T* = 800 K	
Reaction	*A* (s^−1^)	*n*	*E* (cal mol^−1^)	*k* (s^−1^)	*k*/*k_ave_* ^a^
R1	1.03 × 10^3^	−5.5	39,223.6	2.38 × 10^3^	0.1
R2	1.43 × 10^0^	3.5	26,907.7	2.84 × 10^3^	0.2
R3	1.44 × 10^2^	3.3	25,103.5	5.65 × 10^4^	3.5
R4	1.17 × 10^−1^	3.9	26,493.9	2.44 × 10^3^	0.2
**(1) 1,4-H(p)-SC**	**1.04 × 10^1^**	**3.4**	**25,002.9**	**1.60** ** × 10^4^**	**19.9 ^#^**
R5	1.32 × 10^3^	2.8	23,615.1	6.46 × 10^4^	1.8
R6	1.32 × 10^0^	3.5	23,525.8	8.42 × 10^3^	0.2
R7	1.30 × 10^1^	3.2	23,489.2	9.81 × 10^3^	0.3
R8	2.29 × 10^1^	3.2	23,752.6	1.53 × 10^4^	0.4
R9	2.07 × 10^2^	2.9	24,261.5	9.92 × 10^3^	0.3
R10	1.07 × 10^2^	3.2	24,309.1	4.36 × 10^4^	1.2
R11	5.28 × 10^2^	3.0	25,007.4	2.94 × 10^4^	0.8
R12	1.51 × 10^2^	3.1	24,140.7	5.22 × 10^4^	1.5
R13	5.88 × 10^0^	3.4	23,799.6	1.49 × 10^4^	0.4
R14	1.88 × 10^3^	2.7	2,4806	2.46 × 10^4^	0.7
R15	1.14 × 10^3^	2.9	21,443.3	1.37 × 10^5^	3.9
R16	2.09 × 10^3^	2.5	22,445.5	2.97 × 10^4^	0.8
R17	4.30 × 10^2^	2.6	20,074.1	5.60 × 10^4^	1.6
R18	3.19 × 10^2^	2.7	21,694.9	2.65 × 10^4^	0.8
R19	2.25 × 10^0^	3.2	21,506	5.49 × 10^3^	0.2
**(2) 1,4-H(s)-SC**	**2.28** × 10^−1^	**3.8**	**20,847.9**	**3.52** × 10^4^	**25.0**
R20	6.84 × 10^4^	2.6	21,411.6	4.17 × 10^6^	1.6
R21	4.15 × 10^3^	3.0	20,725.3	3.94 × 10^6^	1.5
R22	6.29 × 10^3^	3.0	20,730.7	4.59 × 10^6^	1.7
R23	4.77 × 10^3^	2.3	19,937.6	2.88 × 10^5^	0.1
R24	7.88 × 10^4^	1.9	20,524.6	2.49 × 10^5^	0.1
**(3) 1,4-H(t)-SC**	**6.31 × 10^3^**	**2.9**	**20,893.1**	**2.65** ** × 10^6^**	**18.4**
R25	3.75 × 10^6^	1.8	20,203.9	2.25 × 10^6^	0.4
R26	1.01 × 10^6^	1.9	19,779	1.07 × 10^6^	0.2
R27	6.42 × 10^6^	1.7	17,492.2	1.21 × 10^7^	2.4
**(4) 1,5-H(p)-SC**	**1.07 × 10^5^**	**2.2**	**17,314.6**	**5.15 × 10^6^**	**11.4**
R28	3.58 × 10^9^	0.6	16,301	5.25 × 10^6^	1.3
R29	1.54 × 10^7^	1.4	16,675.8	6.47 × 10^6^	1.6
R30	1.16 × 10^6^	1.9	16,530.9	9.34 × 10^6^	2.3
R31	2.20 × 10^7^	1.5	16,129.9	9.49 × 10^6^	2.3
R32	2.23 × 10^8^	1.2	17,494.1	9.56 × 10^6^	2.3
R33	2.70 × 10^6^	1.7	17,128.1	5.57 × 10^6^	1.4
R34	1.37 × 10^8^	1.1	17,962.8	2.77 × 10^6^	0.7
R35	1.09 × 10^6^	1.6	16,346.1	1.99 × 10^6^	0.5
R36	1.45 × 10^6^	1.6	16,352.5	2.64 × 10^6^	0.6
R37	1.11 × 10^6^	1.8	19,452.7	7.86 × 10^5^	0.2
R38	1.04 × 10^6^	1.8	19,282.3	1.14 × 10^6^	0.3
R39	1.69 × 10^6^	1.6	19,263.5	4.74 × 10^5^	0.1
R40	5.09 × 10^5^	1.8	18,859.8	5.37 × 10^5^	0.1
R41	2.22 × 10^6^	1.7	19,069.4	1.43 × 10^6^	0.3
**(5) 1,5-H(s)-SC**	**8.27 × 10^5^**	**1.8**	**16,262.7**	**4.10** ** × 10^6^**	**20.2**
R42	4.61 × 10^6^	1.5	12,886.3	4.22 × 10^7^	1.0
**(6) 1,5-H(t)-SC**	**4.61 × 10^6^**	**1.5**	**12,886.3**	**4.22** × 10^7^	**1.0**
R43	2.03 × 10^6^	1.9	21,852.2	9.00 × 10^5^	1.1
R44	3.65 × 10^3^	2.3	18,974	2.59 × 10^5^	0.3
R45	9.45 × 10^4^	2.0	18,890.6	3.06 × 10^5^	0.4
R46	9.92 × 10^6^	1.5	17,272.6	2.47 × 10^6^	3.0
R47	2.14 × 10^5^	1.8	19,498.7	1.35 × 10^5^	0.2
**(7) 1,6-H(p)-SC**	**5.94 × 10^1^**	**3.0**	**15,968.2**	**8.14** ** × 10^5^**	**18.3**
R48	4.03 × 10^8^	1.3	18,582.4	2.32 × 10^7^	1.6
R49	2.61 × 10^5^	1.7	14,855.9	2.38 × 10^6^	0.2
R50	8.06 × 10^6^	1.7	17,325.9	1.41 × 10^7^	1.0
R51	1.92 × 10^5^	2.0	14,936.6	7.94 × 10^6^	0.6
R52	1.35 × 10^6^	1.7	15,890.3	5.19 × 10^6^	0.4
R53	1.64 × 10^7^	1.7	18,217.9	1.15 × 10^7^	0.8
R54	2.79 × 10^5^	2.2	18,906.4	4.46 × 10^6^	0.3
R55	5.08 × 10^11^	0.1	19,946.5	2.84 × 10^6^	0.2
**(8) 1,6-H(s)-SC**	**2.48 × 10^5^**	**2.1**	**16,692.7**	**1.43** × 10^7^	**9.7**
R56	1.19 × 10^7^	1.5	16,540.4	7.37 × 10^6^	1.0
**(9) 1,6-H(t)-SC**	**1.19 × 10^7^**	**1.5**	**16,540.4**	**7.37 × 10^6^**	**1.0**

^#^ *f* = *k*_max_/*k*_min_ in each subclass. ^a^ *k_ave_* is the average rate constants at 800 K in each reaction subclass.

**Table 7:** Calculated rate constants and derived rate rules in each subclass for the reactions with the •P(OOH)_2_ product when the reaction center involves only cycle atoms at high pressure.

	Modified Arrhenius Parameters			*T* = 800 K	
Reaction	*A* (s^−1^)	*n*	*E* (cal mol^−1^)	*k* (s^−1^)	*k/k_ave_* ^a^
R57	2.83 × 10^3^	2.7	24,279.3	5.08 × 10^4^	3.3
R58	1.05 × 10^2^	3.0	23,839.1	1.20 × 10^4^	0.8
R59	9.88 × 10^2^	2.8	23,306.5	6.24 × 10^4^	4.1
R60	5.19 × 10^1^	3.2	25,740.6	6.81 × 10^3^	0.4
R61	7.52 × 10^1^	3.1	25,766.7	5.77 × 10^3^	0.4
R62	5.21 × 10^1^	3.2	25,824.7	6.50 × 10^3^	0.4
R63	5.23 × 10^1^	3.2	25,814.2	6.62 × 10^3^	0.4
R64	4.23 × 10^1^	3.1	25,558.5	6.08 × 10^3^	0.4
R65	3.86 × 10^1^	3.2	25,738.9	9.57 × 10^3^	0.6
R66	6.40 × 10^1^	3.2	25,449.9	1.15 × 10^4^	0.7
R67	8.34 × 10^1^	3.1	25,135.3	1.53 × 10^4^	1.0
R68	2.24 × 10^1^	3.3	25,625.3	7.03 × 10^3^	0.5
R69	1.11 × 10^1^	3.3	25,619.1	3.52 × 10^3^	0.2
R70	6.36 × 10^1^	3.2	25,488.4	1.06 × 10^4^	0.7
R71	5.44 × 10^1^	3.2	25,476.1	1.06 × 10^4^	0.7
R72	1.22 × 10^2^	3.0	26,018	5.22 × 10^3^	0.3
R73	6.64 × 10^6^	1.8	29,064	9.84 × 10^3^	0.6
R74	3.43 × 10^3^	2.5	26,498.1	4.95 × 10^3^	0.3
R75	9.71 × 10^1^	2.9	21,773.6	3.28 × 10^4^	2.1
R76	8.75 × 10^1^	2.9	21,710.7	3.02 × 10^4^	2.0
**(10) 1,4-H(s)-CY**	**7.09 × 10^-2^**	**3.9**	**22,409.4**	**1.54 × 10^4^**	**17.7 ^#^**
R77	9.50 × 10^5^	1.9	19,641.8	1.72 × 10^6^	2.6
R78	5.01 × 10^5^	1.9	19,151.5	1.10 × 10^6^	1.7
R79	8.77 × 10^6^	1.4	20,139	2.92 × 10^5^	0.4
R80	6.45 × 10^5^	1.7	18,860	4.10 × 10^5^	0.6
R81	2.14 × 10^5^	1.9	18,981.2	5.20 × 10^5^	0.8
R82	5.35 × 10^5^	1.9	18,479.5	1.69 × 10^6^	2.5
R83	3.43 × 10^5^	2.0	21,779.8	1.98 × 10^5^	0.3
R84	1.21 × 10^5^	2.0	20,609.7	2.32 × 10^5^	0.3
R85	6.15 × 10^5^	1.9	22,412.8	1.78 × 10^5^	0.3
R86	1.66 × 10^5^	2.0	18,681.5	8.81 × 10^5^	1.3
R87	5.13 × 10^4^	2.0	20,338.8	9.00 × 10^4^	0.1
**(11) 1,5-H(s)-OOH_side_-CY**	**4.33 × 10^4^**	**2.2**	**18,830.5**	**6.65 × 10^5^**	**19.1**
R88	6.26 × 10^4^	2.2	24,236.7	3.78 × 10^4^	0.2
R89	1.86 × 10^5^	2.0	23,082.5	7.73 × 10^4^	0.5
R90	8.68 × 10^1^	3.0	19,560.2	2.42 × 10^5^	1.6
R91	8.51 × 10^4^	2.3	23,225.7	1.82 × 10^5^	1.2
R92	4.10 × 10^4^	2.3	23,605.3	5.78 × 10^4^	0.4
R93	9.30 × 10^5^	2.0	22,862.9	3.14 × 10^5^	2.1
**(12) 1,5-H(s)-OOH_cycle_-CY**	**1.10 × 10^3^**	**2.8**	**21,461.1**	**1.52 × 10^5^**	**8.3**
R94	7.83 × 10^5^	1.9	21,958.9	2.18 × 10^5^	0.4
R95	6.53 × 10^6^	1.7	21,710.2	7.74 × 10^5^	1.6
**(13) 1,5-H(t)-CY**	**3.12 × 10^6^**	**1.8**	**21,745.3**	**4.96 × 10^5^**	**3.5**
R96	1.65 × 10^4^	2.1	22,397.1	1.77 × 10^4^	0.4
R97	5.74 × 10^4^	2.0	21,991.9	3.32 × 10^4^	0.8
R98	3.08 × 10^4^	2.1	22,174.6	4.45 × 10^4^	1.1
R99	1.19 × 10^5^	2.2	25,902.4	2.03 × 10^4^	0.5
R100	3.68 × 10^4^	2.3	25,336.2	2.79 × 10^4^	0.7
R101	7.63 × 10^4^	2.3	25,430.9	3.11 × 10^4^	0.8
R102	3.31 × 10^4^	2.2	22,040.7	8.11 × 10^4^	2.0
R103	2.54 × 10^4^	2.2	22,400.5	6.18 × 10^4^	1.6
**(14) 1,6-H(s)-OOH_side_-CY**	**5.53 × 10^1^**	**3.0**	**21,661.3**	**3.97 × 10^4^**	**4.6**
R104	1.35 × 10^6^	2.1	28,924.9	2.45 × 10^4^	0.5
R105	1.96 × 10^5^	2.4	27,260.5	4.87 × 10^4^	1.1
R106	1.08 × 10^5^	2.5	28,135	2.97 × 10^4^	0.7
R107	4.12 × 10^5^	2.3	27,257.6	9.28 × 10^4^	2.1
R108	3.44 × 10^5^	2.3	27,888.2	4.67 × 10^4^	1.0
R109	4.66 × 10^4^	2.5	26,899.1	3.13 × 10^4^	0.7
R110	4.94 × 10^3^	2.8	26,457.4	4.05 × 10^4^	0.9
**(15) 1,6-H(s)-OOH_cycle_-CY**	**7.71 × 10^4^**	**2.5**	**27,290.9**	**4.49 × 10^4^**	**3.8**

^#^*f* = *k*_max_/*k*_min_ in each subclass. ^a^ *k_ave_* is the average rate constant at 800 K in each reaction subclass.

## Data Availability

The original contributions presented in this study are included in the article/Supplementary Material. Further inquiries can be directed to the corresponding author.

## Supplementary Materials

The following supporting information can be downloaded at: https://www.mdpi.com/article/10.3390/molecules30132805/s1, A list of reactions, potential energy profiles for internal rotations, calculated values of the barrier width L for all studied reactions, schemes for the reactions from ROO → QOOH → OOQOOH → products, and the update of the low-temperature combustion mechanism for ethyl cyclohexane are given in the Supplemental Material—File S1. The Cartesian coordinates for all species calculated in this work are provided in the Supplemental Material—File S2. The thermodynamic data including the enthalpies, entropies, and heat capacities are fitted to the NASA format with 14 parameters, which are given in the Supplemental Material—File S3.
