# Supplementary material for: Computational Kinetic Study on the Intramolecular H-Migration of Hydroperoxyalkylperoxy Radicals (•OOQOOH) in Normal-Alkyl Cyclohexanes
Source: Molecules. 2025 Jun 29;30(13):2805. doi: 10.3390/molecules30132805 (PMC12250757; doi:10.3390/molecules30132805)
Supplement: Supplementary file 1 [file molecules-30-02805-s001.zip › Supplemental Material-File S1.pdf]

Supplemental Material-File S1

Computational Kinetic Study on the Intramolecular H-Migration of Hydroperoxyalkylperoxy Radicals (•OOQOOH) in Normal-Alkyl Cyclohexanes

Xiaoxia Yao [1], Juanqin Li [2] and Zerong Li [1,3,*]

[1] College of Chemistry, Sichuan University, Chengdu 610064, China; yaoxxkgd@163.com
[2] College of Chemical Engineering, Sichuan University, Chengdu 610065, China; lijuanqin@scu.edu.cn
[3] Engineering Research Center of Combustion and Cooling for Aerospace Power, Ministry of Education, Sichuan University, Chengdu 610065, China
* Correspondence: lizerong@scu.edu.cn

Contents:

1. A list of reactions

Table S1. A list of reactions of the H-migration of •OOQOOH class in this work.

2. Potential energy profiles for internal rotations

Figure S1(a). Potential energy profiles for internal rotations of the reactant in R60 at B3LYP/CBSB7 level.

Figure S1(b). Potential energy profiles for internal rotations of the transition state in R60 at B3LYP/CBSB7 level.

Figure S1(c). Potential energy profiles for internal rotations of the product in R60 at B3LYP/CBSB7 level.

3. The calculated values of the barrier width *L* for all studied reactions

Table S2. Calculated barrier width *L* (Å) for the class of the H-migration reactions of •OOQOOH.

4. Schemes for the reactions from ROO → QOOH → OOQOOH → products

Scheme S1. Scheme for the reactions of methyl cyclohexane from ROO → QOOH → OOQOOH → products.

Scheme S2. Scheme for the reactions of ethyl cyclohexane from ROO → QOOH → OOQOOH → products.

Scheme S3. Scheme for the reactions of n-propyl cyclohexane from ROO → QOOH → OOQOOH → products.

Scheme S4. Scheme for the reactions of n-butyl cyclohexane from ROO → QOOH → OOQOOH → products.

5. Update of the low-temperature combustion mechanism for ethyl cyclohexane

Table S3. A list of the high-pressure limit rate constants of the H-migration of •OOQOOH reactions in the updated mechanism and in the original mechanism constructed by Zou et al.

6. Update of the low-temperature combustion mechanism for n-propyl cyclohexane

Table S4. A list of the high-pressure limit rate constants of the H-migration of •OOQOOH reactions in the updated mechanism and in the original mechanism constructed by Liu et al.

1. A list of reactions

Table S1. A list of reactions of H-migration reactions of •OOQOOH class in this study.

No.	Reactions
**R1**	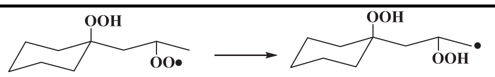
**R2**	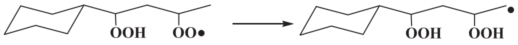
**R3**	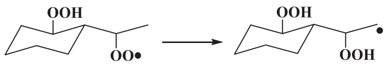
**R4**	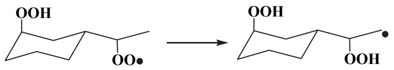
**R5**	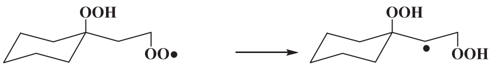
**R6**	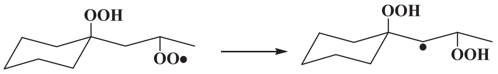
**R7**	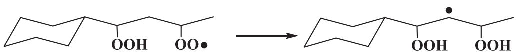
**R8**	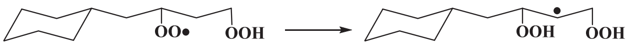
**R9**	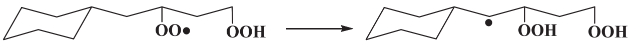
**R10**	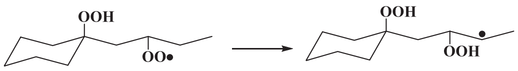
**R11**	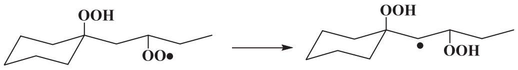
**R12**	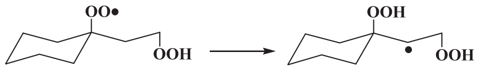
**R13**	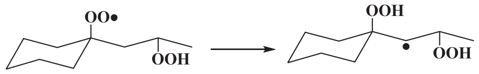
**R14**	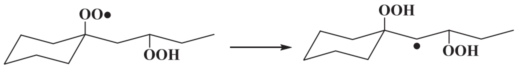
**R15**	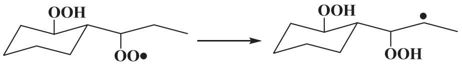
**R16**	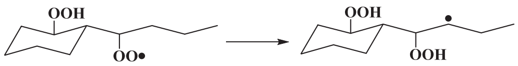
**R17**	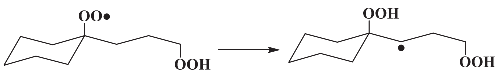
**R18**	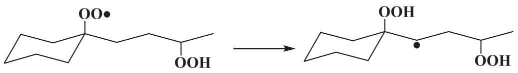
**R19**	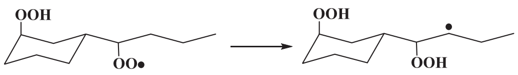
**R20**	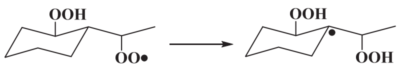
**R21**	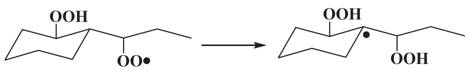

R22	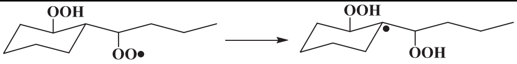
R23	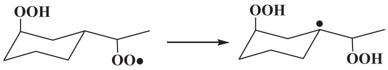
R24	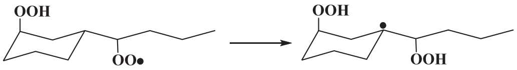
R25	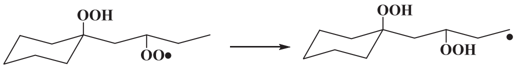
R26	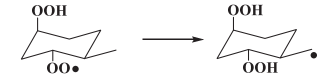
R27	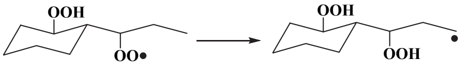
R28	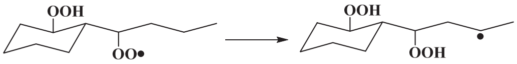
R29	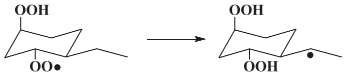
R30	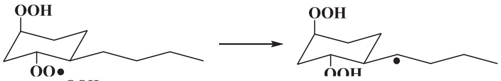
R31	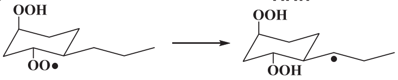
R32	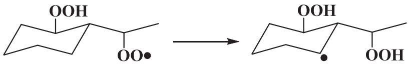
R33	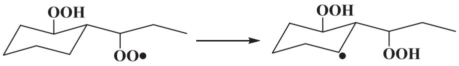
R34	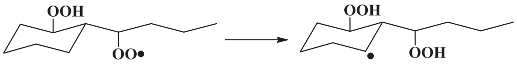
R35	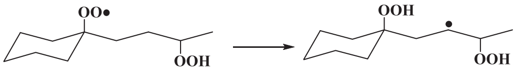
R36	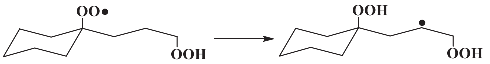
R37	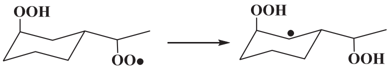
R38	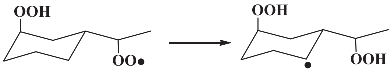
R39	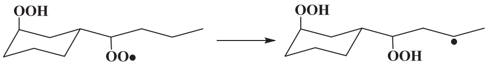
R40	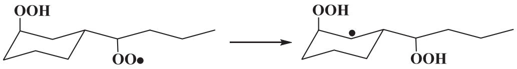
R41	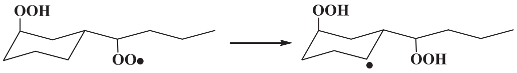
R42	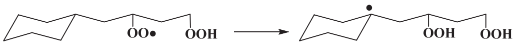
R43	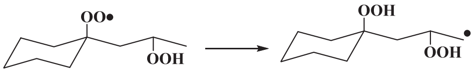
R44	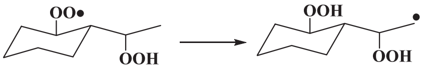
R45	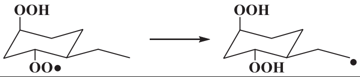

**R46**	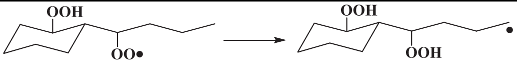
**R47**	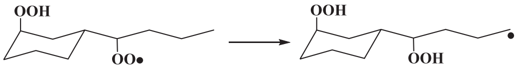
**R48**	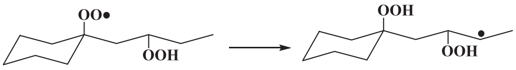
**R49**	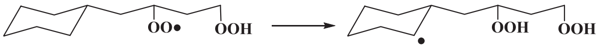
**R50**	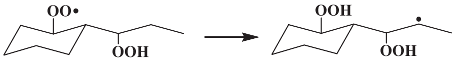
**R51**	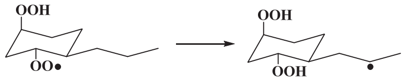
**R52**	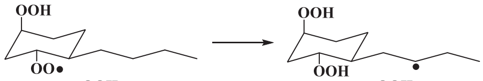
**R53**	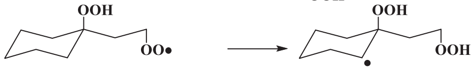
**R54**	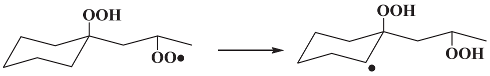
**R55**	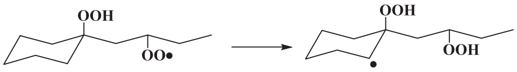
**R56**	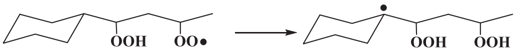
**R57**	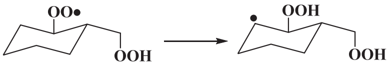
**R58**	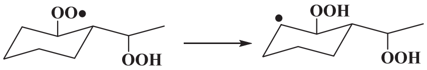
**R59**	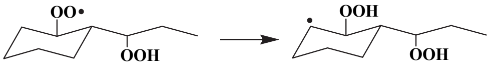
**R60**	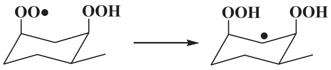
**R61**	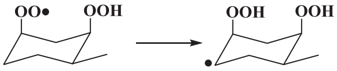
**R62**	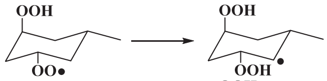
**R63**	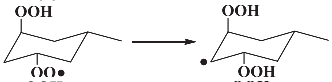
**R64**	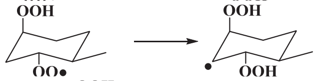
**R65**	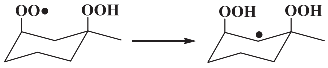
**R66**	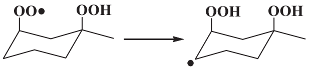
**R67**	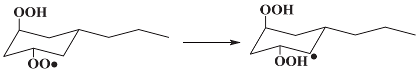
**R68**	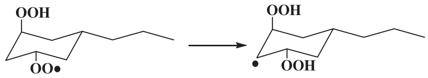
**R69**	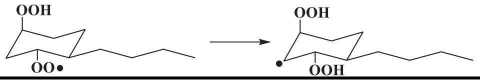

R70 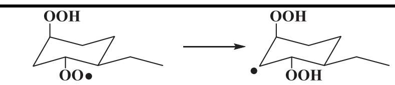

R71 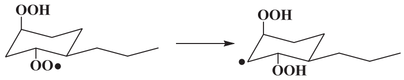

R72 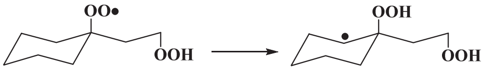

R73 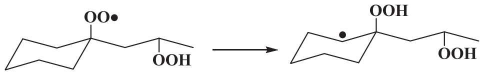

R74 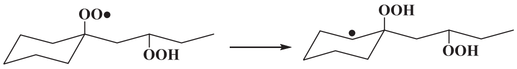

R75 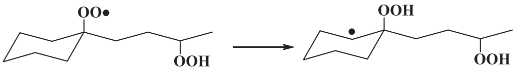

R76 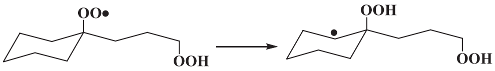

R77 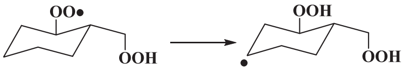

R78 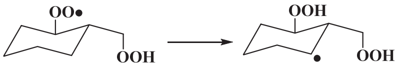

R79 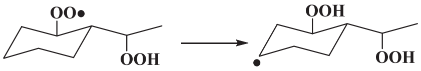

R80 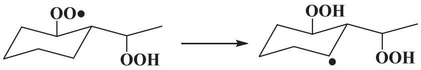

R81 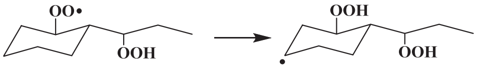

R82 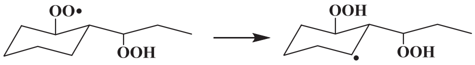

R83 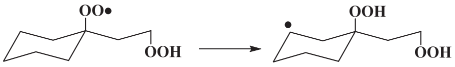

R84 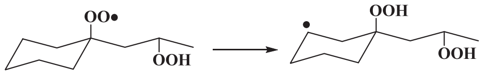

R85 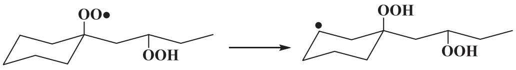

R86 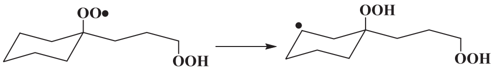

R87 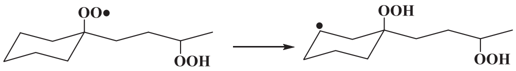

R88 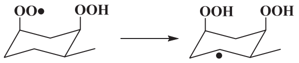

R89 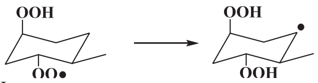

R90 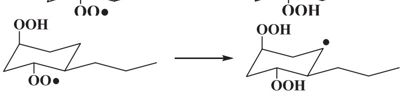

R91 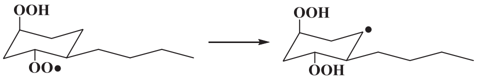

R92 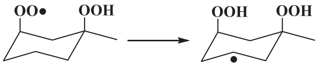

R93 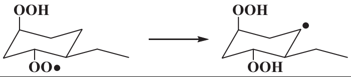

**R94**	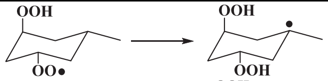
**R95**	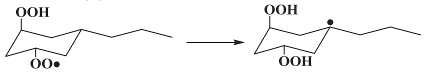
**R96**	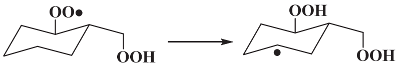
**R97**	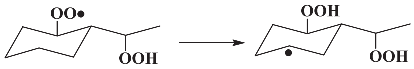
**R98**	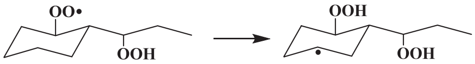
**R99**	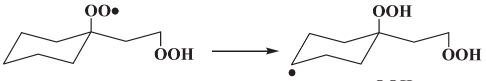
**R100**	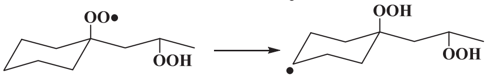
**R101**	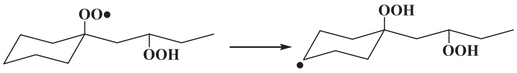
**R102**	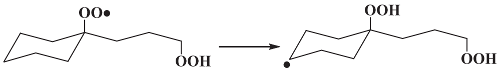
**R103**	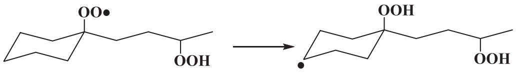
**R104**	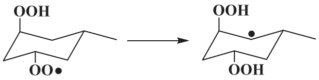
**R105**	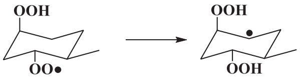
**R106**	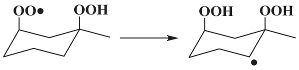
**R107**	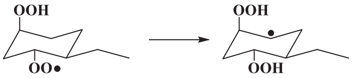
**R108**	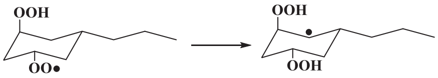
**R109**	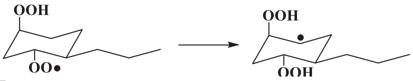
**R110**	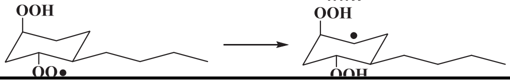

2. Potential energy profiles for internal rotations

In our study, one-dimensional (1-D) hindered internal rotors are used to treat low-frequency vibrations corresponding to torsions about a single bond for reactants, transition states and products. When dealing with the hindrance potentials of transition states, 1-D hindered rotor scans are implemented by freezing the atoms involved in the reaction centers. Since cyclohexane has a relatively stable cycle structure, the anharmonic correction corresponding to the internal rotations of the single bond on the cycle of the reactants, transition states, and products in this work are all not considered. Here, we only choose the reaction of R60 in the H-migration reactions of OOQOOH class as an

example, and the potential energy profiles for internal rotations about C-C, C-OO and CO-OH single bonds of the reactant, the transition state, and the product in R60 are shown in Fig. S1(a), Fig. S1(b), and Fig. S1(c).

For the reactant, it can be seen from Fig. S1(a) that the calculated rotational profile of C6H9(OO)(OOH)---CH3 group in reactant shows a symmetry three-fold barrier of 2.7 kcal mol^{-1}. The rotation of the C6H9(OO)---(OOH)CH3 group in reactant shows an asymmetrical three-fold potential barrier of 11.2 kcal mol^{-1}. The rotations of the C6H9---(OO)(OOH)CH3 group and the C6H9(OO)(O---OH)CH3 group in reactant show asymmetrical two-fold potential barriers of 5.0 and 10.3 kcal mol^{-1}, respectively.

For the transition state, there are only three potential energy profiles for internal rotations about single bonds because the anharmonic correction corresponding to the single bonds involved in the reaction centers are not considered. It can be seen that from Fig. S1(b) that, the calculated rotational profile of C6H9(OO)(OOH)---CH3 group in transition state shows a symmetry three-fold barrier of 2.6 kcal mol^{-1}, and the rotation of C6H9(OO)---(OOH)CH3 group in transition state shows an asymmetrical three-fold potential barriers of 11.4 kcal mol^{-1}. The rotation of the C6H9(OO)O---OHCH3 group in transition state shows an asymmetrical two-fold potential barrier of 11.0 kcal mol^{-1}.

From Fig. S1(c), it can be seen that, the calculated rotational profile of C6H8OOH(OOH)---CH3 group in product shows a three-fold symmetry with a barrier of 2.7 kcal mol^{-1}. The rotations of the C6H8---(OOH)(OOH)CH3 group and the C6H8OOH---(OOH)CH3 group in product show asymmetrical three-fold potential barriers of 9.2 and 10.7 kcal mol^{-1}. The rotations of the C6H8(O---OH)(OOH)CH3 group and the C6H8(OOH)(O---OH)CH3 group in product show asymmetrical two-fold potential barriers of 6.6 and 9.6 kcal mol^{-1}.

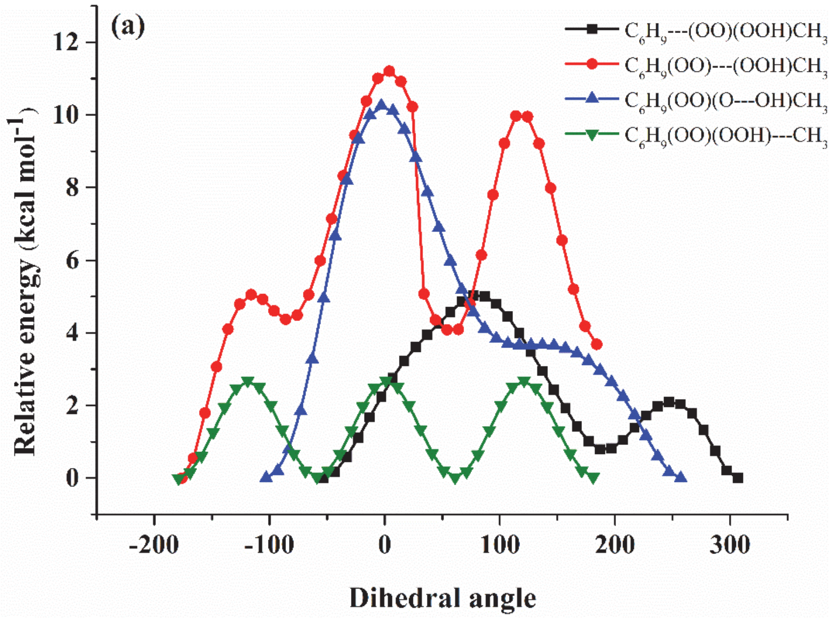

Figure S1(a). Potential energy profiles for internal rotations of the reactant in R60 at B3LYP/CBSB7 level.

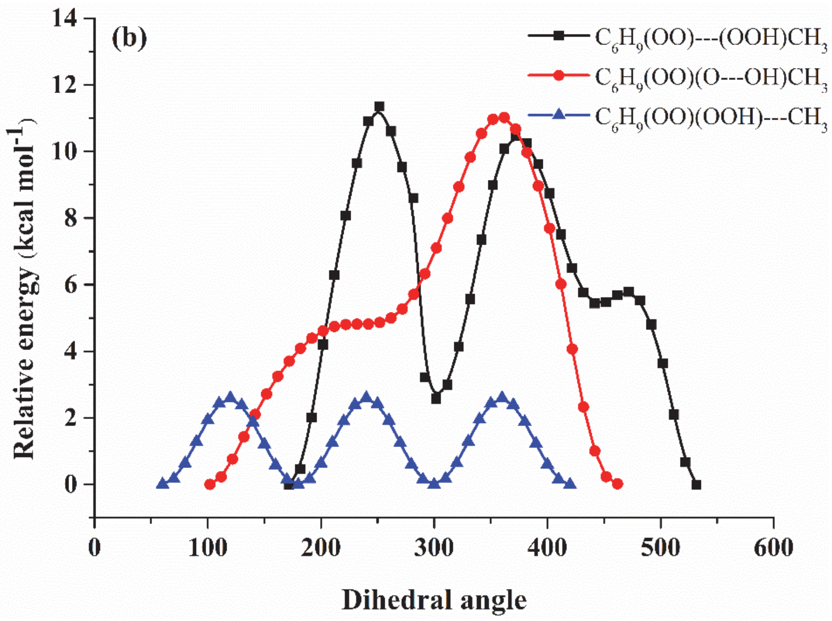

Figure S1(b). Potential energy profiles for internal rotations of the transition state in R60 at B3LYP/CBSB7 level.

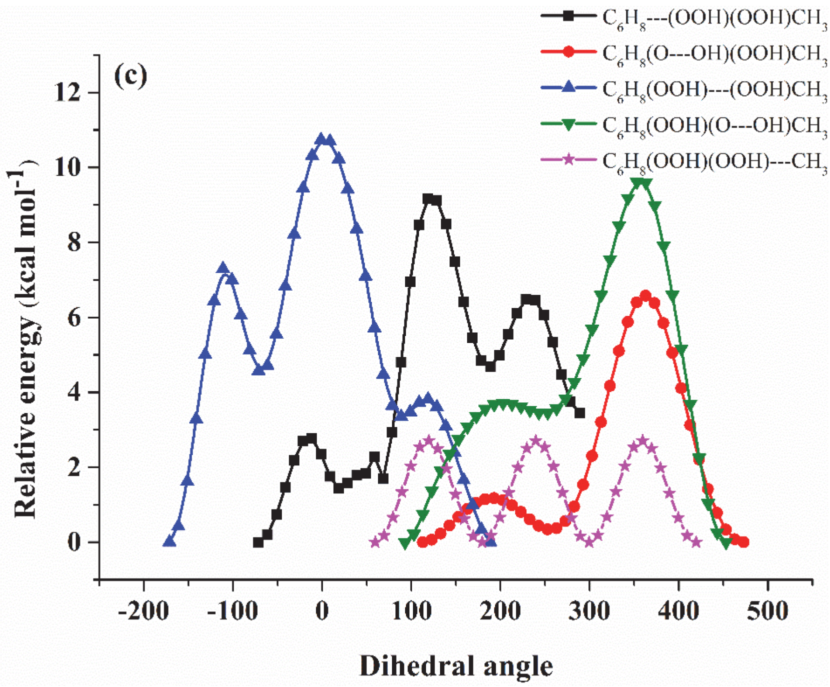

Figure S1(c). Potential energy profiles for internal rotations of the product in R60 at B3LYP/CBSB7 level.

3. The calculated values of the barrier width *L* for all studied reactions

Table S2. Calculated barrier width *L* (Å) for the class of the H-migration reactions of •OOQOOH.

Reaction	Barrier width *L*	Reaction	Barrier width *L*	Reaction	Barrier width *L*
R1	1.05	R38	0.92	R75	1.05
R2	1.04	R39	0.92	R76	1.02
R3	0.96	R40	0.95	R77	1.06
R4	0.99	R41	0.84	R78	1.02
R5	1.02	R42	0.79	R79	1.04
R6	1.03	R43	0.99	R80	1.01
R7	1.02	R44	0.93	R81	1.02
R8	1.02	R45	0.89	R82	0.98
R9	1.05	R46	0.71	R83	1.10
R10	1.04	R47	0.87	R84	1.07
R11	1.04	R48	0.87	R85	1.12
R12	1.02	R49	0.82	R86	1.05
R13	1.04	R50	0.92	R87	1.11
R14	1.04	R51	0.80	R88	1.12
R15	0.96	R52	0.82	R89	1.09
R16	0.98	R53	0.92	R90	0.82
R17	1.00	R54	0.94	R91	1.10
R18	1.04	R55	0.85	R92	1.11
R19	1.07	R56	0.91	R93	1.08
R20	1.01	R57	1.06	R94	1.04
R21	1.00	R58	1.05	R95	1.03

R22	1.02	R59	1.02	R96	1.01
R23	1.05	R60	1.00	R97	1.06
R24	1.01	R61	1.04	R98	1.08
R25	0.95	R62	1.04	R99	1.04
R26	0.88	R63	1.00	R100	1.04
R27	0.77	R64	1.01	R101	1.04
R28	0.79	R65	1.01	R102	1.04
R29	0.85	R66	1.04	R103	1.04
R30	0.84	R67	1.02	R104	0.99
R31	0.79	R68	0.99	R105	1.18
R32	0.96	R69	0.99	R106	1.19
R33	0.96	R70	1.00	R107	1.16
R34	0.98	R71	1.00	R108	1.07
R35	1.01	R72	1.09	R109	1.18
R36	1.01	R73	1.09	R110	1.17
R37	0.96	R74	1.09		

4. Schemes for the reactions from ROO → QOOH → OOQOOH → products

In this work, QOOH formed from the H-migration of ROO• with the lowest energy barriers are chosen to be the dominant intermediate undergoing further O_2 addition from our previous work [1]. The schemes for the reactions of methyl cyclohexane, ethyl cyclohexane, propyl cyclohexane and butyl cyclohexane from ROO → QOOH → OOQOOH → products are shown in Scheme S1-S4. The reactions listed in the left side of the schemes are referred to our previous work, in which the unit of the reaction barrier is kcal mol^{-1}, and the reactions listed in the right side of the scheme are studied in this work.

[1] X.X. Yao, J.B. Wang, Q. Yao, Y.Q. Li, Z.R. Li, X.Y. Li, Pressure-dependent rate rules for intramolecular H-migration reactions of normal-alkyl cyclohexylperoxy radicals, Combust. Flame 204 (2019) 176-188.

Scheme S1. Scheme for the reactions of methyl cyclohexane from ROO → QOOH → OOQOOH → products, units: kcal·mol^{-1}. (The reactions in red are the targets of this work)

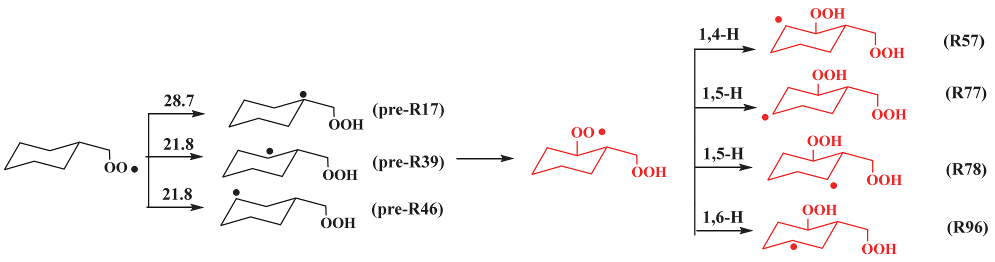

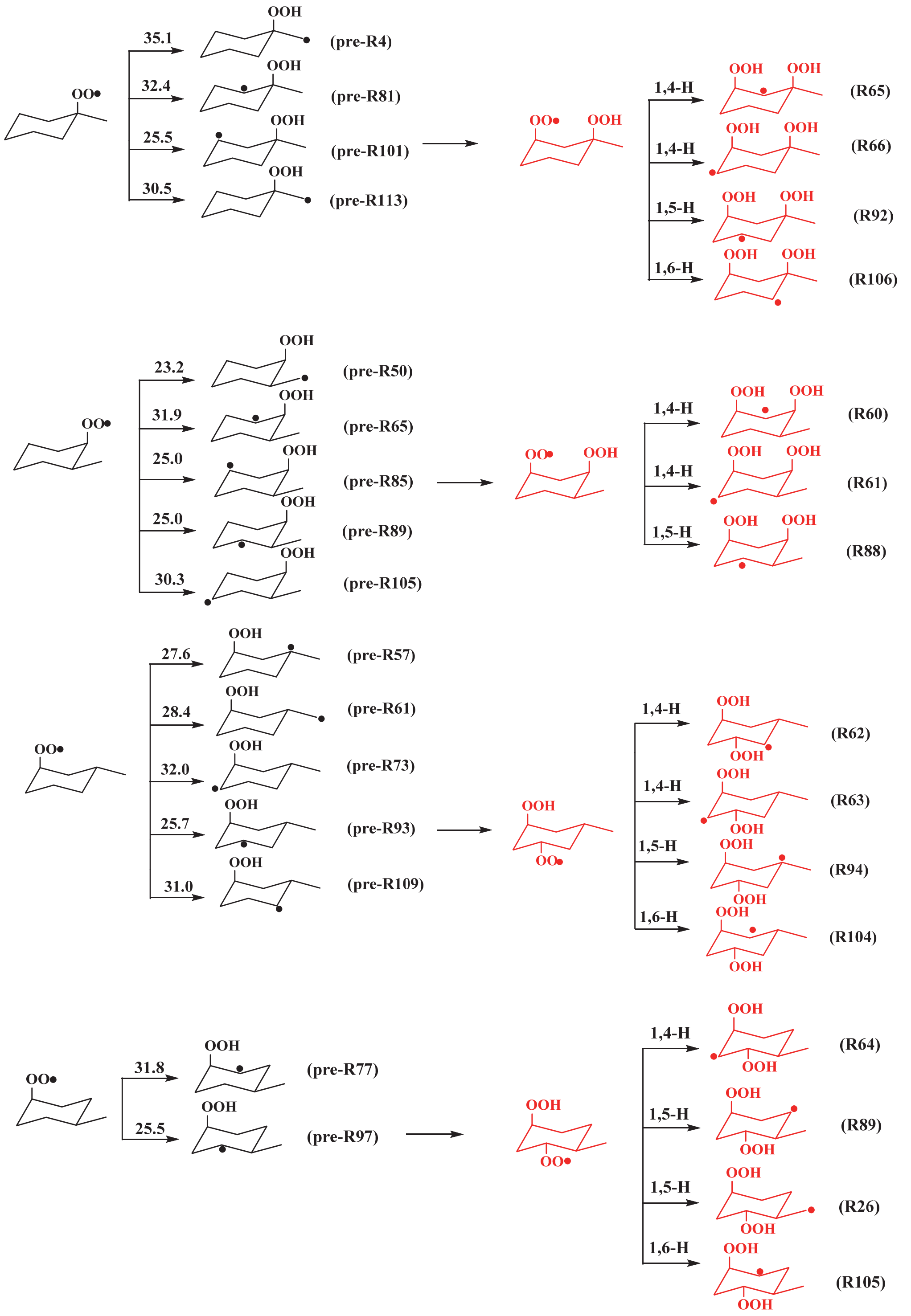

Scheme S2. Scheme for the reactions of ethyl cyclohexane from ROO → QOOH → OOQOOH → products, units: kcal·mol^{-1}. (The reactions in red are the targets of this work)

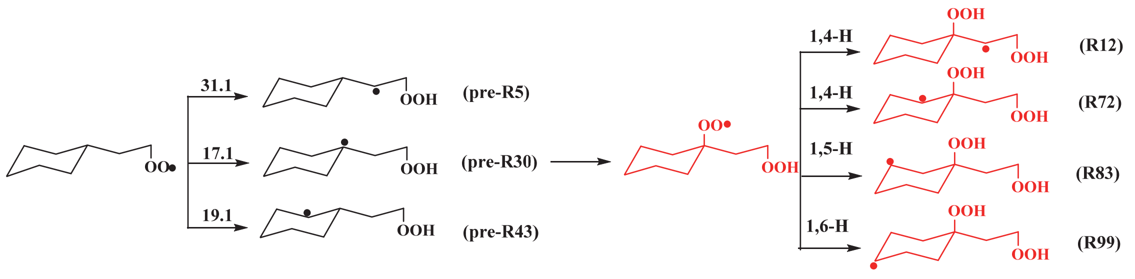
31.1
(pre-R5)
17.1
(pre-R30)
19.1
(pre-R43)
1,4-H
(R12)
1,4-H
(R72)
1,5-H
(R83)
1,6-H
(R99)

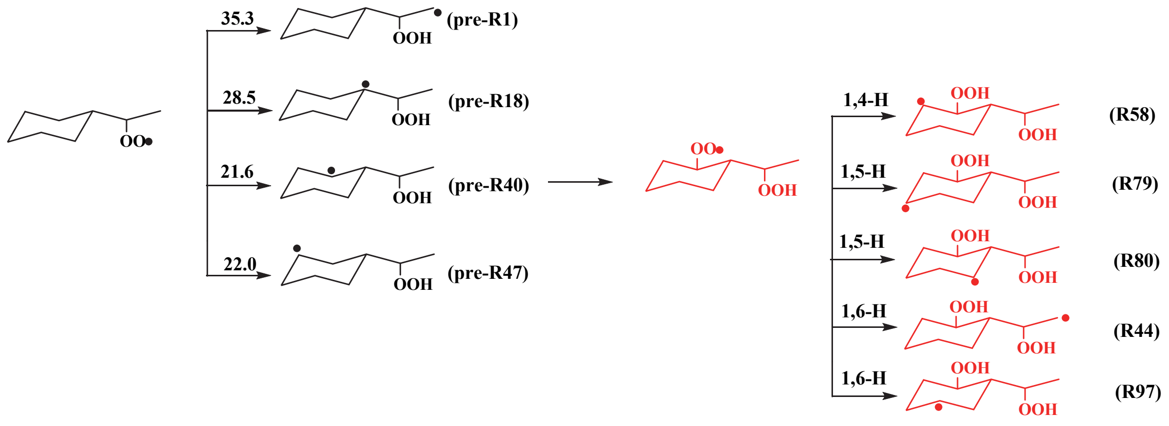
35.3
(pre-R1)
28.5
(pre-R18)
21.6
(pre-R40)
22.0
(pre-R47)
1,4-H
(R58)
1,5-H
(R79)
1,5-H
(R80)
1,6-H
(R44)
1,6-H
(R97)

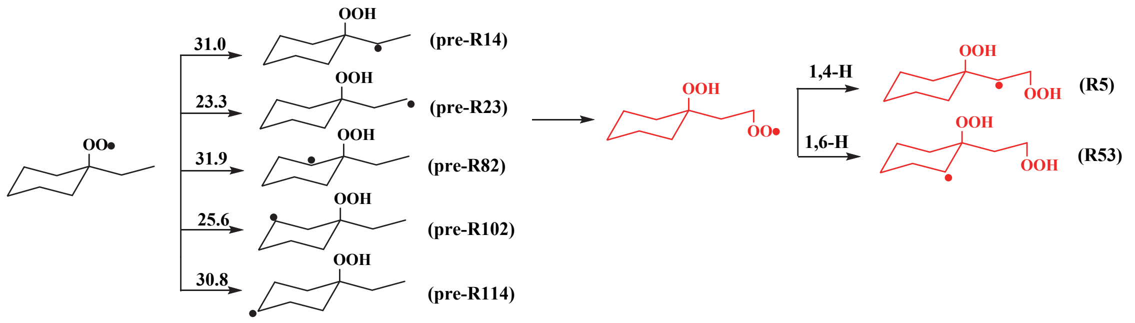
31.0
(pre-R14)
23.3
(pre-R23)
31.9
(pre-R82)
25.6
(pre-R102)
30.8
(pre-R114)
1,4-H
(R5)
1,6-H
(R53)

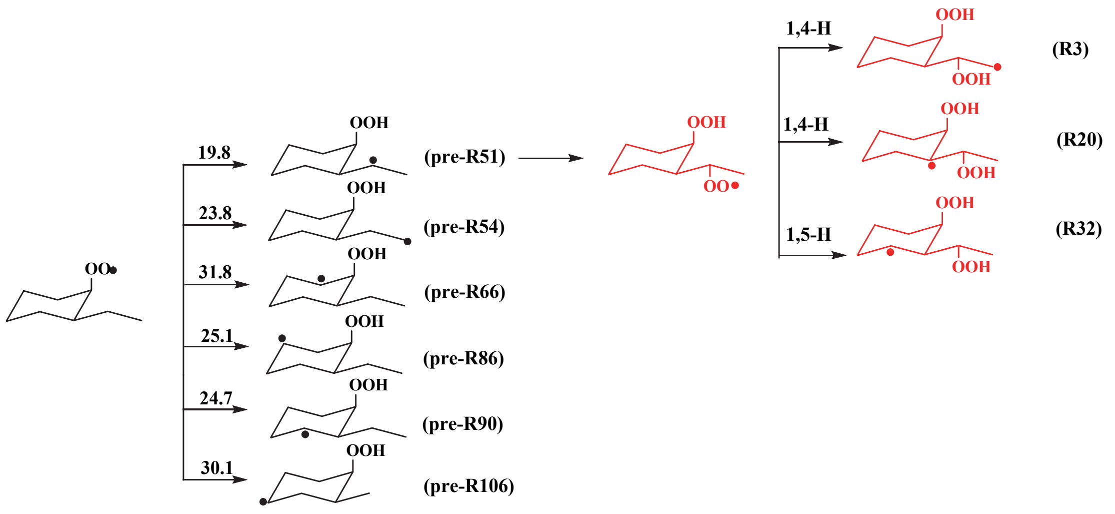
19.8
(pre-R51)
23.8
(pre-R54)
31.8
(pre-R66)
25.1
(pre-R86)
24.7
(pre-R90)
30.1
(pre-R106)
1,4-H
(R3)
1,4-H
(R20)
1,5-H
(R32)

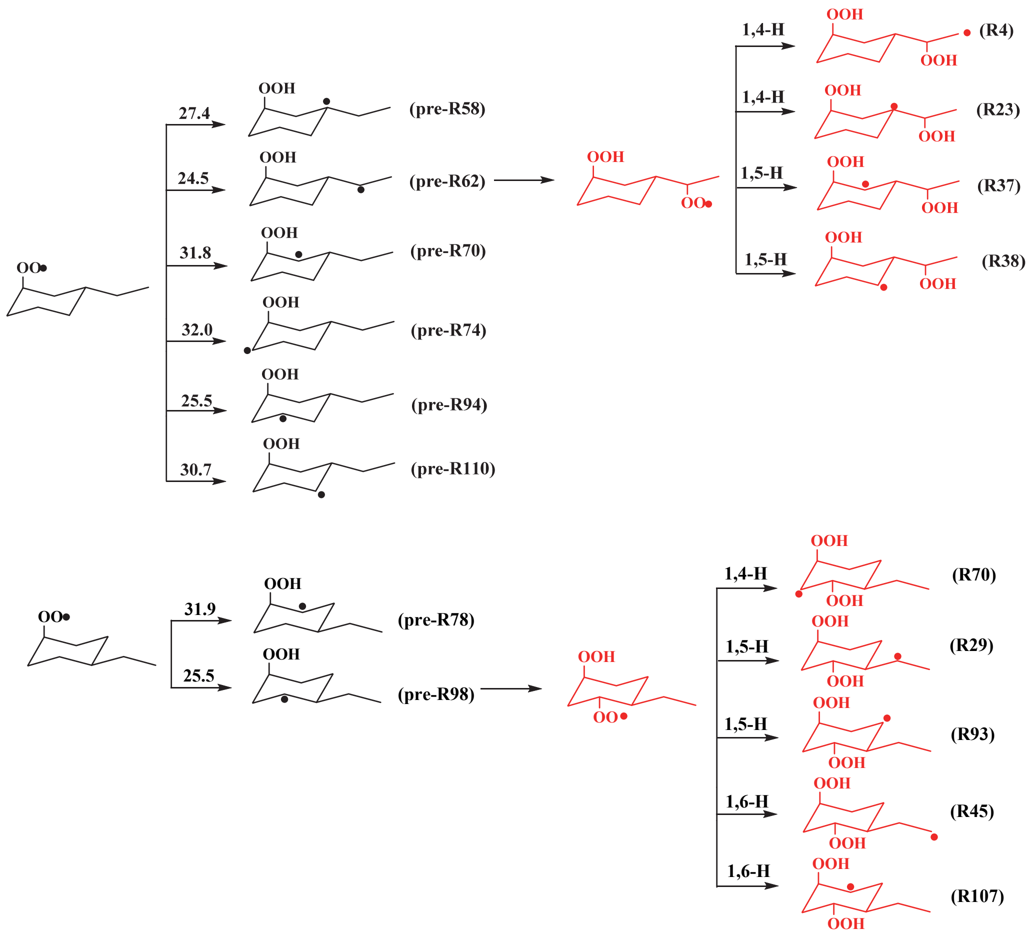

Scheme S3. Scheme for the reactions of propyl cyclohexane from ROO → QOOH → OOQOOH → products, units: kcal·mol^{-1}. (The reactions in red are the targets of this work)

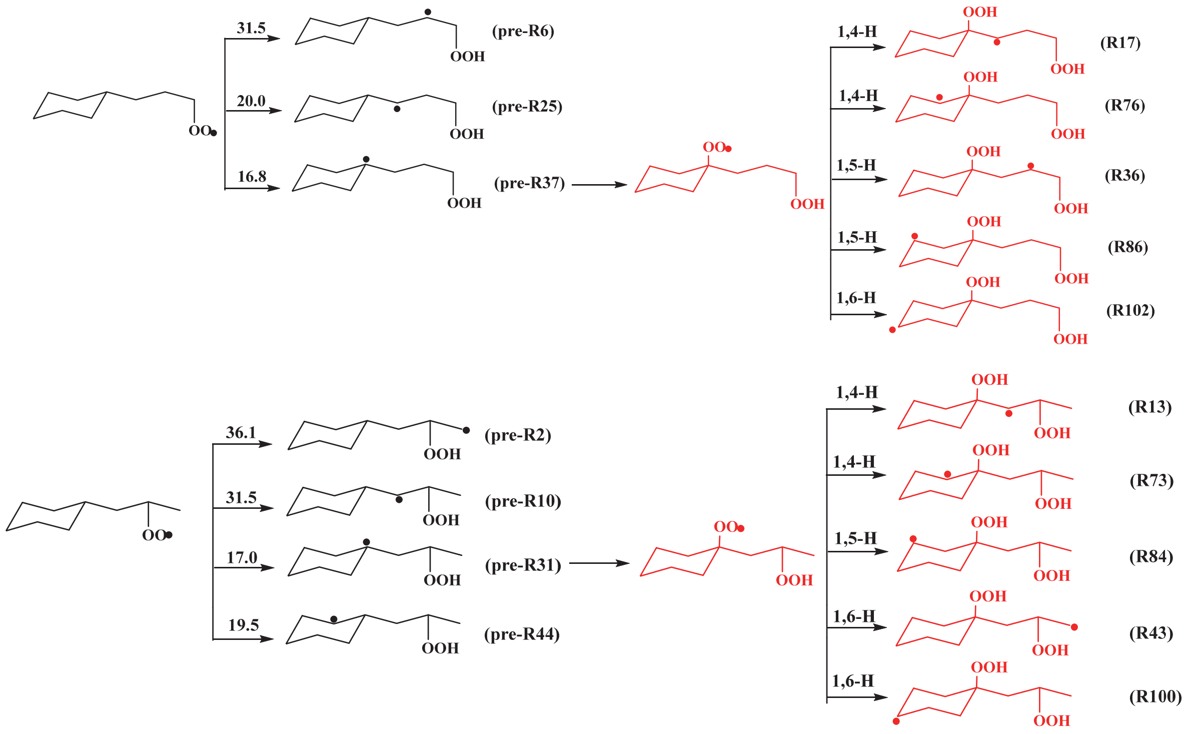

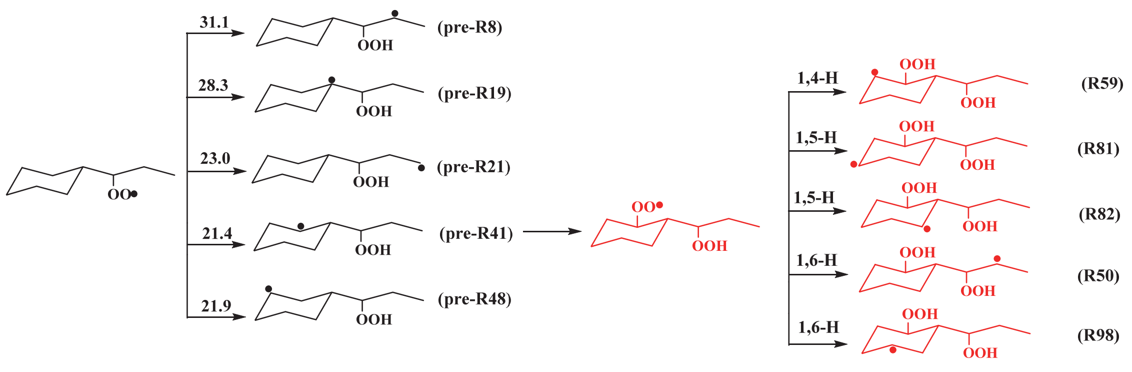

OO•
31.1
OOH
(pre-R8)
28.3
(pre-R19)
23.0
(pre-R21)
21.4
(pre-R41)
21.9
(pre-R48)
1,4-H
(R59)
1,5-H
(R81)
1,5-H
(R82)
1,6-H
(R50)
1,6-H
(R98)

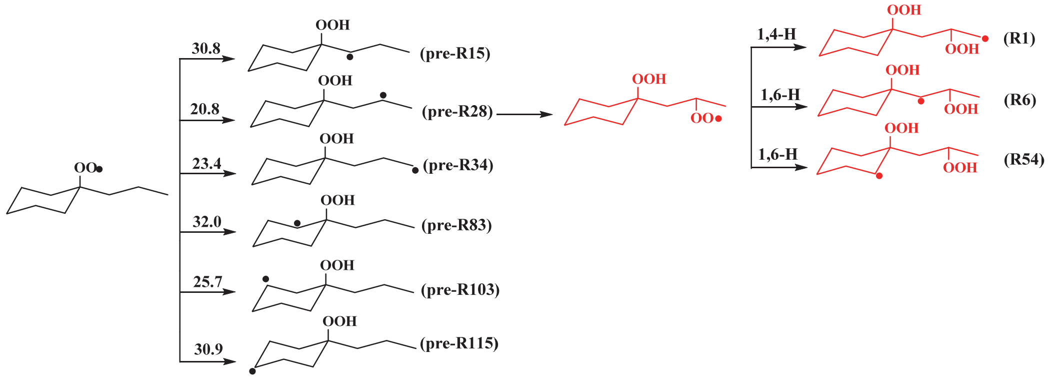

OO•
30.8
OOH
(pre-R15)
20.8
(pre-R28)
23.4
(pre-R34)
32.0
(pre-R83)
25.7
(pre-R103)
30.9
(pre-R115)
1,4-H
(R1)
1,6-H
(R6)
1,6-H
(R54)

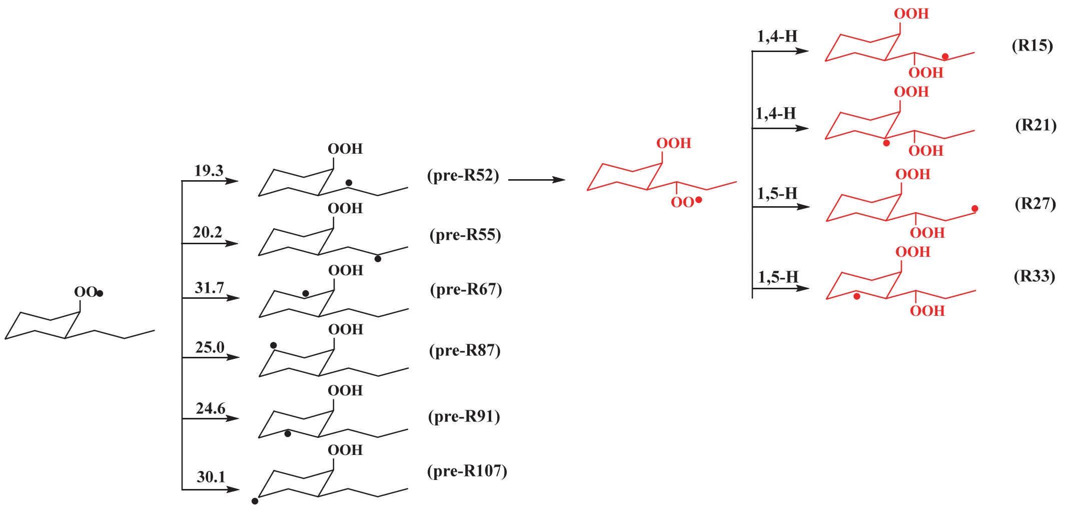

OO•
19.3
OOH
(pre-R52)
20.2
(pre-R55)
31.7
(pre-R67)
25.0
(pre-R87)
24.6
(pre-R91)
30.1
(pre-R107)
1,4-H
(R15)
1,4-H
(R21)
1,5-H
(R27)
1,5-H
(R33)

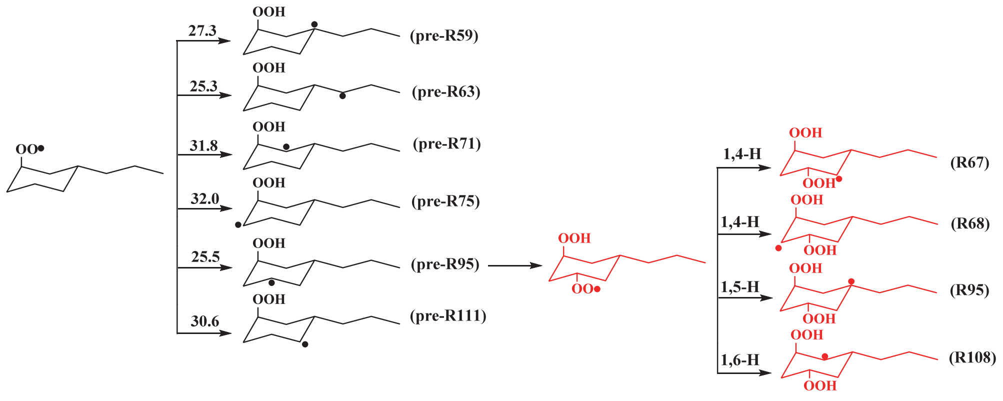

OO•
27.3
OOH
(pre-R59)
25.3
(pre-R63)
31.8
(pre-R71)
32.0
(pre-R75)
25.5
(pre-R95)
30.6
(pre-R111)
1,4-H
(R67)
1,4-H
(R68)
1,5-H
(R95)
1,6-H
(R108)

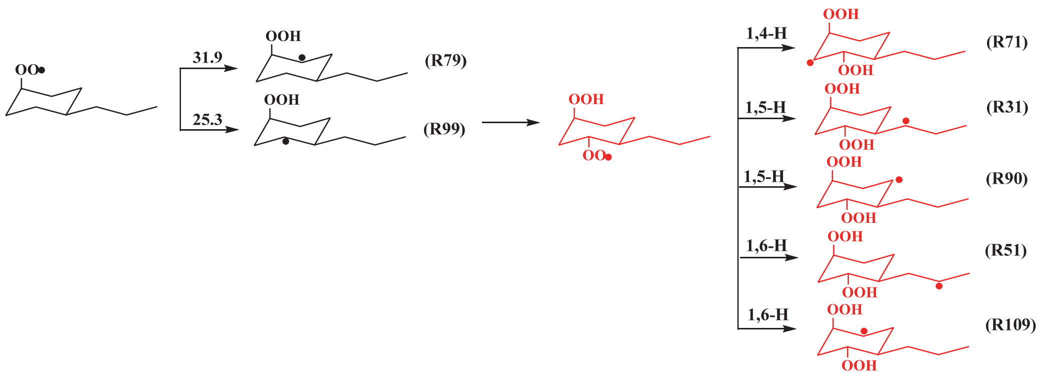

Scheme S4. Scheme for the reactions of butyl cyclohexane from ROO → QOOH → OOQOOH → products, units: kcal·mol^{-1}. (The reactions in red are the targets of this work)

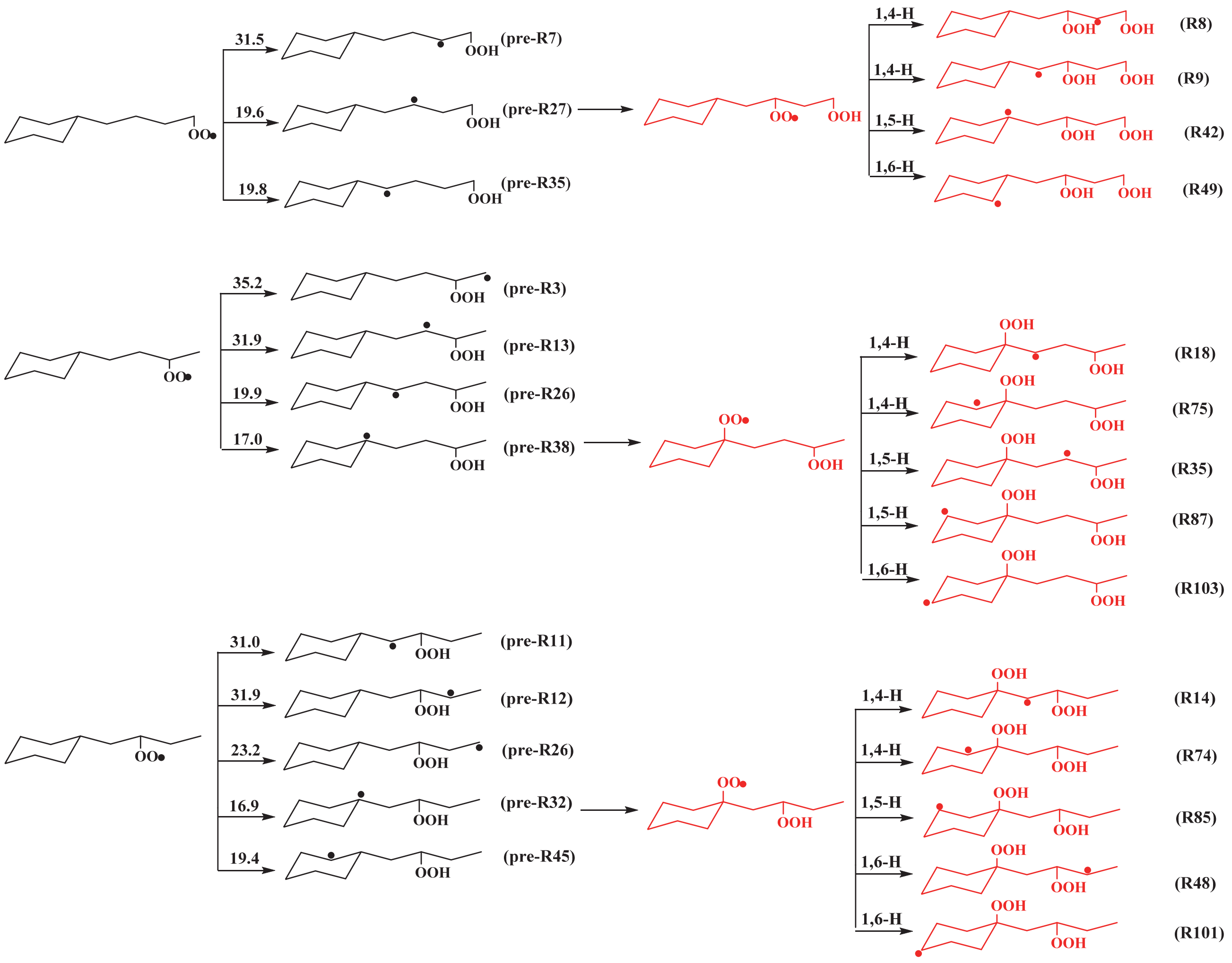

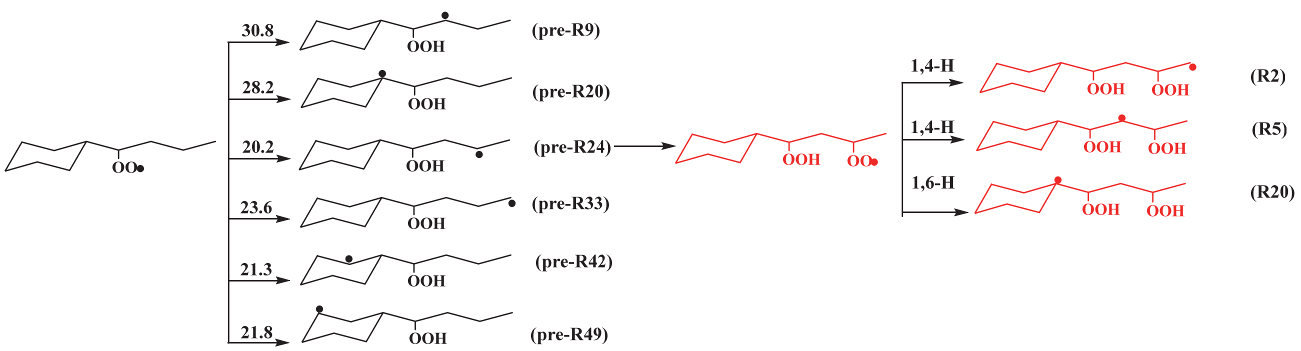
OO•
30.8
28.2
20.2
23.6
21.3
21.8
OOH
(pre-R9)
(pre-R20)
(pre-R24)
(pre-R33)
(pre-R42)
(pre-R49)
1,4-H
1,4-H
1,6-H
(R2)
(R5)
(R20)

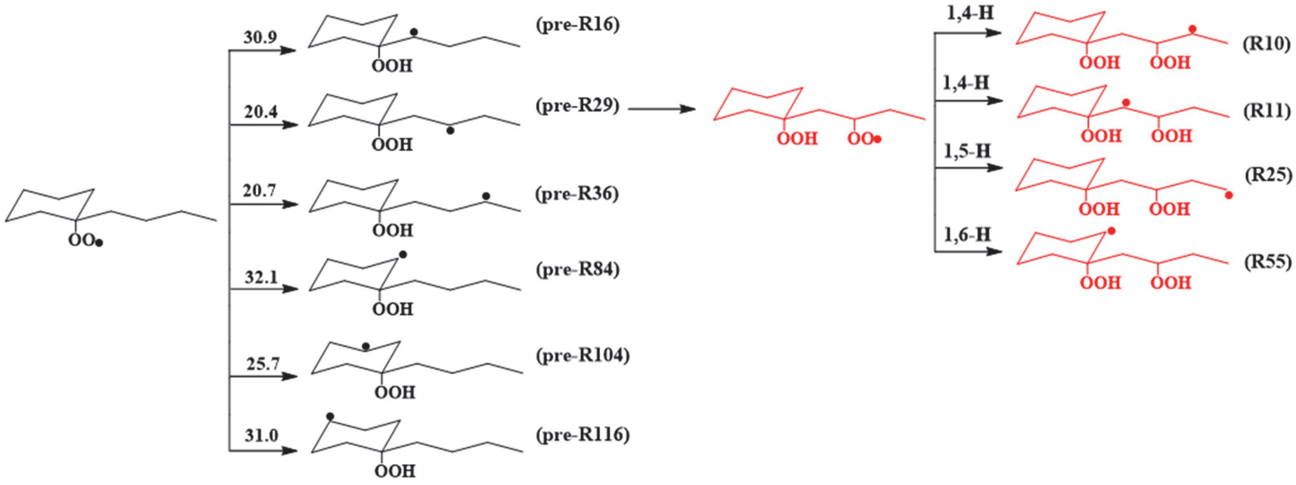
OO•
30.9
20.4
20.7
32.1
25.7
31.0
OOH
(pre-R16)
(pre-R29)
(pre-R36)
(pre-R84)
(pre-R104)
(pre-R116)
1,4-H
1,4-H
1,5-H
1,6-H
(R10)
(R11)
(R25)
(R55)

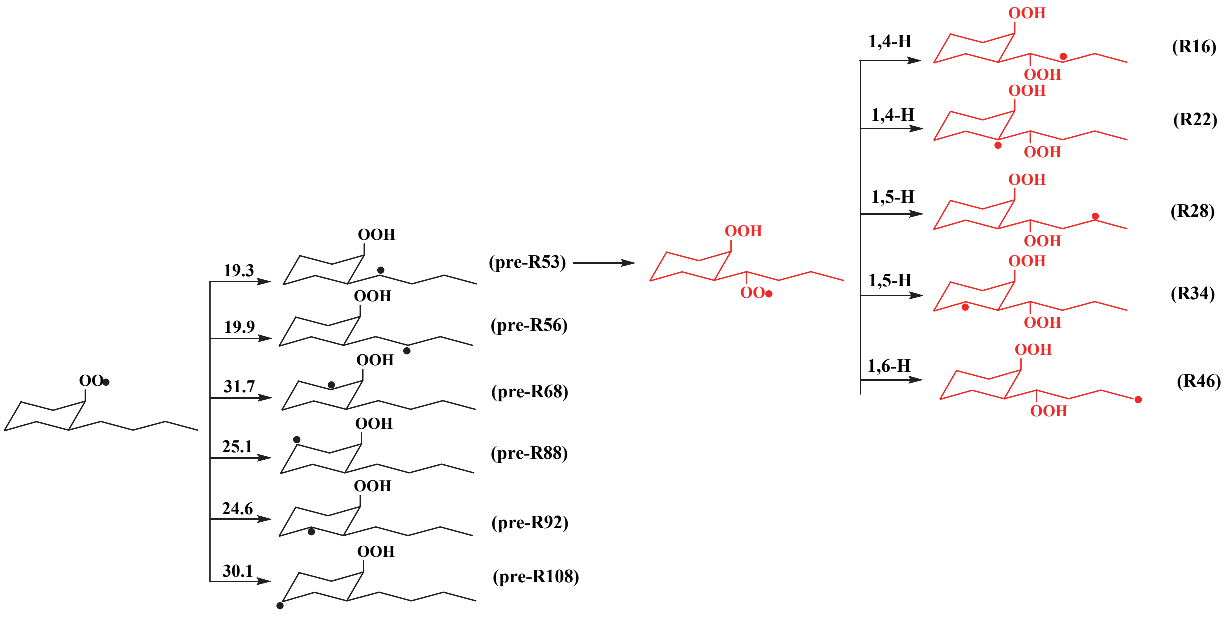
OO•
19.3
19.9
31.7
25.1
24.6
30.1
OOH
(pre-R53)
(pre-R56)
(pre-R68)
(pre-R88)
(pre-R92)
(pre-R108)
1,4-H
1,4-H
1,5-H
1,5-H
1,6-H
(R16)
(R22)
(R28)
(R34)
(R46)

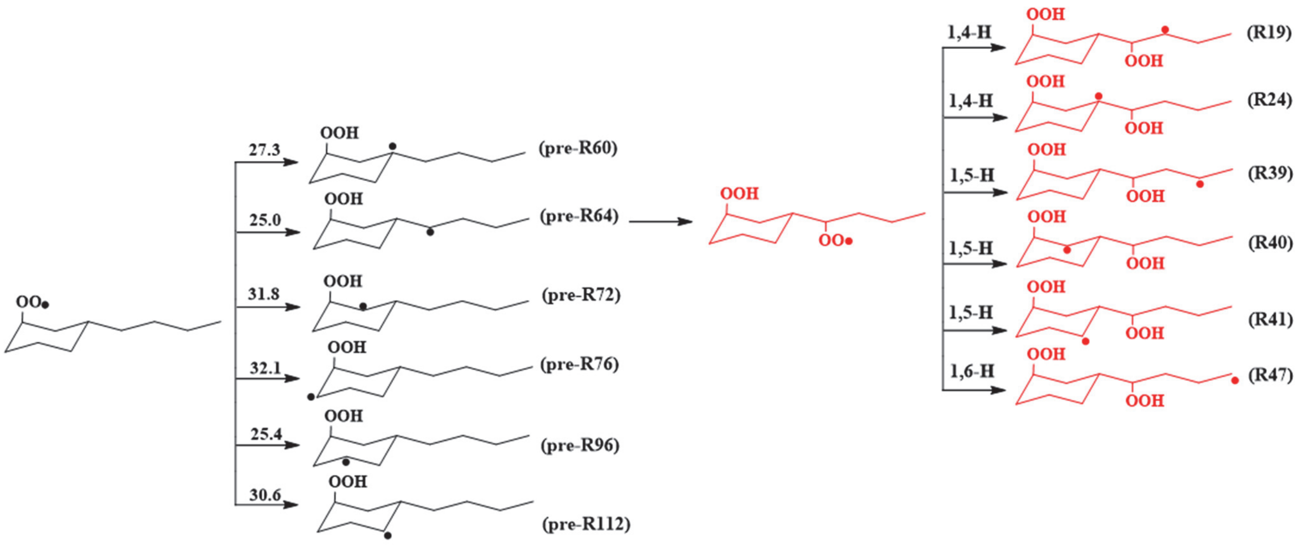

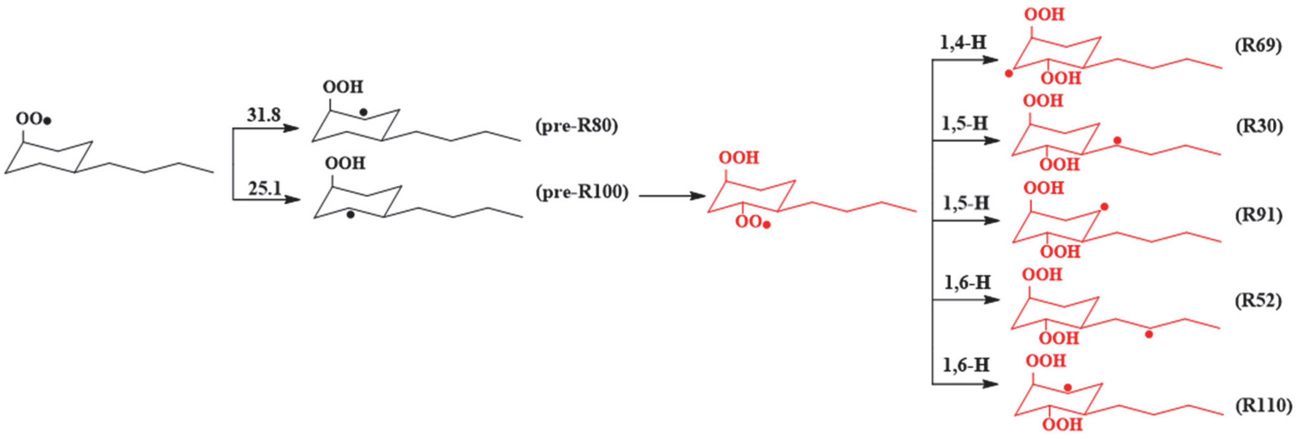

5. Update of the low-temperature combustion mechanism for ethyl cyclohexane

Table S3. A list of the high-pressure limit rate constants of the H-migration of •OOQOOH reactions in the updated mechanism and in the original mechanism constructed by Zou et al. [2].

Reactions	Updated mechanism (From this work)			Original mechanism (From Zou et al. [2])		
	A	*n*	E_a	*A*	*n*	E_a
ECH2Q7QJ = ECH2Q7Q1J<br>The data from P(OOH)2-R3	1.44E+02	3.3	25103.5	6.15E+11	-0.3	26800
ECH7Q2QJ = ECH2Q7Q3J<br>The data from P(OOH)2-R44	3.65E+03	2.3	18974.0	6.15E+11	-0.3	28800
ECH8Q1QJ = ECH1Q8Q7J<br>The data from P(OOH)2-R12	1.51E+02	3.1	24140.7	4.68E-01	3.85	23632.3
ECH8Q1QJ = ECH1Q8Q2J<br>The data from P(OOH)2-R72	1.22E+02	3.0	26018.0	3.47E-01	3.88	24029.6
ECH2Q7QJ = ECH2Q7Q6J<br>The data from P(OOH)2-R32	2.23E+08	1.2	17494.1	7.75E+06	1.68	18000.6
ECH4Q2QJ = ECH2Q4Q6J<br>The data from P(OOH)2-R93	9.30E+05	2.0	22862.9	1.38E+04	2.33	20201.4
ECH4Q2QJ = ECH2Q4Q7J<br>The data from P(OOH)2-R29	1.54E+07	1.4	16675.8	4.90E+07	1.43	16465
ECH7Q2QJ = ECH2Q7Q4J	8.77E+06	1.4	20139.0	2.83E+02	2.7	21637

The data from P(OOH)2-R79						
ECH7Q2QJ = ECH2Q7Q6J<br>The data from P(OOH)2-R80	6.45E+05	1.7	18860.0	1.38E+04	2.33	20201.4
ECH8Q1QJ = ECH1Q8Q3J<br>The data from P(OOH)2-R83	3.43E+05	2.0	21779.8	1.0	0.0	0.0
ECH2Q7QJ = ECH2O7Q + OH<br>The data from KHP-R118	1.96E+08	1.2	13815.8	7.75E+06	1.68	15000.6
ECH7Q2QJ = ECH7O2Q + OH<br>The data from KHP-R116	6.33E+06	1.6	12001.2	2.45E+07	1.43	13465
ECH8Q1QJ = ECH8O1Q + OH<br>The data from KHP-R112	1.40E+07	1.8	16302.7	4.31E+05	2.19	16372.9

[2] J.B. Zou, Y.Y. Li, L.L. Ye, H.F. Jin, A comprehensive study on low-temperature oxidation chemistry of cyclohexane. I. Conformational analysis and theoretical study of first and second oxygen addition. Combust. Flame 235 (2022) 111658.

6. Update of the low-temperature combustion mechanism for n-propyl cyclohexane

Table S4. A list of the high-pressure limit rate constants of the H-migration of •OOQOOH reactions in the updated mechanism and in the original mechanism constructed by Liu et al. [3].

Reactions	Updated mechanism (From this work)			Original mechanism (From Liu et al. [3])		
	A	*n*	E_a	*A*	*n*	E_a
PCH1Q4QJ = PCHQ14-2<br>The data from P(OOH)2-R36	1.45E+06	1.6	16352.5	6.03E+06	1.40	17381.2
PCH1Q4QJ = PCHQ14-6<br>The data from P(OOH)2-R86	1.66E+05	2.0	18681.5	1.51E+04	2.40	21085.4
PCH2Q4QJ = PCHQ24-6<br>The data from P(OOH)2-R84	1.21E+05	2.0	20609.7	1.51E+04	2.40	21085.4
PCH3Q5QJ = PCHQ35-9<br>The data from P(OOH)2-R82	5.35E+05	1.9	18479.5	9.12E+04	2.12	20427.1
PCH3Q5QJ = PCHQ35-7<br>The data from P(OOH)2-R81	2.14E+05	1.9	18981.2	4.47E+04	2.19	20604.9
PCH5Q3QJ = PCHQ35-1<br>The data from P(OOH)2-R27	6.42E+06	1.7	17492.2	3.47E+06	1.81	19446.1
PCH5Q3QJ = PCHQ35-9<br>The data from P(OOH)2-R33	2.70E+06	1.7	17128.1	2.945E+05	1.76	17669.1
PCH6Q8QJ = PCHQ68-4<br>The data from P(OOH)2-R95	6.53E+06	1.7	21710.2	4.36E+03	2.55	21988.8
PCH7Q5QJ = PCHQ57-3<br>The data from P(OOH)2-R31	2.20E+07	1.5	16129.9	4.68E+06	1.54	16164.2
PCH7Q5QJ = PCHQ57-9<br>The data from P(OOH)2-R90	8.68E+01	3.0	19560.2	4.56E+04	2.12	20427.1
PCH1Q4QJ = PCHQ14-7<br>The data from KHP-R102	3.31E+04	2.2	22040.7	1.17E+03	2.79	25658.3
PCH2Q4QJ = PCHQ24-7<br>The data from P(OOH)2-R100	3.68E+04	2.3	25336.2	1.17E+03	2.79	25658.3
PCH3Q5QJ = PCHQ35-8<br>The data from P(OOH)2-R98	3.08E+04	2.1	22174.6	1.62E+03	2.61	24927.3
PCH4Q2QJ = PCHQ24-5<br>The data from P(OOH)2-R54	2.79E+05	2.2	18906.4	1.20E+06	1.61	16268.2
PCH6Q8QJ = PCHQ68-5<br>The data from P(OOH)2-R108	3.44E+05	2.3	27888.2	2.19E+03	2.83	25308.9
PCH6Q9QJ = PCHQ58-2<br>The data from P(OOH)2-R51	1.92E+05	2.0	14936.6	8.13E+07	1.04	17564.7

PCH7Q5QJ = PCHQ57-8<br>The data from P(OOH)2-R109	4.66E+04	2.5	26899.1	1.62E+03	2.61	24927.3
PCH1Q4QJ = OH + PCH1O4Q<br>The data from KHP-R122	1.65E+07	1.2	12214.7	5.81E+04	1.94	16351.9
PCH2Q4QJ = OH + PCH2O4Q<br>The data from KHP-R114	6.54E+07	1.6	14156.3	3.02E+06	1.40	14381.2
PCH3Q5QJ = OH + PCH3O5Q<br>The data from KHP-R117	1.10E+06	2.0	13839.7	2.34E+06	1.54	13164.2
PCH5Q3QJ = OH + PCH5O3Q<br>The data from KHP-R119	1.63E+07	1.7	13605.5	2.95E+05	1.76	14669.1

[3] M.X. Liu, R.Z. Fang, C.J. Sung, K. Aljohani, A. Farooq, Y. Almarzooq, O. Mathieu, E.L. Petersen, P. Dagaut, J. Zhao, Z. Tao, L. Yang, C.W. Zhou. A comprehensive experimental and modeling study of n-propylcyclohexane oxidation. Combust. Flame 238 (2022) 111944.
