# Supplementary material for: Computational Kinetic Study on the Intramolecular H-Migration of Hydroperoxyalkylperoxy Radicals (•OOQOOH) in Normal-Alkyl Cyclohexanes
Source: Molecules. 2025 Jun 29;30(13):2805. doi: 10.3390/molecules30132805 (PMC12250757; doi:10.3390/molecules30132805)
Supplement: Supplementary file 1 [file molecules-30-02805-s001.zip › Supplemental Material-File S2.pdf]

Supplemental Material-File S2

Computational Kinetic Study on the Intramolecular H-Migration of Hydroperoxyalkylperoxy Radicals (•OOQOOH) in Normal-Alkyl Cyclohexanes

Xiaoxia Yao [1], Juanqin Li [2] and Zerong Li [1,3,*]

[1] College of Chemistry, Sichuan University, Chengdu 610064, China; yaoxxkgd@163.com
[2] College of Chemical Engineering, Sichuan University, Chengdu 610065, China; lijuanqin@scu.edu.cn
[3] Engineering Research Center of Combustion and Cooling for Aerospace Power, Ministry of Education, Sichuan University, Chengdu 610065, China
* Correspondence: lizerong@scu.edu.cn

Contents:

Cartesian coordinates for all reactants, transition states and products

R1

C	-0.89171200	0.25014500	0.90215200
C	-2.00765800	0.70041400	-0.05316700
O	-3.19496600	-0.18142600	0.09845900
O	-2.97824800	-1.41342400	-0.30108900
O	0.29006700	-0.84885000	-0.93365200
O	-0.17098300	-2.15502900	-0.50651300
H	-1.69706700	0.55712000	-1.08934600
H	-1.13634100	-2.04477500	-0.59890200
H	-1.19559700	-0.68693200	1.37292100
H	-0.76519000	0.97977400	1.70680800
C	0.47116700	0.01127200	0.21464700
C	1.04981000	1.29621500	-0.41028600
C	1.47012900	-0.60547900	1.20998700
C	2.44953900	1.09494500	-1.01044500
H	1.09306300	2.05399400	0.38122500
H	0.36630100	1.67318000	-1.17752100
C	2.86158400	-0.81162400	0.59840000
H	1.54025300	0.06210800	2.07783900
H	1.06510000	-1.55553300	1.56352900
C	3.42011800	0.49094900	0.01198500
H	2.82702400	2.05538000	-1.37589300
H	2.37327500	0.43132600	-1.87653900
H	3.53908700	-1.20832200	1.36100900
H	2.79291400	-1.56760800	-0.18983600

H	4.39311600	0.31043500	-0.45551300
H	3.59208300	1.21215100	0.82258900
C	-2.54555700	2.09920500	0.18424800
H	-2.92542100	2.19988200	1.20403100
H	-1.74329000	2.82690400	0.04507400
H	-3.35079100	2.33449200	-0.51424300

TS1

C	-1.94680200	-0.77140800	-0.19802900
C	-3.15386700	-1.47942100	0.42214400
H	-3.69886600	-0.25852800	0.65624700
H	-3.01684500	-2.01351800	1.35828000
O	-2.55058500	0.44023200	-0.74413700
O	-3.43686400	0.86807800	0.28955000
C	-0.83574700	-0.46605900	0.80869700
H	-1.18331200	0.35079400	1.44686700
H	-1.56534000	-1.29157200	-1.07955100
H	-0.69071500	-1.33361000	1.46043200
C	0.53198600	-0.10484100	0.18401000
C	1.43800500	0.56597500	1.23168800
C	1.22960500	-1.34127600	-0.42113200
C	2.84019900	0.87293700	0.69117000
H	1.50935300	-0.10336700	2.09855200
H	0.95109600	1.48269200	1.57013300
C	2.63700500	-1.03583100	-0.95501200
H	1.29337400	-2.10021200	0.36763300
H	0.61044900	-1.76096600	-1.21996100
C	3.51466600	-0.37993800	0.11831700
H	3.45222800	1.30469600	1.48938900
H	2.75737300	1.63294900	-0.09179300
H	3.09711400	-1.96309800	-1.31166400
H	2.55434500	-0.36780800	-1.81703300
H	4.49361800	-0.12611900	-0.30036900
H	3.69849500	-1.09878500	0.92853000
H	-3.84044300	-1.95706300	-0.27419100
O	0.35703700	0.75035100	-0.96614400
O	-0.21896200	2.01721500	-0.55776600
H	-1.15723100	1.83567500	-0.74464200

P1

C	-1.98144100	-0.71394500	0.08447600
C	-2.96248700	-1.70190700	0.60606800
H	-3.48404000	-1.49766000	1.53476100
C	-0.73016100	-0.56375000	0.97907000
H	-0.97787200	0.14649700	1.77415200
H	-1.69652200	-0.94828700	-0.94302000

H	-0.53136500	-1.51966100	1.47053700
C	0.56001000	-0.11443000	0.25229300
C	1.60511200	0.36137700	1.27837500
C	1.13935200	-1.24231500	-0.62644800
C	2.94228600	0.73516600	0.62614000
H	1.75711500	-0.44247600	2.00992700
H	1.19249900	1.21687400	1.81688600
C	2.48198200	-0.87018600	-1.27264900
H	1.27192400	-2.12230000	0.01410800
H	0.41355400	-1.51439500	-1.39851500
C	3.50206000	-0.41370500	-0.22232300
H	3.65929600	1.02077800	1.40247600
H	2.79131800	1.61533200	-0.00634000
H	2.86562300	-1.73085000	-1.83010200
H	2.31926200	-0.06674100	-1.99663400
H	4.43258300	-0.10628500	-0.70995000
H	3.75638400	-1.25951400	0.43111500
H	-3.08942900	-2.66901500	0.13726300
O	0.27760200	0.92162900	-0.70917300
O	-0.22189400	2.10319200	-0.03329500
H	-1.17956900	1.92749000	-0.08948100
O	-2.56936100	0.61953600	0.07038600
O	-3.54619500	0.68089400	-0.99503900
H	-4.36225900	0.49037400	-0.51022800

R2

C	1.25881000	0.67051300	-0.29331200
C	0.18923200	-0.05456400	0.54206300
C	2.64849300	0.62488700	0.36131100
O	3.65724800	0.12984400	-0.61017300
O	3.46431200	-1.12227800	-0.95697300
O	0.64230900	-1.37424600	0.88508500
O	0.78845100	-2.15196800	-0.33319000
H	0.12321300	0.40376200	1.53880300
H	2.66042400	-0.11386400	1.16436200
H	1.73970700	-2.02779800	-0.51204500
H	1.30915100	0.18933200	-1.27214300
H	0.96387200	1.70761000	-0.46175600
C	3.18582600	1.96463700	0.82700600
H	4.17675000	1.85861100	1.27225900
H	3.24800000	2.66943200	-0.00587700
H	2.51348000	2.38301600	1.58001700
C	-1.21329700	-0.06834300	-0.09330000
C	-2.18607800	-0.91985100	0.74527300

C	-1.77958600	1.35247400	-0.28959500
H	-1.11407400	-0.54072400	-1.07822200
C	-3.59726500	-0.94366700	0.14341500
H	-2.23476100	-0.50576500	1.76240900
H	-1.79398500	-1.93347300	0.84055300
C	-3.19710200	1.33136400	-0.88298700
H	-1.80335500	1.86665200	0.68179700
H	-1.13029300	1.94462600	-0.94114200
C	-4.15533100	0.47177000	-0.04961400
H	-4.26323200	-1.53138900	0.78328200
H	-3.56728900	-1.45653500	-0.82649000
H	-3.57710600	2.35463500	-0.96806400
H	-3.14986100	0.93121600	-1.90385400
H	-5.14032900	0.43403900	-0.52595600
H	-4.30108600	0.94048500	0.93250400

TS2

C	-3.81022300	0.41007100	0.87147600
C	-2.35311900	0.89234100	0.86674600
C	-1.37205200	-0.22615800	0.46685600
C	-1.77977100	-0.84615100	-0.88572200
C	-3.23773100	-1.32939900	-0.87371300
C	-4.20492100	-0.20689700	-0.47649500
H	-2.23911500	1.72315800	0.16289100
H	-2.08434700	1.28049000	1.85563500
H	-3.94401500	-0.33913000	1.66326800
H	-4.47602700	1.24324500	1.11821500
H	-1.64877100	-0.09428700	-1.67091100
H	-1.12079300	-1.68222900	-1.13994500
H	-3.50247200	-1.72904300	-1.85789500
H	-3.33914500	-2.16075600	-0.16323500
H	-4.18941400	0.57244500	-1.24888000
H	-5.23098100	-0.58663200	-0.43539200
H	-1.43133900	-1.01413600	1.23211200
C	0.08278100	0.28785500	0.49451200
H	0.23557800	0.84689500	1.42475100
C	1.12426300	-0.84385400	0.40848300
H	1.01433100	-1.39251100	-0.53103000
H	0.91979800	-1.54937000	1.22259600
C	2.58219100	-0.41594000	0.53559700
H	2.71568800	0.32838300	1.32806100
O	3.01430600	0.16650100	-0.72060500
O	4.42966200	0.23450200	-0.57719800
C	3.58140700	-1.55372300	0.73079200
H	3.44465100	-2.42359100	0.08900200

H	4.49866000	-0.77995100	0.08538400
H	3.90303600	-1.78343400	1.74328600
O	0.20412700	1.22085200	-0.58549000
O	1.20360300	2.21418500	-0.24385700
H	1.98414500	1.84326700	-0.69190700

P2

C	3.65474000	0.49324300	-0.79495100
C	2.18620200	0.93853800	-0.80273400
C	1.22118000	-0.23402500	-0.54542600
C	1.59542800	-0.96744800	0.75844500
C	3.06595900	-1.41160200	0.76194800
C	4.01613100	-0.23448200	0.50589500
H	2.02611100	1.69763600	-0.02941700
H	1.94352800	1.41207100	-1.76059200
H	3.83736600	-0.17698700	-1.64548400
H	4.30658200	1.36075500	-0.93879400
H	1.41700200	-0.29317200	1.60357700
H	0.95137400	-1.83986000	0.90696800
H	3.30431200	-1.89333300	1.71559200
H	3.21610100	-2.17143300	-0.01644600
H	3.95192000	0.47075500	1.34433400
H	5.05269600	-0.58499200	0.47096300
H	1.32643000	-0.94662400	-1.37689300
C	-0.24201900	0.24809100	-0.57982600
H	-0.37359300	0.89391100	-1.45236800
C	-1.27124100	-0.88943500	-0.61055400
H	-1.17755800	-1.50103000	0.29009600
H	-1.03660800	-1.54229900	-1.45815900
C	-2.73669300	-0.46667200	-0.75833900
H	-2.86497400	0.14441400	-1.66256500
C	-3.67685400	-1.61556500	-0.77810800
H	-3.37757600	-2.59219800	-0.41950100
H	-4.72168100	-1.44250700	-1.00224300
O	-0.45804500	1.09832400	0.57912200
O	-1.09943100	2.33022600	0.16693700
H	-2.03495200	2.04442000	0.17702500
O	-3.14232700	0.51665100	0.25237900
O	-2.88157700	-0.01056000	1.57741700
H	-1.97243300	0.31948900	1.70896600

R3

C	1.93739100	-1.55134300	-0.78692500
C	0.44319400	-1.20385500	-0.84713700
C	-0.03930700	-0.59724700	0.48504700

C	0.79715800	0.65158100	0.85696600
C	2.29116700	0.30247200	0.90406700
C	2.78444100	-0.33057300	-0.40482500
H	0.26977900	-0.48392600	-1.65140700
H	-0.14489600	-2.09378300	-1.09064500
H	2.09647800	-2.35160200	-0.05184500
H	2.26164400	-1.94841400	-1.75367000
H	0.47656500	1.04279600	1.82859100
H	2.85018000	1.21333200	1.13103600
H	2.45054700	-0.39310700	1.73672700
H	2.73143800	0.41528100	-1.20423000
H	3.83679500	-0.61361700	-0.30509900
H	0.18646600	-1.32775000	1.27367400
C	-1.57680900	-0.39857400	0.55984100
H	-1.82619400	0.55229500	1.03056400
O	0.68943300	1.71929700	-0.09218300
O	-0.50283600	2.49168900	0.20033600
H	-1.11353300	2.12833700	-0.46887000
O	-2.19330600	-0.34179300	-0.79136200
O	-2.07127900	0.81283800	-1.40386300
C	-2.30421800	-1.55186800	1.23237100
H	-2.00829900	-1.60480400	2.28245000
H	-2.05554100	-2.50408100	0.75705000
H	-3.38562200	-1.41076000	1.18581800

TS3

C	-1.59365800	-0.12290100	-0.57274900
C	-2.68329400	-1.19750500	-0.54256500
H	-3.10905600	-0.64023200	0.63763400
H	-2.39376800	-2.23428000	-0.39737400
O	-2.23390300	0.99397700	0.08972700
O	-2.86822300	0.38995500	1.21323100
H	-1.38244400	0.24633200	-1.57975700
C	-0.28899900	-0.55457700	0.11865300
C	0.47808800	-1.61172200	-0.70018000
C	0.60512600	0.65925500	0.43604800
H	-0.55669400	-0.98619200	1.09064800
C	1.78494200	-2.02424400	-0.00585100
H	0.70636000	-1.20229000	-1.69029200
H	-0.14986900	-2.49400400	-0.86029700
C	1.91846900	0.24958700	1.10519500
H	0.04852300	1.35712200	1.06803600
C	2.67330400	-0.80923800	0.29151300
H	2.32270300	-2.74455200	-0.63020200
H	1.54689900	-2.54127100	0.93307000

H	2.52954800	1.14388900	1.24542100
H	1.68344300	-0.13779100	2.10448400
H	3.57413800	-1.12044000	0.82910100
H	3.00527500	-0.36263000	-0.65127700
H	-3.51679600	-1.05588900	-1.22777800
O	0.83650800	1.29735100	-0.83486600
O	1.46849100	2.57777200	-0.59120100
H	0.70488600	3.16786400	-0.66693300

P3

C	-1.38464000	-0.99975700	-0.17527800
C	-1.85303500	-2.40592000	-0.11653100
H	-1.34474200	-3.14399000	0.49165600
H	-1.50360600	-0.57915700	-1.17739600
C	0.06269200	-0.80557200	0.30594100
C	1.07182300	-1.48830500	-0.63875100
C	0.40602500	0.68071700	0.49783000
H	0.14432000	-1.27083300	1.29814900
C	2.51790800	-1.28015200	-0.16263800
H	0.95490900	-1.06835700	-1.64447200
H	0.85088400	-2.55737300	-0.71632600
C	1.85112400	0.89729200	0.95051000
H	-0.28924600	1.11518200	1.21765600
C	2.84816600	0.20909400	0.00920800
H	3.21014700	-1.74249300	-0.87330900
H	2.66130800	-1.79865400	0.79473600
H	2.03781400	1.97337700	1.00068200
H	1.96154200	0.50348200	1.96930200
H	3.86800800	0.33091000	0.38690900
H	2.80937000	0.70416600	-0.96669200
H	-2.80304600	-2.67345700	-0.56180200
O	0.17171400	1.31567100	-0.77686400
O	-0.29436000	2.66308500	-0.53975600
H	-1.24342700	2.48312900	-0.42632400
O	-2.32361500	-0.25248100	0.67974400
O	-2.65724000	1.02071500	0.04613200
H	-3.60661500	0.90376000	-0.08856800

R4

C	-0.01423400	1.75542600	-0.05421300
C	-1.53645500	1.82032100	0.13698800
C	-2.27400100	0.85586500	-0.79835500
C	-1.74954600	-0.57463300	-0.65849200
C	-0.22740000	-0.65180200	-0.81028100
C	0.52200700	0.32120400	0.12309300

H	-3.34720600	0.86315700	-0.59815400
H	-1.78081900	1.56660000	1.17329000
H	-1.88773200	2.84310700	-0.02920200
H	0.24435400	2.11491300	-1.06027600
H	0.46991400	2.43199000	0.65478800
H	-2.22947800	-1.22978100	-1.39620200
H	0.10245300	-1.67689400	-0.63347600
H	0.01069000	-0.41147800	-1.85395100
H	0.33686000	0.01205600	1.15857800
H	-2.13933400	1.16479600	-1.84253700
C	2.89174500	1.13075100	0.78130700
H	3.94284600	0.88017700	0.63053600
H	2.75449200	2.18502800	0.53440900
H	2.63896400	0.98135000	1.83432500
O	-2.00790500	-1.11197400	0.65023400
O	-3.44780300	-1.19611200	0.82832800
H	-3.56021100	-2.15644500	0.83305200
C	2.03313100	0.24980500	-0.11247300
H	2.27223300	0.42147000	-1.16652900
O	2.40686300	-1.16060900	0.14272900
O	3.58640600	-1.46026300	-0.35680200

TS4

C	1.85891600	-0.08789100	0.68132200
C	2.29023500	-1.54940900	0.52459400
H	2.83381400	-1.18605300	-0.68570400
H	1.52534100	-2.30651300	0.37859700
O	2.89671700	0.62867500	-0.03019800
O	3.07092600	-0.13597600	-1.21736100
H	1.94953600	0.27353900	1.71308200
C	0.45124600	0.19761700	0.13956900
C	-0.62123700	-0.44229700	1.03944900
C	0.19375900	1.70684800	-0.00272900
H	0.38115600	-0.25581100	-0.85501500
C	-2.04153700	-0.16800300	0.53361100
H	-0.54077500	-0.03669700	2.05687800
H	-0.48892500	-1.52322400	1.11651900
C	-1.21084200	1.98316100	-0.55469200
H	0.30390600	2.18620200	0.98077000
H	0.95269300	2.14788400	-0.65159500
C	-2.29975400	1.32462800	0.30447500
H	-2.77347900	-0.57507000	1.24270700
H	-1.38846500	3.06163600	-0.60761400
H	-1.27877600	1.60166800	-1.57813100
H	-3.28711600	1.45300900	-0.14796500

H	-2.33500100	1.80208900	1.29071400
H	3.13710400	-1.87142500	1.12740600
O	-2.27362400	-0.78068000	-0.74587600
O	-2.15016200	-2.22114400	-0.58558100
H	-3.06658400	-2.48616200	-0.74211900

P4

C	1.81277900	-0.31158700	0.37072200
C	2.02921900	-1.78512700	0.38166500
H	1.83743100	-2.35512900	-0.52107500
H	2.07195900	0.12660200	1.34110100
C	0.37939900	0.10832600	-0.02135000
C	-0.65147400	-0.52580000	0.92947800
C	0.21366500	1.63825500	-0.05091700
H	0.19256100	-0.28263600	-1.02853700
C	-2.08834600	-0.12079500	0.58521600
H	-0.44800600	-0.20948900	1.96103900
H	-0.58130200	-1.61489600	0.90910600
C	-1.21514100	2.03998800	-0.44143000
H	0.44918600	2.03951400	0.94492200
H	0.93468200	2.07619500	-0.74238700
C	-2.25978600	1.39793800	0.48193300
H	-2.77752900	-0.53007900	1.33495600
H	-1.31878700	3.12911300	-0.41126300
H	-1.40970100	1.73368700	-1.47388700
H	-3.27495900	1.62328600	0.14296600
H	-2.16174200	1.80567800	1.49477500
H	2.37412600	-2.30945200	1.26314300
O	-2.48719400	-0.61971800	-0.70361200
O	-2.46606000	-2.07236900	-0.65538300
H	-3.41448500	-2.25625100	-0.69424300
O	2.65650700	0.31437600	-0.62925100
O	4.02950700	0.24223500	-0.17225000
H	4.34259000	-0.53952300	-0.64854900

R5

C	1.01519200	-0.50567700	0.99341500
C	2.06115700	-1.18356400	0.10092400
O	3.37170700	-0.52938500	0.20080400
O	3.36914200	0.68024300	-0.31626500
O	0.04235900	0.56823500	-0.97198300
O	0.69510700	1.82472000	-0.65936300
H	1.78217300	-1.14100200	-0.95204900
H	2.28508200	-2.20944200	0.39081800
H	1.63289100	1.56309500	-0.72371600

H	1.45057000	0.41277500	1.39305400
H	0.77183600	-1.13789700	1.85051900
C	-0.28552800	-0.13346900	0.24871200
C	-1.03972200	-1.37769100	-0.26103200
C	-1.19437300	0.72282100	1.14765500
C	-2.38415200	-1.03689900	-0.92090600
H	-1.20766600	-2.03274100	0.60223200
H	-0.40742900	-1.93097100	-0.96273200
C	-2.53099100	1.06552500	0.47750900
H	-1.37495300	0.16639200	2.07583500
H	-0.65758700	1.63485900	1.41555000
C	-3.26848700	-0.19450300	0.00694000
H	-2.89519200	-1.96413000	-1.19914200
H	-2.19732200	-0.48506700	-1.84665300
H	-3.15321200	1.63322500	1.17639500
H	-2.33943300	1.71840900	-0.37958900
H	-4.19657900	0.07820700	-0.50498800
H	-3.55828000	-0.79547000	0.87961800

TS5

O	3.04660100	-0.69071100	-0.77440500
H	1.91201600	-0.37317300	-1.14169900
O	2.97351600	0.08821200	0.41504100
C	2.19930500	1.23126900	0.00420000
H	2.82372200	1.90379500	-0.59012800
H	1.86655700	1.71649500	0.92317100
C	1.06853200	0.65142200	-0.85479100
H	0.90813800	1.14124400	-1.81502400
O	0.03025400	-0.20322400	1.17695300
O	0.74838000	-1.46217600	1.20047400
H	1.66857800	-1.14170400	1.23036500
C	-0.21776600	0.20937500	-0.18182300
C	-1.15265400	1.43594800	0.00649100
C	-0.93103700	-0.89390600	-0.98844100
C	-2.51674400	1.04904800	0.59738400
H	-1.29126200	1.90073100	-0.97578000
H	-0.65400400	2.17120500	0.64496700
C	-2.29169500	-1.26904200	-0.38940000
H	-1.05905800	-0.53145600	-2.01581800
H	-0.27776200	-1.76657700	-1.02929300
C	-3.20171600	-0.04449900	-0.23102200
H	-3.14678300	1.94305300	0.64830800
H	-2.37386700	0.69751800	1.62272300
H	-2.77051700	-2.02000200	-1.02534400
H	-2.13113300	-1.73575300	0.58707000

H	-4.14661900	-0.33335700	0.23927400
H	-3.45536800	0.35347900	-1.22287500

P5

C	2.10595500	-0.96001600	0.43824700
H	2.70186300	-1.63235700	1.06323900
H	1.93651600	-1.41564200	-0.54034400
C	0.83454000	-0.56621600	1.09640100
H	0.79619000	-0.51945800	2.17909700
O	0.02377600	0.44174500	-0.94639400
O	0.65589100	1.72257600	-0.69372100
H	1.58282100	1.44295500	-0.57032200
C	-0.37755900	-0.16920500	0.30344300
C	-1.15529900	-1.42578800	-0.17506700
C	-1.30369300	0.76268600	1.10913200
C	-2.45072600	-1.07301900	-0.92149400
H	-1.38533000	-2.02606500	0.71223500
H	-0.49808100	-2.02794100	-0.80903300
C	-2.58877800	1.11048200	0.34971000
H	-1.55015000	0.25611300	2.05059800
H	-0.74876200	1.66791100	1.36030300
C	-3.34781900	-0.14853400	-0.08931500
H	-2.98135900	-1.99758500	-1.17191000
H	-2.19609100	-0.58367400	-1.86563200
H	-3.22514900	1.73775200	0.98203600
H	-2.32864200	1.70711100	-0.52990500
H	-4.23817800	0.12655800	-0.66344700
H	-3.70334900	-0.68852000	0.79886700
O	2.87216400	0.26273800	0.22761500
O	4.04752500	-0.08909200	-0.55735300
H	4.74313100	0.06677500	0.09670400

R6

C	-0.89171200	0.25014500	0.90215200
C	-2.00765800	0.70041400	-0.05316700
O	-3.19496600	-0.18142600	0.09845900
O	-2.97824800	-1.41342400	-0.30108900
O	0.29006700	-0.84885000	-0.93365200
O	-0.17098300	-2.15502900	-0.50651300
H	-1.69706700	0.55712000	-1.08934600
H	-1.13634100	-2.04477500	-0.59890200
H	-1.19559700	-0.68693200	1.37292100
H	-0.76519000	0.97977400	1.70680800
C	0.47116700	0.01127200	0.21464700
C	1.04981000	1.29621500	-0.41028600

C	1.47012900	-0.60547900	1.20998700
C	2.44953900	1.09494500	-1.01044500
H	1.09306300	2.05399400	0.38122500
H	0.36630100	1.67318000	-1.17752100
C	2.86158400	-0.81162400	0.59840000
H	1.54025300	0.06210800	2.07783900
H	1.06510000	-1.55553300	1.56352900
C	3.42011800	0.49094900	0.01198500
H	2.82702400	2.05538000	-1.37589300
H	2.37327500	0.43132600	-1.87653900
H	3.53908700	-1.20832200	1.36100900
H	2.79291400	-1.56760800	-0.18983600
H	4.39311600	0.31043500	-0.45551300
H	3.59208300	1.21215100	0.82258900
C	-2.54555700	2.09920500	0.18424800
H	-2.92542100	2.19988200	1.20403100
H	-1.74329000	2.82690400	0.04507400
H	-3.35079100	2.33449200	-0.51424300

TS6

C	-2.09130000	0.70822300	-0.28960300
C	-0.92576600	0.44304900	0.68180000
H	-1.59416300	-0.64645000	1.11255400
H	-0.88665500	1.09520900	1.55468700
O	-2.65037800	-0.61521100	-0.51718300
O	-2.64403200	-1.20711100	0.78199800
H	-1.74040100	1.01962300	-1.27651500
C	-3.13670800	1.66422800	0.26536800
H	-2.70558000	2.65783800	0.41573400
H	-3.51783300	1.29773200	1.22015200
C	0.44623700	0.11469600	0.12096500
C	1.18239800	1.43281300	-0.24606300
C	1.28462100	-0.71239500	1.11605800
C	2.61887000	1.18784700	-0.73229700
H	1.19600100	2.06486400	0.64863000
H	0.60600500	1.96409500	-1.00927200
C	2.71628800	-0.94753300	0.61981600
H	1.30122900	-0.17541000	2.07245900
H	0.77785800	-1.66366400	1.28431300
C	3.42641900	0.36946900	0.28225600
H	3.10013300	2.15402000	-0.91598800
H	2.58587300	0.65810500	-1.68812000
H	3.27611300	-1.49759700	1.38253200
H	2.68178700	-1.58419200	-0.26933800
H	4.42802000	0.16962100	-0.11066000

H	3.56257200	0.95729200	1.20011900
H	-3.97135200	1.75239400	-0.43275900
O	0.33787800	-0.54602700	-1.15532200
O	-0.17970100	-1.88935200	-0.98635300
H	-1.13609800	-1.72425600	-1.08344600

P6

C	-1.97370200	0.67346700	0.02324300
H	-1.76172000	0.76786900	-1.04583900
C	-0.70555300	0.62016600	0.80250200
H	-0.72921000	0.91223100	1.84768300
O	0.33043700	-0.87886800	-0.78858300
O	-0.18045500	-2.07620400	-0.14972100
H	-1.13403900	-1.86767200	-0.12002400
C	0.58696100	0.14258100	0.20474600
C	1.25249100	1.26505400	-0.63720900
C	1.55848500	-0.36497600	1.28842000
C	2.61951100	0.84948100	-1.20210200
H	1.36662400	2.13970100	0.01300200
H	0.57356300	1.55114000	-1.44572300
C	2.91761800	-0.77804300	0.71186900
H	1.69128300	0.44001000	2.02194100
H	1.08992300	-1.20428800	1.80475100
C	3.56063500	0.35632800	-0.09653300
H	3.06191300	1.70097400	-1.72970600
H	2.47356900	0.05548800	-1.93959800
H	3.57872000	-1.08656900	1.52804300
H	2.77756000	-1.65277400	0.06948500
H	4.50787300	0.02208800	-0.53134300
H	3.80254000	1.19072600	0.57586000
O	-2.57423200	-0.65678200	0.19652900
O	-3.65197000	-0.80061000	-0.77889600
H	-4.41275700	-0.86414200	-0.18533500
C	-2.93948100	1.75456100	0.48810900
H	-3.87282600	1.69652600	-0.07481600
H	-3.15812500	1.64176400	1.55351900
H	-2.50691600	2.74493400	0.32520200

R7

C	1.25881000	0.67051300	-0.29331200
C	0.18923200	-0.05456400	0.54206300
C	2.64849300	0.62488700	0.36131100
O	3.65724800	0.12984400	-0.61017300
O	3.46431200	-1.12227800	-0.95697300
O	0.64230900	-1.37424600	0.88508500

O	0.78845100	-2.15196800	-0.33319000
H	0.12321300	0.40376200	1.53880300
H	2.66042400	-0.11386400	1.16436200
H	1.73970700	-2.02779800	-0.51204500
H	1.30915100	0.18933200	-1.27214300
H	0.96387200	1.70761000	-0.46175600
C	3.18582600	1.96463700	0.82700600
H	4.17675000	1.85861100	1.27225900
H	3.24800000	2.66943200	-0.00587700
H	2.51348000	2.38301600	1.58001700
C	-1.21329700	-0.06834300	-0.09330000
C	-2.18607800	-0.91985100	0.74527300
C	-1.77958600	1.35247400	-0.28959500
H	-1.11407400	-0.54072400	-1.07822200
C	-3.59726500	-0.94366700	0.14341500
H	-2.23476100	-0.50576500	1.76240900
H	-1.79398500	-1.93347300	0.84055300
C	-3.19710200	1.33136400	-0.88298700
H	-1.80335500	1.86665200	0.68179700
H	-1.13029300	1.94462600	-0.94114200
C	-4.15533100	0.47177000	-0.04961400
H	-4.26323200	-1.53138900	0.78328200
H	-3.56728900	-1.45653500	-0.82649000
H	-3.57710600	2.35463500	-0.96806400
H	-3.14986100	0.93121600	-1.90385400
H	-5.14032900	0.43403900	-0.52595600
H	-4.30108600	0.94048500	0.93250400

TS7

C	2.78579000	-0.27454300	-0.55545100
C	0.21391000	0.22618500	-0.75408900
C	1.32592800	-0.70078100	-0.30618800
H	1.58002500	-0.61784800	1.01957200
H	1.11662900	-1.75333500	-0.48862300
O	3.11569000	0.50489800	0.62717700
O	2.54386600	-0.25069300	1.69406800
H	2.86934900	0.44127100	-1.37699000
C	-1.13138700	0.04416200	-0.01889800
C	-2.18410800	1.02635000	-0.56487500
C	-1.64998300	-1.40484800	-0.09759800
H	-0.94851500	0.29173300	1.03270300
C	-3.53594800	0.86726500	0.14319900
H	-2.31677500	0.84474400	-1.64091800
H	-1.81683900	2.04868000	-0.46312700
C	-3.00994200	-1.56344500	0.60091500

H	-1.75029700	-1.69716600	-1.15260100
H	-0.93456300	-2.09841300	0.35458700
C	-4.05127100	-0.57510700	0.06155100
H	-4.26547800	1.55757400	-0.29199700
H	-3.42734600	1.15454400	1.19675600
H	-3.36484500	-2.59285100	0.48760800
H	-2.87822200	-1.39617200	1.67739800
H	-4.99049500	-0.67811400	0.61434900
H	-4.27623300	-0.82072000	-0.98465300
H	0.04568000	0.03499400	-1.82835500
O	0.64192300	1.59171200	-0.79169000
O	0.78774400	2.08527800	0.56666500
H	1.72757200	1.88035200	0.72974000
C	3.73577500	-1.44973300	-0.73019300
H	3.48278700	-2.01577200	-1.63099900
H	4.76186300	-1.08975000	-0.82659800
H	3.67934300	-2.11634300	0.13215400

P7

C	-2.50094200	1.13919900	-0.27366200
C	-0.24611600	-0.20783100	-0.29289500
C	-1.21670400	0.71416900	0.36839600
H	-0.98947300	1.08347400	1.36294500
H	-2.29417700	1.92996400	-1.01469700
C	1.20377000	-0.04116400	0.18230800
C	2.13382900	-1.10630800	-0.42474100
C	1.72721600	1.37313900	-0.13823100
H	1.20199100	-0.17163400	1.27208000
C	3.58717900	-0.91939900	0.03238500
H	2.08696400	-1.03763800	-1.52055600
H	1.77604500	-2.10266900	-0.15978700
C	3.18227100	1.56297000	0.31551500
H	1.66329400	1.53740000	-1.22298000
H	1.09127900	2.13280300	0.32773200
C	4.10620500	0.49098200	-0.27704900
H	4.22487300	-1.67152700	-0.44279200
H	3.65013300	-1.09828400	1.11352700
H	3.52996600	2.56288900	0.03656900
H	3.22623600	1.51248600	1.41104700
H	5.12429600	0.61591700	0.10552800
H	4.16325100	0.62503600	-1.36516700
H	-0.30119400	-0.09532900	-1.38366500
O	-0.68751000	-1.60536200	-0.17183600
O	-0.78060900	-1.95143600	1.23454400
H	-1.69468800	-1.68610100	1.42860300

O	-3.04297400	0.13053100	-1.14471500
O	-3.37149400	-1.05214500	-0.35871900
H	-2.59507200	-1.59887700	-0.57769800
C	-3.54280800	1.65267200	0.71910900
H	-3.14081700	2.48946900	1.29658400
H	-4.43143400	1.99093300	0.18394800
H	-3.83417000	0.85840500	1.40777200

R8

C	-2.31185600	-0.74620200	-0.98249100
C	-3.08724700	0.51509700	-1.37593900
C	-1.05305900	-0.43193200	-0.15927600
O	-1.07553000	-1.20750600	1.10760600
O	-2.04304200	-0.84181400	1.91773600
O	-3.36047800	1.36348700	-0.26811200
O	-4.24614000	0.64806800	0.62992500
H	-4.01990800	0.24594200	-1.88280700
H	-2.50147200	1.16003000	-2.03979300
H	-1.07289000	0.61027700	0.16271800
H	-3.59971000	0.27067500	1.25587500
H	-2.97160300	-1.38320400	-0.39168400
H	-2.05363500	-1.30796200	-1.88522800
C	0.26156200	-0.80227200	-0.83113600
H	0.26173100	-1.88671200	-0.99444200
H	0.25971500	-0.34192500	-1.82698300
C	1.53849200	-0.39229300	-0.07691300
C	2.77496800	-1.06006400	-0.70697200
C	1.73233300	1.13497700	-0.02004200
H	1.45181700	-0.76114100	0.95344000
C	4.07385600	-0.67480300	0.01447600
H	2.84371600	-0.76015900	-1.76210100
H	2.65047200	-2.14852800	-0.70065300
C	3.02851200	1.52585900	0.70541900
H	1.76134400	1.52659700	-1.04660600
H	0.88086500	1.61545500	0.47225900
C	4.25397900	0.84779900	0.07901000
H	4.93073600	-1.13872200	-0.48438800
H	4.05093600	-1.07913200	1.03447600
H	3.14793300	2.61383400	0.69377600
H	2.95216900	1.23272700	1.76021800
H	5.15697600	1.09935700	0.64430800
H	4.40308600	1.23771000	-0.93643800

TS8

C	1.03154500	-0.04120200	0.29262000

C	2.28726800	-0.48568200	1.06542900
H	2.44082100	-1.46833700	0.13905400
H	2.11491700	-1.01386200	2.00360100
O	1.31204100	-0.46813200	-1.07057600
O	1.87529200	-1.76452400	-0.90565200
C	-0.25375700	-0.66452400	0.83112700
H	-0.13192200	-1.75206300	0.80129300
H	0.96565800	1.04678300	0.21750200
H	-0.35860600	-0.38394700	1.88712900
C	-1.52403900	-0.26636600	0.06171400
C	-2.69818000	-1.19043000	0.43541800
C	-1.91976200	1.20614500	0.28238800
H	-1.31552800	-0.40716600	-1.00694700
C	-3.98649700	-0.82495600	-0.31458100
H	-2.87700900	-1.12162500	1.51771500
H	-2.42660400	-2.23166900	0.23060300
C	-3.20660500	1.57926600	-0.46855000
H	-2.06761700	1.37514100	1.35847000
H	-1.11051500	1.87488900	-0.02890300
C	-4.36769100	0.64590400	-0.10071400
H	-4.80386700	-1.47972500	0.00440600
H	-3.84150900	-1.00833500	-1.38686800
H	-3.47310900	2.61989500	-0.25739600
H	-3.02240100	1.51846300	-1.54863500
H	-5.25668100	0.89714200	-0.68803400
H	-4.63614200	0.80043900	0.95261800
C	3.46902300	0.45254300	1.10908800
H	3.36472800	1.14047100	1.96147200
H	4.40692300	-0.09925200	1.24610400
O	3.56633300	1.32019400	-0.00625500
O	3.98628700	0.53019100	-1.14766900
H	3.12101000	0.28674900	-1.52560500

P8

C	1.00100000	-0.12921600	0.46991000
H	0.77493700	0.78932500	1.02249100
C	2.18004100	-0.83451000	1.05976900
H	2.06587400	-1.86581300	1.37467900
C	3.53226100	-0.21217400	1.16118100
H	3.55590800	0.61001000	1.89153700
H	4.28557100	-0.95120700	1.44537400
O	3.96632500	0.45375700	-0.04345700
O	4.01892900	-0.52634400	-1.11052000
H	3.09489800	-0.48724200	-1.42504900
C	-0.23721100	-1.02239200	0.36545000

H	-0.39950300	-1.49224500	1.34358100
H	-0.00402700	-1.83431200	-0.33370800
C	-1.51832300	-0.30621700	-0.08913900
C	-2.59692300	-1.32741600	-0.49596500
C	-2.07421400	0.65204300	0.98088200
H	-1.26466700	0.28531400	-0.97748100
C	-3.89525300	-0.64877400	-0.95436000
H	-2.81367000	-1.98157600	0.36053800
H	-2.21111200	-1.97477000	-1.29126100
C	-3.37142700	1.33775000	0.52639000
H	-2.26871200	0.08240400	1.90077100
H	-1.33115000	1.41439400	1.23578500
C	-4.43658100	0.31458800	0.11003300
H	-4.64745500	-1.40563400	-1.19950100
H	-3.70110300	-0.09142200	-1.87957000
H	-3.75346400	1.98135000	1.32541000
H	-3.14935900	1.99533400	-0.32356900
H	-5.32991400	0.82681400	-0.26162900
H	-4.75085400	-0.25951900	0.99167000

R9

C	-2.31185600	-0.74620200	-0.98249100
C	-3.08724700	0.51509700	-1.37593900
C	-1.05305900	-0.43193200	-0.15927600
O	-1.07553000	-1.20750600	1.10760600
O	-2.04304200	-0.84181400	1.91773600
O	-3.36047800	1.36348700	-0.26811200
O	-4.24614000	0.64806800	0.62992500
H	-4.01990800	0.24594200	-1.88280700
H	-2.50147200	1.16003000	-2.03979300
H	-1.07289000	0.61027700	0.16271800
H	-3.59971000	0.27067500	1.25587500
H	-2.97160300	-1.38320400	-0.39168400
H	-2.05363500	-1.30796200	-1.88522800
C	0.26156200	-0.80227200	-0.83113600
H	0.26173100	-1.88671200	-0.99444200
H	0.25971500	-0.34192500	-1.82698300
C	1.53849200	-0.39229300	-0.07691300
C	2.77496800	-1.06006400	-0.70697200
C	1.73233300	1.13497700	-0.02004200
H	1.45181700	-0.76114100	0.95344000
C	4.07385600	-0.67480300	0.01447600
H	2.84371600	-0.76015900	-1.76210100
H	2.65047200	-2.14852800	-0.70065300

C	3.02851200	1.52585900	0.70541900
H	1.76134400	1.52659700	-1.04660600
H	0.88086500	1.61545500	0.47225900
C	4.25397900	0.84779900	0.07901000
H	4.93073600	-1.13872200	-0.48438800
H	4.05093600	-1.07913200	1.03447600
H	3.14793300	2.61383400	0.69377600
H	2.95216900	1.23272700	1.76021800
H	5.15697600	1.09935700	0.64430800
H	4.40308600	1.23771000	-0.93643800

TS9

C	1.11508400	-0.15033000	0.18326000
C	-0.20393100	0.32685300	0.81513700
H	0.22213700	1.58248500	0.63630600
H	-0.29772000	0.17251400	1.89171700
O	1.41365300	0.92390300	-0.75059100
O	1.14575700	2.10967500	-0.00458000
H	0.98320700	-1.02931100	-0.45609500
C	2.22577400	-0.37765800	1.20957100
H	1.82701800	-1.03132800	1.99552200
H	2.46332400	0.58157300	1.67893200
C	3.51297400	-1.03134300	0.69330400
H	4.18118200	-1.21422400	1.54107800
H	3.30846400	-1.99016300	0.20337800
C	-1.48076300	0.13869500	0.03613400
C	-1.99122000	-1.32189900	0.15904600
C	-2.57917400	1.12448600	0.48252500
H	-1.25952900	0.32852500	-1.02243600
C	-3.29979200	-1.52990600	-0.61792800
H	-2.15615600	-1.55078200	1.21991400
H	-1.22793300	-2.01964300	-0.19972500
C	-3.89047700	0.91098400	-0.28593400
H	-2.76041300	0.99456000	1.55808000
H	-2.22426000	2.15050400	0.34458900
C	-4.38391600	-0.53789400	-0.17730000
H	-3.64678200	-2.55928600	-0.48215900
H	-3.10557200	-1.40459000	-1.69037100
H	-4.65408900	1.60147600	0.08530900
H	-3.73226700	1.16149400	-1.34241700
H	-5.28836600	-0.67610400	-0.77775400
H	-4.66475300	-0.74827400	0.86282000
O	4.29540700	-0.22428000	-0.17479900
O	3.73976400	-0.32149200	-1.51046300
H	3.12850900	0.43619400	-1.51010900

P9

C	1.05418700	0.62335800	0.09972000
C	-0.27687800	0.83365000	-0.52618900
H	-0.32149200	1.23415300	-1.53565400
H	1.05358100	0.99480000	1.13559000
C	2.20326800	1.25620900	-0.69082100
H	2.00910500	2.33303700	-0.75109000
H	2.16695700	0.87752700	-1.71615200
C	3.61366100	1.06343600	-0.12971500
H	4.34082000	1.52476000	-0.80512200
H	3.73439400	1.50556700	0.86153700
C	-1.53610400	0.33602000	0.10047500
C	-2.63963400	1.42050900	0.11734200
C	-2.05907500	-0.94090600	-0.61332700
H	-1.31748400	0.05642500	1.14021900
C	-3.94492000	0.90607200	0.74087000
H	-2.83026500	1.74492900	-0.91417100
H	-2.28300800	2.30185400	0.66022900
C	-3.36268100	-1.45149500	0.01602900
H	-2.23151600	-0.70455600	-1.67145700
H	-1.28593800	-1.71179800	-0.58478300
C	-4.44650400	-0.36573600	0.04316200
H	-4.70942300	1.68889100	0.70077200
H	-3.77539900	0.69138200	1.80389600
H	-3.71837800	-2.32935800	-0.53316500
H	-3.16026900	-1.78795800	1.04093200
H	-5.34714500	-0.73966200	0.54071800
H	-4.73789100	-0.12111900	-0.98662000
O	4.00839500	-0.31739500	-0.04220900
O	3.84037300	-0.76830100	1.32559100
H	2.89221000	-1.00966800	1.31212700
O	1.29401500	-0.80400200	0.37322600
O	1.53058100	-1.51032400	-0.87153000
H	2.50229800	-1.43573800	-0.92370800

R10

C	0.57221800	0.15048700	0.99770900
C	1.80066000	-0.18019600	0.13656700
O	2.79715900	0.91717100	0.24434800
O	2.40438600	2.03204300	-0.32851100
O	-0.68263300	0.82425400	-0.98989300
O	-0.47383500	2.22800800	-0.69481100
H	1.52943500	-0.19666000	-0.92113000
H	0.49913600	2.28159800	-0.75216500

H	0.67841200	1.16838100	1.37785100
H	0.53347100	-0.50774300	1.87027900
C	-0.77196500	0.06873800	0.23982300
C	-1.08283300	-1.35654500	-0.25866200
C	-1.91592600	0.59581500	1.12453400
C	-2.46133500	-1.47260100	-0.92665500
H	-1.03663000	-2.02413100	0.61006900
H	-0.30291300	-1.68192900	-0.95418200
C	-3.28663900	0.48444300	0.44519300
H	-1.91546700	0.02013600	2.05853900
H	-1.70272200	1.63458800	1.38376600
C	-3.57649500	-0.95058400	-0.01230100
H	-2.64489500	-2.51763600	-1.19631500
H	-2.45521300	-0.89880300	-1.85782600
H	-4.06356700	0.82952300	1.13460700
H	-3.30829200	1.15510100	-0.41927000
H	-4.53929600	-0.99666400	-0.53074100
H	-3.66356800	-1.60359200	0.86678600
C	2.56003600	-1.43737400	0.53945700
H	2.93306300	-1.30290400	1.56108600
H	1.83149500	-2.25313800	0.58304100
C	3.70685600	-1.80471600	-0.40574100
H	4.45021600	-1.00656500	-0.45245200
H	4.20987300	-2.71439800	-0.06964200
H	3.33939100	-1.98176800	-1.42091100

TS10

C	-1.71824000	-0.37316200	-0.06310200
C	-2.99811300	-0.85328600	0.64452600
H	-3.34501600	0.43104200	0.78387900
H	-2.86327000	-1.25646600	1.64777900
O	-2.16416800	0.85719000	-0.70500200
O	-2.94087200	1.50091200	0.30497200
C	-0.54277300	-0.14010300	0.88739900
H	-0.75271600	0.77192700	1.45250700
H	-1.44354500	-1.01908900	-0.90124300
H	-0.49778300	-0.95669600	1.61531000
C	0.84086700	-0.03384800	0.20457300
C	1.86495200	0.59476200	1.16628200
C	1.34015300	-1.40636000	-0.29398200
C	3.27804200	0.64971600	0.57233100
H	1.87045300	0.00424000	2.09139800
H	1.52136900	1.59820400	1.42520400
C	2.75820600	-1.35285700	-0.88156100
H	1.32363800	-2.09152800	0.56189900

H	0.64282400	-1.80521500	-1.03711400
C	3.75287700	-0.73286800	0.10772400
H	3.96979200	1.06148000	1.31404500
H	3.27627000	1.33919600	-0.27745500
H	3.07335700	-2.36438400	-1.15784400
H	2.74125000	-0.76095600	-1.80109800
H	4.74360500	-0.65930200	-0.35182300
H	3.86099200	-1.39373700	0.97868800
O	0.74989100	0.72923700	-1.01780300
O	0.36617200	2.09586800	-0.72064000
H	-0.59492500	2.02848700	-0.86388600
C	-4.03176900	-1.54782900	-0.19707900
H	-4.98387500	-1.64340600	0.33011000
H	-4.19861700	-1.01073200	-1.13376000
H	-3.69719500	-2.56371500	-0.45148600

P10

C	1.74231700	-0.32635700	0.39080000
C	2.86278700	-1.31389100	0.37115200
C	0.58557700	-0.66439100	-0.57700300
H	0.84439200	-0.26590100	-1.56282000
H	1.36636700	-0.19365700	1.40645600
H	0.52374000	-1.75017400	-0.68943400
C	-0.80857200	-0.14301400	-0.15371600
C	-1.78241900	-0.20620200	-1.34500100
C	-1.37176700	-0.93335300	1.04580200
C	-3.20746800	0.21571400	-0.96504400
H	-1.78606000	-1.23386400	-1.73008400
H	-1.39593800	0.43628000	-2.13879900
C	-2.80080700	-0.51471300	1.42150300
H	-1.36093100	-1.99533600	0.77304200
H	-0.70697200	-0.81669400	1.90671800
C	-3.74588200	-0.60089300	0.21650900
H	-3.86409100	0.10822400	-1.83435300
H	-3.20063900	1.27757800	-0.70082400
H	-3.16131200	-1.15110100	2.23631500
H	-2.78449700	0.51104500	1.80058100
H	-4.74468600	-0.25068300	0.49586500
H	-3.85648300	-1.65137000	-0.08569100
H	2.97300200	-1.97483900	1.22409000
O	-0.72204600	1.19941200	0.36546400
O	-0.26217200	2.10317500	-0.67100000
H	0.69684200	2.07355200	-0.49533700
O	2.20590400	0.98439300	-0.05880900
O	3.02482400	1.56536200	0.98211900

H	3.90845300	1.27678300	0.71091900
C	3.67631600	-1.56310400	-0.85391700
H	3.73385000	-0.67210400	-1.48520100
H	4.69377200	-1.87885600	-0.60468900
H	3.24862900	-2.36639200	-1.47545200

R11

C	0.57221800	0.15048700	0.99770900
C	1.80066000	-0.18019600	0.13656700
O	2.79715900	0.91717100	0.24434800
O	2.40438600	2.03204300	-0.32851100
O	-0.68263300	0.82425400	-0.98989300
O	-0.47383500	2.22800800	-0.69481100
H	1.52943500	-0.19666000	-0.92113000
H	0.49913600	2.28159800	-0.75216500
H	0.67841200	1.16838100	1.37785100
H	0.53347100	-0.50774300	1.87027900
C	-0.77196500	0.06873800	0.23982300
C	-1.08283300	-1.35654500	-0.25866200
C	-1.91592600	0.59581500	1.12453400
C	-2.46133500	-1.47260100	-0.92665500
H	-1.03663000	-2.02413100	0.61006900
H	-0.30291300	-1.68192900	-0.95418200
C	-3.28663900	0.48444300	0.44519300
H	-1.91546700	0.02013600	2.05853900
H	-1.70272200	1.63458800	1.38376600
C	-3.57649500	-0.95058400	-0.01230100
H	-2.64489500	-2.51763600	-1.19631500
H	-2.45521300	-0.89880300	-1.85782600
H	-4.06356700	0.82952300	1.13460700
H	-3.30829200	1.15510100	-0.41927000
H	-4.53929600	-0.99666400	-0.53074100
H	-3.66356800	-1.60359200	0.86678600
C	2.56003600	-1.43737400	0.53945700
H	2.93306300	-1.30290400	1.56108600
H	1.83149500	-2.25313800	0.58304100
C	3.70685600	-1.80471600	-0.40574100
H	4.45021600	-1.00656500	-0.45245200
H	4.20987300	-2.71439800	-0.06964200
H	3.33939100	-1.98176800	-1.42091100

TS11

C	1.83762700	-0.34409000	-0.11499400
C	0.60415100	-0.14882800	0.78444600
H	1.10375600	1.05485700	1.13113400

H	0.61072600	-0.71631800	1.71553000
O	2.23426000	1.01724800	-0.44679600
O	2.08847700	1.71771600	0.78820100
H	1.58049000	-0.78448100	-1.08258200
C	2.96800100	-1.11176100	0.56554000
H	2.58632100	-2.10129100	0.84276600
H	3.22166700	-0.59521300	1.49602800
C	-0.77153700	-0.06196400	0.14844000
C	-1.30991300	-1.49493600	-0.11717500
C	-1.75736400	0.72953000	1.03120000
C	-2.74178700	-1.49107500	-0.67363900
H	-1.28209500	-2.04052500	0.83241800
H	-0.63351700	-2.00932300	-0.80619300
C	-3.18270700	0.72352400	0.46584400
H	-1.74804300	0.28261200	2.03295000
H	-1.38874800	1.75188100	1.12622200
C	-3.69665900	-0.70235000	0.23043700
H	-3.08262200	-2.52598600	-0.78155500
H	-2.73408300	-1.04913400	-1.67362500
H	-3.84519300	1.25868200	1.15319400
H	-3.19054200	1.27693200	-0.47796400
H	-4.69643500	-0.67596500	-0.21374000
H	-3.79678000	-1.21879400	1.19470300
O	-0.69169600	0.49130200	-1.18024600
O	-0.36031700	1.90108100	-1.12111200
H	0.61268400	1.85723000	-1.17079700
C	4.20754600	-1.25189100	-0.32018500
H	4.58551200	-0.26932600	-0.60953100
H	5.00465400	-1.78238200	0.20606400
H	3.98230700	-1.80918800	-1.23487400

P11

C	1.83849700	-0.20406800	0.08910700
H	1.64702300	-0.19629400	-0.98893800
C	0.56991700	0.00026300	0.84548400
H	0.63904400	0.08322700	1.92676900
O	-0.72396100	0.69829400	-1.08087700
O	-0.47043300	2.10976100	-0.87261600
H	0.50438100	2.12521900	-0.86646300
C	-0.78438100	0.00829500	0.19616300
C	-1.21694500	-1.42089300	-0.23061800
C	-1.84826800	0.63902100	1.11491100
C	-2.63446400	-1.46560800	-0.82156600
H	-1.16186600	-2.05720200	0.65970600
H	-0.49026300	-1.81044500	-0.94963600

C	-3.25537500	0.59414900	0.50855900
H	-1.83322700	0.09117400	2.06522900
H	-1.55512100	1.66860800	1.32717700
C	-3.66197400	-0.83459900	0.12605100
H	-2.90000600	-2.50602200	-1.03640700
H	-2.63987300	-0.92988500	-1.77481200
H	-3.97123600	1.01617400	1.22114900
H	-3.27852700	1.23197100	-0.38036500
H	-4.65225300	-0.83510900	-0.34017400
H	-3.74423100	-1.44629400	1.03483300
O	2.80782300	0.83790600	0.37898500
O	2.33224600	2.09509000	-0.18428300
H	2.22986300	2.61785800	0.62316800
C	2.58771900	-1.49374700	0.47314000
H	2.86580500	-1.42694300	1.53039200
H	1.87418500	-2.31886600	0.38934200
C	3.82172100	-1.77219500	-0.39058100
H	4.55017700	-0.96487800	-0.30701200
H	4.30392000	-2.70346100	-0.08235000
H	3.54811800	-1.87094000	-1.44518300

R12

C	-1.00016600	-0.25336100	1.25086700
C	-2.11803700	-1.13124100	0.68529800
O	-0.15900900	0.71528200	-0.85579000
O	-0.74612500	1.86150200	-0.59552100
O	-2.62004400	-0.70976500	-0.57755200
O	-3.30247000	0.55189000	-0.38431900
H	-2.94003600	-1.18143700	1.40787700
H	-1.77771400	-2.15113500	0.48506700
H	-2.64105700	1.17938100	-0.72711000
H	-1.40605800	0.73203700	1.48823100
H	-0.67410300	-0.69446100	2.19893700
C	0.24539400	-0.05085300	0.37593500
C	1.30098300	0.78515100	1.11503900
C	0.82859100	-1.34151900	-0.20907800
C	2.59084900	0.99130700	0.31025700
H	1.53149000	0.26407500	2.05143900
H	0.85060800	1.74394600	1.38429400
C	2.13200000	-1.12094900	-0.99320600
H	1.01122900	-2.02505800	0.62881300
H	0.08121500	-1.81234800	-0.85175500
C	3.16948800	-0.34341800	-0.17513500
H	3.31911700	1.52474600	0.92836900

H	2.38243900	1.63349800	-0.55196700
H	2.53314500	-2.09120900	-1.30104800
H	1.90722800	-0.56952600	-1.91188900
H	4.06871600	-0.17096100	-0.77408900
H	3.48035900	-0.94352200	0.69042300

TS12

O	0.88146600	1.83865600	0.67517300
H	1.29205200	1.40248400	-0.38947800
O	0.12045100	0.67220300	0.97982400
C	1.00529800	0.33037100	-1.17098500
H	0.83935800	0.70439300	-2.18192800
C	2.13804800	-0.66122600	-1.10077400
H	1.84455000	-1.60973300	-1.57352200
H	3.01129500	-0.27823700	-1.64088200
O	2.51909100	-1.05362900	0.21112600
O	3.23332000	0.05078300	0.81775600
H	2.51745600	0.51545200	1.28372400
C	-0.27934600	0.14842500	-0.32199300
C	-1.42873600	0.98812000	-0.90426000
C	-0.70248200	-1.30450900	-0.07931300
C	-2.70161900	0.88633400	-0.05415400
H	-1.63154500	0.63248100	-1.92205100
H	-1.09933700	2.02726700	-0.98481300
C	-1.99172500	-1.40273700	0.75408800
H	-0.86303500	-1.77266200	-1.05858700
H	0.11602100	-1.83469300	0.41037500
C	-3.12970800	-0.57303400	0.14665000
H	-3.50403200	1.46085600	-0.52665900
H	-2.51136400	1.34820500	0.92018200
H	-2.28511100	-2.45341400	0.83936600
H	-1.78397300	-1.04981400	1.76947800
H	-4.01453800	-0.62258100	0.78859700
H	-3.42081800	-1.00457400	-0.82038800

P12

C	-0.93779400	-0.37059400	1.37015400
H	-0.73187900	-0.88513900	2.30270300
C	-2.31855000	0.16942000	1.18469600
H	-3.01245400	-0.34871000	1.85337900
H	-2.39551700	1.24479700	1.39328300
O	-2.87233600	-0.03719400	-0.13341700
O	-2.54364200	1.10621400	-0.96059700
H	-1.66939500	0.82607100	-1.29931800
C	0.21439700	-0.16834400	0.42885500
C	0.73671500	1.29200100	0.46219000

C	1.35073800	-1.16475600	0.72203300
C	1.98154200	1.50567700	-0.41347000
H	0.97470300	1.52288900	1.50638400
H	-0.07067200	1.96606700	0.16353100
C	2.58101400	-0.93917800	-0.16454600
H	1.62642900	-1.05422600	1.77792200
H	0.96132900	-2.17656600	0.59283700
C	3.09148600	0.50424900	-0.07098300
H	2.33741600	2.53247200	-0.28130900
H	1.70610900	1.39804200	-1.46679100
H	3.36855300	-1.64176500	0.12498800
H	2.31862100	-1.16804900	-1.20204900
H	3.94568000	0.64808800	-0.73968500
H	3.45541800	0.69761900	0.94709000
O	-0.21688300	-0.31718000	-0.96293200
O	-0.77961500	-1.63586400	-1.17372500
H	-1.71182900	-1.46025100	-0.94117700

R13

C	-0.87635900	-0.44122400	0.91683000
C	-2.00700300	-0.92204000	-0.00322300
O	0.29477100	1.09952100	-0.62074400
O	-0.19096200	2.14970200	0.00238100
O	-2.30536900	0.01409800	-1.05032300
O	-2.84830200	1.22152400	-0.46157600
H	-1.67295800	-1.78289900	-0.59169200
H	-2.07873500	1.81634900	-0.51091200
H	-1.21705800	0.43928900	1.46669300
H	-0.69082900	-1.22177600	1.66149600
C	0.47448300	-0.10054700	0.26854200
C	1.51058700	0.26968800	1.34149900
C	1.00063000	-1.16111500	-0.70511800
C	2.90001400	0.58073300	0.77006800
H	1.57967000	-0.57791800	2.03292800
H	1.12391200	1.11808000	1.91175500
C	2.40282500	-0.84680400	-1.25052300
H	1.01724000	-2.11701400	-0.16793700
H	0.29492300	-1.26839100	-1.53181900
C	3.40475400	-0.55558100	-0.12757600
H	3.59650100	0.75843600	1.59492300
H	2.85598200	1.51001100	0.19232600
H	2.74473200	-1.68644200	-1.86312900
H	2.34116100	0.02078000	-1.91540000
H	4.38071700	-0.29945200	-0.55070700

H	3.55329700	-1.46033600	0.47684000
C	-3.26073300	-1.30851400	0.77816000
H	-4.03702300	-1.64519500	0.08847600
H	-3.04442600	-2.11761500	1.48124400
H	-3.64340400	-0.45223800	1.33506200

TS13

O	0.41721900	1.78641200	1.12896400
H	1.03133200	1.48542800	0.10993000
O	-0.27870500	0.54324400	1.18415500
C	0.89087400	0.53689600	-0.85071900
H	0.83433700	1.07451300	-1.79788900
C	2.04752100	-0.44156200	-0.82738500
H	1.82494200	-1.23750500	-1.55587800
O	2.14139000	-1.20799600	0.37867700
O	2.49638200	-0.33246800	1.47808000
H	1.61343100	-0.10924700	1.81999400
C	-0.48816800	0.20074100	-0.22406600
C	-1.57827600	1.10550300	-0.82501400
C	-0.90550400	-1.27387100	-0.26068700
C	-2.94645500	0.87367700	-0.17139200
H	-1.63878800	0.89374100	-1.89966000
H	-1.27079400	2.14815100	-0.71723500
C	-2.28619900	-1.50386900	0.37772800
H	-0.93533100	-1.57932600	-1.31413200
H	-0.14272500	-1.87782300	0.23084000
C	-3.36125500	-0.60136500	-0.23963900
H	-3.69502500	1.50657400	-0.65762200
H	-2.89439400	1.19087300	0.87526400
H	-2.55971100	-2.55767500	0.26808800
H	-2.21741500	-1.30687000	1.45267100
H	-4.31703700	-0.75007900	0.27216800
H	-3.52018900	-0.88769700	-1.28789500
C	3.38293100	0.20577300	-1.19629800
H	4.16013200	-0.55886100	-1.23932200
H	3.32045100	0.69825400	-2.17018100
H	3.66367800	0.94247600	-0.44333100

P13

C	0.86930100	-1.07392300	-0.27568700
H	0.88659300	-2.14493900	-0.10088100
C	2.16060200	-0.40765000	-0.63735700
H	2.09266400	0.11573300	-1.59716800
O	2.51220400	0.63392700	0.33401400
O	2.04386300	1.91539600	-0.14777500
H	1.12776400	1.90327500	0.19578500

C	-0.43082300	-0.35783600	-0.04345700
C	-0.96987400	0.31808900	-1.32887300
C	-1.48191100	-1.30327100	0.56285300
C	-2.34516300	0.97459300	-1.13020500
H	-1.03771000	-0.46093300	-2.09632800
H	-0.24028800	1.05113700	-1.68350800
C	-2.84557000	-0.63105600	0.75887400
H	-1.58302400	-2.16252600	-0.11145100
H	-1.09722800	-1.68001700	1.51303600
C	-3.36398900	-0.01120500	-0.54499400
H	-2.69644000	1.36295700	-2.09153600
H	-2.24229300	1.83270700	-0.45955700
H	-3.55974500	-1.36624800	1.14249200
H	-2.75176800	0.14746600	1.52228700
H	-4.31845500	0.49508200	-0.37093000
H	-3.56106400	-0.80754500	-1.27525500
O	-0.23310400	0.78766800	0.85633400
O	0.33037200	0.33966300	2.11398700
H	1.28100300	0.36551700	1.89014900
C	3.33815900	-1.37590000	-0.65381100
H	3.15223600	-2.18136400	-1.36883000
H	4.25198600	-0.85364800	-0.93849200
H	3.48470100	-1.82093400	0.33366400

R14

C	-0.57374700	-0.20470300	0.94049300
C	-1.76705000	-0.56256900	0.04498400
O	0.76573000	1.13156700	-0.65132200
O	0.42199500	2.25198400	-0.05700500
O	-1.95719700	0.38066100	-1.02171000
O	-2.33793800	1.66020400	-0.45706000
H	-1.55287400	-1.47249400	-0.52708200
H	-1.49907700	2.15076800	-0.52353000
H	-0.79290100	0.72638300	1.46841700
H	-0.47889800	-0.98189900	1.70537300
C	0.80216300	-0.05556600	0.27269300
C	1.88717200	0.21198400	1.32741600
C	1.18163400	-1.20209200	-0.67128200
C	3.29963100	0.32793100	0.73974800
H	1.85509200	-0.61694100	2.04372900
H	1.61622300	1.11853000	1.87450300
C	2.60726400	-1.08384400	-1.23357400
H	1.08248800	-2.13574900	-0.10466400
H	0.46093100	-1.24522200	-1.49097800

C	3.64862000	-0.88883900	-0.12587400
H	4.02008600	0.43985100	1.55554500
H	3.36859900	1.23819200	0.13467300
H	2.83452000	-1.97784000	-1.82211900
H	2.64957000	-0.23567500	-1.92460700
H	4.64497400	-0.77055500	-0.56227700
H	3.68771100	-1.78714100	0.50456800
C	-3.05046600	-0.77961700	0.85535300
H	-2.85009900	-1.54811400	1.61081000
H	-3.27837600	0.14407300	1.39386900
C	-4.24491200	-1.18927100	-0.00867900
H	-5.14169100	-1.31583100	0.60308400
H	-4.45153500	-0.43022400	-0.76465500
H	-4.05731100	-2.13592400	-0.52555300

TS14

O	-0.13392000	2.12459700	0.63304900
H	0.58109400	1.59512100	-0.21419200
O	-0.75601700	0.89629500	1.00408400
C	0.58156600	0.40284400	-0.86044000
H	0.57389800	0.63782300	-1.92530900
C	1.79020500	-0.42529600	-0.47649400
H	1.69133000	-1.41014300	-0.96240400
O	1.81613100	-0.81483100	0.90215800
O	2.01529500	0.35831900	1.73076200
H	1.09235900	0.59940000	1.92285600
C	-0.82385100	0.14894200	-0.25357300
C	-1.90962100	0.75304900	-1.16155600
C	-1.15014900	-1.30320300	0.11359200
C	-3.31282100	0.60851000	-0.55913200
H	-1.86549400	0.23837900	-2.12929200
H	-1.67391500	1.80416000	-1.34276400
C	-2.56486000	-1.45195200	0.69957300
H	-1.07154100	-1.89789300	-0.80515000
H	-0.39857000	-1.67867500	0.80796200
C	-3.63384700	-0.85293900	-0.22226500
H	-4.05260100	1.01403900	-1.25599900
H	-3.36887100	1.21444000	0.35110600
H	-2.76595400	-2.51173600	0.88335700
H	-2.60040900	-0.95166100	1.67299400
H	-4.61967200	-0.92571800	0.24726200
H	-3.68550000	-1.43847400	-1.14992700
C	3.11379400	0.21085200	-0.92628500
H	3.02991100	0.46103100	-1.98970900
H	3.23705000	1.15082700	-0.38316100

C	4.32173200	-0.69654200	-0.68783400
H	5.24583600	-0.19697200	-0.98849200
H	4.40371500	-0.95879800	0.36803200
H	4.24254400	-1.62578300	-1.26111000

P14

C	-0.58073800	-0.81091200	-0.60657900
H	-0.63683700	-1.44507200	-1.48565100
C	-1.85964100	-0.46325400	0.08745500
H	-1.82739500	-0.71609700	1.15320300
O	-2.09062500	0.98729100	0.05417200
O	-1.64457900	1.57691700	1.29820700
H	-0.69647000	1.70817000	1.09540200
C	0.76481700	-0.25804500	-0.23101100
C	1.20945500	-0.71397700	1.18028600
C	1.82743800	-0.61249000	-1.28494200
C	2.62420000	-0.24010500	1.54934700
H	1.17099300	-1.80891100	1.19028000
H	0.48156400	-0.36369200	1.91705900
C	3.23030500	-0.12598400	-0.90356000
H	1.82910300	-1.70341000	-1.39999000
H	1.51795600	-0.18604400	-2.24145900
C	3.65026000	-0.63661300	0.48054500
H	2.89926400	-0.66362800	2.52055900
H	2.62233300	0.84766600	1.66650300
H	3.94599100	-0.45315000	-1.66413500
H	3.24104000	0.96831500	-0.90861100
H	4.63766200	-0.24592800	0.74507800
H	3.74392800	-1.73056100	0.45317700
O	0.69397600	1.20288400	-0.08372500
O	0.23854500	1.80440200	-1.32125100
H	-0.72816500	1.74655600	-1.19060700
C	-3.08461100	-1.11031600	-0.56291400
H	-2.87943100	-2.18199500	-0.66151200
H	-3.18209000	-0.71781300	-1.58142100
C	-4.37961100	-0.88516300	0.21803900
H	-4.57379100	0.18119400	0.34306900
H	-5.23064300	-1.32997800	-0.30350600
H	-4.32479100	-1.33641500	1.21334800

R15

C	-2.10605800	-1.70889700	0.79513700
C	-0.70009900	-1.12175700	0.98390400
C	-0.14603100	-0.57118300	-0.34514500
C	-1.10488400	0.48849500	-0.94160100

C	-2.51286600	-0.09698300	-1.11683300
C	-3.06950400	-0.67977800	0.18990000
H	-0.74034600	-0.31256100	1.71789700
H	-0.02289100	-1.87948800	1.38887100
H	-2.04946400	-2.58759100	0.13877700
H	-2.48821000	-2.06386000	1.75713200
H	-0.72556600	0.83842100	-1.90771100
H	-3.16671000	0.68878500	-1.50256500
H	-2.46073100	-0.88148400	-1.88129700
H	-3.22977900	0.13420000	0.90398800
H	-4.04693600	-1.13576900	0.00485000
H	-0.15797500	-1.39841200	-1.06743800
C	1.33937100	-0.12655300	-0.27036600
H	1.49593500	0.81337300	-0.80089600
O	-1.27656500	1.63996600	-0.10538700
O	-0.19109900	2.57018700	-0.35181500
H	0.38464000	2.37516700	0.41192700
O	1.75151700	0.14017700	1.13117900
O	1.42523500	1.32860700	1.58427500
C	2.32040800	-1.19180900	-0.75334000
H	2.02756600	-1.45146200	-1.77578600
H	2.18293500	-2.09772700	-0.15229600
C	3.78621500	-0.75072700	-0.72893300
H	4.11081800	-0.50860900	0.28458800
H	4.43363500	-1.54406100	-1.10969300
H	3.94107300	0.13474000	-1.35269900

TS15

C	-1.33021800	0.08690500	-0.28591200
C	-2.53295300	-0.83821700	-0.02700200
H	-2.72202400	-0.15131700	1.12317100
H	-2.29932500	-1.86707900	0.24392000
O	-1.74210300	1.32502800	0.33621600
O	-2.28590300	0.90151600	1.58373600
H	-1.21170700	0.34266900	-1.34302700
C	-0.00730900	-0.45323700	0.28261800
C	0.52567200	-1.65094900	-0.52871500
C	1.05291300	0.65892000	0.39292200
H	-0.19887500	-0.78027600	1.31162700
C	1.85275500	-2.17387700	0.04183300
H	0.67474700	-1.34141400	-1.56899500
H	-0.21478600	-2.45730500	-0.54185900
C	2.38386400	0.13665700	0.93725200
H	0.66273800	1.46136400	1.02529000
C	2.90484300	-1.06085500	0.13243600

H	2.22235000	-2.99655900	-0.57832000
H	1.67583400	-2.59166700	1.04169600
H	3.10627300	0.95582800	0.93103800
H	2.23273100	-0.14933300	1.98571100
H	3.82425900	-1.44370800	0.58578200
H	3.16509700	-0.72441800	-0.87635400
O	1.19618500	1.17582000	-0.94438500
O	1.99145500	2.38481900	-0.88072000
H	1.29384200	3.05547400	-0.90636500
C	-3.74014600	-0.66308500	-0.90508500
H	-4.61289200	-1.18233400	-0.50186800
H	-3.98344000	0.39518700	-1.02647900
H	-3.54842400	-1.07615300	-1.90581300

P15

C	-1.35084500	-0.27606500	-0.02323700
C	-2.31974500	-1.39080500	0.14302400
H	-2.07366300	-2.19008400	0.83519400
H	-1.36359000	0.11363000	-1.04569000
C	0.08563200	-0.63820600	0.38732700
C	0.69293000	-1.70609200	-0.54484100
C	0.99227500	0.60046500	0.46768100
H	0.04086100	-1.05011000	1.40503300
C	2.13071000	-2.06089600	-0.13450000
H	0.69203800	-1.32182400	-1.57142000
H	0.06718700	-2.60391000	-0.54398300
C	2.43064600	0.25215900	0.85630500
H	0.56533400	1.30780100	1.18031800
C	3.02431300	-0.81432600	-0.07305800
H	2.54526500	-2.79058200	-0.83728500
H	2.11620500	-2.54891500	0.84917100
H	3.02521200	1.16932800	0.83084900
H	2.43602400	-0.10689500	1.89370000
H	4.03184400	-1.08432100	0.25801200
H	3.12218000	-0.38914500	-1.07730300
O	0.95484400	1.21442800	-0.83802200
O	1.06719200	2.64593400	-0.67491400
H	0.13397900	2.85569100	-0.49785700
O	-1.88278300	0.81047600	0.82213800
O	-1.68410800	2.10378600	0.16767600
H	-2.59935300	2.34306800	-0.02818200
C	-3.70661500	-1.30826700	-0.39197700
H	-4.11419700	-2.30021300	-0.60879500
H	-4.39072400	-0.83808300	0.33068900
H	-3.75156300	-0.71023400	-1.30781600

R16

C	-2.23438400	-1.85647400	0.91641700
C	-0.97104400	-1.00940300	1.12538300
C	-0.39066000	-0.53701800	-0.22236500
C	-1.45069800	0.25282900	-1.02880400
C	-2.71698800	-0.59201600	-1.22540500
C	-3.29282800	-1.09875900	0.10449800
H	-1.21638000	-0.13625400	1.73576200
H	-0.21920100	-1.57873600	1.67977300
H	-1.96856000	-2.78537600	0.39430800
H	-2.64339600	-2.15202600	1.88741600
H	-1.04339400	0.54352500	-2.00311000
H	-3.45239200	0.01040500	-1.76394700
H	-2.46011100	-1.44296200	-1.86764900
H	-3.65577500	-0.24456000	0.68484300
H	-4.15752000	-1.74135600	-0.08769100
H	-0.19451500	-1.43577300	-0.82210500
C	0.98593200	0.17092900	-0.10016700
H	1.03343200	1.05082400	-0.74271400
O	-1.89335600	1.44893600	-0.37454500
O	-0.96086700	2.52108300	-0.66635900
H	-0.43659200	2.52709400	0.15699800
O	1.20917700	0.67857100	1.27784700
O	0.64436700	1.83529200	1.53672300
C	2.17281900	-0.75345300	-0.35550400
H	2.02553000	-1.19721700	-1.34655100
H	2.13939400	-1.58222700	0.36220000
C	3.54039800	-0.06227500	-0.30242500
H	3.67910400	0.39182300	0.68268900
H	3.55347800	0.76091300	-1.02674600
C	4.69650600	-1.02216800	-0.59526800
H	5.65829800	-0.50499800	-0.55490200
H	4.72815300	-1.83774300	0.13360600
H	4.60156700	-1.46956900	-1.58967400

TS16

C	0.93454700	-0.29756500	-0.24717000
C	2.23040400	0.47605300	0.04955500
H	2.32182600	-0.24975200	1.18806500
H	2.11631000	1.52006000	0.34290100
O	1.18728400	-1.58649500	0.35849500
O	1.75883400	-1.25260600	1.62100300
H	0.80795100	-0.52060400	-1.31073200
C	-0.32876300	0.38245300	0.30697900

C	-0.70046900	1.64829700	-0.49021100
C	-1.51344900	-0.60007100	0.37393800
H	-0.12290700	0.66634300	1.34600900
C	-1.96997400	2.31280300	0.06402500
H	-0.86143000	1.37682600	-1.53930000
H	0.12907700	2.36253200	-0.47213900
C	-2.78618800	0.06464900	0.90230600
H	-1.23327900	-1.45372700	0.99748300
C	-3.14623300	1.32881400	0.11162400
H	-2.22755900	3.18370000	-0.54684800
H	-1.76775500	2.68952100	1.07536000
H	-3.59882500	-0.66420800	0.86526400
H	-2.62586900	0.31239200	1.95910100
H	-4.02452100	1.80838100	0.55443300
H	-3.42183100	1.04298400	-0.90862800
O	-1.68686700	-1.07288700	-0.97607500
O	-2.61798100	-2.18227700	-0.95223900
H	-2.00226000	-2.92876600	-0.97915400
C	3.42920100	0.18438700	-0.81261500
H	3.49438100	-0.89630600	-0.97694900
H	3.25168700	0.63036700	-1.80464400
C	4.74717200	0.71873900	-0.24328100
H	5.57912900	0.50942500	-0.91988800
H	4.70521000	1.80143100	-0.09013900
H	4.97302900	0.25215400	0.71943600

P16

C	-1.00498300	0.23372100	0.06833300
C	-2.24524200	-0.54814200	0.31316500
H	-2.21289600	-1.35748400	1.03797900
H	-0.95042200	0.57965100	-0.96812000
C	0.28413100	-0.51426800	0.44411100
C	0.51478500	-1.74418100	-0.45775600
C	1.51142000	0.41129300	0.43244000
H	0.17118300	-0.85970800	1.48103200
C	1.80746300	-2.48415400	-0.08094200
H	0.57562900	-1.41228100	-1.50068600
H	-0.34068700	-2.42340700	-0.39265100
C	2.80693800	-0.32234400	0.78469500
H	1.34087400	1.23572100	1.12637700
C	3.02427000	-1.54938800	-0.11094500
H	1.96075000	-3.32763100	-0.76154500
H	1.69914000	-2.91090500	0.92507900
H	3.63716500	0.38306400	0.69214300
H	2.76070000	-0.62839400	1.83793200

H	3.92539900	-2.08750200	0.19896900
H	3.19425700	-1.20898200	-1.13764000
O	1.58742400	0.96497600	-0.89829900
O	2.11033100	2.30911600	-0.80626600
H	1.28570900	2.78309200	-0.60213900
O	-1.16187800	1.45315100	0.88634400
O	-0.63582500	2.61431400	0.16990200
H	-1.45552900	3.08767100	-0.02362400
C	-3.57648500	-0.09549400	-0.18456300
H	-3.99401800	0.64502300	0.51742600
H	-3.45024100	0.44373900	-1.13200000
C	-4.58929300	-1.23567400	-0.35945700
H	-4.74840700	-1.76597500	0.58394100
H	-5.55698200	-0.85321800	-0.69362200
H	-4.24006300	-1.96314200	-1.09679100

R17

C	0.64197300	0.14896100	0.22522200
C	1.45126400	1.37459000	-0.21344200
C	2.82478700	1.02119900	-0.80460900
C	3.63129800	0.11887200	0.13712800
C	2.84243500	-1.14883300	0.48847000
C	1.46954400	-0.81382300	1.08724400
H	2.68456800	0.51269300	-1.76396300
H	0.86722700	1.95910500	-0.92907000
H	1.58357600	2.00343400	0.67493100
H	4.58794000	-0.14662600	-0.32269900
H	3.40327800	-1.76639500	1.19629200
H	0.88168600	-1.72156600	1.24691200
H	1.59879800	-0.34069700	2.06769900
H	3.37369800	1.94383400	-1.01602700
H	3.86845400	0.66891400	1.05748400
H	2.70601600	-1.75365700	-0.41426700
O	0.36017500	-0.55077000	-1.08390700
O	-0.42599000	-1.59490500	-0.97528400
C	-0.69625200	0.48680600	0.88779200
H	-0.47220200	1.00739900	1.82443300
H	-1.17372400	-0.45106600	1.16806000
C	-1.66700400	1.33026800	0.03366600
H	-1.49718900	1.15206200	-1.03206500
H	-1.48270800	2.39657000	0.20168800
C	-3.15021300	1.05002800	0.29884400
H	-3.36702300	0.94491000	1.36812300
O	-3.66407000	-0.07897100	-0.40465200

O	-3.19940400	-1.29265300	0.24533200
H	-2.45283600	-1.54481300	-0.32330300
H	-3.76224800	1.86588700	-0.09803900

TS17

C	-2.93984200	-1.04447700	0.51499100
C	-1.61894400	-0.65439400	1.19047500
C	-0.68791600	0.11172300	0.23710700
C	-1.39812400	1.31673600	-0.39024700
C	-2.73266100	0.93072900	-1.05107000
C	-3.65085100	0.17741100	-0.08049200
H	-1.81893600	-0.01300400	2.05779800
H	-1.09768000	-1.54107300	1.55887300
H	-2.72817800	-1.76710400	-0.27994000
H	-3.58648500	-1.54959800	1.23873500
H	-1.57895900	2.04666700	0.40828200
H	-0.73566000	1.79151400	-1.11747800
H	-3.22531000	1.83428300	-1.42315600
H	-2.52692600	0.30066200	-1.92252300
H	-3.95768700	0.85219300	0.72979200
H	-4.56722900	-0.12994800	-0.59355400
C	0.66804800	0.42163100	0.91040300
H	1.03691900	-0.81960100	0.58806600
H	0.65185000	0.51443200	1.99699900
O	-0.27090100	-0.73838700	-0.87416400
O	0.67087300	-1.63387200	-0.28446000
C	1.65948200	1.30543100	0.19500500
H	1.49050600	2.36022100	0.45437200
H	1.52141400	1.21182500	-0.88367000
C	3.11846000	0.96178300	0.52546500
H	3.79594000	1.59431500	-0.05802900
H	3.33424700	1.10218000	1.58987400
O	3.43259800	-0.40942500	0.31719400
O	3.29540700	-0.68707300	-1.10303900
H	2.46243800	-1.19735500	-1.09850700

P17

C	2.77168300	1.00748500	0.75618400
C	1.43660500	0.46403200	1.27995700
C	0.60767200	-0.25387600	0.20053900
C	1.46775700	-1.31245900	-0.54172300
C	2.81089200	-0.76551200	-1.04798900
C	3.60351200	-0.08796500	0.07654300
H	1.62296600	-0.25797100	2.08346600
H	0.82950400	1.26417400	1.70907200
H	2.58152500	1.81269800	0.03726000

H	3.33196600	1.45504400	1.58296500
H	1.64155400	-2.12826000	0.16832300
H	0.88139500	-1.72682800	-1.36599200
H	3.39145400	-1.58584600	-1.48219600
H	2.62952600	-0.04658100	-1.85277000
H	3.88950000	-0.83966200	0.82394100
H	4.53484500	0.33131100	-0.31678400
C	-0.64584200	-0.84947400	0.76137000
H	-0.75479500	-0.88804600	1.84062700
C	-1.73729000	-1.39350400	-0.09743900
H	-1.70051600	-2.49588900	-0.13064900
H	-1.61061500	-1.05010800	-1.12646700
C	-3.14673400	-1.01431800	0.38069800
H	-3.89998300	-1.42869200	-0.29962700
H	-3.33545100	-1.39903400	1.38885000
O	-3.34791600	0.38327800	0.53348000
O	-3.24564400	0.98981000	-0.78180800
H	-2.34668600	1.36456600	-0.72889900
O	0.28750500	0.66077500	-0.89826500
O	-0.52586600	1.75854800	-0.37871600
H	-0.04034000	2.51337500	-0.73748800

R18

C	-0.93781600	-0.08791300	0.22952200
C	-1.57848600	-1.42173900	-0.17075600
C	-2.98308500	-1.26963200	-0.77486500
C	-3.90556900	-0.44994800	0.13545000
C	-3.29035800	0.92038200	0.44600500
C	-1.88906000	0.78666000	1.05752700
H	-2.90511300	-0.77881000	-1.75040400
H	-0.91932800	-1.94747800	-0.86640400
H	-1.63275500	-2.03390500	0.73725000
H	-4.88594900	-0.32635100	-0.33436700
H	-3.93091100	1.48243400	1.13213200
H	-1.42513200	1.76794700	1.18776000
H	-1.96129100	0.33322400	2.05303300
H	-3.40605000	-2.26233800	-0.95639900
H	-4.07465000	-0.99609600	1.07301800
H	-3.22855500	1.50802900	-0.47608200
O	-0.74163900	0.59977700	-1.10101700
O	-0.09270200	1.73717700	-1.02692300
C	0.42805700	-0.22770800	0.90765200
H	0.26569600	-0.74665600	1.85801300
H	0.77651900	0.77169400	1.16321300

C	1.50631500	-0.96278500	0.08453700
H	1.34663900	-0.80654600	-0.98656100
H	1.42848100	-2.04103800	0.25511200
C	2.95027500	-0.51993700	0.38013700
H	3.08084800	-0.30793600	1.44813700
O	3.31387700	0.66320300	-0.35169500
O	2.66936500	1.82194800	0.23677200
H	1.90682000	1.94460700	-0.35292500
C	3.97827200	-1.55210100	-0.07478000
H	4.98933600	-1.17765600	0.09178300
H	3.85105000	-2.48225700	0.48390300
H	3.86187600	-1.76939600	-1.13987500

TS18

C	-3.35902900	-0.84956600	0.46648100
C	-2.00983700	-0.63246300	1.16345200
C	-0.99029900	0.05942600	0.24427900
C	-1.55719700	1.35768900	-0.34175900
C	-2.91932300	1.14539800	-1.02453600
C	-3.92493100	0.46397600	-0.08804800
H	-2.14639400	-0.00435300	2.05252900
H	-1.59337300	-1.58406300	1.50207900
H	-3.22053600	-1.56206600	-0.35331600
H	-4.06459800	-1.30571400	1.16735800
H	-1.66488600	2.07341200	0.48244000
H	-0.83930400	1.78290100	-1.04667200
H	-3.30558400	2.11055700	-1.36640600
H	-2.77523000	0.52791500	-1.91710200
H	-4.16397900	1.13913800	0.74448100
H	-4.86427400	0.27833900	-0.61782700
C	0.38505000	0.19206600	0.93670200
H	0.62076000	-1.06725000	0.56756000
H	0.36621200	0.23854800	2.02624300
O	-0.66049500	-0.79095900	-0.89529000
O	0.17185200	-1.80581500	-0.33396600
C	1.47151500	0.99941100	0.27201800
H	1.39426100	2.05297600	0.57447500
H	1.34750100	0.96462200	-0.81247400
C	2.88999400	0.50513000	0.61105300
H	3.02909400	0.50744200	1.69922100
O	3.04158100	-0.89305900	0.32531500
O	2.86388400	-1.10192100	-1.10428000
H	1.98103000	-1.52011700	-1.10517900
C	3.98080200	1.33813900	-0.05263400
H	3.91066100	2.38520500	0.25536800

H	4.96402600	0.95705400	0.22947500
H	3.88954700	1.28426300	-1.13853400

P18

C	3.20857100	0.86230500	0.64009500
C	1.83825900	0.51158200	1.23224700
C	0.90697600	-0.19082700	0.22912000
C	1.62516200	-1.39579500	-0.43527600
C	3.00637000	-1.04555000	-1.00961200
C	3.89876100	-0.36825300	0.03834900
H	1.96828800	-0.16409200	2.08569100
H	1.33437700	1.40426100	1.60887600
H	3.08535400	1.62278500	-0.13947300
H	3.83669000	1.31100200	1.41592400
H	1.72771700	-2.16418500	0.33873200
H	0.97577700	-1.80969600	-1.21104300
H	3.48182300	-1.95855000	-1.38239300
H	2.88270500	-0.38017900	-1.86950700
H	4.12593800	-1.08367000	0.83985800
H	4.85747100	-0.08595300	-0.40787000
C	-0.39167200	-0.58982800	0.85765000
H	-0.48915100	-0.49148700	1.93406400
C	-1.54575900	-1.11621700	0.07296200
H	-1.58599200	-2.21657500	0.13254800
H	-1.42653900	-0.87004500	-0.98544300
C	-2.91146100	-0.58961500	0.55439700
H	-3.03049900	-0.82715900	1.61901600
O	-2.95345500	0.84222500	0.59509200
O	-2.78571600	1.35119100	-0.75641300
H	-1.85286300	1.63299800	-0.71945400
O	0.66388700	0.66501100	-0.93559500
O	-0.00108200	1.88796500	-0.48760200
H	0.53326400	2.54778000	-0.94939800
C	-4.08263500	-1.16516000	-0.23599200
H	-4.09722100	-2.25687200	-0.17021700
H	-5.02488200	-0.77950600	0.15852200
H	-4.00676600	-0.87784700	-1.28592800

R19

C	0.41832000	1.55351300	0.59876800
C	1.87960700	1.95995800	0.35905300
C	2.86004000	0.92382100	0.91779300
C	2.56420200	-0.47515500	0.37541300
C	1.10041300	-0.88157700	0.57239900
C	0.10606700	0.15877900	0.01650200

H	3.89054700	1.19039500	0.67503400
H	2.05265100	2.07046700	-0.71603400
H	2.07035400	2.93757800	0.81203200
H	0.21416900	1.54293400	1.67862200
H	-0.24098500	2.30806600	0.16238800
H	3.21911100	-1.21486700	0.85257900
H	0.93691100	-1.85469400	0.10751400
H	0.93430900	-1.01167200	1.64829900
H	0.23367400	0.20285900	-1.07125100
H	2.78623200	0.88509800	2.01178200
C	-2.41434100	0.58845400	-0.39925800
H	-2.23730300	1.64286600	-0.16787800
H	-2.27963500	0.48036700	-1.48215100
O	2.76954900	-0.54972200	-1.04675400
O	4.17699000	-0.30779200	-1.31521600
H	4.44824400	-1.19113100	-1.60000700
C	-1.34894300	-0.25024700	0.30189500
H	-1.52810100	-0.30880500	1.37913300
O	-1.54755200	-1.63052300	-0.19025700
O	-1.58744200	-2.52498700	0.77672600
C	-3.85102200	0.21771400	-0.01055800
H	-4.01845700	-0.84362900	-0.21478700
H	-3.97573800	0.34688200	1.07113700
C	-4.89687000	1.05559600	-0.75076400
H	-4.81855800	0.91890600	-1.83366400
H	-5.91052400	0.77429800	-0.45417900
H	-4.77410000	2.12313600	-0.54201800

TS19

C	1.15266200	-0.73086500	-0.45655900
C	2.07353100	0.43029300	-0.04164500
H	2.28369500	-0.24605200	1.11394600
H	1.58156900	1.36561400	0.22607400
O	1.75988700	-1.86993700	0.19589100
O	2.05449100	-1.38579400	1.50217900
H	1.23116900	-0.96722100	-1.52593100
C	-0.31659000	-0.52375900	-0.06379800
C	-0.96396900	0.57101000	-0.93084000
C	-1.12398900	-1.82839900	-0.16639300
H	-0.33643900	-0.19298600	0.98007000
C	-2.43563000	0.80175500	-0.57159500
H	-0.91655800	0.28089700	-1.98908200
H	-0.43581000	1.52206400	-0.83758700
C	-2.58729200	-1.61108000	0.24042900
H	-1.08015500	-2.20032300	-1.20029100

H	-0.66271400	-2.59371000	0.46043400
C	-3.24834400	-0.49701100	-0.58394700
H	-2.87694400	1.53168500	-1.26213300
H	-3.15313400	-2.54004900	0.12026300
H	-2.63233500	-1.34956300	1.30214200
H	-4.26040400	-0.29082000	-0.22425500
H	-3.33851500	-0.81212300	-1.63014000
O	-2.57949700	1.31133100	0.76490200
O	-1.91885400	2.60586400	0.82774500
H	-2.68620000	3.17880800	0.95978200
C	3.38322500	0.57903500	-0.76825600
H	3.82644100	-0.41246900	-0.90942200
H	3.17283700	0.96295000	-1.77984600
C	4.37643000	1.51242000	-0.06833700
H	4.64260500	1.12646100	0.91926800
H	5.29699400	1.61131700	-0.64849700
H	3.95548100	2.51342500	0.06511100

P19

C	-1.23543900	-0.47565500	0.06945800
C	-1.89289500	0.81686200	-0.27968700
H	-1.76357300	1.16995900	-1.30063300
H	-1.48090100	-0.76411300	1.09968300
C	0.29876300	-0.47601100	-0.11495600
C	0.94684000	0.63785200	0.72652300
C	0.92426300	-1.84040100	0.22698600
H	0.49697900	-0.25660500	-1.17079100
C	2.47216000	0.66361900	0.58558400
H	0.70869900	0.48786200	1.78782900
H	0.55316800	1.61624700	0.44524000
C	2.44752800	-1.82378500	0.04121200
H	0.68451700	-2.08896600	1.27066600
H	0.47510000	-2.61960700	-0.39000500
C	3.10820100	-0.70346400	0.85608500
H	2.89319800	1.41815300	1.26242800
H	2.87080200	-2.78947400	0.33477600
H	2.68348100	-1.68577300	-1.01857400
H	4.18106000	-0.64814700	0.65050600
H	2.99692500	-0.90731200	1.92745900
O	2.87792500	0.99428800	-0.75415500
O	2.41511700	2.34000800	-1.05061900
H	3.26003200	2.80987300	-1.06348200
O	-1.69046800	-1.54450900	-0.80272200
O	-3.08066400	-1.82452000	-0.50119500
H	-3.52571400	-1.33146700	-1.20472500

C	-2.78605700	1.57961700	0.64191900
H	-2.60503700	2.65520300	0.52432100
H	-2.54420000	1.33176700	1.68175200
C	-4.29214800	1.31171500	0.40994800
H	-4.52465500	0.25800600	0.57650300
H	-4.90199700	1.91149300	1.09179400
H	-4.58045700	1.56867100	-0.61364000

R20

C	1.93739100	-1.55134300	-0.78692500
C	0.44319400	-1.20385500	-0.84713700
C	-0.03930700	-0.59724700	0.48504700
C	0.79715800	0.65158100	0.85696600
C	2.29116700	0.30247200	0.90406700
C	2.78444100	-0.33057300	-0.40482500
H	0.26977900	-0.48392600	-1.65140700
H	-0.14489600	-2.09378300	-1.09064500
H	2.09647800	-2.35160200	-0.05184500
H	2.26164400	-1.94841400	-1.75367000
H	0.47656500	1.04279600	1.82859100
H	2.85018000	1.21333200	1.13103600
H	2.45054700	-0.39310700	1.73672700
H	2.73143800	0.41528100	-1.20423000
H	3.83679500	-0.61361700	-0.30509900
H	0.18646600	-1.32775000	1.27367400
C	-1.57680900	-0.39857400	0.55984100
H	-1.82619400	0.55229500	1.03056400
O	0.68943300	1.71929700	-0.09218300
O	-0.50283600	2.49168900	0.20033600
H	-1.11353300	2.12833700	-0.46887000
O	-2.19330600	-0.34179300	-0.79136200
O	-2.07127900	0.81283800	-1.40386300
C	-2.30421800	-1.55186800	1.23237100
H	-2.00829900	-1.60480400	2.28245000
H	-2.05554100	-2.50408100	0.75705000
H	-3.38562200	-1.41076000	1.18581800

TS20

C	-1.72099600	-0.51082200	-0.44628500
H	-1.07335600	0.60014100	1.07737800
O	-2.20284000	-0.95465900	0.84263400
O	-2.08749600	0.20416400	1.65533900
H	-1.48299300	-1.43341200	-0.98438500
C	-2.77463900	0.29275800	-1.19498600
H	-2.41125200	0.60035900	-2.17864400

H	-3.06152100	1.17998900	-0.62863600
C	-0.43052400	0.26194800	-0.06806600
C	0.70442000	-0.63831200	0.39898800
C	0.00627600	1.46827300	-0.86253500
C	1.84405500	0.13736800	1.06001100
H	0.30896900	-1.40622700	1.07127200
C	1.15353200	2.23352500	-0.17651300
H	0.35032600	1.12371100	-1.85056200
H	-0.83914700	2.13604200	-1.04503100
C	2.31850700	1.30616700	0.18869700
H	2.66041800	-0.55769000	1.26728900
H	1.48320800	0.50747700	2.02720100
H	1.49515800	3.03847500	-0.83376000
H	0.76828500	2.71263900	0.73211000
H	3.09844800	1.86786900	0.71117400
H	2.77163400	0.90928500	-0.72554200
H	-3.66267000	-0.32624900	-1.33690900
O	1.14498100	-1.29559800	-0.81633300
O	2.07902300	-2.34523200	-0.44832400
H	1.52136100	-3.12724200	-0.56536000

P20

C	1.33365300	-1.16273000	0.22423300
H	1.43613600	-2.09143500	-0.34581100
C	1.95057300	-1.34011800	1.61167000
H	1.43038600	-2.13740600	2.14653600
H	1.87933400	-0.42458200	2.20236500
C	-0.10805600	-0.72748800	0.21801100
C	-0.43751900	0.65705400	0.68660900
C	-1.08034300	-1.47269800	-0.64820300
C	-1.92855700	0.91452700	0.90783300
H	0.12092000	0.92350700	1.58493200
C	-2.55602000	-1.17376200	-0.34211000
H	-0.87136100	-1.20942400	-1.70060500
H	-0.88430000	-2.54980200	-0.58599200
C	-2.80194200	0.33270300	-0.20808300
H	-2.07455200	1.99242800	1.01068700
H	-2.21070800	0.45483600	1.86315800
H	-3.18584400	-1.60162400	-1.12731500
H	-2.84025800	-1.66791900	0.59488300
H	-3.85685800	0.52952600	0.00411700
H	-2.57210600	0.83544000	-1.15477900
H	3.00428000	-1.61073100	1.51865700
O	0.04527100	1.61625200	-0.34268400
O	1.16046100	2.36786500	0.19255800

H	1.88261400	1.72113200	0.07167100
O	2.19277400	-0.17938600	-0.42434500
O	1.78032300	-0.04930100	-1.80866500
H	1.08095200	0.62768400	-1.71717900

R21

C	-2.10605800	-1.70889700	0.79513700
C	-0.70009900	-1.12175700	0.98390400
C	-0.14603100	-0.57118300	-0.34514500
C	-1.10488400	0.48849500	-0.94160100
C	-2.51286600	-0.09698300	-1.11683300
C	-3.06950400	-0.67977800	0.18990000
H	-0.74034600	-0.31256100	1.71789700
H	-0.02289100	-1.87948800	1.38887100
H	-2.04946400	-2.58759100	0.13877700
H	-2.48821000	-2.06386000	1.75713200
H	-0.72556600	0.83842100	-1.90771100
H	-3.16671000	0.68878500	-1.50256500
H	-2.46073100	-0.88148400	-1.88129700
H	-3.22977900	0.13420000	0.90398800
H	-4.04693600	-1.13576900	0.00485000
H	-0.15797500	-1.39841200	-1.06743800
C	1.33937100	-0.12655300	-0.27036600
H	1.49593500	0.81337300	-0.80089600
O	-1.27656500	1.63996600	-0.10538700
O	-0.19109900	2.57018700	-0.35181500
H	0.38464000	2.37516700	0.41192700
O	1.75151700	0.14017700	1.13117900
O	1.42523500	1.32860700	1.58427500
C	2.32040800	-1.19180900	-0.75334000
H	2.02756600	-1.45146200	-1.77578600
H	2.18293500	-2.09772700	-0.15229600
C	3.78621500	-0.75072700	-0.72893300
H	4.11081800	-0.50860900	0.28458800
H	4.43363500	-1.54406100	-1.10969300
H	3.94107300	0.13474000	-1.35269900

TS21

C	1.45697900	0.36562900	-0.11331600
H	0.52993500	-0.77406300	1.23569100
O	1.79037700	0.69428900	1.25668600
O	1.48284100	-0.49601600	1.96713600
H	1.36279500	1.33632900	-0.61188500
C	2.54517900	-0.47125900	-0.78126600
H	2.21736300	-0.72934600	-1.79371900

H	2.65694800	-1.40833500	-0.22881600
C	0.07548200	-0.32167100	0.04189800
C	-1.04337500	0.64151400	0.41350500
C	-0.34039100	-1.44654400	-0.87502800
C	-2.31393500	-0.07192500	0.87550900
H	-0.68549600	1.34059100	1.17591200
C	-1.62037900	-2.15102200	-0.38722300
H	-0.52641000	-1.02517500	-1.87543000
H	0.47000300	-2.17027500	-0.98971200
C	-2.75325500	-1.15377000	-0.11802200
H	-3.09743900	0.67533100	1.01797600
H	-2.11230800	-0.51765900	1.85700400
H	-1.93000800	-2.89229500	-1.12973500
H	-1.39342900	-2.70434700	0.53251100
H	-3.63374400	-1.67731900	0.26596600
H	-3.05217100	-0.67644600	-1.05690200
O	-1.27035000	1.39217300	-0.80631800
O	-2.16487900	2.49416200	-0.49789300
H	-1.54062500	3.23342800	-0.49599100
C	3.88528500	0.26678200	-0.83911900
H	4.21864000	0.53850700	0.16427200
H	4.65541400	-0.35828100	-1.29742800
H	3.80707900	1.18609400	-1.42822600

P21

C	-1.27794700	-0.67637800	0.22587300
H	-1.51685500	-1.39657000	1.01622600
C	-2.11275000	-0.99805300	-1.02295900
H	-1.71230500	-1.92314200	-1.44971500
H	-1.95320100	-0.21526600	-1.77166500
C	0.21102500	-0.65329100	0.00273500
C	0.79985100	0.48102900	-0.77787800
C	1.08552800	-1.47563700	0.90278100
C	2.25630100	0.27695700	-1.19648400
H	0.19371000	0.72671700	-1.65086600
C	2.52482700	-1.65080700	0.39501700
H	1.11116300	-0.98748800	1.89384000
H	0.61967000	-2.45157500	1.08444700
C	3.11487100	-0.31719100	-0.07529900
H	2.64916900	1.23843600	-1.53571300
H	2.26810000	-0.40186100	-2.05831800
H	3.14044100	-2.08979300	1.18537000
H	2.53147000	-2.36067300	-0.44102300
H	4.14128400	-0.45718800	-0.42675000
H	3.16269600	0.38721400	0.76339300

O	0.73292800	1.70251400	0.07063000
O	-0.19557400	2.64259300	-0.52024200
H	-1.04401200	2.26055400	-0.22411000
O	-1.76766000	0.61724600	0.67850600
O	-1.17501200	0.90625100	1.97016500
H	-0.33484900	1.31698000	1.68394400
C	-3.60904900	-1.15395000	-0.73376600
H	-4.02470000	-0.23883400	-0.31004200
H	-4.15651600	-1.38786900	-1.65030300
H	-3.78865500	-1.96454600	-0.02130500

R22

C	-2.23438400	-1.85647400	0.91641700
C	-0.97104400	-1.00940300	1.12538300
C	-0.39066000	-0.53701800	-0.22236500
C	-1.45069800	0.25282900	-1.02880400
C	-2.71698800	-0.59201600	-1.22540500
C	-3.29282800	-1.09875900	0.10449800
H	-1.21638000	-0.13625400	1.73576200
H	-0.21920100	-1.57873600	1.67977300
H	-1.96856000	-2.78537600	0.39430800
H	-2.64339600	-2.15202600	1.88741600
H	-1.04339400	0.54352500	-2.00311000
H	-3.45239200	0.01040500	-1.76394700
H	-2.46011100	-1.44296200	-1.86764900
H	-3.65577500	-0.24456000	0.68484300
H	-4.15752000	-1.74135600	-0.08769100
H	-0.19451500	-1.43577300	-0.82210500
C	0.98593200	0.17092900	-0.10016700
H	1.03343200	1.05082400	-0.74271400
O	-1.89335600	1.44893600	-0.37454500
O	-0.96086700	2.52108300	-0.66635900
H	-0.43659200	2.52709400	0.15699800
O	1.20917700	0.67857100	1.27784700
O	0.64436700	1.83529200	1.53672300
C	2.17281900	-0.75345300	-0.35550400
H	2.02553000	-1.19721700	-1.34655100
H	2.13939400	-1.58222700	0.36220000
C	3.54039800	-0.06227500	-0.30242500
H	3.67910400	0.39182300	0.68268900
H	3.55347800	0.76091300	-1.02674600
C	4.69650600	-1.02216800	-0.59526800
H	5.65829800	-0.50499800	-0.55490200
H	4.72815300	-1.83774300	0.13360600

H	4.60156700	-1.46956900	-1.58967400

TS22

C	-1.05591000	-0.48078300	0.09037600
H	-0.04791800	0.62669300	1.40879500
O	-1.21688500	-0.91433700	1.46275600
O	-0.89745200	0.24547400	2.21676100
H	-0.96304100	-1.41091100	-0.48032700
C	-2.25473600	0.32660200	-0.39902700
H	-2.05295400	0.67766500	-1.41751400
H	-2.36506400	1.21384000	0.23234900
C	0.29499000	0.27965400	0.14418900
C	1.49901900	-0.63431200	0.32480800
C	0.54388000	1.48438300	-0.73068200
C	2.77252400	0.12571400	0.69573600
H	1.26709500	-1.40126700	1.07049600
C	1.83065300	2.23380800	-0.33664200
H	0.63839900	1.14045100	-1.77268600
H	-0.31230300	2.16262000	-0.70521800
C	3.03865500	1.29276500	-0.26200900
H	3.60653000	-0.57926400	0.70102300
H	2.65759900	0.49577500	1.72165400
H	2.01448200	3.03828900	-1.05475000
H	1.67893500	2.71263600	0.63871100
H	3.92724300	1.84391600	0.05968700
H	3.25555900	0.89484100	-1.25864500
O	1.62907200	-1.29037700	-0.96191800
O	2.61053800	-2.35291300	-0.82932300
H	2.03116800	-3.12769100	-0.81370100
C	-3.55480400	-0.48607700	-0.38429100
H	-3.71922500	-0.86853100	0.62710900
H	-3.43962700	-1.36409800	-1.03191900
C	-4.76903300	0.32852500	-0.83743600
H	-4.64173500	0.70092000	-1.85885600
H	-5.68062500	-0.27422800	-0.81555500
H	-4.92812400	1.19389600	-0.18699400

P22

C	-0.93959800	-0.34087400	0.61273100
H	-1.14107900	-0.88773400	1.54029800
C	-2.00668400	-0.69815700	-0.43186100
H	-1.80885300	-1.72337700	-0.76415400
H	-1.87856600	-0.05619800	-1.31057000
C	0.48062600	-0.59423000	0.18093200
C	1.07162500	0.27613200	-0.88581300
C	1.37896500	-1.37022000	1.09886000

C	2.40623900	-0.22051200	-1.44287000
H	0.36918200	0.44663300	-1.70280400
C	2.68733700	-1.84848500	0.45085200
H	1.62058000	-0.72618000	1.96342700
H	0.82970600	-2.21716500	1.52745400
C	3.35332500	-0.72455400	-0.34983900
H	2.85580700	0.59182900	-2.01885400
H	2.19573000	-1.03843900	-2.14308700
H	3.36140200	-2.22646500	1.22488800
H	2.47483400	-2.68939400	-0.22054200
H	4.28637600	-1.07705900	-0.79903800
H	3.61839000	0.10367400	0.31776300
O	1.29514200	1.62792200	-0.30528800
O	0.37541500	2.56548100	-0.91347700
H	-0.43474400	2.37146200	-0.40345100
O	-1.16433900	1.06955100	0.90384900
O	-0.30807900	1.45530800	2.00855600
H	0.50663300	1.68736700	1.52028200
C	-3.44598000	-0.58831100	0.08837500
H	-3.62070900	0.42787600	0.45059200
H	-3.56307400	-1.24863100	0.95572500
C	-4.48484300	-0.95004800	-0.97627600
H	-4.34796600	-1.97471800	-1.33649900
H	-5.50039600	-0.87082900	-0.57990700
H	-4.41442600	-0.28319300	-1.84118900

R23

C	-0.01423400	1.75542600	-0.05421300
C	-1.53645500	1.82032100	0.13698800
C	-2.27400100	0.85586500	-0.79835500
C	-1.74954600	-0.57463300	-0.65849200
C	-0.22740000	-0.65180200	-0.81028100
C	0.52200700	0.32120400	0.12309300
H	-3.34720600	0.86315700	-0.59815400
H	-1.78081900	1.56660000	1.17329000
H	-1.88773200	2.84310700	-0.02920200
H	0.24435400	2.11491300	-1.06027600
H	0.46991400	2.43199000	0.65478800
H	-2.22947800	-1.22978100	-1.39620200
H	0.10245300	-1.67689400	-0.63347600
H	0.01069000	-0.41147800	-1.85395100
H	0.33686000	0.01205600	1.15857800
H	-2.13933400	1.16479600	-1.84253700
C	2.89174500	1.13075100	0.78130700

H	3.94284600	0.88017700	0.63053600
H	2.75449200	2.18502800	0.53440900
H	2.63896400	0.98135000	1.83432500
O	-2.00790500	-1.11197400	0.65023400
O	-3.44780300	-1.19611200	0.82832800
H	-3.56021100	-2.15644500	0.83305200
C	2.03313100	0.24980500	-0.11247300
H	2.27223300	0.42147000	-1.16652900
O	2.40686300	-1.16060900	0.14272900
O	3.58640600	-1.46026300	-0.35680200

TS23

C	-1.99890300	-0.13715000	0.58056400
H	-0.56835700	0.36231000	-0.69206600
O	-2.01267000	1.27665900	0.27832600
O	-1.30102700	1.36542200	-0.95835200
H	-2.23243400	-0.18358900	1.65102600
C	-3.03733800	-0.90122600	-0.22787500
H	-3.04539600	-1.96024800	0.04302500
H	-2.84154700	-0.81080000	-1.29749900
C	-0.52231400	-0.50738100	0.30100500
C	0.47313900	0.11790800	1.25790800
C	-0.11298500	-1.87296000	-0.19464900
C	1.86233900	0.20462500	0.61226900
H	0.54468900	-0.49437300	2.16923900
H	0.14270800	1.11128700	1.56134100
C	1.31025900	-1.84964900	-0.79321200
H	-0.13692500	-2.57420000	0.65618400
H	-0.81953500	-2.25826000	-0.93316300
C	2.33420800	-1.17121300	0.13100800
H	2.57999600	0.63869900	1.31710100
H	1.62968200	-2.87213600	-1.01356800
H	1.28163000	-1.31434800	-1.74594700
H	3.29283700	-1.06414200	-0.38313900
H	2.51277400	-1.78897800	1.01862100
H	-4.02733000	-0.48893200	-0.02391400
O	1.83290900	1.01353800	-0.57265200
O	1.40053200	2.35271400	-0.22062500
H	0.47883500	2.33332100	-0.54291900

P23

C	-1.58124600	-0.55526900	0.48190100
H	-1.96852200	-0.21698800	1.45176200
C	-2.46853700	-1.66391400	-0.06441500
H	-2.41587800	-2.54907400	0.57472000
H	-2.17156700	-1.94538500	-1.07699900

C	-0.12242700	-0.84773400	0.61086300
C	0.73997800	0.11038600	1.37227800
C	0.60318300	-1.94944900	-0.09738000
C	1.86396500	0.68741400	0.48335100
H	1.23342000	-0.40465600	2.21172500
H	0.15918700	0.93013700	1.79629600
C	1.75937800	-1.39792800	-0.97072100
H	1.04139000	-2.62705900	0.65436100
H	-0.06588900	-2.55873100	-0.70742700
C	2.66330200	-0.43552400	-0.18579400
H	2.51692100	1.32855700	1.08834100
H	2.35156300	-2.23182700	-1.36045800
H	1.33590700	-0.86974600	-1.82813700
H	3.41210600	0.00629200	-0.84891900
H	3.20713000	-0.97611900	0.59791800
H	-3.50548200	-1.32532500	-0.08746300
O	1.34901500	1.44939700	-0.61251200
O	0.50377800	2.51096700	-0.10731500
H	-0.36877200	2.09128400	-0.22852400
O	-1.65006200	0.62524400	-0.39497200
O	-2.97506300	1.23507700	-0.24556900
H	-3.32843200	1.09526300	-1.13475300

R24

C	0.41832000	1.55351300	0.59876800
C	1.87960700	1.95995800	0.35905300
C	2.86004000	0.92382100	0.91779300
C	2.56420200	-0.47515500	0.37541300
C	1.10041300	-0.88157700	0.57239900
C	0.10606700	0.15877900	0.01650200
H	3.89054700	1.19039500	0.67503400
H	2.05265100	2.07046700	-0.71603400
H	2.07035400	2.93757800	0.81203200
H	0.21416900	1.54293400	1.67862200
H	-0.24098500	2.30806600	0.16238800
H	3.21911100	-1.21486700	0.85257900
H	0.93691100	-1.85469400	0.10751400
H	0.93430900	-1.01167200	1.64829900
H	0.23367400	0.20285900	-1.07125100
H	2.78623200	0.88509800	2.01178200
C	-2.41434100	0.58845400	-0.39925800
H	-2.23730300	1.64286600	-0.16787800
H	-2.27963500	0.48036700	-1.48215100
O	2.76954900	-0.54972200	-1.04675400

O	4.17699000	-0.30779200	-1.31521600
H	4.44824400	-1.19113100	-1.60000700
C	-1.34894300	-0.25024700	0.30189500
H	-1.52810100	-0.30880500	1.37913300
O	-1.54755200	-1.63052300	-0.19025700
O	-1.58744200	-2.52498700	0.77672600
C	-3.85102200	0.21771400	-0.01055800
H	-4.01845700	-0.84362900	-0.21478700
H	-3.97573800	0.34688200	1.07113700
C	-4.89687000	1.05559600	-0.75076400
H	-4.81855800	0.91890600	-1.83366400
H	-5.91052400	0.77429800	-0.45417900
H	-4.77410000	2.12313600	-0.54201800

TS24

C	1.21544400	-0.16059400	0.69231900
H	-0.20212800	-0.43517500	-0.66279700
O	1.06254400	-1.55648100	0.33875200
O	0.40907100	-1.51596800	-0.93167500
H	1.39680200	-0.18077200	1.77459500
C	2.38944000	0.49400500	-0.02966900
H	2.42390400	1.55479900	0.24573300
H	2.21151200	0.44823500	-1.10885100
C	-0.18638100	0.40392100	0.35601400
C	-1.29863800	-0.11518600	1.24594800
C	-0.39803200	1.82301000	-0.11383500
C	-2.65518400	-0.00802300	0.53738100
H	-1.33667700	0.47677200	2.17261500
H	-1.11063200	-1.15011400	1.53160400
C	-1.78280300	1.99515700	-0.77503000
H	-0.32708800	2.49350200	0.75879400
H	0.38492400	2.13527200	-0.80818900
C	-2.92717000	1.42873900	0.08063500
H	-3.45437600	-0.36636900	1.19554300
H	-1.96033500	3.05521900	-0.97717600
H	-1.77583700	1.48516600	-1.74195900
H	-3.86598500	1.45800800	-0.47845400
H	-3.06881000	2.04055800	0.97897300
O	-2.67129300	-0.78213400	-0.67102900
O	-2.42586200	-2.17411200	-0.34481100
H	-1.49461800	-2.26174500	-0.62567300
C	3.73195700	-0.16731100	0.30394900
H	3.66644500	-1.23324600	0.06828800
H	3.91023900	-0.10016400	1.38446300
C	4.90695900	0.46222900	-0.44845800

H	5.01159700	1.52542200	-0.20939700
H	5.84910300	-0.02927400	-0.19237300
H	4.77272900	0.37777400	-1.53115500

P24

C	-0.98106300	0.08951100	0.54798200
H	-1.23955500	0.54295600	1.51380700
C	-2.15778500	-0.73700100	0.03019300
H	-2.24161300	-1.62243200	0.67298200
H	-1.92253600	-1.10230900	-0.97504600
C	0.32369700	-0.62934200	0.66865200
C	1.47230200	0.07971000	1.31770400
C	0.63611100	-1.98129000	0.10381500
C	2.69397700	0.15258800	0.37619900
H	1.79559000	-0.46435000	2.21961200
H	1.20012900	1.08750500	1.63318100
C	1.88226600	-1.94063300	-0.81643100
H	0.85377200	-2.67233400	0.93606600
H	-0.20877700	-2.41041900	-0.43692300
C	3.07072800	-1.23851100	-0.14311800
H	3.53692600	0.61801800	0.90147000
H	2.16155300	-2.96117900	-1.09686500
H	1.62772700	-1.40827900	-1.73586600
H	3.90326900	-1.14403400	-0.84562100
H	3.43104100	-1.83079700	0.70612100
O	2.41386900	0.90258500	-0.80958000
O	2.00077900	2.24125600	-0.44499500
H	1.03281900	2.12813300	-0.48116900
O	-0.71170100	1.24392400	-0.33504200
O	-1.49021100	2.39517900	0.12515000
H	-2.12056500	2.48742800	-0.60208000
C	-3.51366600	-0.01962900	0.00370000
H	-3.48597800	0.79566200	-0.72675700
H	-3.70497000	0.44534000	0.97681700
C	-4.66534400	-0.96324800	-0.35534200
H	-4.75707400	-1.77164100	0.37660500
H	-5.62005700	-0.43173200	-0.38386900
H	-4.51091800	-1.42202300	-1.33684000

R25

C	0.57221800	0.15048700	0.99770900
C	1.80066000	-0.18019600	0.13656700
O	2.79715900	0.91717100	0.24434800
O	2.40438600	2.03204300	-0.32851100
O	-0.68263300	0.82425400	-0.98989300

O	-0.47383500	2.22800800	-0.69481100
H	1.52943500	-0.19666000	-0.92113000
H	0.49913600	2.28159800	-0.75216500
H	0.67841200	1.16838100	1.37785100
H	0.53347100	-0.50774300	1.87027900
C	-0.77196500	0.06873800	0.23982300
C	-1.08283300	-1.35654500	-0.25866200
C	-1.91592600	0.59581500	1.12453400
C	-2.46133500	-1.47260100	-0.92665500
H	-1.03663000	-2.02413100	0.61006900
H	-0.30291300	-1.68192900	-0.95418200
C	-3.28663900	0.48444300	0.44519300
H	-1.91546700	0.02013600	2.05853900
H	-1.70272200	1.63458800	1.38376600
C	-3.57649500	-0.95058400	-0.01230100
H	-2.64489500	-2.51763600	-1.19631500
H	-2.45521300	-0.89880300	-1.85782600
H	-4.06356700	0.82952300	1.13460700
H	-3.30829200	1.15510100	-0.41927000
H	-4.53929600	-0.99666400	-0.53074100
H	-3.66356800	-1.60359200	0.86678600
C	2.56003600	-1.43737400	0.53945700
H	2.93306300	-1.30290400	1.56108600
H	1.83149500	-2.25313800	0.58304100
C	3.70685600	-1.80471600	-0.40574100
H	4.45021600	-1.00656500	-0.45245200
H	4.20987300	-2.71439800	-0.06964200
H	3.33939100	-1.98176800	-1.42091100

TS25

C	-3.39764400	-0.35687900	-0.87773400
C	-1.99023700	-0.91776700	-1.12141800
C	-0.90273700	-0.22327400	-0.27168400
C	-1.30374200	-0.22106400	1.21462600
C	-2.70685300	0.35524000	1.44664300
C	-3.76451700	-0.37597900	0.61117500
H	-1.96906300	-1.98730300	-0.88099000
H	-1.72018700	-0.82231400	-2.17777900
H	-3.43867300	0.67081600	-1.24909300
H	-4.12271500	-0.93647200	-1.45832200
H	-1.26598900	-1.25763500	1.57320400
H	-0.57252600	0.34907600	1.78905800
H	-2.95196900	0.29326300	2.51176300
H	-2.69977900	1.41720000	1.18365100
H	-3.84590400	-1.41627800	0.95508200

H	-4.74838200	0.07933400	0.76213900
C	0.44635200	-0.93615800	-0.52804500
H	0.62092900	-0.96570500	-1.60913500
H	0.33716600	-1.97541700	-0.20081600
C	1.71334900	-0.38472200	0.12527400
H	1.53402100	0.00193800	1.13164900
C	2.86310600	-1.42912600	0.15728300
H	2.56379800	-2.21831000	0.85898600
H	2.93722300	-1.87668400	-0.83713100
C	4.18084300	-0.82367900	0.56800600
H	4.36889200	-0.73611700	1.63658900
H	5.05819800	-1.09613100	-0.01305700
H	3.92540500	0.49289600	0.23681100
O	2.17606400	0.71110900	-0.68999000
O	3.20594600	1.38205300	0.01157900
O	-0.90543700	1.11277500	-0.82136500
O	-0.17005300	2.02898600	0.02802000
H	0.70985600	1.98491400	-0.38332200

P25

C	-3.39307900	-0.37411000	-0.84442300
C	-1.98195100	-0.90352000	-1.13188800
C	-0.88647300	-0.20443300	-0.29473500
C	-1.25077800	-0.23777000	1.20038100
C	-2.66039600	0.30204100	1.47681800
C	-3.72299200	-0.43380100	0.65181000
H	-1.93746400	-1.97671400	-0.91206600
H	-1.73862100	-0.78368700	-2.19226200
H	-3.46133900	0.66100900	-1.19033200
H	-4.12107200	-0.95395300	-1.42118900
H	-1.17907500	-1.27888500	1.54026200
H	-0.52008400	0.34178100	1.76564000
H	-2.87857500	0.21404300	2.54601200
H	-2.68190800	1.36888800	1.23504200
H	-3.77438400	-1.48279300	0.97452200
H	-4.71204000	-0.00274500	0.83694400
C	0.46649500	-0.89168100	-0.60165000
H	0.63084000	-0.84839300	-1.68408400
H	0.35785100	-1.95073600	-0.34640800
C	1.74692200	-0.39217200	0.08112000
H	1.56426800	-0.06695700	1.10792100
C	2.85105700	-1.47280900	0.05055200
H	2.44509800	-2.34155000	0.59644200
H	2.98706400	-1.79756800	-0.98602000
C	4.15887400	-1.08595800	0.65145400

H	4.20190800	-0.70956800	1.66740000
H	5.09017200	-1.39179000	0.19045600
O	-0.92858100	1.14053900	-0.81815600
O	-0.18109800	2.05306400	0.02286700
H	0.70427300	1.97321000	-0.37440800
O	2.16464600	0.76944500	-0.67341600
O	3.08641800	1.54987200	0.12539200
H	3.92593300	1.08832300	-0.04400100

R26

C	-1.17823600	-1.33558100	-0.65978600
C	-0.00260400	-2.08634900	-0.01653000
C	1.09824900	-1.13612300	0.46275600
C	0.54309700	-0.02584200	1.36218900
C	-0.65982500	0.69482000	0.75916800
C	-1.75997400	-0.25265800	0.26796800
H	-0.34991600	-2.66115700	0.84988500
H	0.42834100	-2.80045300	-0.72276000
H	-0.84619700	-0.87129100	-1.59228100
H	-1.96904600	-2.04506100	-0.92196900
H	0.22795100	-0.46721700	2.31375400
H	1.32510600	0.69734500	1.59205600
H	-1.05903000	1.42997500	1.46384400
H	-2.12553400	-0.74982000	1.17745900
O	1.67258700	-0.62193100	-0.74759000
O	2.76355000	0.26496100	-0.41376400
H	2.34894100	1.13211400	-0.56820600
O	-0.24171300	1.48894500	-0.42224300
O	0.71205700	2.35097700	-0.14707900
C	-2.93805900	0.49413800	-0.36898000
H	-3.73672400	-0.20508600	-0.62983600
H	-3.35518400	1.23773200	0.31663900
H	-2.63108200	1.01183200	-1.28001200
H	1.87464200	-1.68696300	1.00612000

TS26

C	-1.03675500	1.87179100	-0.13647800
C	0.36359200	1.61389500	-0.71065700
C	-0.77505600	-0.11725800	1.39955900
C	-1.67862700	0.58040400	0.37769600
H	0.27260500	0.98108500	-1.59679500
H	0.81085100	2.55862200	-1.03440100
H	-0.98902100	2.58581500	0.69403500
H	-1.68171300	2.31324300	-0.90064500
H	-1.21359300	-1.07000300	1.69844200

H	-0.73453400	0.51121900	2.29539100
H	-2.65020700	0.78509600	0.84448700
C	2.64628300	0.58672300	-0.31898800
H	2.86360700	1.00698100	-1.29875700
H	3.50441800	0.55941900	0.35036200
H	2.45236600	-0.77555700	-0.54163100
O	0.61627400	-1.37008000	-0.08363000
O	1.93952800	-1.79478500	-0.33034000
C	1.31009200	0.93182400	0.29365700
H	1.47399200	1.60576700	1.14903000
C	0.65476100	-0.34486000	0.91750300
H	1.28241000	-0.71056400	1.73702900
O	-1.89055600	-0.18436000	-0.81743800
O	-2.27486600	-1.53860600	-0.48396500
H	-1.41997000	-1.98358700	-0.60731700

P26

C	1.04064500	-1.88780800	-0.14749400
C	-0.36067600	-1.65151800	-0.72946800
C	0.71684000	0.09243300	1.39220800
C	1.65496600	-0.59326000	0.39306800
H	-0.27788500	-1.01444800	-1.61365600
H	-0.79138100	-2.60318100	-1.05527000
H	0.99762300	-2.61446900	0.67219300
H	1.70259900	-2.30533000	-0.91066900
H	1.14332500	1.04281300	1.71401900
H	0.64499700	-0.54616300	2.27898200
H	2.61610400	-0.79197000	0.88356600
C	-2.68878900	-0.79212900	-0.27083400
H	-2.96926000	-1.22529500	-1.22415700
H	-3.47887200	-0.39143700	0.35444200
C	-1.31502800	-0.98361000	0.27848700
H	-1.39684600	-1.64681400	1.16026700
C	-0.69821200	0.32319300	0.85366100
H	-1.33819600	0.72142800	1.64622800
O	1.89074700	0.18350700	-0.78990800
O	2.28032700	1.53049500	-0.43324400
H	1.41853600	1.97367300	-0.51485900
O	-0.58320600	1.35258800	-0.14852800
O	-1.84507500	2.04364700	-0.26659600
H	-2.31158300	1.47549600	-0.90266300

R27

C	-2.10605800	-1.70889700	0.79513700
C	-0.70009900	-1.12175700	0.98390400

C	-0.14603100	-0.57118300	-0.34514500
C	-1.10488400	0.48849500	-0.94160100
C	-2.51286600	-0.09698300	-1.11683300
C	-3.06950400	-0.67977800	0.18990000
H	-0.74034600	-0.31256100	1.71789700
H	-0.02289100	-1.87948800	1.38887100
H	-2.04946400	-2.58759100	0.13877700
H	-2.48821000	-2.06386000	1.75713200
H	-0.72556600	0.83842100	-1.90771100
H	-3.16671000	0.68878500	-1.50256500
H	-2.46073100	-0.88148400	-1.88129700
H	-3.22977900	0.13420000	0.90398800
H	-4.04693600	-1.13576900	0.00485000
H	-0.15797500	-1.39841200	-1.06743800
C	1.33937100	-0.12655300	-0.27036600
H	1.49593500	0.81337300	-0.80089600
O	-1.27656500	1.63996600	-0.10538700
O	-0.19109900	2.57018700	-0.35181500
H	0.38464000	2.37516700	0.41192700
O	1.75151700	0.14017700	1.13117900
O	1.42523500	1.32860700	1.58427500
C	2.32040800	-1.19180900	-0.75334000
H	2.02756600	-1.45146200	-1.77578600
H	2.18293500	-2.09772700	-0.15229600
C	3.78621500	-0.75072700	-0.72893300
H	4.11081800	-0.50860900	0.28458800
H	4.43363500	-1.54406100	-1.10969300
H	3.94107300	0.13474000	-1.35269900

TS27

C	2.13923600	-2.01213700	-0.05067100
C	0.73404700	-1.61978700	-0.52371300
C	0.11023200	-0.52469800	0.36577200
C	1.04118200	0.69539700	0.50016200
C	2.45949900	0.30038500	0.92356700
C	3.06221200	-0.78992200	0.02874900
H	0.78672200	-1.25197900	-1.55458000
H	0.07219900	-2.48795300	-0.53044900
H	2.07356200	-2.48354100	0.93870100
H	2.56093000	-2.76393200	-0.72485400
H	0.62912000	1.40563000	1.22375700
H	3.08153600	1.19909100	0.92945900
H	2.41389200	-0.05318800	1.96091200
H	3.21475500	-0.38888800	-0.97992200
H	4.04927300	-1.07521000	0.40528700

H	0.00050900	-0.92700300	1.38182000
C	-1.29901900	-0.15277400	-0.11765800
H	-1.29742300	0.05596200	-1.19131900
C	-1.97484400	1.02577900	0.63695900
H	-1.43011300	1.93881600	0.37073000
H	-1.85835200	0.85548100	1.71085800
C	-3.43284500	1.16235500	0.27966300
H	-3.66562400	1.73960200	-0.61324500
H	-3.66663200	-0.14398500	-0.17792800
O	-2.10171900	-1.31768800	0.11369100
O	-3.31588200	-1.16094700	-0.58704700
H	-4.13585300	1.30867300	1.09567000
O	1.01722500	1.33182800	-0.79037200
O	1.62183000	2.64632100	-0.64164200
H	2.36168300	2.56871800	-1.25890000

P27

C	2.32665200	-1.76574500	-0.46112300
C	0.87807000	-1.49870200	-0.89695500
C	0.05753600	-0.85359600	0.23754600
C	0.73995000	0.43965600	0.72916100
C	2.17719000	0.16454100	1.18534200
C	3.00822200	-0.50057400	0.07803900
H	0.85643200	-0.84805200	-1.77537000
H	0.40051100	-2.43759200	-1.19457900
H	2.32894600	-2.53682300	0.32058500
H	2.90086400	-2.17296900	-1.29904400
H	0.16074400	0.90435500	1.53179700
H	2.63365600	1.10324700	1.50905300
H	2.13614500	-0.48314500	2.06877800
H	3.14584800	0.20997100	-0.74454000
H	4.00737300	-0.73720800	0.45624200
H	0.12184600	-1.52665200	1.10462700
C	-1.45659400	-0.79993800	-0.09070800
C	-2.35680100	-0.38407000	1.08388600
H	-2.17090800	0.67789700	1.30007400
H	-2.04719800	-0.94090800	1.97625000
C	-3.80442400	-0.59876400	0.80947700
H	-4.50364400	-0.74963100	1.62226600
H	-4.19278200	-0.42398500	-0.18516500
O	0.74536500	1.36463800	-0.38712400
O	1.11877700	2.67472400	0.12165900
H	1.93352800	2.82970100	-0.37626200
O	-1.77441400	-0.09769100	-1.30026400
O	-1.89524500	1.32684700	-1.05317000

H	-0.95611500	1.58986600	-1.03389300
H	-1.72991300	-1.82305700	-0.37762400

R28

C	-2.23438400	-1.85647400	0.91641700
C	-0.97104400	-1.00940300	1.12538300
C	-0.39066000	-0.53701800	-0.22236500
C	-1.45069800	0.25282900	-1.02880400
C	-2.71698800	-0.59201600	-1.22540500
C	-3.29282800	-1.09875900	0.10449800
H	-1.21638000	-0.13625400	1.73576200
H	-0.21920100	-1.57873600	1.67977300
H	-1.96856000	-2.78537600	0.39430800
H	-2.64339600	-2.15202600	1.88741600
H	-1.04339400	0.54352500	-2.00311000
H	-3.45239200	0.01040500	-1.76394700
H	-2.46011100	-1.44296200	-1.86764900
H	-3.65577500	-0.24456000	0.68484300
H	-4.15752000	-1.74135600	-0.08769100
H	-0.19451500	-1.43577300	-0.82210500
C	0.98593200	0.17092900	-0.10016700
H	1.03343200	1.05082400	-0.74271400
O	-1.89335600	1.44893600	-0.37454500
O	-0.96086700	2.52108300	-0.66635900
H	-0.43659200	2.52709400	0.15699800
O	1.20917700	0.67857100	1.27784700
O	0.64436700	1.83529200	1.53672300
C	2.17281900	-0.75345300	-0.35550400
H	2.02553000	-1.19721700	-1.34655100
H	2.13939400	-1.58222700	0.36220000
C	3.54039800	-0.06227500	-0.30242500
H	3.67910400	0.39182300	0.68268900
H	3.55347800	0.76091300	-1.02674600
C	4.69650600	-1.02216800	-0.59526800
H	5.65829800	-0.50499800	-0.55490200
H	4.72815300	-1.83774300	0.13360600
H	4.60156700	-1.46956900	-1.58967400

TS28

C	2.69890100	-1.81032600	0.20183800
C	1.30565500	-1.62372600	-0.41152800
C	0.48386100	-0.55413700	0.33653400
C	1.25285300	0.77602900	0.44812400
C	2.66381600	0.58548600	1.01381200
C	3.46495700	-0.48286000	0.25905200

H	1.40733200	-1.32332400	-1.46043200
H	0.75066200	-2.56375400	-0.40354900
H	2.60006700	-2.21742800	1.21670000
H	3.26246500	-2.54988100	-0.37527500
H	0.69931100	1.47931000	1.07805800
H	3.17602400	1.55096000	0.99916500
H	2.56658400	0.30086500	2.06868000
H	3.65993300	-0.13647000	-0.76251100
H	4.44071200	-0.62013400	0.73507100
H	0.33266400	-0.89607500	1.36932000
C	-0.91171100	-0.39123800	-0.28361600
H	-0.83865400	-0.26214000	-1.36744900
C	-1.78207300	0.74019700	0.31772700
H	-1.33335500	1.69894200	0.03255000
H	-1.74634700	0.65801800	1.40930200
C	-3.21898400	0.67491900	-0.15822200
H	-3.38380000	1.13700400	-1.13375900
H	-3.24444800	-0.64565600	-0.53282300
O	-1.59030600	-1.62922500	-0.02429900
O	-2.74831600	-1.66846700	-0.83044400
O	1.27105000	1.31471700	-0.88633600
O	1.70688300	2.69940500	-0.79449800
H	2.50467000	2.66748500	-1.33938000
C	-4.31722400	0.92074400	0.84508500
H	-4.29352600	1.95865700	1.20798400
H	-5.30563600	0.75072800	0.41165200
H	-4.20945600	0.27093000	1.71850000

P28

C	-2.66870800	-1.82574600	0.33454700
C	-1.30854500	-1.46411600	0.95045300
C	-0.36049900	-0.84397300	-0.09492000
C	-1.01919100	0.38107000	-0.76274800
C	-2.36386600	0.00782300	-1.39676900
C	-3.31669400	-0.62830400	-0.37418200
H	-1.43953400	-0.76581300	1.78167600
H	-0.84093300	-2.36046300	1.37041500
H	-2.53034300	-2.63977500	-0.38926200
H	-3.33901800	-2.21134600	1.10902400
H	-0.35043100	0.82493000	-1.50533300
H	-2.80679300	0.90115800	-1.84436200
H	-2.17108600	-0.68928200	-2.22046200
H	-3.59396400	0.12300400	0.37349900
H	-4.24348100	-0.93335100	-0.86946300
H	-0.26694200	-1.56905000	-0.91617000

C	1.08562800	-0.68860400	0.44168700
C	2.12673600	-0.29404000	-0.61395400
H	1.93122300	0.74102100	-0.93189700
H	1.97515200	-0.92009200	-1.50445400
C	3.53222700	-0.42200800	-0.12824100
H	3.71040900	-0.27159200	0.93045800
O	-1.22220200	1.36953000	0.27813300
O	-1.57667700	2.62612600	-0.36186900
H	-2.46442800	2.76144200	-0.00215000
O	1.18768500	0.09806400	1.63796900
O	1.28973000	1.50982700	1.32096800
H	0.35504000	1.72460000	1.14329300
H	1.36077000	-1.67723300	0.83046500
C	4.67885500	-0.36361100	-1.07772200
H	4.84828900	0.65879700	-1.45719600
H	5.61216400	-0.68387300	-0.60761200
H	4.50996600	-0.99240300	-1.96098300

R29

C	-0.91826500	-1.25626900	-0.54744800
C	0.24978100	-2.06634500	0.03612900
C	1.42998500	-1.17912200	0.43910500
C	0.99140100	-0.03237800	1.35636100
C	-0.20278500	0.74864000	0.81546700
C	-1.38535500	-0.13496300	0.40129300
H	-0.07861400	-2.61298300	0.92777700
H	0.59584500	-2.81033900	-0.68588100
H	-0.61391300	-0.81661600	-1.50131800
H	-1.75173900	-1.93066300	-0.75852800
H	0.70816500	-0.44781000	2.32939600
H	1.82413100	0.64805300	1.53241900
H	-0.51690400	1.51196500	1.53315500
H	-1.72433200	-0.60363000	1.33728900
O	1.96050300	-0.70865600	-0.80844000
O	3.11764400	0.11718100	-0.54926100
H	2.74397900	1.00538600	-0.68782000
O	0.18947200	1.50783200	-0.39806800
O	1.20436000	2.31692800	-0.18891900
C	-2.55782900	0.69721300	-0.15229600
H	-2.73212700	1.54219700	0.52464200
H	-2.26801300	1.13306900	-1.11309000
H	2.20421800	-1.76973700	0.94234000
C	-3.86443200	-0.08687200	-0.31043900
H	-4.67184700	0.57214700	-0.63945200

H	-3.77672200	-0.88665000	-1.04974400
H	-4.17297200	-0.54009600	0.63728600

TS29

C	-1.21509000	1.93252600	-0.14806100
C	0.11647500	1.53402700	-0.80046900
C	-1.03981800	-0.04433900	1.41441000
C	-1.93929600	0.71603300	0.43467700
H	-0.08936700	0.89632000	-1.66359500
H	0.62682600	2.42682200	-1.17499200
H	-1.04937100	2.65372100	0.66082700
H	-1.86358700	2.41853800	-0.88169400
H	-1.54406200	-0.94644600	1.76280100
H	-0.88495000	0.59409200	2.29057100
H	-2.85615500	1.01859800	0.95597300
C	2.32514700	0.30965400	-0.53198100
H	2.44161900	0.67226200	-1.55447200
H	1.97972700	-0.99674200	-0.71714500
O	0.13105900	-1.45021900	-0.12030200
O	1.39301200	-1.99113000	-0.44736800
C	1.05864600	0.78486600	0.15935600
H	1.34092200	1.45860800	0.98397900
C	0.32922300	-0.41270400	0.84940800
H	0.96783100	-0.82254800	1.63909700
C	3.60509700	0.25988200	0.26613700
H	3.46787700	-0.26130100	1.21788900
H	4.40210000	-0.24731300	-0.28282800
H	3.95974100	1.27396600	0.49666000
O	-2.29691800	-0.04581600	-0.72733900
O	-2.77386900	-1.35584700	-0.34142700
H	-1.96772700	-1.87416200	-0.50244600

P29

C	0.73846100	-2.12725400	0.00215600
C	-0.52472100	-1.63821600	-0.72112200
C	0.77266600	0.02167100	1.34498300
C	1.59983500	-0.97295900	0.52114800
H	-0.23877000	-1.12655100	-1.64326700
H	-1.14215400	-2.49527200	-1.00586100
H	0.46390600	-2.75309200	0.85953400
H	1.34261700	-2.74799400	-0.66488300
H	1.39206200	0.87243100	1.62915600
H	0.47709600	-0.47033300	2.27885500
H	2.41862400	-1.35573000	1.14324300
C	-2.62142800	-0.23737000	-0.55155600
H	-2.61574500	-0.19742900	-1.63589800

C	-1.37120500	-0.68100700	0.14148400
H	-1.65985900	-1.20892200	1.06514400
C	-0.50447600	0.50672000	0.65254400
H	-1.08854300	1.12230900	1.34786700
C	-3.74813200	0.40604700	0.18176900
H	-3.58093200	1.48455000	0.34157700
H	-4.68908400	0.31854900	-0.36884000
H	-3.89336300	-0.03613100	1.17427300
O	2.16601300	-0.39169900	-0.66081200
O	2.98982800	0.73955600	-0.28819500
H	2.35602800	1.46797800	-0.41533600
O	-0.24368300	1.28667400	-0.52291500
O	0.61070000	2.41070400	-0.13293400
H	0.22926700	3.09797000	-0.69516700

R30

C	-0.05906900	-1.31427000	-0.43350300
C	1.19135100	-2.08106800	0.02439800
C	2.36550900	-1.15069500	0.33594900
C	1.96489100	-0.03932900	1.31229700
C	0.69499900	0.69804800	0.89688400
C	-0.48353500	-0.22825500	0.57511300
H	0.96983300	-2.65531500	0.93148400
H	1.50007000	-2.79776500	-0.74084800
H	0.13556100	-0.84792200	-1.40334000
H	-0.87870300	-2.02193700	-0.57898800
H	1.78860300	-0.48285200	2.29814900
H	2.78107300	0.67348900	1.42656200
H	-0.71561300	-0.72513900	1.52886300
O	2.76042200	-0.63678700	-0.94432000
O	3.90047300	0.23464800	-0.77266400
H	3.47859800	1.10798700	-0.85724800
O	0.94423200	1.49121200	-0.33257900
O	1.93772100	2.34169400	-0.19900900
H	3.20631800	-1.71526600	0.75519900
H	0.41702700	1.43644300	1.65429600
C	-1.73428600	0.56200400	0.14559200
H	-1.88319100	1.38744800	0.85360000
H	-1.55053300	1.02726500	-0.82845700
C	-3.02256600	-0.26805000	0.08544900
H	-2.92426900	-1.06184700	-0.66350300
H	-3.17620000	-0.77278300	1.04875300
C	-4.26233100	0.57141400	-0.24569100
H	-4.37617400	1.36002300	0.50789400

H	-4.10356000	1.08551900	-1.20106300
C	-5.54923500	-0.25509700	-0.31841500
H	-5.75167500	-0.75773900	0.63261700
H	-6.41336500	0.37250600	-0.55206900
H	-5.48095000	-1.02647600	-1.09176900

TS30

C	-2.13090700	1.88418000	-0.14844800
C	-0.78055700	1.55924100	-0.80306000
C	-1.83629900	-0.06886100	1.42587300
C	-2.78144200	0.63141400	0.44431200
H	-0.95199700	0.90532200	-1.66154100
H	-0.32385900	2.47767400	-1.18479400
H	-2.00468200	2.61875500	0.65552300
H	-2.80886900	2.32719600	-0.88268700
H	-2.28637200	-0.99624400	1.78203200
H	-1.71603600	0.58350100	2.29718200
H	-3.71280400	0.88344700	0.96681000
C	1.49649400	0.46629700	-0.53293400
H	1.59238500	0.82822400	-1.55902200
H	1.22653000	-0.85932000	-0.71166400
O	-0.59087600	-1.41626000	-0.10149200
O	0.69920100	-1.88502000	-0.43251700
C	0.20646200	0.87198100	0.15804900
H	0.45111800	1.56638800	0.97756000
C	-0.44975700	-0.36113000	0.85881300
H	0.21426500	-0.72651600	1.64939700
C	2.78276100	0.49471500	0.25994700
H	2.62831000	0.01413800	1.23415500
H	3.03385600	1.54462800	0.48256800
O	-3.09773500	-0.15742100	-0.71177000
O	-3.49437100	-1.49124200	-0.31655300
H	-2.65871800	-1.96114000	-0.47598600
C	3.97142900	-0.16443000	-0.45172000
H	4.10734100	0.30469800	-1.43330700
H	3.73235700	-1.21598400	-0.64426300
C	5.27415600	-0.07190300	0.34660400
H	6.09925600	-0.55365700	-0.18414400
H	5.55741800	0.97013700	0.52538200
H	5.17671200	-0.56072800	1.32086300

P30

C	1.77388700	-2.07623700	0.04284800
C	0.46426400	-1.71758400	-0.67369100
C	1.62834600	0.09094700	1.34845300
C	2.53303300	-0.83958000	0.53229800

H	0.69328600	-1.19817400	-1.60723500
H	-0.07485100	-2.63239400	-0.93688400
H	1.56608500	-2.70866600	0.91411400
H	2.42524700	-2.65173200	-0.62033900
H	2.17039900	1.00008500	1.60824100
H	1.38919400	-0.40830400	2.29452500
H	3.38945300	-1.13655300	1.15070300
C	-1.75101700	-0.51058100	-0.49670700
H	-1.76729200	-0.50013300	-1.58295200
C	-0.45639000	-0.82679300	0.18418300
H	-0.68302900	-1.36240000	1.12044000
C	0.30486600	0.44398500	0.66399700
H	-0.32553900	1.01627500	1.35557800
C	-2.92114900	0.06079900	0.23228100
H	-2.79933100	1.15347600	0.35018300
H	-2.95234500	-0.33472500	1.25752800
O	3.03246700	-0.23124700	-0.66627900
O	3.75247200	0.97780000	-0.32391900
H	3.05302600	1.64254200	-0.45571500
O	0.48041700	1.22327200	-0.52795700
O	1.23046300	2.42839500	-0.16646600
H	0.77961000	3.06795200	-0.73357800
C	-4.27122100	-0.19578700	-0.45735300
H	-4.42312400	-1.27648700	-0.55182200
H	-4.22769400	0.19740200	-1.47965000
C	-5.45436200	0.43133100	0.28398800
H	-6.39790400	0.23131500	-0.23065500
H	-5.54090800	0.03437200	1.30036700
H	-5.34236800	1.51739200	0.36232600

R31

C	-0.42916700	-1.35505100	-0.50081200
C	0.83614600	-2.07791200	-0.01322600
C	1.95897100	-1.10456100	0.35318800
C	1.47986100	-0.02826700	1.33324800
C	0.19407300	0.66256100	0.88808300
C	-0.93268600	-0.30666200	0.51106200
H	0.60893400	-2.67762300	0.87584600
H	1.20030500	-2.76707200	-0.77941600
H	-0.22093800	-0.86270500	-1.45476200
H	-1.21340000	-2.09269900	-0.68734100
H	1.29055100	-0.49595100	2.30537300
H	2.26096100	0.71599700	1.48623100
H	-0.14018700	1.37439300	1.64828100

H	-1.17432200	-0.83011600	1.44809500
O	2.37316400	-0.55285100	-0.90505600
O	3.46908000	0.36231300	-0.68257900
H	3.01348900	1.21843100	-0.76803500
O	0.44844400	1.48826400	-0.31870100
O	1.40179700	2.37550000	-0.13901800
C	-2.20073100	0.43883000	0.05396900
H	-2.39923800	1.25408800	0.76184700
H	-2.01124200	0.91719400	-0.91272700
H	2.80881700	-1.64086500	0.79101400
C	-3.45635000	-0.43695100	-0.04286100
H	-3.30681400	-1.22570300	-0.78719600
H	-3.61736300	-0.94589000	0.91578700
C	-4.70798300	0.36595000	-0.40998900
H	-5.58883200	-0.27812300	-0.47556700
H	-4.91578700	1.13910500	0.33616900
H	-4.58762100	0.86409500	-1.37674900

TS31

C	-1.68652900	1.89168000	-0.19545900
C	-0.31173500	1.55317200	-0.78942900
C	-1.47635000	-0.05614700	1.39880700
C	-2.37221500	0.64680400	0.37383700
H	-0.45104400	0.89644000	-1.65156500
H	0.16842700	2.46614500	-1.15511800
H	-1.58944500	2.62901400	0.61002600
H	-2.32840800	2.33652500	-0.96037900
H	-1.94886100	-0.97827400	1.73917000
H	-1.38882200	0.59947000	2.27157900
H	-3.32348800	0.90848600	0.85398700
C	1.94230800	0.44347100	-0.41577600
H	2.08607600	0.79848700	-1.43878800
H	1.67061100	-0.88192700	-0.59900400
O	-0.17624000	-1.42046300	-0.06706000
O	1.12328900	-1.90138100	-0.33824000
C	0.62703200	0.86272100	0.21668100
H	0.84174800	1.55914100	1.04291700
C	-0.06887700	-0.36186000	0.89380300
H	0.55729800	-0.72858400	1.71409200
C	3.19380600	0.46688600	0.43187400
H	2.99304100	-0.01084000	1.39789800
H	3.43725700	1.51564600	0.66317100
O	-2.64380200	-0.14514600	-0.79139700
O	-3.06900900	-1.47353400	-0.40773400
H	-2.23146700	-1.95146500	-0.52865300

C	4.40211700	-0.20000700	-0.23300900
H	4.63229600	0.26883900	-1.19432100
H	5.29104600	-0.12209400	0.39765100
H	4.21248900	-1.26112500	-0.41518900

P31

C	1.19877100	-2.11674900	-0.02164400
C	-0.10969600	-1.65190300	-0.67699600
C	1.25226700	0.02234300	1.33551200
C	2.05926600	-0.94724000	0.46336900
H	0.11748900	-1.12723100	-1.60815800
H	-0.72088500	-2.52099200	-0.93753500
H	0.98131100	-2.75454500	0.84329400
H	1.78238200	-2.71905600	-0.72300500
H	1.86527000	0.88562100	1.59413900
H	1.01543600	-0.48267300	2.27915300
H	2.91630800	-1.31605800	1.04074500
C	-2.22735000	-0.30256800	-0.38942800
H	-2.26803200	-0.22295300	-1.47223300
C	-0.93376400	-0.72097500	0.23582500
H	-1.16222900	-1.26265900	1.16778100
C	-0.06872300	0.48268000	0.71225200
H	-0.63063800	1.07970300	1.44111700
C	-3.35267100	0.27506500	0.40321700
H	-3.16457300	1.34486400	0.60516200
H	-3.38991000	-0.19884100	1.39347300
O	2.55285400	-0.34482300	-0.74035000
O	3.36946600	0.80168100	-0.39971600
H	2.71445300	1.51683300	-0.48866600
O	0.11549600	1.27734800	-0.46805000
O	0.96296800	2.41729600	-0.11082900
H	0.53670700	3.10121500	-0.64420800
C	-4.71829600	0.14649200	-0.28607900
H	-4.97742700	-0.90297800	-0.44879300
H	-5.50977800	0.60262200	0.31432000
H	-4.71098200	0.64224500	-1.26119100

R32

C	1.93739100	-1.55134300	-0.78692500
C	0.44319400	-1.20385500	-0.84713700
C	-0.03930700	-0.59724700	0.48504700
C	0.79715800	0.65158100	0.85696600
C	2.29116700	0.30247200	0.90406700
C	2.78444100	-0.33057300	-0.40482500
H	0.26977900	-0.48392600	-1.65140700

H	-0.14489600	-2.09378300	-1.09064500
H	2.09647800	-2.35160200	-0.05184500
H	2.26164400	-1.94841400	-1.75367000
H	0.47656500	1.04279600	1.82859100
H	2.85018000	1.21333200	1.13103600
H	2.45054700	-0.39310700	1.73672700
H	2.73143800	0.41528100	-1.20423000
H	3.83679500	-0.61361700	-0.30509900
H	0.18646600	-1.32775000	1.27367400
C	-1.57680900	-0.39857400	0.55984100
H	-1.82619400	0.55229500	1.03056400
O	0.68943300	1.71929700	-0.09218300
O	-0.50283600	2.49168900	0.20033600
H	-1.11353300	2.12833700	-0.46887000
O	-2.19330600	-0.34179300	-0.79136200
O	-2.07127900	0.81283800	-1.40386300
C	-2.30421800	-1.55186800	1.23237100
H	-2.00829900	-1.60480400	2.28245000
H	-2.05554100	-2.50408100	0.75705000
H	-3.38562200	-1.41076000	1.18581800

TS32

C	-0.49454300	1.05749300	0.48718100
C	-2.01978600	1.11367500	0.64612700
C	-2.69763800	0.09510400	-0.28008000
C	-2.23326600	-1.32923700	0.04175000
C	-0.74033100	-1.47382300	0.25424400
C	0.03918100	-0.34158300	0.90357700
H	-3.78497700	0.15820400	-0.18018000
H	-2.27740300	0.91041800	1.69260100
H	-2.35465600	2.12733700	0.41597000
H	-0.01944700	1.83646700	1.08971600
H	-2.71875500	-1.65607400	0.97632600
H	-2.57419100	-2.03494500	-0.72274100
H	-0.47241800	-2.44902700	0.66847800
H	-0.10787100	-0.40484200	1.99524500
H	-2.45647000	0.34504000	-1.31575600
C	1.55415900	-0.60815200	0.68850700
C	2.51715000	0.38306800	1.32409100
H	1.76520000	-1.61944700	1.05563600
H	3.54596300	0.07231400	1.13257000
H	2.37577300	1.38882300	0.92983000
H	2.36287300	0.40352100	2.40582600
O	1.82494600	-0.61800500	-0.72913000
O	1.14292700	-1.70320400	-1.32566000

H	0.03784600	-1.59596100	-0.85825800
O	-0.29152100	1.38180100	-0.89444600
O	1.02571100	1.94048300	-1.09343700
H	1.51246400	1.13031100	-1.33626600

P32

C	-0.47876000	0.73039500	0.71942500
C	-1.99178300	0.85651000	0.92578200
C	-2.78857900	0.18708200	-0.20012200
C	-2.39527500	-1.29576700	-0.34899000
C	-0.91394500	-1.47428900	-0.40465100
C	-0.04964800	-0.75865900	0.59100700
H	-3.86118000	0.27579600	-0.00424100
H	-2.24603800	0.39636000	1.88900800
H	-2.24038000	1.91830900	0.99706200
H	0.04973400	1.22129300	1.53762100
H	-2.80444800	-1.84813600	0.51804000
H	-2.86906800	-1.73267000	-1.23253700
H	-0.48036200	-2.25876500	-1.01214000
H	-0.25842600	-1.19099800	1.58924700
H	-2.58960900	0.71309000	-1.13856300
C	1.46670400	-0.99597100	0.37595000
C	2.32541200	-0.71375200	1.60553100
H	1.60330900	-2.03762700	0.06473700
H	3.37597200	-0.90050700	1.37708700
H	2.22681400	0.32260500	1.93572800
H	2.02878900	-1.36958000	2.42675600
O	-0.14862800	1.47104100	-0.48298500
O	1.00444200	2.30922400	-0.22874500
H	1.72029800	1.66902900	-0.41259300
O	2.05379300	-0.16776700	-0.64863300
O	1.42101900	-0.43273000	-1.92167000
H	0.65482800	0.17036400	-1.86254900

R33

C	-2.10605800	-1.70889700	0.79513700
C	-0.70009900	-1.12175700	0.98390400
C	-0.14603100	-0.57118300	-0.34514500
C	-1.10488400	0.48849500	-0.94160100
C	-2.51286600	-0.09698300	-1.11683300
C	-3.06950400	-0.67977800	0.18990000
H	-0.74034600	-0.31256100	1.71789700
H	-0.02289100	-1.87948800	1.38887100
H	-2.04946400	-2.58759100	0.13877700
H	-2.48821000	-2.06386000	1.75713200

H	-0.72556600	0.83842100	-1.90771100
H	-3.16671000	0.68878500	-1.50256500
H	-2.46073100	-0.88148400	-1.88129700
H	-3.22977900	0.13420000	0.90398800
H	-4.04693600	-1.13576900	0.00485000
H	-0.15797500	-1.39841200	-1.06743800
C	1.33937100	-0.12655300	-0.27036600
H	1.49593500	0.81337300	-0.80089600
O	-1.27656500	1.63996600	-0.10538700
O	-0.19109900	2.57018700	-0.35181500
H	0.38464000	2.37516700	0.41192700
O	1.75151700	0.14017700	1.13117900
O	1.42523500	1.32860700	1.58427500
C	2.32040800	-1.19180900	-0.75334000
H	2.02756600	-1.45146200	-1.77578600
H	2.18293500	-2.09772700	-0.15229600
C	3.78621500	-0.75072700	-0.72893300
H	4.11081800	-0.50860900	0.28458800
H	4.43363500	-1.54406100	-1.10969300
H	3.94107300	0.13474000	-1.35269900

TS33

C	0.77975600	1.04926000	-0.50055300
C	2.27492700	1.07944200	-0.84490700
C	3.04581200	0.06258400	0.00773300
C	2.52516700	-1.35855000	-0.23233600
C	1.01554300	-1.48225700	-0.26264000
C	0.18004300	-0.34599900	-0.83077700
H	4.11365400	0.10643600	-0.22482100
H	2.40039500	0.85814900	-1.91169400
H	2.64982200	2.09053400	-0.67234000
H	0.24476800	1.82878300	-1.04975100
H	2.89005800	-1.70696800	-1.21285900
H	2.94544400	-2.05841100	0.49724700
H	0.68654000	-2.45797100	-0.62895100
H	0.19621200	-0.42551000	-1.93100400
H	2.93648600	0.33082900	1.06094300
C	-1.30195100	-0.58689800	-0.43459100
C	-2.32668600	0.40621700	-0.97733600
H	-1.56845900	-1.60155400	-0.75682600
H	-2.12803500	1.39561600	-0.56083500
H	-2.16711800	0.48012500	-2.05876500
O	-1.39865000	-0.57231300	1.00590600
O	-0.67042100	-1.66288800	1.53317200
H	0.37269200	-1.58031300	0.93636400

O	0.75379400	1.39169900	0.89160700
O	-0.51939600	1.97432200	1.24661200
H	-0.98591400	1.17395800	1.55305400
C	-3.77272900	-0.01166400	-0.69130300
H	-3.95027600	-0.09783500	0.38222500
H	-4.47373600	0.72380400	-1.09300400
H	-4.00444500	-0.97902400	-1.14709800

P33

C	0.79857100	0.56612000	-0.84326800
C	2.26999300	0.40982600	-1.24099600
C	3.11988700	-0.16461900	-0.10116600
C	2.55306500	-1.51306900	0.38491000
C	1.08442300	-1.43797000	0.64349600
C	0.19373800	-0.78698400	-0.37341900
H	4.15580500	-0.28696800	-0.43077000
H	2.32198000	-0.25054900	-2.11580600
H	2.64694300	1.38807000	-1.54967700
H	0.22889500	0.97997700	-1.67621600
H	2.75489500	-2.27131600	-0.39523600
H	3.08481100	-1.85392100	1.27771200
H	0.63934200	-2.02072200	1.44031100
H	0.20323000	-1.42142400	-1.28123800
H	3.12974100	0.54721400	0.72958600
C	-1.29629600	-0.73812100	0.05077600
C	-2.27740700	-0.56082500	-1.11396900
H	-1.52621500	-1.67109500	0.57955800
H	-2.11619200	0.41969100	-1.57404700
H	-2.02530000	-1.30787300	-1.87323200
O	0.74796300	1.55246600	0.21864800
O	-0.30285400	2.50791100	-0.06328600
H	-1.06619000	2.03379300	0.32160300
O	-1.61222400	0.34632300	0.94714500
O	-0.86279700	0.21053600	2.17584900
H	-0.03131500	0.65481500	1.92016700
C	-3.74589800	-0.71108000	-0.70513700
H	-4.02253400	0.02848100	0.04748700
H	-4.40250900	-0.58249500	-1.56922800
H	-3.93655000	-1.70323600	-0.28518700

R34

C	-2.23438400	-1.85647400	0.91641700
C	-0.97104400	-1.00940300	1.12538300
C	-0.39066000	-0.53701800	-0.22236500
C	-1.45069800	0.25282900	-1.02880400

C	-2.71698800	-0.59201600	-1.22540500
C	-3.29282800	-1.09875900	0.10449800
H	-1.21638000	-0.13625400	1.73576200
H	-0.21920100	-1.57873600	1.67977300
H	-1.96856000	-2.78537600	0.39430800
H	-2.64339600	-2.15202600	1.88741600
H	-1.04339400	0.54352500	-2.00311000
H	-3.45239200	0.01040500	-1.76394700
H	-2.46011100	-1.44296200	-1.86764900
H	-3.65577500	-0.24456000	0.68484300
H	-4.15752000	-1.74135600	-0.08769100
H	-0.19451500	-1.43577300	-0.82210500
C	0.98593200	0.17092900	-0.10016700
H	1.03343200	1.05082400	-0.74271400
O	-1.89335600	1.44893600	-0.37454500
O	-0.96086700	2.52108300	-0.66635900
H	-0.43659200	2.52709400	0.15699800
O	1.20917700	0.67857100	1.27784700
O	0.64436700	1.83529200	1.53672300
C	2.17281900	-0.75345300	-0.35550400
H	2.02553000	-1.19721700	-1.34655100
H	2.13939400	-1.58222700	0.36220000
C	3.54039800	-0.06227500	-0.30242500
H	3.67910400	0.39182300	0.68268900
H	3.55347800	0.76091300	-1.02674600
C	4.69650600	-1.02216800	-0.59526800
H	5.65829800	-0.50499800	-0.55490200
H	4.72815300	-1.83774300	0.13360600
H	4.60156700	-1.46956900	-1.58967400

TS34

C	-0.99403500	-1.12094200	-0.37790900
C	-2.43746600	-1.34530600	-0.84795100
C	-3.38098700	-0.32317100	-0.19972700
C	-2.97251100	1.10748500	-0.56650200
C	-1.48530100	1.38289800	-0.47808600
C	-0.49571600	0.28183800	-0.82469500
H	-4.41133700	-0.50397600	-0.51909600
H	-2.47443600	-1.26157000	-1.94081700
H	-2.73307000	-2.36343200	-0.58586700
H	-0.33627800	-1.89849100	-0.77571800
H	-3.26775000	1.30184900	-1.61101900
H	-3.52679200	1.83870700	0.03085000
H	-1.21271800	2.33904900	-0.93185800
H	-0.40861500	0.23229000	-1.92330900

H	-3.35389800	-0.45643000	0.88408800
C	0.91092300	0.71910200	-0.33225200
C	2.07138700	-0.21777400	-0.65571900
H	1.11248600	1.71254500	-0.75249000
H	1.93210500	-1.16582500	-0.13066100
H	2.02432800	-0.44126800	-1.72835100
O	0.86358900	0.87966800	1.10211100
O	-0.01188400	1.94164000	1.42371800
H	-0.97779000	1.68351500	0.75173500
O	-1.07754100	-1.29606900	1.04281800
O	0.20124600	-1.69901100	1.58017300
H	0.55992200	-0.82447400	1.82248200
C	3.44442100	0.37649000	-0.31459300
H	3.46929700	0.63169300	0.74858300
H	3.57645600	1.31885200	-0.85954500
C	4.59627700	-0.57505600	-0.64747800
H	4.61461500	-0.82138000	-1.71404900
H	5.56209700	-0.13094300	-0.39324200
H	4.50614700	-1.51391400	-0.09281200

P34

C	1.04922300	0.37172800	-0.96134500
C	2.43342600	0.03373500	-1.52524300
C	3.42415900	-0.36575800	-0.42548700
C	2.88360900	-1.54832700	0.40222100
C	1.47556800	-1.32092500	0.84431300
C	0.46693900	-0.81648200	-0.14511900
H	4.39104200	-0.62427600	-0.86705600
H	2.32058200	-0.78778900	-2.24384900
H	2.79934900	0.90210600	-2.07866400
H	0.37635900	0.65685500	-1.77100600
H	2.93411600	-2.45841200	-0.22515500
H	3.53032000	-1.74262800	1.26254200
H	1.13315100	-1.70921800	1.79540800
H	0.30802700	-1.61379100	-0.89720200
H	3.59093400	0.49255600	0.23204100
C	-0.93597900	-0.57719300	0.46915400
C	-2.07633400	-0.54759000	-0.55426400
H	-1.12294500	-1.37389300	1.19924600
H	-1.95653800	0.32670500	-1.20415400
H	-1.97237000	-1.42988600	-1.19559200
O	1.20650200	1.54626200	-0.12556400
O	0.16354700	2.50095300	-0.43813300
H	-0.54745800	2.16662600	0.14357100
O	-1.05934700	0.67780100	1.16986300

O	-0.13207900	0.72355700	2.27769600
H	0.66707800	1.04928100	1.82022700
C	-3.47552500	-0.53368200	0.07474700
H	-3.56343900	0.33156000	0.73663100
H	-3.58994600	-1.41972800	0.71031700
C	-4.59206200	-0.50578100	-0.97193100
H	-4.54514300	-1.37910300	-1.63052500
H	-5.57716200	-0.50073600	-0.49851900
H	-4.52308600	0.38683900	-1.60143900

R35

C	-0.93781600	-0.08791300	0.22952200
C	-1.57848600	-1.42173900	-0.17075600
C	-2.98308500	-1.26963200	-0.77486500
C	-3.90556900	-0.44994800	0.13545000
C	-3.29035800	0.92038200	0.44600500
C	-1.88906000	0.78666000	1.05752700
H	-2.90511300	-0.77881000	-1.75040400
H	-0.91932800	-1.94747800	-0.86640400
H	-1.63275500	-2.03390500	0.73725000
H	-4.88594900	-0.32635100	-0.33436700
H	-3.93091100	1.48243400	1.13213200
H	-1.42513200	1.76794700	1.18776000
H	-1.96129100	0.33322400	2.05303300
H	-3.40605000	-2.26233800	-0.95639900
H	-4.07465000	-0.99609600	1.07301800
H	-3.22855500	1.50802900	-0.47608200
O	-0.74163900	0.59977700	-1.10101700
O	-0.09270200	1.73717700	-1.02692300
C	0.42805700	-0.22770800	0.90765200
H	0.26569600	-0.74665600	1.85801300
H	0.77651900	0.77169400	1.16321300
C	1.50631500	-0.96278500	0.08453700
H	1.34663900	-0.80654600	-0.98656100
H	1.42848100	-2.04103800	0.25511200
C	2.95027500	-0.51993700	0.38013700
H	3.08084800	-0.30793600	1.44813700
O	3.31387700	0.66320300	-0.35169500
O	2.66936500	1.82194800	0.23677200
H	1.90682000	1.94460700	-0.35292500
C	3.97827200	-1.55210100	-0.07478000
H	4.98933600	-1.17765600	0.09178300
H	3.85105000	-2.48225700	0.48390300
H	3.86187600	-1.76939600	-1.13987500

TS35

C	3.04203500	1.26019800	0.03087300
C	1.74738200	1.11831700	0.84516900
C	0.87427900	-0.05640400	0.38326800
C	1.68715300	-1.35844500	0.31434700
C	2.96426400	-1.20813400	-0.52251100
C	3.83511700	-0.05239100	-0.01323600
H	1.98441400	0.95292000	1.90262300
H	1.15757900	2.03876800	0.79545000
H	2.78879100	1.56536300	-0.98920200
H	3.65186300	2.06201600	0.45848700
H	1.95034200	-1.63061200	1.34339800
H	1.06296700	-2.16036800	-0.08258300
H	3.52534100	-2.14749800	-0.50038400
H	2.68805700	-1.02971400	-1.56651400
H	4.20327800	-0.29083800	0.99363800
H	4.71880300	0.06501000	-0.64793800
C	-0.40720300	-0.19535500	1.27340300
H	-0.69388300	0.80571600	1.59751600
H	-0.12320700	-0.77114700	2.16118800
C	-1.58573900	-0.85213900	0.57644700
H	-1.71612100	-1.91136200	0.79921400
H	-1.10527700	-0.90526700	-0.70434600
C	-2.90017300	-0.08878000	0.45243000
H	-3.09238600	0.44627700	1.39444400
O	0.41863800	0.34269600	-0.94061400
O	-0.29591000	-0.71869000	-1.53700500
O	-2.89940800	0.88350700	-0.60425700
O	-1.94487100	1.92973700	-0.29326900
H	-1.16478400	1.62931400	-0.79355200
C	-4.08033800	-0.99399700	0.11635700
H	-4.23530600	-1.72317000	0.91486100
H	-4.98821000	-0.39985700	0.00379600
H	-3.90051000	-1.53078000	-0.81821700

P35

C	-3.08857700	1.12013100	-0.24227500
C	-1.80969500	0.93069800	-1.07040100
C	-0.81285900	-0.05903400	-0.43630600
C	-1.50949700	-1.37975300	-0.07903500
C	-2.77299000	-1.17598800	0.76731900
C	-3.75871100	-0.22020500	0.08368900
H	-2.05914000	0.54782700	-2.06686300
H	-1.30829000	1.89245800	-1.22208400
H	-2.83441700	1.63330700	0.68965000

H	-3.77859800	1.77173100	-0.78779700
H	-1.77541600	-1.86957300	-1.02364800
H	-0.80673800	-2.03243600	0.43928500
H	-3.24639300	-2.14615400	0.94854500
H	-2.48720000	-0.77569100	1.74465400
H	-4.12897600	-0.67939000	-0.84288400
H	-4.63330200	-0.05695200	0.72105700
C	0.40178300	-0.26444800	-1.39827200
H	0.68075900	0.71614600	-1.79451700
H	0.02185000	-0.85392000	-2.24403400
C	1.60682900	-0.92786200	-0.82385800
H	1.54867600	-1.97362600	-0.53894900
C	2.84237500	-0.17365000	-0.47011700
H	3.13681800	0.50629900	-1.27561700
O	2.61597900	0.71036700	0.69527400
O	2.04814400	1.96407200	0.23880700
H	1.10043100	1.81291800	0.42504900
O	-0.39188700	0.65357000	0.76589100
O	0.21220300	-0.24340400	1.73562100
H	1.15892000	-0.02981600	1.60516300
C	4.00839300	-1.06252700	-0.06053600
H	4.27021400	-1.73172500	-0.88361100
H	4.87887400	-0.45732300	0.19492900
H	3.74411000	-1.67509000	0.80567400

R36

C	0.64197300	0.14896100	0.22522200
C	1.45126400	1.37459000	-0.21344200
C	2.82478700	1.02119900	-0.80460900
C	3.63129800	0.11887200	0.13712800
C	2.84243500	-1.14883300	0.48847000
C	1.46954400	-0.81382300	1.08724400
H	2.68456800	0.51269300	-1.76396300
H	0.86722700	1.95910500	-0.92907000
H	1.58357600	2.00343400	0.67493100
H	4.58794000	-0.14662600	-0.32269900
H	3.40327800	-1.76639500	1.19629200
H	0.88168600	-1.72156600	1.24691200
H	1.59879800	-0.34069700	2.06769900
H	3.37369800	1.94383400	-1.01602700
H	3.86845400	0.66891400	1.05748400
H	2.70601600	-1.75365700	-0.41426700
O	0.36017500	-0.55077000	-1.08390700
O	-0.42599000	-1.59490500	-0.97528400

C	-0.69625200	0.48680600	0.88779200
H	-0.47220200	1.00739900	1.82443300
H	-1.17372400	-0.45106600	1.16806000
C	-1.66700400	1.33026800	0.03366600
H	-1.49718900	1.15206200	-1.03206500
H	-1.48270800	2.39657000	0.20168800
C	-3.15021300	1.05002800	0.29884400
H	-3.36702300	0.94491000	1.36812300
O	-3.66407000	-0.07897100	-0.40465200
O	-3.19940400	-1.29265300	0.24533200
H	-2.45283600	-1.54481300	-0.32330300
H	-3.76224800	1.86588700	-0.09803900

TS36

C	2.57369600	1.44432700	-0.02365000
C	1.30784800	1.18967700	0.80852900
C	0.56007600	-0.08339800	0.38972100
C	1.50563100	-1.29382800	0.34521800
C	2.75228500	-1.03217500	-0.51007200
C	3.50042700	0.22194100	-0.04060000
H	1.56962200	1.08093500	1.86750000
H	0.62353100	2.04095300	0.74009600
H	2.28147800	1.69201300	-1.04889600
H	3.09857900	2.31782600	0.37519600
H	1.80544800	-1.50831000	1.37786000
H	0.96598700	-2.16760300	-0.02296900
H	3.40940600	-1.90640500	-0.47035600
H	2.44966700	-0.91163600	-1.55503500
H	3.89905200	0.05139400	0.96844400
H	4.36209900	0.41394300	-0.68724800
C	-0.69029600	-0.33201800	1.30071400
H	-1.06987800	0.64261300	1.61063100
H	-0.34018400	-0.86018500	2.19405700
C	-1.80888600	-1.11288200	0.63252800
H	-1.85712800	-2.16880000	0.89864200
H	-1.32673000	-1.16932400	-0.64978600
C	-3.17357100	-0.46385400	0.48150100
H	-3.92912500	-1.21421100	0.23398600
H	-3.46839600	0.03293700	1.41760800
O	0.05033900	0.22742900	-0.93805400
O	-0.55167600	-0.92065700	-1.49574900
O	-3.29323000	0.46056400	-0.59617800
O	-2.45401600	1.61098200	-0.31295200
H	-1.64802600	1.38057700	-0.80961400

P36

C	2.63834300	1.32559200	0.21404300
C	1.39487400	1.01162200	1.05757500
C	0.50561000	-0.08761300	0.44481200
C	1.33879900	-1.33041100	0.10052800
C	2.56410300	-1.00577200	-0.76336500
C	3.44694600	0.06135600	-0.10423500
H	1.69284400	0.67075200	2.05599700
H	0.79400200	1.91560000	1.20246100
H	2.32286200	1.79800200	-0.72068200
H	3.25956800	2.05371500	0.74534700
H	1.66585400	-1.77109300	1.05007700
H	0.70696800	-2.06561900	-0.39819700
H	3.13778600	-1.92213500	-0.93438100
H	2.22781000	-0.65535100	-1.74369300
H	3.87515100	-0.34276300	0.82309400
H	4.29168400	0.31064600	-0.75390300
C	-0.67440500	-0.41102800	1.41745000
H	-1.05899300	0.53811700	1.80233500
H	-0.22976300	-0.94465600	2.26844300
C	-1.79931700	-1.21178700	0.85576900
H	-1.63177000	-2.25417700	0.60839200
C	-3.09564500	-0.59420400	0.47264500
H	-3.82477300	-1.34445500	0.15881400
H	-3.53066400	0.01566600	1.27030400
O	-2.99896000	0.28105100	-0.69891400
O	-2.57588000	1.59856100	-0.25940100
H	-1.61748500	1.55484600	-0.44708000
O	0.00169200	0.55837900	-0.76338300
O	-0.49405500	-0.41323000	-1.72263500
H	-1.45930400	-0.30171700	-1.60398000

R37

C	-0.01423400	1.75542600	-0.05421300
C	-1.53645500	1.82032100	0.13698800
C	-2.27400100	0.85586500	-0.79835500
C	-1.74954600	-0.57463300	-0.65849200
C	-0.22740000	-0.65180200	-0.81028100
C	0.52200700	0.32120400	0.12309300
H	-3.34720600	0.86315700	-0.59815400
H	-1.78081900	1.56660000	1.17329000
H	-1.88773200	2.84310700	-0.02920200
H	0.24435400	2.11491300	-1.06027600
H	0.46991400	2.43199000	0.65478800
H	-2.22947800	-1.22978100	-1.39620200

H	0.10245300	-1.67689400	-0.63347600
H	0.01069000	-0.41147800	-1.85395100
H	0.33686000	0.01205600	1.15857800
H	-2.13933400	1.16479600	-1.84253700
C	2.89174500	1.13075100	0.78130700
H	3.94284600	0.88017700	0.63053600
H	2.75449200	2.18502800	0.53440900
H	2.63896400	0.98135000	1.83432500
O	-2.00790500	-1.11197400	0.65023400
O	-3.44780300	-1.19611200	0.82832800
H	-3.56021100	-2.15644500	0.83305200
C	2.03313100	0.24980500	-0.11247300
H	2.27223300	0.42147000	-1.16652900
O	2.40686300	-1.16060900	0.14272900
O	3.58640600	-1.46026300	-0.35680200

TS37

C	1.47960100	2.04867000	-0.30593400
C	-0.00883500	2.01264100	0.09245900
C	-0.52105200	0.55788200	-0.03316300
C	0.31125800	-0.30309600	0.88762400
C	1.76612900	-0.38766700	0.49856700
C	2.34511100	1.04764500	0.47990200
H	-0.14061200	2.36894600	1.12145200
H	-0.58107000	2.68042400	-0.55825800
H	1.56143200	1.83016400	-1.37521100
H	1.87878100	3.05729600	-0.16134100
H	0.17101100	-0.07908900	1.94902000
H	-0.48423100	-1.44391800	0.84533300
H	2.33650100	-1.01416200	1.19063300
H	2.43316800	1.36989100	1.52308000
H	3.35780400	1.00753600	0.06898100
H	-0.32205600	0.24394200	-1.06584200
C	-2.01997900	0.29177000	0.19413500
H	-2.28911300	0.43556700	1.24808700
C	-2.95923700	1.06243400	-0.71790000
H	-2.92197800	2.12994400	-0.49173100
H	-2.68803400	0.91821500	-1.76670400
O	-2.21710400	-1.10043800	-0.13547400
O	-1.55259500	-1.90826600	0.81911100
H	-3.98471700	0.71728200	-0.57526200
O	1.94664900	-0.90307000	-0.82600800
O	1.64655900	-2.32128200	-0.78686200
H	0.72705300	-2.33925400	-1.09253000

P37

C	-1.65863600	-1.84471600	-0.57381800
C	-0.18988200	-1.96040400	-0.13006600
C	0.43251700	-0.55019800	0.02998500
C	-0.40889000	0.23486700	0.98312500
C	-1.84601600	0.39761800	0.64490900
C	-2.50415100	-0.96601800	0.36358500
H	-0.12372300	-2.49874700	0.82371600
H	0.36698000	-2.54290100	-0.86959400
H	-1.68992200	-1.42145000	-1.58243400
H	-2.10851200	-2.84055500	-0.63565900
H	0.02786800	0.74056000	1.83352600
H	-2.38851300	0.93779500	1.42888700
H	-2.63310500	-1.46315500	1.33108800
H	-3.50404200	-0.80813600	-0.05241600
H	0.36416100	-0.07597400	-0.96028400
C	1.91675900	-0.52070600	0.42790600
H	2.04510200	-1.00598600	1.40398400
C	2.86252600	-1.14242500	-0.59273600
H	2.64850500	-2.20417100	-0.73705200
H	2.77216300	-0.63167100	-1.55320300
H	3.89326300	-1.04409000	-0.24663200
O	-2.01034900	1.12891400	-0.60688700
O	-1.25159500	2.36657900	-0.50930100
H	-1.96887200	3.01445200	-0.49156900
O	2.31561900	0.82834800	0.74778000
O	2.12548800	1.66676900	-0.42524900
H	1.23998600	2.03103900	-0.26045600

R38

C	-0.01423400	1.75542600	-0.05421300
C	-1.53645500	1.82032100	0.13698800
C	-2.27400100	0.85586500	-0.79835500
C	-1.74954600	-0.57463300	-0.65849200
C	-0.22740000	-0.65180200	-0.81028100
C	0.52200700	0.32120400	0.12309300
H	-3.34720600	0.86315700	-0.59815400
H	-1.78081900	1.56660000	1.17329000
H	-1.88773200	2.84310700	-0.02920200
H	0.24435400	2.11491300	-1.06027600
H	0.46991400	2.43199000	0.65478800
H	-2.22947800	-1.22978100	-1.39620200
H	0.10245300	-1.67689400	-0.63347600
H	0.01069000	-0.41147800	-1.85395100
H	0.33686000	0.01205600	1.15857800

H	-2.13933400	1.16479600	-1.84253700
C	2.89174500	1.13075100	0.78130700
H	3.94284600	0.88017700	0.63053600
H	2.75449200	2.18502800	0.53440900
H	2.63896400	0.98135000	1.83432500
O	-2.00790500	-1.11197400	0.65023400
O	-3.44780300	-1.19611200	0.82832800
H	-3.56021100	-2.15644500	0.83305200
C	2.03313100	0.24980500	-0.11247300
H	2.27223300	0.42147000	-1.16652900
O	2.40686300	-1.16060900	0.14272900
O	3.58640600	-1.46026300	-0.35680200

TS38

C	1.99383100	-0.04960400	0.55465200
C	0.66832500	0.59006000	1.00167400
C	-0.48775900	0.08932000	0.10654900
C	-0.52340500	-1.41977900	0.19085900
C	0.70564700	-2.07939100	-0.37819000
C	1.93100400	-1.57900100	0.43473400
H	0.48396300	0.32639100	2.04985400
H	0.77317900	1.67505900	0.94589400
H	2.79210800	0.23278100	1.25252300
H	-0.78967000	-1.78649300	1.18844300
H	-1.77998300	-1.60145600	-0.37502700
H	0.65078100	-3.16936000	-0.31558800
H	0.83498300	-1.81249200	-1.43060300
H	1.88069900	-1.98577500	1.45030000
H	2.86119400	-1.94091000	-0.01247200
H	-0.23721800	0.37109500	-0.92350100
C	-1.87894800	0.68815300	0.38263600
H	-2.25253300	0.36249300	1.36243100
C	-1.96998600	2.19851100	0.23912600
H	-1.39060600	2.69469000	1.02030000
H	-1.58831100	2.51899200	-0.73312200
O	-2.75132400	0.15033600	-0.63096000
O	-2.89281300	-1.24142500	-0.42807900
H	-3.00973100	2.51877400	0.32704700
O	2.37233200	0.39699500	-0.75771200
O	2.59565800	1.83235500	-0.70003200
H	3.54805700	1.86049800	-0.86446100

P38

C	-2.10408100	-0.13574400	0.59252800
C	-0.66769000	-0.56881800	0.90024200
C	0.36138700	0.09651500	-0.04344800

C	0.13193700	1.57584300	-0.12067600
C	-1.25622700	2.08719100	-0.32256100
C	-2.27329900	1.38514800	0.59920600
H	-0.44818400	-0.29406500	1.93927200
H	-0.61379000	-1.65564700	0.82902000
H	-2.78787600	-0.59330100	1.31917800
H	0.98002400	2.22467200	-0.29914100
H	-1.29708100	3.16895000	-0.16452400
H	-1.56805100	1.91911100	-1.36690700
H	-2.12976300	1.72233300	1.63102600
H	-3.29749000	1.63886700	0.31178500
H	0.20094000	-0.34140700	-1.04425400
C	1.80741100	-0.24293400	0.36269600
H	2.04675600	0.26829300	1.30339800
C	2.11028000	-1.73283800	0.48011400
H	1.60171600	-2.17873800	1.33743000
H	1.79935400	-2.26329800	-0.42446400
H	3.18296200	-1.87374500	0.62264000
O	-2.51486300	-0.54379000	-0.72360000
O	-2.48982800	-1.99635500	-0.77773000
H	-3.43775000	-2.17941700	-0.83008600
O	2.60797000	0.35662300	-0.67210900
O	3.98357400	0.40670000	-0.19898000
H	4.41510500	-0.13420000	-0.87364000

R39

C	0.41832000	1.55351300	0.59876800
C	1.87960700	1.95995800	0.35905300
C	2.86004000	0.92382100	0.91779300
C	2.56420200	-0.47515500	0.37541300
C	1.10041300	-0.88157700	0.57239900
C	0.10606700	0.15877900	0.01650200
H	3.89054700	1.19039500	0.67503400
H	2.05265100	2.07046700	-0.71603400
H	2.07035400	2.93757800	0.81203200
H	0.21416900	1.54293400	1.67862200
H	-0.24098500	2.30806600	0.16238800
H	3.21911100	-1.21486700	0.85257900
H	0.93691100	-1.85469400	0.10751400
H	0.93430900	-1.01167200	1.64829900
H	0.23367400	0.20285900	-1.07125100
H	2.78623200	0.88509800	2.01178200
C	-2.41434100	0.58845400	-0.39925800
H	-2.23730300	1.64286600	-0.16787800

H	-2.27963500	0.48036700	-1.48215100
O	2.76954900	-0.54972200	-1.04675400
O	4.17699000	-0.30779200	-1.31521600
H	4.44824400	-1.19113100	-1.60000700
C	-1.34894300	-0.25024700	0.30189500
H	-1.52810100	-0.30880500	1.37913300
O	-1.54755200	-1.63052300	-0.19025700
O	-1.58744200	-2.52498700	0.77672600
C	-3.85102200	0.21771400	-0.01055800
H	-4.01845700	-0.84362900	-0.21478700
H	-3.97573800	0.34688200	1.07113700
C	-4.89687000	1.05559600	-0.75076400
H	-4.81855800	0.91890600	-1.83366400
H	-5.91052400	0.77429800	-0.45417900
H	-4.77410000	2.12313600	-0.54201800

TS39

C	2.60010400	-0.56227700	-0.47883800
C	1.11876200	-0.78739000	-0.79307000
C	0.18677500	0.21073300	-0.07545600
C	0.63485400	1.65897200	-0.35227000
C	2.11092600	1.87716000	0.01050600
C	3.03118300	0.88751200	-0.71349000
H	0.99282300	-0.68513200	-1.87799900
H	0.84474800	-1.81040900	-0.53400900
H	3.21148000	-1.24393900	-1.08022300
H	0.48468100	1.88473500	-1.41724400
H	0.01670900	2.36525400	0.20868900
H	2.40474300	2.90440600	-0.22591400
H	2.23240000	1.75813300	1.09295800
H	3.01075900	1.07865300	-1.79335900
H	4.06868700	1.00759500	-0.39283400
H	0.25775600	0.02750900	1.00336000
C	-1.27156000	-0.02769700	-0.48519800
H	-1.38617100	0.08096700	-1.57167300
C	-2.30831800	0.86618900	0.24501100
H	-2.16911600	1.90210700	-0.08686200
H	-2.08599100	0.82732300	1.31637300
C	-3.73763500	0.42984500	-0.00568100
H	-4.16477100	0.81512000	-0.93392800
H	-3.48349400	-0.86170500	-0.39720500
C	-4.69272800	0.40488000	1.16068400
H	-4.87925100	1.42047700	1.53930700
H	-5.65962000	-0.02022500	0.88138100
H	-4.28795200	-0.18007300	1.99159200

O	-1.58069200	-1.38532200	-0.14522100
O	-2.79316000	-1.72897000	-0.78077400
O	2.74489000	-0.94973400	0.89842900
O	4.17074300	-1.01372200	1.18705400
H	4.22315800	-0.39636900	1.92849700

P39

C	2.60209700	-0.53244400	-0.45321200
C	1.12248900	-0.84129800	-0.69574000
C	0.16739600	0.17440600	-0.03426800
C	0.54845400	1.60958800	-0.44801400
C	2.02464800	1.91577900	-0.15427600
C	2.96490600	0.90733000	-0.82338100
H	0.96058400	-0.83433800	-1.78093100
H	0.89966500	-1.84858600	-0.34559300
H	3.22166500	-1.23898000	-1.01633500
H	0.35678000	1.73697900	-1.52265200
H	-0.07620800	2.34096400	0.07094000
H	2.26651100	2.93091000	-0.48382800
H	2.18238500	1.89243100	0.92993200
H	2.90394500	1.00493000	-1.91418900
H	4.00569700	1.09589000	-0.54933400
H	0.28057200	0.08864100	1.05340800
C	-1.29695800	-0.16767300	-0.37322200
H	-1.42709500	-0.18086000	-1.46247200
C	-2.33339700	0.77059800	0.27370300
H	-2.11539000	1.79980700	-0.05683100
H	-2.17539100	0.76095400	1.35924700
C	-3.76455500	0.46386900	-0.03791400
H	-4.02645900	0.30314100	-1.08079400
C	-4.85250100	0.85288100	0.90654400
H	-5.00873400	1.94528400	0.91997700
H	-5.81063800	0.40230400	0.63551800
H	-4.61541900	0.56510300	1.93675600
O	2.80805200	-0.79494200	0.94598600
O	4.24379900	-0.77988300	1.18805700
H	4.29766500	-0.08613700	1.85841200
O	-1.48723000	-1.51137200	0.10210400
O	-2.54975000	-2.13643800	-0.65310200
H	-3.33457700	-1.81556700	-0.17691300

R40

C	0.41832000	1.55351300	0.59876800
C	1.87960700	1.95995800	0.35905300
C	2.86004000	0.92382100	0.91779300

C	2.56420200	-0.47515500	0.37541300
C	1.10041300	-0.88157700	0.57239900
C	0.10606700	0.15877900	0.01650200
H	3.89054700	1.19039500	0.67503400
H	2.05265100	2.07046700	-0.71603400
H	2.07035400	2.93757800	0.81203200
H	0.21416900	1.54293400	1.67862200
H	-0.24098500	2.30806600	0.16238800
H	3.21911100	-1.21486700	0.85257900
H	0.93691100	-1.85469400	0.10751400
H	0.93430900	-1.01167200	1.64829900
H	0.23367400	0.20285900	-1.07125100
H	2.78623200	0.88509800	2.01178200
C	-2.41434100	0.58845400	-0.39925800
H	-2.23730300	1.64286600	-0.16787800
H	-2.27963500	0.48036700	-1.48215100
O	2.76954900	-0.54972200	-1.04675400
O	4.17699000	-0.30779200	-1.31521600
H	4.44824400	-1.19113100	-1.60000700
C	-1.34894300	-0.25024700	0.30189500
H	-1.52810100	-0.30880500	1.37913300
O	-1.54755200	-1.63052300	-0.19025700
O	-1.58744200	-2.52498700	0.77672600
C	-3.85102200	0.21771400	-0.01055800
H	-4.01845700	-0.84362900	-0.21478700
H	-3.97573800	0.34688200	1.07113700
C	-4.89687000	1.05559600	-0.75076400
H	-4.81855800	0.91890600	-1.83366400
H	-5.91052400	0.77429800	-0.45417900
H	-4.77410000	2.12313600	-0.54201800

TS40

C	1.80927900	2.25844300	-0.28263100
C	0.40516900	1.93440700	0.26412700
C	0.14622800	0.41696800	0.10234100
C	1.21766400	-0.31207900	0.87990500
C	2.61183300	-0.11394000	0.33965100
C	2.92185500	1.40228000	0.34761900
H	0.32893100	2.21723800	1.32117200
H	-0.34442200	2.51442900	-0.28178500
H	1.80788100	2.10252500	-1.36591100
H	2.03610900	3.31651200	-0.11935400
H	1.15923900	-0.15873500	1.96136700
H	0.64240800	-1.57987500	0.85448600
H	3.35758600	-0.65340600	0.93101200

H	3.06823800	1.69268300	1.39359400
H	3.87281800	1.56462600	-0.16751200
H	0.28166600	0.18776500	-0.96215500
C	-1.24504700	-0.13382300	0.46964200
H	-1.41045000	-0.08971100	1.55419300
C	-2.41589800	0.48896000	-0.28143500
H	-2.41140900	1.56709100	-0.08836000
H	-2.24928500	0.36144600	-1.35773600
O	-1.21601300	-1.52380300	0.07461900
O	-0.31921200	-2.23372000	0.90850000
O	2.73198200	-0.53394500	-1.02492500
O	2.69202400	-1.98343700	-1.04038300
H	1.76122200	-2.15527100	-1.24859800
C	-3.77979100	-0.09466500	0.10948300
H	-3.76654700	-1.17449400	-0.05883400
H	-3.93524100	0.04752400	1.18554800
C	-4.93738100	0.54080000	-0.66458700
H	-5.89636800	0.10959700	-0.36607800
H	-4.99046500	1.62036100	-0.49020500
H	-4.82563400	0.38483100	-1.74202400

P40

C	-1.94034200	-2.11723200	-0.54408800
C	-0.52367100	-1.90758900	0.01896200
C	-0.21416300	-0.39254900	0.13286800
C	-1.27689500	0.24055200	0.97154100
C	-2.68130300	0.09294900	0.51182900
C	-3.02623200	-1.38818500	0.26534100
H	-0.43674100	-2.37423900	1.00831300
H	0.20151900	-2.39970300	-0.63516000
H	-1.96585800	-1.75805300	-1.57738300
H	-2.17338300	-3.18614800	-0.57617900
H	-1.02966800	0.83450400	1.84076900
H	-3.38875300	0.54527800	1.21599100
H	-3.14070500	-1.85609100	1.24907000
H	-3.99547800	-1.45705900	-0.23828700
H	-0.29691200	0.00928700	-0.88769500
C	1.19338100	-0.03127200	0.63576600
H	1.34650300	-0.45176000	1.63892300
C	2.33202400	-0.46279100	-0.28894200
H	2.24531300	-1.53817300	-0.48052100
H	2.19932900	0.04330800	-1.25070700
O	-2.87564900	0.71764300	-0.79203800
O	-2.38374800	2.08439800	-0.71830000
H	-3.21387900	2.57778000	-0.76522800

O	1.26040300	1.38179300	0.92775800
O	0.98782200	2.12907500	-0.28975200
H	0.03021800	2.27852100	-0.21615700
C	3.72496200	-0.15528300	0.27176100
H	3.78625100	0.91254700	0.49737200
H	3.85526800	-0.68011300	1.22661300
C	4.85162500	-0.55206200	-0.68586100
H	4.76739300	-0.01674800	-1.63664600
H	5.83285100	-0.32146700	-0.26212500
H	4.82978900	-1.62418400	-0.90737200

R41

C	0.41832000	1.55351300	0.59876800
C	1.87960700	1.95995800	0.35905300
C	2.86004000	0.92382100	0.91779300
C	2.56420200	-0.47515500	0.37541300
C	1.10041300	-0.88157700	0.57239900
C	0.10606700	0.15877900	0.01650200
H	3.89054700	1.19039500	0.67503400
H	2.05265100	2.07046700	-0.71603400
H	2.07035400	2.93757800	0.81203200
H	0.21416900	1.54293400	1.67862200
H	-0.24098500	2.30806600	0.16238800
H	3.21911100	-1.21486700	0.85257900
H	0.93691100	-1.85469400	0.10751400
H	0.93430900	-1.01167200	1.64829900
H	0.23367400	0.20285900	-1.07125100
H	2.78623200	0.88509800	2.01178200
C	-2.41434100	0.58845400	-0.39925800
H	-2.23730300	1.64286600	-0.16787800
H	-2.27963500	0.48036700	-1.48215100
O	2.76954900	-0.54972200	-1.04675400
O	4.17699000	-0.30779200	-1.31521600
H	4.44824400	-1.19113100	-1.60000700
C	-1.34894300	-0.25024700	0.30189500
H	-1.52810100	-0.30880500	1.37913300
O	-1.54755200	-1.63052300	-0.19025700
O	-1.58744200	-2.52498700	0.77672600
C	-3.85102200	0.21771400	-0.01055800
H	-4.01845700	-0.84362900	-0.21478700
H	-3.97573800	0.34688200	1.07113700
C	-4.89687000	1.05559600	-0.75076400
H	-4.81855800	0.91890600	-1.83366400
H	-5.91052400	0.77429800	-0.45417900

H	-4.77410000	2.12313600	-0.54201800

TS41

C	-2.40779400	-0.83316700	0.58219700
C	-0.92084600	-0.70449700	0.95502400
C	-0.23896800	0.35518300	0.05948700
C	-0.99085500	1.65700100	0.22717900
C	-2.41056200	1.59825900	-0.27236000
C	-3.15369300	0.50807300	0.54690800
H	-0.84431500	-0.42114900	2.01142200
H	-0.45264700	-1.68348400	0.83958100
H	-2.90502200	-1.51305200	1.28545800
H	-0.90412900	2.07296200	1.23728400
H	-0.04536600	2.49217800	-0.35769200
H	-2.92604100	2.55486900	-0.15328400
H	-2.43615000	1.33553900	-1.33344800
H	-3.26554400	0.84841700	1.58183500
H	-4.15970800	0.34776000	0.14880100
H	-0.35794100	0.01905500	-0.97748600
C	1.27292700	0.56905400	0.26882900
H	1.46626500	1.01502800	1.25418800
C	2.14334200	-0.66414200	0.05326500
H	1.80470300	-1.44860100	0.73837500
H	1.97152400	-1.03951500	-0.96224500
O	1.67643500	1.51824100	-0.73958000
O	1.08572300	2.77131500	-0.46199900
O	-2.56984100	-1.36725000	-0.74217300
O	-2.01580600	-2.71101700	-0.75937000
H	-2.82454600	-3.22436800	-0.89105500
C	3.63994900	-0.40679400	0.27075500
H	3.96374000	0.39589500	-0.39657700
H	3.79629100	-0.03986100	1.29232700
C	4.49360900	-1.65598300	0.03823300
H	5.55412200	-1.44693000	0.20154100
H	4.20723000	-2.46777700	0.71485000
H	4.38278500	-2.02519700	-0.98600200

P41

C	2.53205900	0.66317500	0.62320900
C	1.00948400	0.64293700	0.78784300
C	0.33090600	-0.40919700	-0.12014700
C	1.02211000	-1.73470200	-0.01215300
C	2.51309500	-1.79127800	-0.06020300
C	3.16385300	-0.71490000	0.83018800
H	0.78869900	0.42113500	1.83941200
H	0.62975600	1.64291600	0.57645000

H	2.96647800	1.38551000	1.32641900
H	0.44281200	-2.63284400	-0.18617000
H	2.87180700	-2.78253300	0.23283600
H	2.86010000	-1.63585100	-1.09550800
H	3.02945500	-0.97819700	1.88447200
H	4.23961200	-0.65393200	0.64304000
H	0.42932200	-0.04269700	-1.15732200
C	-1.18043500	-0.53648400	0.16451500
H	-1.32269600	-1.02049300	1.13636400
C	-1.94445000	0.79199600	0.11068400
H	-1.59678800	1.42191200	0.93712900
H	-1.67522100	1.31637100	-0.81461900
O	2.92079700	1.04790000	-0.70664100
O	2.45664000	2.40538500	-0.94123800
H	3.30287900	2.87280400	-0.95539200
O	-1.66696200	-1.45812500	-0.83567500
O	-2.68263700	-2.30700000	-0.23491000
H	-3.47599900	-1.98809500	-0.68581400
C	-3.47170000	0.67511000	0.19791900
H	-3.84450100	0.14398300	-0.68505800
H	-3.74809600	0.07121100	1.06899200
C	-4.16006600	2.04118400	0.27663500
H	-3.91663600	2.65721700	-0.59447600
H	-5.24774900	1.93780100	0.31741500
H	-3.84507900	2.59147000	1.16859800

R42

C	-2.31185600	-0.74620200	-0.98249100
C	-3.08724700	0.51509700	-1.37593900
C	-1.05305900	-0.43193200	-0.15927600
O	-1.07553000	-1.20750600	1.10760600
O	-2.04304200	-0.84181400	1.91773600
O	-3.36047800	1.36348700	-0.26811200
O	-4.24614000	0.64806800	0.62992500
H	-4.01990800	0.24594200	-1.88280700
H	-2.50147200	1.16003000	-2.03979300
H	-1.07289000	0.61027700	0.16271800
H	-3.59971000	0.27067500	1.25587500
H	-2.97160300	-1.38320400	-0.39168400
H	-2.05363500	-1.30796200	-1.88522800
C	0.26156200	-0.80227200	-0.83113600
H	0.26173100	-1.88671200	-0.99444200
H	0.25971500	-0.34192500	-1.82698300
C	1.53849200	-0.39229300	-0.07691300

C	2.77496800	-1.06006400	-0.70697200
C	1.73233300	1.13497700	-0.02004200
H	1.45181700	-0.76114100	0.95344000
C	4.07385600	-0.67480300	0.01447600
H	2.84371600	-0.76015900	-1.76210100
H	2.65047200	-2.14852800	-0.70065300
C	3.02851200	1.52585900	0.70541900
H	1.76134400	1.52659700	-1.04660600
H	0.88086500	1.61545500	0.47225900
C	4.25397900	0.84779900	0.07901000
H	4.93073600	-1.13872200	-0.48438800
H	4.05093600	-1.07913200	1.03447600
H	3.14793300	2.61383400	0.69377600
H	2.95216900	1.23272700	1.76021800
H	5.15697600	1.09935700	0.64430800
H	4.40308600	1.23771000	-0.93643800

TS42

C	-3.15206200	-0.89129900	1.20046200
C	-2.00084500	0.11165300	1.37161500
C	-1.37422500	0.51606600	0.04575100
C	-2.37476100	0.92519900	-1.02215000
C	-3.52743800	-0.07973600	-1.17038300
C	-4.18461200	-0.39389300	0.18032500
H	-2.38531200	1.02330400	1.85779000
H	-1.23794700	-0.29429500	2.04373500
H	-2.74296800	-1.85189300	0.86545100
H	-3.62827500	-1.07547100	2.16818200
H	-2.78563900	1.91144600	-0.74743500
H	-1.86185100	1.06513000	-1.97904900
H	-4.26858000	0.30935700	-1.87498600
H	-3.13592800	-1.00647100	-1.60658200
H	-4.67131800	0.51090900	0.56767900
H	-4.97421100	-1.14012600	0.04952700
H	-0.81991700	-0.61049000	-0.38993500
C	-0.10547700	1.35192500	0.13808400
H	-0.14412600	2.00375000	1.02090900
H	-0.01155200	1.99385400	-0.74322400
C	1.17978300	0.49524000	0.23551000
O	1.13806200	-0.45419500	-0.84943200
O	0.10303800	-1.38184900	-0.58069200
H	1.19266400	-0.07296900	1.17134500
C	2.45450100	1.32079200	0.06890900
H	2.33363400	2.23876200	0.65586400
H	2.53449900	1.62438700	-0.98035300

C	3.76881500	0.66535000	0.50839500
H	4.57838200	1.39622600	0.41178100
H	3.72343500	0.33588000	1.55216700
O	4.20003000	-0.41672700	-0.30353900
O	3.49496900	-1.61125800	0.11857500
H	2.71413600	-1.57683500	-0.46125200

P42

C	-0.26696600	1.11627700	-0.96366100
H	-0.27082800	2.21407000	-0.98974100
H	-0.02903900	0.77120600	-1.97601400
C	0.90178500	0.68427300	-0.05284800
H	0.66540800	0.92482200	0.99023800
C	2.23343100	1.32850300	-0.45674400
H	2.12369600	2.41417300	-0.35536700
H	2.41061900	1.13534100	-1.51863500
C	3.46828100	0.90924100	0.34522600
H	4.35652800	1.39828800	-0.06669300
H	3.38901900	1.17028500	1.40253600
O	3.74629600	-0.50094200	0.26508800
O	3.25903800	-1.14588900	1.46892900
H	2.32183700	-1.28243000	1.22521900
O	0.98685000	-0.75912600	0.01623300
O	1.45992400	-1.28201500	-1.25258300
H	2.42079800	-1.29794300	-1.07891400
C	-1.60050400	0.59588000	-0.52576400
C	-2.50576000	1.43439100	0.32633000
C	-2.16439800	-0.65785200	-1.12215900
C	-3.33960400	0.59441400	1.31536200
H	-3.20763500	1.99359300	-0.32154300
H	-1.93400300	2.19712900	0.86755500
C	-2.99761400	-1.46942900	-0.11115900
H	-2.82065300	-0.39626100	-1.97452700
H	-1.36212400	-1.27589000	-1.53355400
C	-4.02181100	-0.58646500	0.61324800
H	-4.08302100	1.22995800	1.80760200
H	-2.67949000	0.20976600	2.10227100
H	-3.50015900	-2.29646100	-0.62296100
H	-2.31983300	-1.91608200	0.62564000
H	-4.58545200	-1.17982400	1.34053400
H	-4.75357400	-0.20536100	-0.11197400

R43

C	-0.87635900	-0.44122400	0.91683000
C	-2.00700300	-0.92204000	-0.00322300

O	0.29477100	1.09952100	-0.62074400
O	-0.19096200	2.14970200	0.00238100
O	-2.30536900	0.01409800	-1.05032300
O	-2.84830200	1.22152400	-0.46157600
H	-1.67295800	-1.78289900	-0.59169200
H	-2.07873500	1.81634900	-0.51091200
H	-1.21705800	0.43928900	1.46669300
H	-0.69082900	-1.22177600	1.66149600
C	0.47448300	-0.10054700	0.26854200
C	1.51058700	0.26968800	1.34149900
C	1.00063000	-1.16111500	-0.70511800
C	2.90001400	0.58073300	0.77006800
H	1.57967000	-0.57791800	2.03292800
H	1.12391200	1.11808000	1.91175500
C	2.40282500	-0.84680400	-1.25052300
H	1.01724000	-2.11701400	-0.16793700
H	0.29492300	-1.26839100	-1.53181900
C	3.40475400	-0.55558100	-0.12757600
H	3.59650100	0.75843600	1.59492300
H	2.85598200	1.51001100	0.19232600
H	2.74473200	-1.68644200	-1.86312900
H	2.34116100	0.02078000	-1.91540000
H	4.38071700	-0.29945200	-0.55070700
H	3.55329700	-1.46033600	0.47684000
C	-3.26073300	-1.30851400	0.77816000
H	-4.03702300	-1.64519500	0.08847600
H	-3.04442600	-2.11761500	1.48124400
H	-3.64340400	-0.45223800	1.33506200

TS43

C	3.05302000	0.64047400	0.58445500
C	1.64926800	0.54975800	1.19775200
C	0.60942500	-0.02485100	0.22397000
C	1.09612400	-1.35172000	-0.38704300
C	2.49896200	-1.24901200	-1.00340600
C	3.51400700	-0.70551800	0.01027800
H	1.66808000	-0.09146400	2.08793100
H	1.30769100	1.53661100	1.52003700
H	3.04265900	1.39011300	-0.21312100
H	3.75902100	0.99484700	1.34201600
H	1.10492000	-2.09601400	0.41710400
H	0.37324500	-1.69639000	-1.13228600
H	2.80826100	-2.23531800	-1.36346600
H	2.46322600	-0.58918000	-1.87549600
H	3.63547900	-1.42928000	0.82750100

H	4.49660700	-0.59885300	-0.45985400
C	-0.76803900	-0.17877400	0.91547000
H	-0.88805100	0.59394600	1.67971300
H	-0.79035600	-1.14352700	1.42844300
C	-1.97111200	-0.12513900	-0.03892600
C	-2.40108800	1.28559000	-0.36001900
H	-1.14169800	1.90632100	-0.57544500
H	-2.93961900	1.44973500	-1.28982600
O	0.49017800	0.83951700	-0.94575500
O	-0.03005100	2.09866300	-0.58462100
H	-2.78269900	1.84629400	0.49320700
H	-1.76234000	-0.68278000	-0.95480200
O	-3.01364900	-0.82740300	0.67385400
O	-4.10198200	-1.07109000	-0.25791200
H	-4.79379800	-0.51740800	0.12863900

P43

C	2.93043000	0.68933900	0.64381700
C	1.60549200	0.27860400	1.29842300
C	0.54046000	-0.13740100	0.26728500
C	1.10283400	-1.21530300	-0.68176400
C	2.43530500	-0.81107700	-1.33028000
C	3.47473200	-0.41076500	-0.27569000
H	1.77046000	-0.56820100	1.97655400
H	1.20615400	1.09976700	1.89663700
H	2.76891600	1.60304000	0.06366000
H	3.66121500	0.93210800	1.42176100
H	1.24336300	-2.12835700	-0.09144800
H	0.36434700	-1.44672900	-1.45507300
H	2.80715900	-1.64153300	-1.93908700
H	2.26171700	0.02967500	-2.00810600
H	3.74196100	-1.29050100	0.32555400
H	4.39566100	-0.07609800	-0.76351000
C	-0.74116300	-0.62012700	0.98862300
H	-0.98222800	0.05083700	1.81906400
H	-0.53874400	-1.59727700	1.43417800
C	-2.00282600	-0.73663300	0.10118100
H	-1.71181500	-1.01188200	-0.91815400
O	0.24362700	0.95416600	-0.63253500
O	-0.20747500	2.10957900	0.11898900
H	-1.16356100	1.91987600	0.15028900
C	-3.02007500	-1.68120400	0.62857100
H	-3.73576500	-1.34158200	1.36584100
H	-3.01467600	-2.72135000	0.32924700
O	-2.53343400	0.61281100	0.05578100

O	-3.50933400	0.69522900	-1.00863600
H	-2.97238000	1.08134700	-1.71720200

R44

C	2.28433000	-1.38460300	-0.62618900
C	0.75156300	-1.35651000	-0.69813000
C	0.12746500	-0.67537600	0.53648100
C	0.74414300	0.71409200	0.79564600
C	2.27168800	0.70849600	0.79274000
C	2.86017500	0.02530300	-0.44893100
H	0.43365900	-0.81903100	-1.59514800
H	0.36222400	-2.37424900	-0.78843700
H	2.60358000	-2.01702300	0.21295000
H	2.68763500	-1.84482700	-1.53309300
H	0.34875800	1.14240200	1.71822400
H	2.63062200	1.73718000	0.88666600
H	2.59436400	0.17594500	1.69546400
H	2.63274400	0.62687600	-1.33549500
H	3.95026800	-0.01086800	-0.36430400
H	0.39638000	-1.26539700	1.42421800
C	-1.41518100	-0.61827700	0.49724800
H	-1.75670900	0.14222200	1.20455700
O	-1.78416500	-0.16457200	-0.81980400
O	-2.92600900	0.71234700	-0.70877900
H	-2.47300600	1.57366300	-0.70099500
O	0.32095500	1.65762200	-0.26129900
O	-0.69147400	2.40969200	0.11358800
C	-2.10695300	-1.94240900	0.80700400
H	-1.88104500	-2.27585200	1.82447000
H	-1.80006800	-2.72285300	0.10709800
H	-3.18724700	-1.81319800	0.71896700

TS44

C	2.21663200	-0.20099300	-1.06260000
C	0.90826500	0.56374800	-0.83749500
C	-0.18755200	-0.35749100	-0.25562300
C	0.30028400	-1.05580200	1.02580800
C	1.59195700	-1.84442100	0.76709100
C	2.69363800	-0.94172800	0.19575900
H	0.56870500	1.02324200	-1.77166800
H	0.49240600	-0.31082200	1.80508500
H	-0.48743200	-1.71620000	1.39576800
H	1.93415500	-2.31480700	1.69410500
H	1.38330900	-2.65965600	0.06208800
H	2.99206400	-0.20813400	0.95183400

H	3.58542800	-1.52986800	-0.04196600
C	-1.56162900	0.32773900	-0.10762900
H	-1.84050700	0.77314600	-1.07042600
C	-1.66724700	1.37554800	0.96776300
H	-0.60219500	2.24607200	0.55041400
H	-2.54825700	2.01084200	0.92735600
H	-1.35242700	1.09800700	1.96993100
O	-2.58333800	-0.63755500	0.23398600
O	-2.78044500	-1.50952200	-0.91836000
H	-3.66915100	-1.23718000	-1.18529500
H	2.97700200	0.50215500	-1.41263400
H	2.04315400	-0.91851500	-1.87285100
H	-0.34901000	-1.12917600	-1.01863400
O	1.24045100	1.64238900	0.06616900
O	0.26166500	2.65110000	-0.02448900

P44

C	1.95559800	-0.56373400	-1.03887000
C	0.84455200	0.44350100	-0.71855800
C	-0.41335200	-0.26451300	-0.18768000
C	-0.10292300	-1.17441400	1.01251100
C	1.00300000	-2.18291300	0.66979900
C	2.26624300	-1.47977200	0.15383400
H	0.59204800	1.02846300	-1.61070000
H	0.20748100	-0.56660400	1.86836000
H	-1.01890500	-1.69302600	1.30623000
H	1.24193900	-2.78988900	1.54877900
H	0.63548800	-2.87693600	-0.09740100
H	2.69983900	-0.88215900	0.96406900
H	3.02464100	-2.21521900	-0.13214100
C	-1.61142400	0.68477400	0.04767600
H	-1.69758800	1.34213400	-0.83698600
C	-1.59038700	1.49855500	1.28972000
H	-2.52911200	1.77701300	1.75024500
H	-0.66113500	1.92124200	1.63923600
O	-2.83812600	-0.07512500	0.12860900
O	-3.10823500	-0.63918900	-1.18337200
H	-3.87034600	-0.10489500	-1.44581400
H	2.84610200	-0.01690300	-1.35980300
H	1.63250300	-1.16414800	-1.89760400
H	-0.73461200	-0.90371600	-1.01828700
O	1.25719700	1.37380200	0.30104200
O	2.25158400	2.25834800	-0.28380500
H	3.02445800	2.03306600	0.25143400

R45

C	-0.91826500	-1.25626900	-0.54744800
C	0.24978100	-2.06634500	0.03612900
C	1.42998500	-1.17912200	0.43910500
C	0.99140100	-0.03237800	1.35636100
C	-0.20278500	0.74864000	0.81546700
C	-1.38535500	-0.13496300	0.40129300
H	-0.07861400	-2.61298300	0.92777700
H	0.59584500	-2.81033900	-0.68588100
H	-0.61391300	-0.81661600	-1.50131800
H	-1.75173900	-1.93066300	-0.75852800
H	0.70816500	-0.44781000	2.32939600
H	1.82413100	0.64805300	1.53241900
H	-0.51690400	1.51196500	1.53315500
H	-1.72433200	-0.60363000	1.33728900
O	1.96050300	-0.70865600	-0.80844000
O	3.11764400	0.11718100	-0.54926100
H	2.74397900	1.00538600	-0.68782000
O	0.18947200	1.50783200	-0.39806800
O	1.20436000	2.31692800	-0.18891900
C	-2.55782900	0.69721300	-0.15229600
H	-2.73212700	1.54219700	0.52464200
H	-2.26801300	1.13306900	-1.11309000
H	2.20421800	-1.76973700	0.94234000
C	-3.86443200	-0.08687200	-0.31043900
H	-4.67184700	0.57214700	-0.63945200
H	-3.77672200	-0.88665000	-1.04974400
H	-4.17297200	-0.54009600	0.63728600

TS45

C	-0.19367800	1.61873700	-0.63690600
C	-0.99373700	0.98454000	0.51548800
C	-0.30819400	-0.29563300	1.03963500
C	1.16584600	-0.05315700	1.37466000
C	1.95957200	0.60835700	0.24216500
C	1.26935300	1.88432700	-0.24847000
H	-0.83536300	-0.66982600	1.92307800
H	-0.95350800	1.68242800	1.36436300
H	-0.20768400	0.95874800	-1.50688200
H	-0.67210300	2.55743900	-0.93548400
H	1.21484600	0.60663000	2.24734300
H	1.63592100	-0.99514300	1.65904000
H	2.97517800	0.82439400	0.59695600
H	1.32388900	2.63300300	0.55042400
H	1.82358600	2.28451200	-1.10154800

C	-2.49880800	0.81699700	0.18966900
H	-3.02577000	0.49919700	1.09632800
H	-2.90172500	1.81141100	-0.05674000
C	-2.83652300	-0.13562600	-0.93055700
H	-2.33809300	0.01808100	-1.88611700
H	-2.24314600	-1.30777300	-0.50942700
O	-0.31836400	-1.37650100	0.07184800
O	-1.56950200	-2.02646800	0.08792900
H	-3.88703900	-0.40498800	-1.02624900
O	2.04546400	-0.19734000	-0.94235500
O	2.47907900	-1.53588600	-0.60725100
H	1.61459900	-1.97979700	-0.57055500

P45

C	1.00007100	-1.25100600	-0.57781200
C	1.43895900	-0.12881000	0.38092000
C	0.23879000	0.74619400	0.79184200
C	-0.91784800	-0.09581000	1.34162500
C	-1.34005500	-1.25554500	0.43120900
C	-0.13792700	-2.10246400	0.00415300
H	0.55070700	1.47907600	1.54785300
H	1.77965000	-0.59292300	1.31771400
H	0.67302700	-0.80769000	-1.52175700
H	1.86120100	-1.88682000	-0.80299500
H	-0.59560900	-0.51469000	2.30186700
H	-1.77666600	0.53987000	1.55541500
H	-2.07605700	-1.87373700	0.96051400
H	0.21504600	-2.65128800	0.88530800
H	-0.46982500	-2.84696200	-0.72433800
C	2.61465000	0.70108500	-0.17672200
H	2.76252100	1.57021400	0.48987500
O	-1.92350200	-0.81810500	-0.80344000
O	-3.06512300	0.02668300	-0.51939200
H	-2.65344600	0.90686800	-0.58148900
O	-0.11535000	1.46477400	-0.40058100
O	-1.27890400	2.29790900	-0.09429900
H	-1.03815900	3.09251200	-0.58885500
C	3.88726300	-0.06427700	-0.29080200
H	4.62314600	0.17923000	-1.04662800
H	4.16624900	-0.78656300	0.46897700
H	2.33783000	1.12660100	-1.14624200

R46

C	-2.23438400	-1.85647400	0.91641700
C	-0.97104400	-1.00940300	1.12538300

C	-0.39066000	-0.53701800	-0.22236500
C	-1.45069800	0.25282900	-1.02880400
C	-2.71698800	-0.59201600	-1.22540500
C	-3.29282800	-1.09875900	0.10449800
H	-1.21638000	-0.13625400	1.73576200
H	-0.21920100	-1.57873600	1.67977300
H	-1.96856000	-2.78537600	0.39430800
H	-2.64339600	-2.15202600	1.88741600
H	-1.04339400	0.54352500	-2.00311000
H	-3.45239200	0.01040500	-1.76394700
H	-2.46011100	-1.44296200	-1.86764900
H	-3.65577500	-0.24456000	0.68484300
H	-4.15752000	-1.74135600	-0.08769100
H	-0.19451500	-1.43577300	-0.82210500
C	0.98593200	0.17092900	-0.10016700
H	1.03343200	1.05082400	-0.74271400
O	-1.89335600	1.44893600	-0.37454500
O	-0.96086700	2.52108300	-0.66635900
H	-0.43659200	2.52709400	0.15699800
O	1.20917700	0.67857100	1.27784700
O	0.64436700	1.83529200	1.53672300
C	2.17281900	-0.75345300	-0.35550400
H	2.02553000	-1.19721700	-1.34655100
H	2.13939400	-1.58222700	0.36220000
C	3.54039800	-0.06227500	-0.30242500
H	3.67910400	0.39182300	0.68268900
H	3.55347800	0.76091300	-1.02674600
C	4.69650600	-1.02216800	-0.59526800
H	5.65829800	-0.50499800	-0.55490200
H	4.72815300	-1.83774300	0.13360600
H	4.60156700	-1.46956900	-1.58967400

TS46

C	2.40995400	-1.78265000	-0.78062100
C	0.98727500	-1.21200200	-0.87967200
C	0.56556200	-0.52891100	0.43795800
C	1.57750000	0.58281800	0.81512900
C	2.99631600	0.00503100	0.91623800
C	3.42577600	-0.70737100	-0.37418500
H	0.94079900	-0.47749200	-1.68773100
H	0.28556800	-2.01031400	-1.13728300
H	2.42374100	-2.59496900	-0.04121500
H	2.69633500	-2.22952200	-1.73784700
H	1.29182600	1.04393900	1.76676900
H	3.68412300	0.81888000	1.15807800

H	3.01740500	-0.69973400	1.75612200
H	3.51519200	0.03221900	-1.17599300
H	4.41715300	-1.15145600	-0.24031100
H	0.65455800	-1.27164900	1.24320300
C	-0.91574800	-0.09205300	0.47179100
H	-1.06555100	0.64015300	1.27209200
C	-1.87197900	-1.27990700	0.66398800
H	-1.55665100	-1.79742500	1.57620200
H	-1.73527800	-1.98695600	-0.16096600
C	-3.37106300	-0.94386100	0.80307400
H	-3.90209400	-1.86048400	1.09891400
H	-3.51616500	-0.23467300	1.62556300
C	-4.00444000	-0.39585100	-0.45053100
H	-5.00036500	0.03191400	-0.34828000
H	-3.21316300	0.68745000	-0.71173100
H	-3.86836200	-0.98116800	-1.36020500
O	-1.18208400	0.60146600	-0.77854500
O	-2.32376800	1.42735500	-0.63871000
O	1.67327500	1.62897400	-0.15883900
O	0.60850900	2.58358800	0.06409000
H	-0.07144400	2.23878100	-0.53836100

P46

C	-2.09812900	-2.04804400	0.76396500
C	-0.81359900	-1.23938300	0.99155100
C	-0.31979500	-0.57265500	-0.30753000
C	-1.42892100	0.28712600	-0.95507700
C	-2.72284600	-0.51365900	-1.14706300
C	-3.20199800	-1.17800500	0.15010700
H	-1.01142500	-0.47473500	1.75047200
H	-0.02759900	-1.87607000	1.40611100
H	-1.88444000	-2.89262800	0.09566900
H	-2.43659800	-2.47920300	1.71118600
H	-1.08922700	0.67263600	-1.91892600
H	-3.48629700	0.15628900	-1.54974100
H	-2.53345900	-1.28413100	-1.90471300
H	-3.49626700	-0.40101000	0.86446700
H	-4.09620900	-1.77693700	-0.04728300
H	-0.13872600	-1.37775700	-1.03283600
C	1.04593900	0.15116900	-0.13442700
H	1.12224900	0.99340600	-0.83107400
C	2.23769400	-0.78885100	-0.35920500
H	2.11174600	-1.24612500	-1.34656500
H	2.19980600	-1.60347100	0.37175400
C	3.60609600	-0.09405700	-0.28957300

H	3.60404700	0.74587800	-1.00844200
O	-1.76257700	1.44369100	-0.15195100
O	-1.10077400	2.60834500	-0.71309700
H	-0.33486600	2.67486300	-0.11251400
O	1.26035400	0.66106200	1.19720100
O	0.53239100	1.89520200	1.40150800
H	-0.34305200	1.57547200	1.67680300
C	4.74555600	-1.00774500	-0.57882400
H	4.63060000	-1.84459000	-1.25882700
H	5.74425200	-0.77684800	-0.22981200
H	3.73032300	0.37319400	0.69144700

R47

C	0.41832000	1.55351300	0.59876800
C	1.87960700	1.95995800	0.35905300
C	2.86004000	0.92382100	0.91779300
C	2.56420200	-0.47515500	0.37541300
C	1.10041300	-0.88157700	0.57239900
C	0.10606700	0.15877900	0.01650200
H	3.89054700	1.19039500	0.67503400
H	2.05265100	2.07046700	-0.71603400
H	2.07035400	2.93757800	0.81203200
H	0.21416900	1.54293400	1.67862200
H	-0.24098500	2.30806600	0.16238800
H	3.21911100	-1.21486700	0.85257900
H	0.93691100	-1.85469400	0.10751400
H	0.93430900	-1.01167200	1.64829900
H	0.23367400	0.20285900	-1.07125100
H	2.78623200	0.88509800	2.01178200
C	-2.41434100	0.58845400	-0.39925800
H	-2.23730300	1.64286600	-0.16787800
H	-2.27963500	0.48036700	-1.48215100
O	2.76954900	-0.54972200	-1.04675400
O	4.17699000	-0.30779200	-1.31521600
H	4.44824400	-1.19113100	-1.60000700
C	-1.34894300	-0.25024700	0.30189500
H	-1.52810100	-0.30880500	1.37913300
O	-1.54755200	-1.63052300	-0.19025700
O	-1.58744200	-2.52498700	0.77672600
C	-3.85102200	0.21771400	-0.01055800
H	-4.01845700	-0.84362900	-0.21478700
H	-3.97573800	0.34688200	1.07113700
C	-4.89687000	1.05559600	-0.75076400
H	-4.81855800	0.91890600	-1.83366400

H	-5.91052400	0.77429800	-0.45417900
H	-4.77410000	2.12313600	-0.54201800

TS47

C	-2.24746200	-1.91329100	-0.33773700
C	-0.76071900	-1.79168800	0.01720100
C	-0.27362300	-0.33037300	-0.04546900
C	-1.13623600	0.55333100	0.87588100
C	-2.63495900	0.42721100	0.57849800
C	-3.11676500	-1.02577100	0.56229700
H	-0.59475900	-2.17669300	1.03350200
H	-0.15911800	-2.40787900	-0.65181000
H	-2.39790400	-1.62588200	-1.38307400
H	-2.56971900	-2.95533800	-0.24807100
H	-0.97342900	0.26795800	1.92377400
H	-0.87001700	1.60657500	0.78377300
H	-3.20525500	1.00737100	1.31520800
H	-3.07855400	-1.39275600	1.59467200
H	-4.16487300	-1.05345500	0.25089900
H	-0.40491200	0.03045400	-1.07294400
C	1.22722200	-0.23089800	0.28784900
H	1.41772000	-0.67322200	1.27399000
C	1.79737200	1.19283700	0.22397200
H	1.22621400	1.82152300	0.91199100
H	1.62088700	1.58807400	-0.78338900
C	3.28949400	1.34763200	0.59013500
H	3.51685500	2.42314300	0.64008600
H	3.46014700	0.96120800	1.60165500
C	4.25246900	0.69028400	-0.36505900
H	5.28962900	0.61963600	-0.04183100
H	3.80464300	-0.63066900	-0.35768000
H	4.13364600	0.93224800	-1.42112500
O	1.87774500	-1.08198600	-0.68747900
O	3.12059300	-1.52535600	-0.18927200
O	-2.95879400	0.93240100	-0.72896800
O	-2.65047300	2.35287900	-0.75640500
H	-3.54453100	2.71855100	-0.79803200

P47

C	2.45635300	-1.67548700	0.08651600
C	0.97191700	-1.77802700	-0.28608300
C	0.24041400	-0.43956700	-0.07546100
C	0.93815600	0.67642100	-0.87698900
C	2.43397500	0.78510300	-0.56191400
C	3.16189300	-0.55570100	-0.68975300
H	0.87700200	-2.07857900	-1.33950500

H	0.48936300	-2.55767800	0.30442000
H	2.54811800	-1.48342500	1.16020700
H	2.95865200	-2.62859300	-0.10688200
H	0.83482400	0.47999700	-1.95291800
H	0.48611200	1.65035300	-0.68457500
H	2.89644400	1.53106900	-1.22117100
H	3.19546400	-0.80650000	-1.75658800
H	4.19684200	-0.43621800	-0.35646000
H	0.30386100	-0.18273600	0.98700800
C	-1.25243700	-0.54297900	-0.44387500
H	-1.34072000	-0.72571000	-1.52436700
C	-2.09450100	0.67643300	-0.05770700
H	-1.62216700	1.58091100	-0.45033600
H	-2.08442600	0.76983400	1.03234000
C	-3.54249800	0.60894200	-0.56587100
H	-3.52615000	0.54284900	-1.66901900
O	2.66182700	1.19590400	0.79790800
O	2.10714100	2.52891300	0.96823600
H	2.92253600	3.03958800	1.06350300
O	-1.84525700	-1.75679000	0.06081000
O	-1.75449900	-1.74295800	1.51728500
H	-2.69233000	-1.70822100	1.74773000
C	-4.38050000	1.76347200	-0.13916700
H	-3.92843300	2.70490100	0.15079500
H	-5.45945400	1.72875100	-0.23038300
H	-4.00587100	-0.33281100	-0.24588900

R48

C	-0.57374700	-0.20470300	0.94049300
C	-1.76705000	-0.56256900	0.04498400
O	0.76573000	1.13156700	-0.65132200
O	0.42199500	2.25198400	-0.05700500
O	-1.95719700	0.38066100	-1.02171000
O	-2.33793800	1.66020400	-0.45706000
H	-1.55287400	-1.47249400	-0.52708200
H	-1.49907700	2.15076800	-0.52353000
H	-0.79290100	0.72638300	1.46841700
H	-0.47889800	-0.98189900	1.70537300
C	0.80216300	-0.05556600	0.27269300
C	1.88717200	0.21198400	1.32741600
C	1.18163400	-1.20209200	-0.67128200
C	3.29963100	0.32793100	0.73974800
H	1.85509200	-0.61694100	2.04372900
H	1.61622300	1.11853000	1.87450300

C	2.60726400	-1.08384400	-1.23357400
H	1.08248800	-2.13574900	-0.10466400
H	0.46093100	-1.24522200	-1.49097800
C	3.64862000	-0.88883900	-0.12587400
H	4.02008600	0.43985100	1.55554500
H	3.36859900	1.23819200	0.13467300
H	2.83452000	-1.97784000	-1.82211900
H	2.64957000	-0.23567500	-1.92460700
H	4.64497400	-0.77055500	-0.56227700
H	3.68771100	-1.78714100	0.50456800
C	-3.05046600	-0.77961700	0.85535300
H	-2.85009900	-1.54811400	1.61081000
H	-3.27837600	0.14407300	1.39386900
C	-4.24491200	-1.18927100	-0.00867900
H	-5.14169100	-1.31583100	0.60308400
H	-4.45153500	-0.43022400	-0.76465500
H	-4.05731100	-2.13592400	-0.52555300

TS48

C	-3.29008400	0.55158700	-0.68583400
C	-1.94483300	0.15567900	-1.30885600
C	-0.86902800	-0.15405600	-0.25612700
C	-1.38409100	-1.17033600	0.77911500
C	-2.72803900	-0.76251400	1.40061900
C	-3.78407400	-0.49585300	0.32038100
H	-2.06905700	-0.73437000	-1.93842800
H	-1.57135600	0.95584700	-1.95269800
H	-3.17452700	1.51475400	-0.17873800
H	-4.02984500	0.69855100	-1.47902600
H	-1.49655300	-2.13168500	0.26531900
H	-0.62952700	-1.31156500	1.55836900
H	-3.06621500	-1.55132400	2.08006200
H	-2.58739100	0.13878100	2.00494600
H	-4.00947300	-1.43238100	-0.20746500
H	-4.72010200	-0.16514400	0.78135900
C	0.43914300	-0.62729800	-0.93453800
H	0.56050100	-0.11939400	-1.89506200
H	0.35047800	-1.69587000	-1.14538700
C	1.71271400	-0.41488400	-0.09932900
C	2.25813900	0.99943700	-0.19040900
H	1.09390400	1.71946200	-0.23941300
O	-0.61025700	1.03150200	0.55298700
O	-0.02678400	2.04461500	-0.23130200
H	2.67257400	1.20913400	-1.18013900
H	1.53966700	-0.69149300	0.94483800

O	2.63781700	-1.37249600	-0.65947000
O	3.78378500	-1.47268600	0.23385000
H	4.49722300	-1.25319300	-0.38032000
C	3.03218100	1.56642700	0.96963400
H	3.96329100	1.00540800	1.11975800
H	3.28820600	2.61605800	0.80712000
H	2.45954800	1.49180800	1.89844800

P48

C	-3.21377700	0.23977300	-0.95905100
C	-1.79836000	-0.20019200	-1.35342700
C	-0.81315300	-0.16571100	-0.17086300
C	-1.38073900	-0.95852700	1.02486100
C	-2.80069200	-0.52287700	1.41553100
C	-3.75623700	-0.58149900	0.21724000
H	-1.82055500	-1.22435100	-1.74664700
H	-1.40891400	0.44206900	-2.14570400
H	-3.18980100	1.29876400	-0.68417100
H	-3.87759100	0.15124100	-1.82495000
H	-1.38700200	-2.01737700	0.74073000
H	-0.70886300	-0.86338700	1.88289800
H	-3.16434400	-1.16284000	2.22606600
H	-2.76723900	0.49845400	1.80566800
H	-3.88472800	-1.62670900	-0.09558300
H	-4.74747800	-0.21951800	0.50812500
C	0.57020300	-0.70116700	-0.61123800
H	0.83282800	-0.28468200	-1.58850200
H	0.49310600	-1.78266600	-0.74809300
C	1.73663700	-0.41200900	0.36090900
H	1.36228900	-0.37097300	1.38790200
O	-0.69995200	1.17323500	0.36344800
O	-0.26438800	2.08802400	-0.67421500
H	0.70170500	2.00213500	-0.57148200
C	2.87254100	-1.37308000	0.26353100
H	3.03549600	-2.05376800	1.09120700
O	2.15842700	0.93760200	-0.00061200
O	3.06124900	1.43376600	1.01490100
H	2.45415100	1.95266500	1.56379700
C	3.85824500	-1.31450600	-0.85108000
H	4.58880600	-0.50729700	-0.69376500
H	4.41752600	-2.24878900	-0.93999500
H	3.37990200	-1.10523700	-1.81416500

R49

C	-2.31185600	-0.74620200	-0.98249100

C	-3.08724700	0.51509700	-1.37593900
C	-1.05305900	-0.43193200	-0.15927600
O	-1.07553000	-1.20750600	1.10760600
O	-2.04304200	-0.84181400	1.91773600
O	-3.36047800	1.36348700	-0.26811200
O	-4.24614000	0.64806800	0.62992500
H	-4.01990800	0.24594200	-1.88280700
H	-2.50147200	1.16003000	-2.03979300
H	-1.07289000	0.61027700	0.16271800
H	-3.59971000	0.27067500	1.25587500
H	-2.97160300	-1.38320400	-0.39168400
H	-2.05363500	-1.30796200	-1.88522800
C	0.26156200	-0.80227200	-0.83113600
H	0.26173100	-1.88671200	-0.99444200
H	0.25971500	-0.34192500	-1.82698300
C	1.53849200	-0.39229300	-0.07691300
C	2.77496800	-1.06006400	-0.70697200
C	1.73233300	1.13497700	-0.02004200
H	1.45181700	-0.76114100	0.95344000
C	4.07385600	-0.67480300	0.01447600
H	2.84371600	-0.76015900	-1.76210100
H	2.65047200	-2.14852800	-0.70065300
C	3.02851200	1.52585900	0.70541900
H	1.76134400	1.52659700	-1.04660600
H	0.88086500	1.61545500	0.47225900
C	4.25397900	0.84779900	0.07901000
H	4.93073600	-1.13872200	-0.48438800
H	4.05093600	-1.07913200	1.03447600
H	3.14793300	2.61383400	0.69377600
H	2.95216900	1.23272700	1.76021800
H	5.15697600	1.09935700	0.64430800
H	4.40308600	1.23771000	-0.93643800

TS49

C	-3.99755200	-0.78357500	-0.53903900
C	-2.70467600	-1.47232000	-0.07888400
C	-1.47378800	-0.54625500	-0.24208300
C	-1.75157700	0.75910000	0.48026100
C	-2.99423500	1.48575000	0.02707200
C	-4.22282200	0.55476100	0.17808700
H	-2.79605600	-1.76307500	0.97553500
H	-2.54219700	-2.39522300	-0.64698200
H	-3.94649400	-0.60926400	-1.62115800
H	-4.85276400	-1.44608500	-0.37181300
H	-1.63693100	0.69212400	1.56716600

H	-0.65026900	1.47180300	0.18528900
H	-3.14254000	2.40554100	0.59897800
H	-2.89547200	1.77141900	-1.02728900
H	-4.40459600	0.37272100	1.24401700
H	-5.11386600	1.05580200	-0.21298700
H	-1.38016000	-0.32060700	-1.31401800
C	-0.17596400	-1.23820700	0.21590100
H	-0.13419700	-2.22627800	-0.25755400
H	-0.21525300	-1.41174600	1.29861800
C	1.14541400	-0.52793800	-0.11449300
H	1.22283500	-0.34009500	-1.19055600
C	2.35327400	-1.32627400	0.38993200
H	2.40777500	-1.22148400	1.47897200
H	2.15834100	-2.38520700	0.18576500
C	3.72378900	-0.99626300	-0.21389200
H	4.44993900	-1.73502700	0.14154300
H	3.69996700	-1.03447000	-1.30861100
O	1.22730400	0.76454200	0.54518500
O	0.45289800	1.71811800	-0.15807600
O	4.28576300	0.24073000	0.19732700
O	3.71370900	1.29513500	-0.61549700
H	2.92337000	1.51828200	-0.09360400

P49

C	3.34658400	1.53667900	-0.24903700
C	2.01858200	1.13730600	0.40955700
C	1.55452000	-0.27451700	-0.02167500
C	2.67843800	-1.25952900	0.09938800
C	4.04617400	-0.89568800	-0.37853700
C	4.45507000	0.52386700	0.06052200
H	2.13916200	1.15227200	1.50097400
H	1.25182600	1.87875700	0.16346800
H	3.20668500	1.60298100	-1.33590000
H	3.64251300	2.53562800	0.08736400
H	2.44080600	-2.30256500	0.28536600
H	4.78200700	-1.63096500	-0.03825300
H	4.07479100	-0.93320400	-1.48366900
H	4.65143300	0.52306800	1.13935400
H	5.38831200	0.81408000	-0.43235800
H	1.26909000	-0.20439300	-1.08687000
C	0.30970400	-0.75831200	0.74439000
H	0.51358500	-0.73009000	1.82150500
H	0.11310300	-1.80338100	0.48576800
C	-0.97136300	0.02878700	0.46799800
H	-0.80376800	1.10411200	0.59567700

C	-2.13658700	-0.41804000	1.35927700
H	-2.25853500	-1.50082000	1.26475400
H	-1.85117800	-0.22968600	2.40033100
C	-3.48422500	0.26614200	1.11711300
H	-4.23922500	-0.16576700	1.78128100
H	-3.44413600	1.34300100	1.29370700
O	-3.99525700	0.06212500	-0.21306300
O	-3.72124400	1.24295200	-1.00882400
H	-2.80768600	1.04848600	-1.29845800
O	-1.30840100	-0.02069600	-0.94233300
O	-1.75680800	-1.35557500	-1.29229800
H	-2.71650200	-1.25211900	-1.14587100

R50

C	2.27339400	-1.73912000	-0.63406600
C	0.79252800	-1.36716800	-0.79100700
C	0.24527200	-0.63906500	0.45337100
C	1.12644500	0.56709000	0.83120400
C	2.61153500	0.22016000	0.92959900
C	3.13054800	-0.50812200	-0.31803900
H	0.66176100	-0.71971100	-1.66216400
H	0.20116300	-2.26729800	-0.97791700
H	2.38383400	-2.47651100	0.17218400
H	2.63166700	-2.22197900	-1.54806600
H	0.76434800	1.04128300	1.74474100
H	3.17846100	1.13600500	1.11862000
H	2.73699100	-0.41916200	1.81180200
H	3.11211400	0.17883900	-1.17079000
H	4.17555200	-0.79407300	-0.16665200
H	0.33132300	-1.32042600	1.31186200
C	-1.24928500	-0.26097100	0.34152500
H	-1.49018900	0.48388800	1.10531300
O	-1.45169500	0.37392400	-0.94031500
O	-2.21725500	1.58602900	-0.76830500
H	-1.49985100	2.24450800	-0.72293400
O	1.00062700	1.61311900	-0.20545700
O	0.24495800	2.61916500	0.18353400
C	-2.19991400	-1.45483900	0.48396700
H	-1.97701000	-1.96363100	1.42973100
H	-1.99126000	-2.17173800	-0.31654900
C	-3.67620100	-1.05381200	0.44104400
H	-3.90946000	-0.53870300	-0.49214900
H	-4.31950700	-1.93434100	0.51696600
H	-3.92895300	-0.37977000	1.26476000

TS50

C	1.20515300	-0.95728200	1.22180900
C	0.38450300	-0.74174900	-0.06311500
C	0.94741700	0.44617000	-0.87789700
C	2.43192200	0.23558600	-1.19148700
C	3.26686000	-0.02586700	0.07069500
C	2.68944800	-1.18373600	0.89664400
H	0.37277600	0.58481500	-1.79666700
H	0.52063800	-1.62293700	-0.70195400
H	1.11229200	-0.08209100	1.87244000
H	0.80573700	-1.81398300	1.77544600
H	2.51204900	-0.61644700	-1.87650700
H	2.80290600	1.11399600	-1.72584900
H	4.30279100	-0.24032100	-0.20952900
H	3.28649600	0.88274300	0.68099600
H	2.79565100	-2.12141900	0.33570400
H	3.25892500	-1.30782700	1.82292900
C	-1.14148700	-0.70625600	0.20454200
H	-1.40626500	-1.65134800	0.70002400
C	-1.63642000	0.45127700	1.05290400
H	-1.15752500	0.48045600	2.03324200
H	-1.04235900	1.53105200	0.44728500
C	-3.10862200	0.77649200	1.07377900
H	-3.31048100	1.62264200	1.73390000
H	-3.47209400	1.03671000	0.07645100
O	0.88132700	1.68535400	-0.13730900
O	-0.41792600	2.22210800	-0.23401000
H	-3.69915500	-0.07859800	1.42787400
O	-1.75527700	-0.73951500	-1.09517400
O	-2.99441100	-1.49765600	-0.98366800
H	-3.62472400	-0.84757800	-1.32174300

P50

C	-2.59494900	-0.03210500	1.05198500
C	-1.17308600	0.52347800	0.88737000
C	-0.22900000	-0.56812000	0.32105000
C	-0.77605800	-1.12429900	-1.00837800
C	-2.20112100	-1.67161300	-0.83788000
C	-3.14767100	-0.61366700	-0.25682400
H	-0.79672600	0.88162000	1.85155300
H	-2.56837000	-0.81067800	1.82375000
H	-3.23703300	0.77067700	1.42240800
H	-0.77630500	-0.32447900	-1.75349800
H	-0.11948000	-1.91265500	-1.38764400
H	-2.57831200	-2.02755900	-1.80181200

H	-2.17648700	-2.54441000	-0.17143700
H	-3.27733400	0.19751100	-0.98017700
H	-4.13858100	-1.04426700	-0.08182400
H	-0.25544300	-1.37879100	1.06236600
O	-1.31177800	1.66251100	0.03003600
O	-0.21385900	2.57466200	0.26994000
H	0.44097900	2.23205300	-0.36617600
C	1.26706400	-0.16605200	0.26393700
H	1.50804600	0.44938900	1.13720200
C	2.19972100	-1.32512100	0.18383500
H	2.04953000	-2.04236400	-0.61676100
C	3.41428800	-1.42777000	1.03894200
H	3.80201400	-2.44846600	1.07941700
H	4.22378700	-0.78272300	0.66158100
H	3.21711400	-1.09271700	2.06361600
O	1.40221500	0.70109300	-0.91012800
O	2.74799600	1.25259200	-0.90799600
H	3.13088900	0.78259000	-1.66159500

R51

C	-0.42916700	-1.35505100	-0.50081200
C	0.83614600	-2.07791200	-0.01322600
C	1.95897100	-1.10456100	0.35318800
C	1.47986100	-0.02826700	1.33324800
C	0.19407300	0.66256100	0.88808300
C	-0.93268600	-0.30666200	0.51106200
H	0.60893400	-2.67762300	0.87584600
H	1.20030500	-2.76707200	-0.77941600
H	-0.22093800	-0.86270500	-1.45476200
H	-1.21340000	-2.09269900	-0.68734100
H	1.29055100	-0.49595100	2.30537300
H	2.26096100	0.71599700	1.48623100
H	-0.14018700	1.37439300	1.64828100
H	-1.17432200	-0.83011600	1.44809500
O	2.37316400	-0.55285100	-0.90505600
O	3.46908000	0.36231300	-0.68257900
H	3.01348900	1.21843100	-0.76803500
O	0.44844400	1.48826400	-0.31870100
O	1.40179700	2.37550000	-0.13901800
C	-2.20073100	0.43883000	0.05396900
H	-2.39923800	1.25408800	0.76184700
H	-2.01124200	0.91719400	-0.91272700
H	2.80881700	-1.64086500	0.79101400
C	-3.45635000	-0.43695100	-0.04286100

H	-3.30681400	-1.22570300	-0.78719600
H	-3.61736300	-0.94589000	0.91578700
C	-4.70798300	0.36595000	-0.40998900
H	-5.58883200	-0.27812300	-0.47556700
H	-4.91578700	1.13910500	0.33616900
H	-4.58762100	0.86409500	-1.37674900

TS51

C	0.19222500	1.62929700	-0.61197900
C	-0.52324600	1.07040400	0.63176000
C	0.14162500	-0.23266100	1.12616900
C	1.65247400	-0.06446300	1.30859600
C	2.36461300	0.52236900	0.08447000
C	1.69840200	1.82247300	-0.37539400
H	-0.31520200	-0.55287500	2.06816100
H	-0.35910700	1.78653200	1.45020800
H	0.05774700	0.94951500	-1.45596500
H	-0.26278900	2.58544500	-0.89137200
H	1.82278700	0.61392800	2.15124000
H	2.09774000	-1.02357200	1.57505400
H	3.42044800	0.68985100	0.33203500
H	1.87114700	2.58660800	0.39144800
H	2.18654600	2.16962000	-1.28992000
C	-2.05949800	0.98208900	0.46297900
H	-2.50771200	0.71534700	1.42802100
H	-2.43229400	1.99192700	0.23013800
C	-2.57515200	0.02222800	-0.58911000
H	-2.11525100	0.13341500	-1.57293200
H	-1.99778300	-1.13493100	-0.20027300
O	-0.02274600	-1.33487500	0.19885100
O	-1.30048600	-1.90705300	0.35266600
O	2.28995400	-0.31605300	-1.07805000
O	2.67520300	-1.66998300	-0.74579500
H	1.79382100	-2.05806400	-0.60941500
C	-4.06238000	-0.22041500	-0.62463900
H	-4.59650000	0.69670100	-0.91259900
H	-4.32966400	-0.99366000	-1.34844600
H	-4.44371400	-0.51999700	0.35612500

P51

C	0.57802200	-1.31085400	-0.45569400
C	0.97990400	-0.23016100	0.56604900
C	-0.21043900	0.69456400	0.88595500
C	-1.45253100	-0.09770100	1.30627600
C	-1.84419100	-1.21636400	0.33240300
C	-0.64990400	-2.11407900	-0.00289800

H	0.06511800	1.39574500	1.68485800
H	1.20750300	-0.72920600	1.51920400
H	0.36374300	-0.83374100	-1.41547200
H	1.42410100	-1.98541400	-0.61510400
H	-1.24240300	-0.55208400	2.28131700
H	-2.29598300	0.57585400	1.45554200
H	-2.65541100	-1.80718400	0.77611300
H	-0.40860200	-2.69902600	0.89257300
H	-0.94805700	-2.82510800	-0.77800300
C	2.23858500	0.55088800	0.14223800
H	2.36221100	1.40645100	0.83292100
O	-2.28829200	-0.72533400	-0.93972500
O	-3.40799500	0.17158100	-0.74120500
H	-2.94880700	1.03015000	-0.74182700
O	-0.41862100	1.45427800	-0.31568100
O	-1.56289400	2.33957000	-0.09445100
H	-1.23565000	3.13392700	-0.53687600
C	3.48550300	-0.26943100	0.12716400
H	3.58360900	-1.06717100	0.85895600
H	2.07491800	1.00455400	-0.84276200
C	4.70322100	0.17726700	-0.60651500
H	5.20957200	1.01590800	-0.09812600
H	5.43954800	-0.62489900	-0.70132500
H	4.45799300	0.53570500	-1.61336300

R52

C	-0.05906900	-1.31427000	-0.43350300
C	1.19135100	-2.08106800	0.02439800
C	2.36550900	-1.15069500	0.33594900
C	1.96489100	-0.03932900	1.31229700
C	0.69499900	0.69804800	0.89688400
C	-0.48353500	-0.22825500	0.57511300
H	0.96983300	-2.65531500	0.93148400
H	1.50007000	-2.79776500	-0.74084800
H	0.13556100	-0.84792200	-1.40334000
H	-0.87870300	-2.02193700	-0.57898800
H	1.78860300	-0.48285200	2.29814900
H	2.78107300	0.67348900	1.42656200
H	-0.71561300	-0.72513900	1.52886300
O	2.76042200	-0.63678700	-0.94432000
O	3.90047300	0.23464800	-0.77266400
H	3.47859800	1.10798700	-0.85724800
O	0.94423200	1.49121200	-0.33257900
O	1.93772100	2.34169400	-0.19900900

H	3.20631800	-1.71526600	0.75519900
H	0.41702700	1.43644300	1.65429600
C	-1.73428600	0.56200400	0.14559200
H	-1.88319100	1.38744800	0.85360000
H	-1.55053300	1.02726500	-0.82845700
C	-3.02256600	-0.26805000	0.08544900
H	-2.92426900	-1.06184700	-0.66350300
H	-3.17620000	-0.77278300	1.04875300
C	-4.26233100	0.57141400	-0.24569100
H	-4.37617400	1.36002300	0.50789400
H	-4.10356000	1.08551900	-1.20106300
C	-5.54923500	-0.25509700	-0.31841500
H	-5.75167500	-0.75773900	0.63261700
H	-6.41336500	0.37250600	-0.55206900
H	-5.48095000	-1.02647600	-1.09176900

TS52

C	0.64191400	1.56164300	-0.80260600
C	0.02020400	1.28664900	0.57896000
C	0.59445200	-0.00161700	1.20794500
C	2.12560600	0.00395100	1.21225200
C	2.74998400	0.31099100	-0.15379700
C	2.17730800	1.60061500	-0.74897000
H	0.21844300	-0.12028000	2.22929400
H	0.35543500	2.08945000	1.25188000
H	0.33875700	0.78544500	-1.50838900
H	0.26215100	2.51425400	-1.18686100
H	2.46372000	0.77322300	1.91451600
H	2.49384700	-0.95272100	1.58419400
H	3.83913500	0.38203500	-0.04040800
H	2.51833400	2.44284900	-0.13565100
H	2.59026300	1.74547000	-1.75080400
C	-1.52605800	1.36795700	0.57923500
H	-1.88373900	1.30406000	1.61413400
H	-1.81016700	2.37161600	0.22642400
C	-2.26091300	0.33615400	-0.25074800
H	-1.91523100	0.24921000	-1.28377000
H	-1.76606400	-0.81419700	0.25627800
O	0.20764600	-1.20038400	0.49010800
O	-1.09906000	-1.57930500	0.85513900
O	2.45297800	-0.67464500	-1.15402000
O	2.72684200	-2.00157800	-0.64789000
H	1.83072200	-2.25290900	-0.36534600
C	-3.76114700	0.26010500	-0.09728800
H	-4.19006000	1.22939100	-0.39923600

H	-4.01362400	0.14676700	0.96390500
C	-4.41203100	-0.86026200	-0.91489900
H	-4.02673600	-1.83907200	-0.61728100
H	-5.49595200	-0.86932700	-0.77692700
H	-4.21239500	-0.73510700	-1.98315100

P52

C	0.23242200	-1.24562800	-0.37929000
C	0.53688000	-0.11335900	0.62004200
C	-0.71643000	0.74583900	0.87712800
C	-1.91884200	-0.10920500	1.28775600
C	-2.21361300	-1.27739100	0.33812200
C	-0.95782000	-2.10968500	0.06171900
H	-0.50756400	1.48643000	1.66065000
H	0.76366900	-0.56967200	1.59453200
H	0.02012900	-0.81040800	-1.35921200
H	1.12270700	-1.87089600	-0.49263200
H	-1.70927300	-0.52164800	2.28135000
H	-2.80625300	0.51413400	1.39359800
H	-2.99986200	-1.90417600	0.77746400
H	-0.70822300	-2.65539400	0.97946900
H	-1.18980700	-2.85774400	-0.70107100
C	1.75781500	0.73251300	0.21245500
H	1.79541500	1.62261300	0.86874400
O	-2.65013600	-0.85123300	-0.95977000
O	-3.82739400	-0.01940700	-0.81843500
H	-3.42090600	0.86536700	-0.82909300
O	-0.93450700	1.45486600	-0.35315600
O	-2.13650900	2.27440700	-0.19120700
H	-1.84882000	3.06990800	-0.65838700
C	3.05722700	0.00163900	0.28421200
H	3.16443800	-0.78875000	1.02458100
H	1.60626200	1.13489200	-0.79675200
C	4.29439500	0.54068400	-0.35370900
H	4.62894300	1.44619800	0.18500000
H	4.06047700	0.89173400	-1.36834300
C	5.45491500	-0.46006200	-0.40417500
H	6.34164100	-0.01654000	-0.86427800
H	5.73150500	-0.79006900	0.60168400
H	5.18218900	-1.34710300	-0.98230900

R53

C	1.01519200	-0.50567700	0.99341500
C	2.06115700	-1.18356400	0.10092400
O	3.37170700	-0.52938500	0.20080400

O	3.36914200	0.68024300	-0.31626500
O	0.04235900	0.56823500	-0.97198300
O	0.69510700	1.82472000	-0.65936300
H	1.78217300	-1.14100200	-0.95204900
H	2.28508200	-2.20944200	0.39081800
H	1.63289100	1.56309500	-0.72371600
H	1.45057000	0.41277500	1.39305400
H	0.77183600	-1.13789700	1.85051900
C	-0.28552800	-0.13346900	0.24871200
C	-1.03972200	-1.37769100	-0.26103200
C	-1.19437300	0.72282100	1.14765500
C	-2.38415200	-1.03689900	-0.92090600
H	-1.20766600	-2.03274100	0.60223200
H	-0.40742900	-1.93097100	-0.96273200
C	-2.53099100	1.06552500	0.47750900
H	-1.37495300	0.16639200	2.07583500
H	-0.65758700	1.63485900	1.41555000
C	-3.26848700	-0.19450300	0.00694000
H	-2.89519200	-1.96413000	-1.19914200
H	-2.19732200	-0.48506700	-1.84665300
H	-3.15321200	1.63322500	1.17639500
H	-2.33943300	1.71840900	-0.37958900
H	-4.19657900	0.07820700	-0.50498800
H	-3.55828000	-0.79547000	0.87961800

TS53

C	2.66164400	0.37079600	0.27490200
C	1.39915100	0.86510900	0.99464600
C	0.10652900	0.37277600	0.29872100
C	0.18641500	-1.14107100	0.16192200
C	1.39442300	-1.67022300	-0.57012500
C	2.68014900	-1.15658300	0.12626200
H	1.38605100	0.50178600	2.02865900
H	1.37936900	1.95712300	1.03502000
H	2.71009900	0.83596900	-0.71398100
H	3.54732300	0.70347100	0.82559100
H	-0.02553600	-1.63678900	1.11209800
H	-1.00318200	-1.46785400	-0.47047200
H	1.38271600	-2.76328000	-0.58574100
H	1.38733500	-1.32223500	-1.60652300
H	2.76067700	-1.62174300	1.11654600
H	3.55707900	-1.47641400	-0.44543100
C	-1.15700500	0.84342700	1.04589200
H	-1.10663200	1.93301100	1.13655300
H	-1.13334000	0.43586500	2.06150300

C	-2.49889000	0.48872100	0.40027200
H	-2.57963400	0.88715600	-0.61414200
O	-2.75840600	-0.91983700	0.35524800
O	-2.11325300	-1.46904600	-0.77510600
H	-3.31524000	0.89004100	1.00764400
O	0.08655800	0.82974400	-1.07509400
O	0.16097200	2.28755100	-1.08264100
H	-0.58855100	2.47888900	-1.66152900

P53

C	2.69886900	1.00998100	0.18051400
C	1.41630400	0.84173400	1.00325100
C	0.39226500	-0.07849200	0.31140300
C	1.04314400	-1.33819800	-0.17756700
C	2.37497900	-1.28438500	-0.84786700
C	3.35473100	-0.34225600	-0.11959400
H	1.65580900	0.41437600	1.98481300
H	0.93721500	1.80836800	1.17341600
H	2.45248500	1.51627700	-0.75816600
H	3.39764400	1.65794000	0.71893500
H	0.45245500	-2.24668100	-0.22937500
H	2.79695300	-2.29018300	-0.93748600
H	2.24122500	-0.91544800	-1.87879400
H	3.67073400	-0.81078600	0.82054900
H	4.25587300	-0.20745600	-0.72553700
C	-0.80372300	-0.37002800	1.24477000
H	-1.08601900	0.54127500	1.78019300
H	-0.47434700	-1.08553700	2.00351300
C	-2.04876600	-0.92602200	0.55429500
H	-2.65122800	-1.52802200	1.24331100
H	-1.80302500	-1.52388400	-0.32597000
O	-0.06297800	0.53653200	-0.93411700
O	-0.70033000	1.81853100	-0.65409700
H	-1.62725600	1.52434400	-0.59289800
O	-2.83146500	0.20620000	0.13674500
O	-3.91941600	-0.28887300	-0.68232600
H	-4.67182500	-0.10771300	-0.10137200

R54

C	-0.89171200	0.25014500	0.90215200
C	-2.00765800	0.70041400	-0.05316700
O	-3.19496600	-0.18142600	0.09845900
O	-2.97824800	-1.41342400	-0.30108900
O	0.29006700	-0.84885000	-0.93365200
O	-0.17098300	-2.15502900	-0.50651300

H	-1.69706700	0.55712000	-1.08934600
H	-1.13634100	-2.04477500	-0.59890200
H	-1.19559700	-0.68693200	1.37292100
H	-0.76519000	0.97977400	1.70680800
C	0.47116700	0.01127200	0.21464700
C	1.04981000	1.29621500	-0.41028600
C	1.47012900	-0.60547900	1.20998700
C	2.44953900	1.09494500	-1.01044500
H	1.09306300	2.05399400	0.38122500
H	0.36630100	1.67318000	-1.17752100
C	2.86158400	-0.81162400	0.59840000
H	1.54025300	0.06210800	2.07783900
H	1.06510000	-1.55553300	1.56352900
C	3.42011800	0.49094900	0.01198500
H	2.82702400	2.05538000	-1.37589300
H	2.37327500	0.43132600	-1.87653900
H	3.53908700	-1.20832200	1.36100900
H	2.79291400	-1.56760800	-0.18983600
H	4.39311600	0.31043500	-0.45551300
H	3.59208300	1.21215100	0.82258900
C	-2.54555700	2.09920500	0.18424800
H	-2.92542100	2.19988200	1.20403100
H	-1.74329000	2.82690400	0.04507400
H	-3.35079100	2.33449200	-0.51424300

TS54

C	2.86661000	-0.67167100	-0.43099400
C	1.48319000	-1.04784300	-0.97917700
C	0.34137600	-0.34249000	-0.20524200
C	0.62063200	1.15291100	-0.20551700
C	1.95653200	1.57185900	0.35593200
C	3.08749500	0.84684800	-0.41642900
H	1.40361000	-0.76197300	-2.03430500
H	1.32607000	-2.12801300	-0.92376600
H	2.96398800	-1.06598000	0.58472900
H	3.64041200	-1.15636100	-1.03493500
H	0.36537900	1.60469700	-1.16674600
H	-0.44535800	1.67128500	0.51385700
H	2.07949800	2.65547300	0.27939600
H	2.01954800	1.30362200	1.41395900
H	3.11674900	1.22442500	-1.44606500
H	4.05346400	1.09045800	0.03718600
C	-1.03953500	-0.70678800	-0.78632900
H	-1.11498600	-1.79819800	-0.78838200
H	-1.07424900	-0.38115600	-1.83148800

C	-2.27268700	-0.14952500	-0.05981300
H	-2.24820600	-0.42894800	0.99719600
O	-2.31836700	1.29371000	-0.10906800
O	-1.50961700	1.82885200	0.91538800
O	0.41555200	-0.69315700	1.19845000
O	0.31151100	-2.14324300	1.32508900
H	-0.40288600	-2.19276400	1.97371400
C	-3.57332600	-0.61135100	-0.70647000
H	-3.63322100	-1.70237000	-0.71029400
H	-4.42957400	-0.21461500	-0.15859900
H	-3.63460300	-0.26058400	-1.74009500

P54

C	3.01093900	0.70398100	0.56925500
C	1.66247100	0.40999400	1.23639600
C	0.60294600	-0.07856500	0.22874700
C	1.15898000	-1.15485200	-0.65553400
C	2.52982500	-1.01745100	-1.22818300
C	3.54725200	-0.51975600	-0.18218000
H	1.78716700	-0.36042700	2.00751400
H	1.27202800	1.30504600	1.72533300
H	2.88573400	1.53817300	-0.12832000
H	3.73153000	1.02765200	1.32701300
H	0.48937400	-1.91647700	-1.04055100
H	2.85653700	-1.96456700	-1.66835700
H	2.49899200	-0.28820000	-2.05504100
H	3.75080000	-1.32669200	0.53255700
H	4.49633700	-0.28416000	-0.67338000
C	-0.68087800	-0.53021600	0.95829600
H	-0.92052300	0.18165400	1.75441100
H	-0.47063000	-1.48409800	1.44984600
C	-1.92413600	-0.69034700	0.07329500
H	-1.64091900	-0.89925900	-0.96146900
O	0.30594400	0.98533900	-0.72851700
O	-0.21353000	2.15450900	-0.02881900
H	-1.16691400	1.95814200	-0.08048700
O	-2.54340600	0.62249800	0.07900800
O	-3.52477900	0.66818100	-0.99029300
H	-4.33691400	0.77845700	-0.47683100
C	-2.90429400	-1.73827800	0.58442900
H	-3.17668300	-1.53884700	1.62484300
H	-3.81015900	-1.74282100	-0.02453500
H	-2.45821900	-2.73485400	0.52929100

R55

C	0.57221800	0.15048700	0.99770900
C	1.80066000	-0.18019600	0.13656700
O	2.79715900	0.91717100	0.24434800
O	2.40438600	2.03204300	-0.32851100
O	-0.68263300	0.82425400	-0.98989300
O	-0.47383500	2.22800800	-0.69481100
H	1.52943500	-0.19666000	-0.92113000
H	0.49913600	2.28159800	-0.75216500
H	0.67841200	1.16838100	1.37785100
H	0.53347100	-0.50774300	1.87027900
C	-0.77196500	0.06873800	0.23982300
C	-1.08283300	-1.35654500	-0.25866200
C	-1.91592600	0.59581500	1.12453400
C	-2.46133500	-1.47260100	-0.92665500
H	-1.03663000	-2.02413100	0.61006900
H	-0.30291300	-1.68192900	-0.95418200
C	-3.28663900	0.48444300	0.44519300
H	-1.91546700	0.02013600	2.05853900
H	-1.70272200	1.63458800	1.38376600
C	-3.57649500	-0.95058400	-0.01230100
H	-2.64489500	-2.51763600	-1.19631500
H	-2.45521300	-0.89880300	-1.85782600
H	-4.06356700	0.82952300	1.13460700
H	-3.30829200	1.15510100	-0.41927000
H	-4.53929600	-0.99666400	-0.53074100
H	-3.66356800	-1.60359200	0.86678600
C	2.56003600	-1.43737400	0.53945700
H	2.93306300	-1.30290400	1.56108600
H	1.83149500	-2.25313800	0.58304100
C	3.70685600	-1.80471600	-0.40574100
H	4.45021600	-1.00656500	-0.45245200
H	4.20987300	-2.71439800	-0.06964200
H	3.33939100	-1.98176800	-1.42091100

TS55

C	3.22052700	-0.70402200	-0.40923300
C	1.83840400	-1.05699000	-0.97567900
C	0.69736700	-0.33927000	-0.21186300
C	0.99845800	1.15195900	-0.20074300
C	2.33374800	1.54828700	0.37846500
C	3.46317700	0.81099100	-0.38439800
H	1.77565200	-0.76451900	-2.03012200
H	1.66487500	-2.13499800	-0.92791500
H	3.30013800	-1.10472100	0.60553800
H	3.99426700	-1.19699000	-1.00652600

H	0.76132100	1.61235800	-1.16253100
H	-0.06691400	1.68439600	0.50768500
H	2.47331700	2.63039300	0.30923700
H	2.38039700	1.27357100	1.43566500
H	3.50988300	1.19316200	-1.41169400
H	4.42713800	1.03847200	0.08168600
C	-0.68148600	-0.68315900	-0.81070100
H	-0.77123600	-1.77340600	-0.81395100
H	-0.69844000	-0.35667600	-1.85593900
C	-1.91341000	-0.10875000	-0.09685200
H	-1.90367800	-0.38643800	0.96179900
O	-1.93129000	1.33538700	-0.14985100
O	-1.13638900	1.86160000	0.88977600
O	0.74994000	-0.69760500	1.19112200
O	0.62599200	-2.14651500	1.31028300
H	-0.10466900	-2.19036200	1.94097300
C	-3.22101500	-0.55777000	-0.75262300
H	-3.19088900	-1.64716100	-0.86117100
H	-3.25579100	-0.14499300	-1.76721500
C	-4.46918200	-0.13799600	0.02728600
H	-4.50060400	0.94565500	0.15038700
H	-5.37832500	-0.45075300	-0.49251600
H	-4.48198300	-0.58960200	1.02401600

P55

C	3.38745900	0.40784000	0.56447800
C	2.02746100	0.24364100	1.25220700
C	0.89691000	-0.05819700	0.24821000
C	1.29979500	-1.15089100	-0.69723700
C	2.66706900	-1.15724900	-1.29510500
C	3.75771200	-0.83396700	-0.25363400
H	2.07303700	-0.57728600	1.97843900
H	1.75992700	1.15141500	1.79726800
H	3.34772000	1.28305400	-0.09157200
H	4.15705600	0.60768400	1.31681300
H	0.53543400	-1.80803100	-1.09795900
H	2.86636200	-2.11701000	-1.78178500
H	2.71177000	-0.39435900	-2.09037900
H	3.87700000	-1.69218900	0.41914800
H	4.71822400	-0.69055200	-0.75806700
C	-0.41958900	-0.38074700	0.98618900
H	-0.56446700	0.33551200	1.80103000
H	-0.30975200	-1.36250400	1.45555100
C	-1.68809000	-0.37685100	0.12179700
H	-1.45659800	-0.61898700	-0.91784600

O	0.71904900	1.08138000	-0.64925700
O	0.35559700	2.26587400	0.11854900
H	-0.61497600	2.18083600	0.08835400
O	-2.14509500	1.00498500	0.12023800
O	-2.80508000	1.28406700	-1.14293600
H	-3.72366800	1.36891100	-0.85334000
C	-2.76129500	-1.31880400	0.67716900
H	-2.89957600	-1.09934200	1.74214100
H	-2.35146600	-2.33418200	0.62272700
C	-4.11761500	-1.28699300	-0.03369900
H	-4.76547400	-2.08024400	0.34805300
H	-4.64139700	-0.34272100	0.13901300
H	-4.01383500	-1.42714600	-1.11257800

R56

C	1.25881000	0.67051300	-0.29331200
C	0.18923200	-0.05456400	0.54206300
C	2.64849300	0.62488700	0.36131100
O	3.65724800	0.12984400	-0.61017300
O	3.46431200	-1.12227800	-0.95697300
O	0.64230900	-1.37424600	0.88508500
O	0.78845100	-2.15196800	-0.33319000
H	0.12321300	0.40376200	1.53880300
H	2.66042400	-0.11386400	1.16436200
H	1.73970700	-2.02779800	-0.51204500
H	1.30915100	0.18933200	-1.27214300
H	0.96387200	1.70761000	-0.46175600
C	3.18582600	1.96463700	0.82700600
H	4.17675000	1.85861100	1.27225900
H	3.24800000	2.66943200	-0.00587700
H	2.51348000	2.38301600	1.58001700
C	-1.21329700	-0.06834300	-0.09330000
C	-2.18607800	-0.91985100	0.74527300
C	-1.77958600	1.35247400	-0.28959500
H	-1.11407400	-0.54072400	-1.07822200
C	-3.59726500	-0.94366700	0.14341500
H	-2.23476100	-0.50576500	1.76240900
H	-1.79398500	-1.93347300	0.84055300
C	-3.19710200	1.33136400	-0.88298700
H	-1.80335500	1.86665200	0.68179700
H	-1.13029300	1.94462600	-0.94114200
C	-4.15533100	0.47177000	-0.04961400
H	-4.26323200	-1.53138900	0.78328200
H	-3.56728900	-1.45653500	-0.82649000

H	-3.57710600	2.35463500	-0.96806400
H	-3.14986100	0.93121600	-1.90385400
H	-5.14032900	0.43403900	-0.52595600
H	-4.30108600	0.94048500	0.93250400

TS56

C	2.48067100	-0.44386200	-1.32500000
C	1.41359400	0.59166200	-0.93682300
C	0.64797300	0.18113300	0.30769000
C	1.52666700	-0.23625500	1.47601800
C	2.60542700	-1.25390900	1.07239100
C	3.40559500	-0.77321200	-0.14572200
H	1.90295200	1.55802700	-0.73867800
H	0.71181000	0.75021400	-1.75992800
H	1.97881400	-1.35719800	-1.66242500
H	3.06304800	-0.07065500	-2.17301000
H	2.01261300	0.67023200	1.87371900
H	0.91540800	-0.63399400	2.29394400
H	3.27251100	-1.43866200	1.92001000
H	2.12658300	-2.21128800	0.83581200
H	3.98017200	0.12169100	0.12778800
H	4.13514800	-1.53440200	-0.43914800
H	0.07099500	-0.95902200	-0.10364500
C	-0.56937200	1.03382700	0.71605400
H	-0.31142700	1.57163500	1.63763600
C	-1.84355500	0.21301000	0.99993500
H	-1.57700900	-0.58491400	1.69818100
H	-2.56173900	0.86192200	1.50810500
C	-2.55051100	-0.39097100	-0.22526500
O	-1.61306100	-0.92083600	-1.19167900
O	-0.69410400	-1.76756100	-0.53872500
H	-3.02017300	0.41129700	-0.80061400
O	-0.96342200	2.01943700	-0.24244600
O	-0.06645600	3.15805400	-0.10117900
H	0.26873200	3.22341600	-1.00575500
C	-3.57854600	-1.44680300	0.16202400
H	-4.31383100	-1.03805900	0.86123300
H	-4.10575800	-1.80503100	-0.72439300
H	-3.08017400	-2.29568700	0.63558900

P56

C	3.66807200	0.52360800	-0.84450300
C	2.26335800	1.09080300	-0.56695500
C	1.27228200	0.03439500	-0.17055400
C	1.72751000	-0.97967100	0.83850100
C	3.14096700	-1.52041900	0.54200900

C	4.14129000	-0.38531800	0.29536600
H	2.33694700	1.82935300	0.24982500
H	1.89745000	1.64994500	-1.43416100
H	3.64700500	-0.05206700	-1.77780900
H	4.37319000	1.34640300	-0.99729400
H	1.74262100	-0.50416300	1.83600600
H	1.02350700	-1.81185400	0.91815400
H	3.47200300	-2.15196100	1.37226500
H	3.09877900	-2.16207400	-0.34646900
H	4.25252500	0.20755500	1.21263300
H	5.12975200	-0.79654000	0.06709900
C	-0.17343600	0.32088500	-0.42167700
O	-0.59904200	1.30221900	0.60814800
O	-1.26653400	2.42498900	-0.02488100
H	-2.16750700	2.05686900	-0.11606300
C	-1.10803000	-0.89306500	-0.39469900
H	-0.72129900	-1.63504100	-1.10161100
H	-1.08221700	-1.36038400	0.59242100
C	-2.57095100	-0.61959900	-0.76780400
H	-2.62057900	-0.18257000	-1.77259900
H	-0.27589000	0.85939500	-1.36785800
O	-3.15779800	0.44696500	0.01605100
O	-3.03534900	0.13411300	1.42741500
H	-2.17161500	0.55237500	1.61239700
C	-3.44152300	-1.86810400	-0.69732800
H	-4.46309800	-1.63038100	-0.99937500
H	-3.05476700	-2.64802000	-1.35880500
H	-3.46425600	-2.25421600	0.32324400

R57

C	2.42172000	-1.05183200	-0.43829900
C	0.94258800	-1.45688100	-0.51874500
C	0.12739000	-0.85996700	0.64336200
C	0.29620000	0.66596300	0.70142700
C	1.76627100	1.08437500	0.77103500
C	2.59312700	0.47224500	-0.37015600
H	0.50884100	-1.12235000	-1.46600000
H	0.84988800	-2.54751300	-0.49885300
H	2.87202500	-1.50822400	0.45311400
H	2.96567000	-1.45106400	-1.29944500
H	-0.29447200	1.11237200	1.50229000
H	1.82568800	2.17623200	0.75760900
H	2.15786900	0.75444800	1.74064500
H	2.27623000	0.91706400	-1.31904500

H	3.64738600	0.73229100	-0.23819000
H	0.56665600	-1.22687100	1.58380200
C	-1.32907500	-1.34612900	0.66816600
H	-1.85791800	-0.98549900	1.55284400
O	-2.09103000	-0.98168900	-0.48633900
O	-3.04060500	0.04276300	-0.11371100
H	-2.62758100	0.84238000	-0.48511700
O	-0.25315300	1.24274700	-0.54438900
O	-1.17385600	2.15895900	-0.31982700
H	-1.33045400	-2.44214600	0.68972300

TS57

C	2.03124400	-1.52721200	-0.37783800
C	0.50785900	-1.64725500	-0.50840700
C	-0.21586500	-0.92845500	0.64321100
C	0.23285100	0.53443700	0.75139900
C	1.75707900	0.71760100	0.73451700
C	2.48074600	-0.04669100	-0.35118500
H	0.17498700	-1.22722100	-1.46162600
H	0.21745900	-2.70288100	-0.50905500
H	2.36347800	-2.02945500	0.53907400
H	2.52628100	-2.03343100	-1.21184600
H	-0.20013800	1.01991800	1.63282100
H	1.53118600	1.95645700	0.27458600
H	2.24135300	0.75289500	1.71005000
H	2.24113500	0.39347500	-1.32467600
H	3.56222600	0.02700900	-0.21510200
H	0.11837700	-1.38681000	1.58675300
C	-1.74203700	-1.13459100	0.62645500
H	-2.21022300	-0.70947400	1.51898300
O	-2.40851900	-0.63017300	-0.52396100
O	-2.84145200	0.72607400	-0.24981600
H	-2.06684500	1.23162900	-0.55506500
O	-0.16081500	1.28461800	-0.42183900
O	0.55257100	2.50850700	-0.26713700
H	-1.94482400	-2.21132200	0.60822000

P57

C	2.42078400	1.03589300	0.35021100
C	0.94240700	1.39730200	0.53151900
C	0.08210900	0.88440200	-0.64208300
C	0.28132600	-0.62112900	-0.93984300
C	1.69505600	-1.06926300	-0.82189700
C	2.61609700	-0.48159600	0.19216100
H	0.57915800	0.98658200	1.47648200
H	0.83191600	2.48527500	0.59605900

H	2.81011200	1.54415100	-0.54037300
H	3.00384400	1.40036700	1.20093200
H	-0.10521800	-0.84075800	-1.94255900
H	1.95112000	-2.01209200	-1.29337700
H	2.42071100	-0.96512000	1.16459100
H	3.65655800	-0.71296800	-0.05804300
H	0.45749900	1.39087300	-1.54384900
C	-1.38640300	1.32055400	-0.53526600
H	-1.93491400	1.13626100	-1.46086200
O	-2.11366800	0.67920900	0.53011900
O	-3.03406100	-0.28607500	-0.03835100
H	-2.42272800	-1.03388200	-0.19688900
H	-1.42832900	2.39342300	-0.31905400
O	-0.60544800	-1.50827500	-0.16647700
O	-0.34201300	-1.37415100	1.25443700
H	-0.96646300	-0.65475400	1.47030300

R58

C	2.28433000	-1.38460300	-0.62618900
C	0.75156300	-1.35651000	-0.69813000
C	0.12746500	-0.67537600	0.53648100
C	0.74414300	0.71409200	0.79564600
C	2.27168800	0.70849600	0.79274000
C	2.86017500	0.02530300	-0.44893100
H	0.43365900	-0.81903100	-1.59514800
H	0.36222400	-2.37424900	-0.78843700
H	2.60358000	-2.01702300	0.21295000
H	2.68763500	-1.84482700	-1.53309300
H	0.34875800	1.14240200	1.71822400
H	2.63062200	1.73718000	0.88666600
H	2.59436400	0.17594500	1.69546400
H	2.63274400	0.62687600	-1.33549500
H	3.95026800	-0.01086800	-0.36430400
H	0.39638000	-1.26539700	1.42421800
C	-1.41518100	-0.61827700	0.49724800
H	-1.75670900	0.14222200	1.20455700
O	-1.78416500	-0.16457200	-0.81980400
O	-2.92600900	0.71234700	-0.70877900
H	-2.47300600	1.57366300	-0.70099500
O	0.32095500	1.65762200	-0.26129900
O	-0.69147400	2.40969200	0.11358800
C	-2.10695300	-1.94240900	0.80700400
H	-1.88104500	-2.27585200	1.82447000
H	-1.80006800	-2.72285300	0.10709800

H	-3.18724700	-1.81319800	0.71896700

TS58

C	1.61546200	-1.98853500	-0.48907400
C	0.16414600	-1.52873300	-0.68121600
C	-0.32184500	-0.68737200	0.51226800
C	0.62310200	0.49649600	0.76227600
C	2.10648500	0.10640100	0.81915200
C	2.57447700	-0.78860400	-0.30559100
H	0.07100200	-0.94016900	-1.59804600
H	-0.47791400	-2.40504700	-0.79897800
H	1.67965100	-2.64825300	0.38510600
H	1.94363200	-2.57640100	-1.35157600
H	0.33996100	1.04589800	1.66700700
H	2.37884700	1.37050900	0.46797700
H	2.50211500	-0.11107100	1.81097100
H	2.58220200	-0.22005200	-1.24130600
H	3.59513800	-1.13275700	-0.12255900
H	-0.22170100	-1.30287100	1.41911400
C	-1.81894200	-0.28586200	0.46203400
H	-2.04583600	0.32611800	1.34035000
O	-2.14682300	0.49892100	-0.69324900
O	-2.08205300	1.90513000	-0.35449100
H	-1.15200500	2.10122500	-0.56765800
O	0.61116200	1.41954200	-0.35237400
O	1.70796500	2.28123900	-0.05942000
C	-2.76100100	-1.48875500	0.42443500
H	-2.52424800	-2.19669100	1.22336600
H	-2.69413500	-2.01008100	-0.53230700
H	-3.79002300	-1.14922600	0.54911500

P58

C	-2.26927400	1.39554200	-0.46430200
C	-0.74964100	1.34514100	-0.66746600
C	-0.03277600	0.73641200	0.55606400
C	-0.61817800	-0.63124700	0.99162300
C	-2.10159200	-0.70277900	0.90796700
C	-2.85245400	0.00449100	-0.16742000
H	-0.52057300	0.76915100	-1.56695300
H	-0.37689900	2.35882900	-0.83704900
H	-2.49895500	2.06799800	0.37127600
H	-2.74959100	1.82028200	-1.35069900
H	-0.28610600	-0.84551600	2.01506000
H	-2.58669500	-1.49624600	1.46658100
H	-2.80142500	-0.60356100	-1.08666900
H	-3.91389400	0.07761100	0.09102800

H	-0.25048900	1.40064300	1.40505000
C	1.50783900	0.71555200	0.45065100
H	1.91475500	0.27501100	1.36371700
O	1.94006200	-0.15079500	-0.63602600
O	2.67017700	-1.27813800	-0.09525000
H	1.92340700	-1.84020600	0.19241600
C	2.14770500	2.08194900	0.22501800
H	1.82174400	2.79675500	0.98606700
H	1.89109100	2.48143400	-0.75758700
H	3.23360900	1.98783600	0.27672600
O	-0.00646700	-1.78844000	0.31776800
O	-0.28711800	-1.74571700	-1.10603100
H	0.47702000	-1.21566900	-1.40545700

R59

C	2.27339400	-1.73912000	-0.63406600
C	0.79252800	-1.36716800	-0.79100700
C	0.24527200	-0.63906500	0.45337100
C	1.12644500	0.56709000	0.83120400
C	2.61153500	0.22016000	0.92959900
C	3.13054800	-0.50812200	-0.31803900
H	0.66176100	-0.71971100	-1.66216400
H	0.20116300	-2.26729800	-0.97791700
H	2.38383400	-2.47651100	0.17218400
H	2.63166700	-2.22197900	-1.54806600
H	0.76434800	1.04128300	1.74474100
H	3.17846100	1.13600500	1.11862000
H	2.73699100	-0.41916200	1.81180200
H	3.11211400	0.17883900	-1.17079000
H	4.17555200	-0.79407300	-0.16665200
H	0.33132300	-1.32042600	1.31186200
C	-1.24928500	-0.26097100	0.34152500
H	-1.49018900	0.48388800	1.10531300
O	-1.45169500	0.37392400	-0.94031500
O	-2.21725500	1.58602900	-0.76830500
H	-1.49985100	2.24450800	-0.72293400
O	1.00062700	1.61311900	-0.20545700
O	0.24495800	2.61916500	0.18353400
C	-2.19991400	-1.45483900	0.48396700
H	-1.97701000	-1.96363100	1.42973100
H	-1.99126000	-2.17173800	-0.31654900
C	-3.67620100	-1.05381200	0.44104400
H	-3.90946000	-0.53870300	-0.49214900
H	-4.31950700	-1.93434100	0.51696600

H	-3.92895300	-0.37977000	1.26476000

TS59

C	-0.98908300	0.43636000	0.76959800
C	0.08281600	-0.61341200	0.44217800
C	-0.35864600	-1.49456600	-0.74016100
C	-1.72854300	-2.13525500	-0.48022100
C	-2.81776400	-1.06682300	-0.22658400
C	-2.40652500	-0.13942000	0.89427300
H	-0.39045200	-0.88780400	-1.64933000
H	0.11318900	-1.24936200	1.33978000
H	-0.72777600	0.99992400	1.67222700
H	-2.02709800	-2.75086900	-1.33410300
H	-3.77811500	-1.53724200	-0.00261000
H	-2.85469300	1.08622900	0.59050400
H	-2.71595300	-0.42457100	1.89953600
H	0.38092200	-2.28135700	-0.90795000
H	-1.66276000	-2.80614800	0.38525900
H	-2.94134100	-0.48898100	-1.14829500
O	-1.15515100	1.37507700	-0.31950700
O	-2.33323500	2.08510500	0.05413700
C	1.50968100	-0.02179600	0.31795500
H	1.71070300	0.59796000	1.19822700
C	2.60226500	-1.09196000	0.19684300
H	2.53511800	-1.55218100	-0.79357400
H	2.40222100	-1.88417200	0.92729200
C	4.00714300	-0.52126300	0.40236600
H	4.13126100	-0.12841000	1.41609700
H	4.76905200	-1.28905800	0.24540700
H	4.19212700	0.29332200	-0.29997200
O	1.65819400	0.82298500	-0.83283900
O	1.45069800	2.20460700	-0.45142200
H	0.49148100	2.28934000	-0.59893000

P59

C	-1.06586000	0.37685400	0.81421300
C	0.02931500	-0.63858200	0.40638300
C	-0.41690700	-1.43740600	-0.83350100
C	-1.75092000	-2.15726700	-0.58986700
C	-2.86277100	-1.18747400	-0.12973200
C	-2.38863700	-0.29411400	0.96887200
H	-0.49625700	-0.75922500	-1.68735800
H	0.06189200	-1.33353900	1.25723700
H	-0.77224500	0.91456500	1.71940900
H	-2.06974600	-2.67781300	-1.49803600
H	-3.75443400	-1.74118800	0.17655500

H	-3.02446300	-0.05099800	1.81119400
H	0.34729100	-2.17526000	-1.09068200
H	-1.61042300	-2.92374200	0.18159600
H	-3.16331400	-0.58118500	-1.00108600
C	1.45916400	-0.04973400	0.30720200
H	1.62654500	0.62741100	1.15186300
C	2.55024400	-1.13005100	0.31124400
H	2.50273000	-1.68475300	-0.63103600
H	2.33107500	-1.84665300	1.11062800
C	3.95397600	-0.54822900	0.49171000
H	4.05588200	-0.05388100	1.46265200
H	4.71367800	-1.33208900	0.43394100
H	4.16207900	0.18948700	-0.28497100
O	1.66593100	0.71275200	-0.89120000
O	1.47170100	2.11935000	-0.61789600
H	0.49826900	2.17160900	-0.65888800
O	-1.13203700	1.37135300	-0.26428200
O	-1.96361700	2.46830900	0.19125400
H	-2.79001600	2.27915500	-0.27516300

R60

C	1.18666700	-1.32685700	-0.66523800
C	1.74657400	-0.24452300	0.27610700
C	0.62480700	0.71280100	0.72203300
C	-0.55113100	-0.03784500	1.35741800
C	-1.07929400	-1.17593100	0.49042200
C	0.01381300	-2.09701000	-0.03889700
H	2.08927900	-0.73818400	1.19626100
H	0.85977900	-0.85638800	-1.59629500
H	1.98282900	-2.03050500	-0.92672500
H	-0.22292400	-0.46758300	2.30992100
H	-1.35999400	0.65585800	1.58543200
H	-1.85175000	-1.73670500	1.02334400
H	0.36824700	-2.69741400	0.80716900
H	-0.42083100	-2.79565000	-0.75949500
O	0.23250000	1.41255400	-0.46644900
O	-0.75760400	2.40664500	-0.12187700
H	-1.57117600	1.96813900	-0.42851800
O	-1.76412800	-0.64981400	-0.71900600
O	-2.69759100	0.23242300	-0.44273400
C	2.93809200	0.49884400	-0.33769600
H	3.33164500	1.24953200	0.35370800
H	3.74715600	-0.19867000	-0.57193800
H	2.64859300	1.01181600	-1.25628000

H	1.01656100	1.44440200	1.43905600

TS60

C	1.12066000	-1.26596200	0.38565900
C	-0.01468400	-2.13388600	-0.15140400
C	-1.20338200	-1.31736000	-0.67587400
C	-1.70142300	-0.28523100	0.35328400
C	-0.55665300	0.70427200	0.70182700
C	0.65042300	-0.05513700	1.20882400
H	-0.91993200	-0.79798400	-1.59412800
H	-0.34154800	-2.78451400	0.66814600
H	1.82743300	-1.85498700	0.98152900
H	-0.89372400	1.42334800	1.45646700
H	1.78784800	0.55727100	0.78834900
H	0.68461200	-0.17506800	2.29079800
O	1.82655200	-0.67416400	-0.72191700
O	2.58810900	0.36368500	-0.11353500
H	0.38602800	-2.79087300	-0.92975800
H	-2.02267100	-1.99689400	-0.93016100
H	-1.93514200	-0.81434800	1.28726000
C	-2.96374100	0.44928000	-0.11217900
H	-3.77741900	-0.26300800	-0.27563000
H	-2.78204500	0.98759400	-1.04324500
H	-3.29706500	1.17423500	0.63563900
O	-0.31218500	1.41300400	-0.51955800
O	0.62891700	2.48241200	-0.26792500
H	1.44528800	2.07987000	-0.61261500

P60

C	1.27619300	-0.44644500	0.80748800
C	0.68891300	-1.75436400	0.26192000
C	-0.60119400	-1.54472400	-0.54326000
C	-1.66062800	-0.74339400	0.23806400
C	-1.06562000	0.61374300	0.68316700
C	0.22263200	0.41723000	1.41481100
H	-0.37662800	-1.01481000	-1.47292700
H	0.48798400	-2.40622700	1.12058800
H	2.07907600	-0.65202200	1.52058200
H	-1.77984100	1.16773300	1.30228900
H	0.44466100	0.99558500	2.30219600
H	1.45247800	-2.26012300	-0.33618200
H	-1.01737100	-2.51790600	-0.82274300
H	-1.88460000	-1.28585400	1.16635100
C	-2.96666700	-0.57501600	-0.54688700
H	-3.39672100	-1.55187200	-0.78606600
H	-2.79803000	-0.03451800	-1.47971700

H	-3.70595100	-0.01636200	0.03428500
O	-0.87222800	1.34003400	-0.55461400
O	-0.12757800	2.55167300	-0.28499600
H	0.77606800	2.23338900	-0.45064900
O	1.85787800	0.37645600	-0.25045200
O	3.08321600	-0.26139000	-0.70794900
H	2.82679600	-0.51338400	-1.60575300

R61

C	1.18666700	-1.32685700	-0.66523800
C	1.74657400	-0.24452300	0.27610700
C	0.62480700	0.71280100	0.72203300
C	-0.55113100	-0.03784500	1.35741800
C	-1.07929400	-1.17593100	0.49042200
C	0.01381300	-2.09701000	-0.03889700
H	2.08927900	-0.73818400	1.19626100
H	0.85977900	-0.85638800	-1.59629500
H	1.98282900	-2.03050500	-0.92672500
H	-0.22292400	-0.46758300	2.30992100
H	-1.35999400	0.65585800	1.58543200
H	-1.85175000	-1.73670500	1.02334400
H	0.36824700	-2.69741400	0.80716900
H	-0.42083100	-2.79565000	-0.75949500
O	0.23250000	1.41255400	-0.46644900
O	-0.75760400	2.40664500	-0.12187700
H	-1.57117600	1.96813900	-0.42851800
O	-1.76412800	-0.64981400	-0.71900600
O	-2.69759100	0.23242300	-0.44273400
C	2.93809200	0.49884400	-0.33769600
H	3.33164500	1.24953200	0.35370800
H	3.74715600	-0.19867000	-0.57193800
H	2.64859300	1.01181600	-1.25628000
H	1.01656100	1.44440200	1.43905600

TS61

C	-1.33587800	-0.30732000	0.86268000
C	-0.25028200	0.56735500	1.47690400
C	1.02686000	0.64918000	0.63386700
C	1.56861500	-0.75307900	0.29509600
C	0.49627600	-1.54395900	-0.50653900
C	-0.82658200	-1.60859500	0.22098800
H	0.01747400	0.15061300	2.45330300
H	-2.11762700	-0.54446500	1.59243300
H	0.86241700	-2.55238200	-0.71470000
H	-1.85132600	-1.48669600	-0.65041100

H	-0.99555400	-2.50551500	0.81666700
H	1.73116500	-1.25909800	1.25560600
H	0.37079900	-1.03394200	-1.46552900
O	-1.94269800	0.41194900	-0.23321500
O	-2.65649200	-0.59392500	-0.93485200
H	-0.64588400	1.56823400	1.65984300
O	0.83154400	1.26572400	-0.64651900
O	0.07213400	2.48724200	-0.51095200
H	-0.80854000	2.15272300	-0.75346300
H	1.78035700	1.22031200	1.19254300
C	2.90285300	-0.70415200	-0.45744300
H	3.65701600	-0.16649900	0.12446500
H	3.27989700	-1.71349400	-0.64370100
H	2.79272900	-0.19429100	-1.41606000

P61

C	-1.04003900	-1.18558300	0.48343700
C	-0.48654400	-0.04756300	1.34798000
C	0.68495100	0.70254500	0.70536300
C	1.82087600	-0.25836400	0.31091900
C	1.29142600	-1.38318600	-0.61083200
C	0.04194500	-2.02534600	-0.10845200
H	-0.13892200	-0.48134200	2.29202700
H	-1.74422700	-1.79067800	1.06614600
H	2.07143900	-2.13660900	-0.76090900
H	-0.17989000	-3.05528000	-0.36330200
H	2.14669800	-0.72397400	1.24988700
H	1.09423000	-0.94407900	-1.60209100
H	-1.28614000	0.64917200	1.59871400
O	0.31680300	1.37246800	-0.50557000
O	-0.74557700	2.31333400	-0.22213900
H	-1.52929300	1.78019300	-0.44709800
H	1.06348500	1.45030200	1.41486100
C	3.02038900	0.46657600	-0.30772000
H	3.40853900	1.23000100	0.37255400
H	3.83061600	-0.23727600	-0.51944700
H	2.74026600	0.96151300	-1.23910100
O	-1.74961800	-0.69297600	-0.68798700
O	-2.81380200	0.22780700	-0.23332200
H	-3.53726400	-0.08149600	-0.79425500

R62

C	-1.70248000	-0.03179100	-0.41076000
C	-1.23004800	-1.27695900	0.36141200
C	0.27292300	-1.25803200	0.65349300

C	0.70395200	0.03079800	1.36137000
C	0.20139400	1.29303300	0.66952800
C	-1.28297900	1.25335600	0.32605400
H	-1.76117600	-1.34555900	1.31928500
H	-1.46952700	-2.18265100	-0.20266600
H	-1.19965400	-0.03683700	-1.38267500
H	0.30013600	0.02521900	2.37956500
H	1.78917600	0.06707700	1.45159800
H	0.45134400	2.17867700	1.25966400
H	-1.83918400	1.33781500	1.26836200
H	-1.53900500	2.13505600	-0.26948200
O	0.88642800	-1.40122800	-0.63599000
O	2.32115000	-1.45436800	-0.47204600
H	2.57038800	-0.55427200	-0.74656200
O	0.89552400	1.49813800	-0.62889300
O	2.20489000	1.48422200	-0.52304700
C	-3.21440800	-0.06171400	-0.64972500
H	-3.54196300	0.80904800	-1.22496800
H	-3.50655200	-0.95672100	-1.20583200
H	-3.76496400	-0.06283500	0.29763300
H	0.55691800	-2.12027100	1.26765800

TS62

C	0.26077200	1.07017800	0.96306300
C	0.97950100	-0.20295600	1.39108300
C	0.69282300	-1.40449800	0.48484400
C	-0.81230000	-1.61256400	0.29256500
C	-1.49050100	-0.36514100	-0.33577200
C	-1.18887800	0.86658800	0.49344200
H	0.64892500	-0.46001700	2.40278400
H	0.28272300	1.82600300	1.75574000
H	-0.97979700	-2.47388300	-0.36043800
H	-0.98765400	1.92824100	-0.32069300
H	-1.95064100	1.13261000	1.22813700
H	-1.26328700	-1.84770800	1.26363400
H	-1.01880200	-0.23618500	-1.31487400
O	0.92892900	1.61193300	-0.19716700
O	0.00209000	2.56456000	-0.69480300
H	2.05374900	-0.01839900	1.44723300
O	1.17988800	-1.24871800	-0.85720200
O	2.53470400	-0.74591900	-0.84978500
H	2.35529700	0.19552000	-1.01454100
H	1.15269700	-2.29718000	0.92733200
C	-2.99419000	-0.57245700	-0.53353800
H	-3.45270800	0.30064000	-1.00571400

H	-3.18828800	-1.43701100	-1.17369400
H	-3.50046100	-0.74237900	0.42263200

P62

C	-0.28818100	-1.24233800	0.66310600
C	-0.61824600	0.08490200	1.35503800
C	-0.07270200	1.31324900	0.62013800
C	1.42622400	1.18061700	0.34241100
C	1.78994200	-0.12365700	-0.40650900
C	1.13576600	-1.31367800	0.22082400
H	-0.17262700	0.06020600	2.35563100
H	-0.54628400	-2.08131900	1.31940100
H	1.76326900	2.04394400	-0.23784900
H	1.61056300	-2.28666400	0.15109900
H	1.95246700	1.20824900	1.30350500
H	1.36952000	-0.02343900	-1.42060500
H	-1.69532400	0.17554900	1.49351800
O	-0.66193100	1.50319600	-0.67265200
O	-2.09751500	1.61792000	-0.52790800
H	-2.37349200	0.69818400	-0.69165700
H	-0.26709100	2.20788700	1.22508200
C	3.30848300	-0.29357600	-0.53557600
H	3.55730400	-1.18880600	-1.11230600
H	3.75534100	0.56548300	-1.04302200
H	3.77809200	-0.38569100	0.44893400
O	-1.02520200	-1.41373800	-0.58019800
O	-2.46763000	-1.28301400	-0.28622500
H	-2.80122100	-2.02160400	-0.81245300

R63

C	-1.70248000	-0.03179100	-0.41076000
C	-1.23004800	-1.27695900	0.36141200
C	0.27292300	-1.25803200	0.65349300
C	0.70395200	0.03079800	1.36137000
C	0.20139400	1.29303300	0.66952800
C	-1.28297900	1.25335600	0.32605400
H	-1.76117600	-1.34555900	1.31928500
H	-1.46952700	-2.18265100	-0.20266600
H	-1.19965400	-0.03683700	-1.38267500
H	0.30013600	0.02521900	2.37956500
H	1.78917600	0.06707700	1.45159800
H	0.45134400	2.17867700	1.25966400
H	-1.83918400	1.33781500	1.26836200
H	-1.53900500	2.13505600	-0.26948200
O	0.88642800	-1.40122800	-0.63599000

O	2.32115000	-1.45436800	-0.47204600
H	2.57038800	-0.55427200	-0.74656200
O	0.89552400	1.49813800	-0.62889300
O	2.20489000	1.48422200	-0.52304700
C	-3.21440800	-0.06171400	-0.64972500
H	-3.54196300	0.80904800	-1.22496800
H	-3.50655200	-0.95672100	-1.20583200
H	-3.76496400	-0.06283500	0.29763300
H	0.55691800	-2.12027100	1.26765800

TS63

C	-0.13432800	1.44957100	0.48760600
C	1.34721100	1.28836200	0.15766000
C	1.71443100	-0.10715600	-0.37438600
C	1.18928500	-1.19949600	0.57451700
C	-0.34052400	-1.13458600	0.74920300
C	-0.75600600	0.24486600	1.21317600
H	1.21659700	-0.24183900	-1.33864700
H	1.91253800	1.49121400	1.07627800
H	-0.31634800	2.35721400	1.07453100
H	1.44093100	-2.19288100	0.19196900
H	-0.68188900	-1.89143000	1.46286900
H	-1.92455800	0.60279000	0.61594200
H	-0.86355500	0.34492400	2.29226000
H	1.65822300	-1.10143700	1.56125800
O	-0.88250500	1.53315400	-0.74171400
O	-2.22608700	1.31187500	-0.32800800
H	1.63175200	2.06447900	-0.56055300
C	3.22634900	-0.23506700	-0.58453500
H	3.59390600	0.52807600	-1.27631500
H	3.76921800	-0.11888500	0.36011300
H	3.48458400	-1.21284500	-1.00005900
O	-0.84126600	-1.48171200	-0.55013800
O	-2.28353700	-1.58076200	-0.49356000
H	-2.53273500	-0.72879100	-0.89173300

P63

C	0.41917100	-0.97815500	0.92323200
C	-1.06468500	-1.11337100	0.56152700
C	-1.58139100	0.01143400	-0.35184300
C	-1.25717000	1.39254100	0.24741100
C	0.24160100	1.55880000	0.53899900
C	0.76726200	0.41146900	1.33788200
H	-1.04906800	-0.05851500	-1.30566600
H	-1.63561600	-1.11469900	1.49923300
H	0.69409400	-1.70110500	1.69617400

H	-1.56995600	2.18707600	-0.43700900
H	0.44628400	2.50762200	1.04535000
H	1.51560900	0.57244800	2.10294400
H	-1.80857000	1.53645700	1.18436800
H	-1.22623900	-2.09337600	0.10174700
C	-3.08158100	-0.13924100	-0.62223200
H	-3.30685200	-1.10968100	-1.07434700
H	-3.65975700	-0.06274900	0.30511800
H	-3.44009300	0.63731700	-1.30377400
O	0.83301600	1.60994900	-0.78532100
O	2.27435700	1.53690800	-0.66982500
H	2.40298600	0.57815200	-0.76397300
O	1.28892800	-1.24019700	-0.22131500
O	1.25036900	-2.66649800	-0.51001400
H	0.80458200	-2.66110700	-1.36822000

R64

C	-1.17823600	-1.33558100	-0.65978600
C	-0.00260400	-2.08634900	-0.01653000
C	1.09824900	-1.13612300	0.46275600
C	0.54309700	-0.02584200	1.36218900
C	-0.65982500	0.69482000	0.75916800
C	-1.75997400	-0.25265800	0.26796800
H	-0.34991600	-2.66115700	0.84988500
H	0.42834100	-2.80045300	-0.72276000
H	-0.84619700	-0.87129100	-1.59228100
H	-1.96904600	-2.04506100	-0.92196900
H	0.22795100	-0.46721700	2.31375400
H	1.32510600	0.69734500	1.59205600
H	-1.05903000	1.42997500	1.46384400
H	-2.12553400	-0.74982000	1.17745900
O	1.67258700	-0.62193100	-0.74759000
O	2.76355000	0.26496100	-0.41376400
H	2.34894100	1.13211400	-0.56820600
O	-0.24171300	1.48894500	-0.42224300
O	0.71205700	2.35097700	-0.14707900
C	-2.93805900	0.49413800	-0.36898000
H	-3.73672400	-0.20508600	-0.62983600
H	-3.35518400	1.23773200	0.31663900
H	-2.63108200	1.01183200	-1.28001200
H	1.87464200	-1.68696300	1.00612000

TS64

C	-0.76003500	-0.78981200	0.68518100
C	-1.81101600	0.23676800	0.24284700

C	-1.16903500	1.39975500	-0.53262700
C	-0.01274300	2.04677800	0.24229200
C	1.10472600	1.03043600	0.54949000
C	0.52691800	-0.17541300	1.26051000
H	-0.80134100	1.03863200	-1.49614200
H	-2.21955100	0.64439400	1.17846400
H	-1.18282300	-1.49066200	1.41565500
H	0.42871800	2.86789700	-0.32880700
H	1.88989600	1.48779000	1.16024300
H	1.14994800	-1.30307500	0.82701800
H	0.58301000	-0.12674300	2.34708200
H	-1.93438600	2.15357300	-0.74232700
H	-0.37121300	2.46338100	1.19023100
O	-0.29673400	-1.53705700	-0.45677900
O	0.87533800	-2.18310200	0.02672900
C	-2.95923200	-0.42815200	-0.52796800
H	-3.42182200	-1.22645400	0.05915100
H	-2.59895500	-0.86423600	-1.46198900
O	1.65208200	0.73504800	-0.74294100
O	2.80446900	-0.12575100	-0.58684100
H	2.41305300	-0.98536500	-0.82041500
H	-3.73327600	0.30560400	-0.76785900

P64

C	0.65302700	0.69682600	0.76180900
C	1.76533200	-0.27303600	0.29177600
C	1.18398000	-1.35588500	-0.63664900
C	0.02018300	-2.12407100	0.00678700
C	-1.09306700	-1.18709400	0.50150300
C	-0.52349600	-0.05387200	1.29310800
H	0.84156300	-0.88901400	-1.56338400
H	2.10886100	-0.76417500	1.21197000
H	1.04406300	1.39210700	1.51321900
H	-0.41292700	-2.83416900	-0.70343800
H	-1.83190100	-1.73627700	1.09441600
H	-1.00523300	0.27504800	2.20462300
H	1.97975000	-2.05859300	-0.90457400
H	0.37836100	-2.70264400	0.86561300
C	2.96027100	0.45758300	-0.33214200
H	3.35424400	1.22267900	0.34344600
H	2.68129800	0.94656600	-1.26737000
O	-1.73666800	-0.73544400	-0.71343300
O	-2.77315300	0.21299200	-0.36264800
H	-2.27092600	1.04746900	-0.39525100
H	3.76757100	-0.24870300	-0.54492800

O	0.32446200	1.46654400	-0.42226200
O	-0.79469700	2.34284900	-0.06753700
H	-0.59165500	3.09145800	-0.64445700

R65

C	0.52174000	1.88594500	-0.90543000
C	-0.90467600	1.65797300	-0.38764500
C	-1.12111300	0.24393800	0.18042600
C	-0.04092200	-0.10271200	1.22234900
C	1.38741900	0.18944800	0.77060000
C	1.57145300	1.57780500	0.17094800
H	-1.13559700	2.37433200	0.40950800
H	-1.63110800	1.82418300	-1.18763500
H	0.69569800	1.24889100	-1.77521300
H	0.63275900	2.92148000	-1.23962700
H	-0.22210900	0.48531500	2.12807100
H	-0.12374000	-1.15223800	1.50610100
H	2.08652700	0.00991900	1.59159500
H	1.49408100	2.29994900	0.99226300
H	2.58381100	1.66590200	-0.23315200
C	-2.52387900	0.07566900	0.76323500
H	-3.27158800	0.32472300	0.00697200
H	-2.68162200	-0.95770300	1.07424200
H	-2.66946100	0.72906600	1.62777400
O	-0.97772900	-0.58836000	-0.99747700
O	-1.13076500	-1.98131300	-0.64244300
H	-0.20300100	-2.27689200	-0.64656600
O	1.81432600	-0.75070100	-0.29752500
O	1.68232100	-2.01279200	0.04479000

TS65

C	1.52121100	0.22372000	0.66974000
C	2.00706700	-1.09453500	0.07464900
C	0.98020800	-1.72458900	-0.87309400
C	-0.39327600	-1.85927800	-0.20422900
C	-0.97523100	-0.49546600	0.24621700
C	0.04661900	0.22266200	1.11399300
H	0.88376100	-1.11286300	-1.77175200
H	2.21439100	-1.77432300	0.90949400
H	2.14481000	0.53663300	1.51519700
H	-1.11556100	-2.31104800	-0.89014000
H	0.05940600	1.55320500	0.82806300
H	-0.11459000	0.10879200	2.18501400
H	-0.32904200	-2.50870000	0.67624900
O	1.57923000	1.24142500	-0.34854400

O	0.79061900	2.29531000	0.19213600
H	2.96136300	-0.91938100	-0.43156600
C	-2.31884800	-0.65611400	0.95522200
H	-2.74277800	0.32163600	1.18626400
H	1.33162400	-2.71125600	-1.18912200
H	-2.20364500	-1.22125500	1.88405900
H	-3.01697400	-1.18703600	0.30495200
O	-1.16660100	0.16821700	-1.02887300
O	-1.74448100	1.48124000	-0.83828600
H	-0.94939900	2.03766800	-0.91316400

P65

C	-1.15038500	0.16758800	0.90189700
C	-1.30616900	1.54390400	0.24601600
C	-0.30313600	1.76907300	-0.89227100
C	1.14280400	1.52491500	-0.43730000
C	1.33599800	0.11703600	0.16495700
C	0.27659800	-0.15840900	1.19054400
H	-0.52942200	1.09381700	-1.72081300
H	-1.15996300	2.29943400	1.02750500
H	-1.75799200	0.09996800	1.80850000
H	1.83383900	1.63437500	-1.27844400
H	0.51895200	-0.74337800	2.06947300
H	1.43511700	2.26026800	0.32060200
H	-2.33717900	1.65453900	-0.10190000
C	2.74073000	-0.10709800	0.71562600
H	2.86103600	-1.15427200	0.99923400
H	-0.40186100	2.78983000	-1.27381300
H	2.92181400	0.51838400	1.59447800
H	3.48525900	0.13211200	-0.04658200
O	1.13630100	-0.73431600	-1.00874700
O	0.99258800	-2.11555600	-0.59652700
H	0.02487300	-2.16757100	-0.51814100
O	-1.59829000	-0.91006800	0.02105400
O	-3.04959700	-0.86020300	-0.06875700
H	-3.16113000	-0.59841800	-0.99310400

R66

C	0.52174000	1.88594500	-0.90543000
C	-0.90467600	1.65797300	-0.38764500
C	-1.12111300	0.24393800	0.18042600
C	-0.04092200	-0.10271200	1.22234900
C	1.38741900	0.18944800	0.77060000
C	1.57145300	1.57780500	0.17094800
H	-1.13559700	2.37433200	0.40950800

H	-1.63110800	1.82418300	-1.18763500
H	0.69569800	1.24889100	-1.77521300
H	0.63275900	2.92148000	-1.23962700
H	-0.22210900	0.48531500	2.12807100
H	-0.12374000	-1.15223800	1.50610100
H	2.08652700	0.00991900	1.59159500
H	1.49408100	2.29994900	0.99226300
H	2.58381100	1.66590200	-0.23315200
C	-2.52387900	0.07566900	0.76323500
H	-3.27158800	0.32472300	0.00697200
H	-2.68162200	-0.95770300	1.07424200
H	-2.66946100	0.72906600	1.62777400
O	-0.97772900	-0.58836000	-0.99747700
O	-1.13076500	-1.98131300	-0.64244300
H	-0.20300100	-2.27689200	-0.64656600
O	1.81432600	-0.75070100	-0.29752500
O	1.68232100	-2.01279200	0.04479000

TS66

C	-1.13226500	0.02043800	1.00214100
C	0.35518300	0.17804900	1.29497300
C	1.28408000	-0.20415200	0.12730300
C	0.91987700	-1.60171900	-0.40347300
C	-0.55224600	-1.67305800	-0.87728600
C	-1.50809200	-1.24313700	0.21105100
H	0.59944600	-0.46693900	2.14484400
H	-1.72038000	0.05791400	1.92565600
H	1.57855500	-1.85018200	-1.24022100
H	-0.78907400	-2.68522700	-1.21330700
H	-2.52272700	-0.51913600	-0.31081400
H	-1.94027900	-2.04467300	0.80996700
H	1.10940800	-2.33384600	0.38874600
H	-0.64814900	-1.00698500	-1.73793600
O	-1.56102700	1.10752800	0.15250500
O	-2.82592000	0.67670600	-0.32458800
H	0.55600800	1.20395700	1.61077900
C	2.75046600	-0.10253600	0.54665400
H	2.98486800	0.92700000	0.82445000
H	2.96959700	-0.75269300	1.39853200
H	3.39607700	-0.38726700	-0.28672100
O	1.06237200	0.62471500	-1.03859400
O	1.03163700	2.02357800	-0.67434200
H	0.06814000	2.15455400	-0.64201200

P66

C	1.15540000	0.26692900	0.88369600

C	-0.26535900	-0.16115000	1.26734900
C	-1.31257800	0.03947800	0.15600800
C	-1.28987700	1.49818400	-0.32806300
C	0.10762300	1.93620500	-0.80826000
C	1.19713100	1.55659600	0.13907500
H	-0.57153400	0.43539700	2.13179900
H	1.78937500	0.30987600	1.77369700
H	-2.01648700	1.61774800	-1.13616300
H	0.12127700	3.01592000	-0.98844300
H	2.11369200	2.13554300	0.16093700
H	-1.61853200	2.13274600	0.50146400
H	0.29184700	1.46449600	-1.78610500
H	-0.25269700	-1.20472500	1.58709900
C	-2.70039400	-0.39385000	0.62599100
H	-2.69740100	-1.46105300	0.85411800
H	-3.00592000	0.15488900	1.52132900
H	-3.43445900	-0.21350200	-0.16247400
O	-0.95992200	-0.68406500	-1.04681000
O	-0.73143000	-2.08145300	-0.74472600
H	0.23625700	-2.06902100	-0.64235100
O	1.67129900	-0.85759200	0.08717500
O	3.09162700	-0.68337400	-0.09977700
H	3.12032700	-0.32078400	-0.99658300

R67

C	-0.95425900	-0.31378700	0.06000300
C	-0.15355000	-1.53541800	0.54969600
C	1.36023800	-1.31185800	0.51490500
C	1.77005600	-0.04729100	1.27592700
C	0.96034500	1.17982800	0.87495900
C	-0.54595400	0.94522100	0.84903400
H	-0.43626600	-1.77799300	1.58188400
H	-0.39476700	-2.40950100	-0.06146400
H	-0.69267500	-0.14915200	-0.99068500
H	1.61382800	-0.21470500	2.34708000
H	2.83269700	0.15011600	1.13805900
H	1.21819600	2.03234300	1.50898000
H	-0.88035500	0.85194700	1.89036300
H	-1.03171400	1.83520600	0.44131800
O	1.67508300	-1.23122600	-0.88306500
O	3.10350000	-1.07561900	-1.03636000
H	3.16374600	-0.12484200	-1.23653700
O	1.30784200	1.61814100	-0.50254300
O	2.59707200	1.79905500	-0.67843900

C	-2.46543200	-0.58766600	0.13886900
H	-2.66282400	-1.55510700	-0.33934900
H	-2.75727400	-0.70652000	1.19189800
H	1.88676100	-2.17556500	0.93699800
C	-3.35917900	0.47069200	-0.52085500
H	-3.23218500	1.43695400	-0.02107700
H	-3.03378700	0.61758200	-1.55756000
C	-4.84263100	0.08979400	-0.50004600
H	-5.20478400	-0.03698800	0.52506100
H	-5.45775300	0.85784200	-0.97629200
H	-5.01661800	-0.85151700	-1.03037100

TS67

C	1.13521600	1.05078300	0.99799400
C	2.01483700	-0.17204200	1.22323600
C	1.62293300	-1.37603700	0.36136400
C	0.12934700	-1.68982900	0.48650900
C	-0.75284200	-0.48647600	0.05391500
C	-0.36444600	0.74963500	0.84060600
H	1.92654900	-0.46923000	2.27337700
H	1.27410100	1.79284200	1.79190300
H	-0.11392500	-2.55040700	-0.14304500
H	-0.41499500	1.83783600	0.04328500
H	-0.96386700	0.94146300	1.73235000
H	-0.08602400	-1.97352200	1.52313700
H	-0.51016600	-0.30801300	-0.99918600
O	1.50000200	1.66049500	-0.25911900
O	0.42649300	2.55335400	-0.51359600
H	3.06100700	0.09023500	1.05602300
O	1.80041300	-1.16396300	-1.04809700
O	3.08685000	-0.56100800	-1.31255700
H	2.81094700	0.36689700	-1.40493800
H	2.22574200	-2.24026700	0.66792600
C	-2.24907100	-0.81309400	0.16334900
H	-2.43520600	-1.76489400	-0.34866400
H	-2.50908300	-0.97823400	1.21804300
C	-3.17350600	0.25708800	-0.43084400
H	-3.01843500	1.21039700	0.08717800
H	-2.89191900	0.43135700	-1.47590800
C	-4.65444000	-0.12416400	-0.35462800
H	-4.97274400	-0.27416600	0.68182000
H	-5.28819800	0.65452900	-0.78680200
H	-4.85202400	-1.05302400	-0.89855900

P67

C	-1.30289600	-1.27936100	0.60828400

C	-1.64942500	0.03320800	1.31804200
C	-0.88388800	1.24200800	0.77380000
C	0.62696000	0.99574200	0.75327300
C	1.02333300	-0.29913300	0.00233000
C	0.16743100	-1.45250700	0.42307200
H	-1.39634100	-0.08238500	2.37775800
H	-1.74047000	-2.12615500	1.14933200
H	1.11482200	1.85909400	0.29615100
H	0.56845600	-2.45954700	0.37778900
H	0.97492500	0.93520500	1.79118000
H	0.81218500	-0.11523200	-1.06494800
H	-2.72315200	0.21291900	1.26994000
O	-1.21306000	1.55191400	-0.58680600
O	-2.63755700	1.78666200	-0.68963700
H	-2.95071400	0.90538000	-0.96185800
H	-1.11209200	2.11849000	1.39351100
C	2.52248700	-0.62934500	0.13423000
H	2.70168000	-1.60391700	-0.33698300
H	2.77037900	-0.75478400	1.19653300
O	-1.81153500	-1.31873800	-0.75511900
O	-3.26931700	-1.08001300	-0.71302100
H	-3.55693800	-1.76171900	-1.33449000
C	3.47098700	0.39759100	-0.49611800
H	3.35638100	1.36813500	-0.00250800
H	3.18594100	0.55223100	-1.54372300
C	4.93960200	-0.03099200	-0.42351000
H	5.59517300	0.71553900	-0.87968800
H	5.26197800	-0.16593500	0.61376100
H	5.10198600	-0.97918400	-0.94552100

R68

C	-0.95425900	-0.31378700	0.06000300
C	-0.15355000	-1.53541800	0.54969600
C	1.36023800	-1.31185800	0.51490500
C	1.77005600	-0.04729100	1.27592700
C	0.96034500	1.17982800	0.87495900
C	-0.54595400	0.94522100	0.84903400
H	-0.43626600	-1.77799300	1.58188400
H	-0.39476700	-2.40950100	-0.06146400
H	-0.69267500	-0.14915200	-0.99068500
H	1.61382800	-0.21470500	2.34708000
H	2.83269700	0.15011600	1.13805900
H	1.21819600	2.03234300	1.50898000
H	-0.88035500	0.85194700	1.89036300

H	-1.03171400	1.83520600	0.44131800
O	1.67508300	-1.23122600	-0.88306500
O	3.10350000	-1.07561900	-1.03636000
H	3.16374600	-0.12484200	-1.23653700
O	1.30784200	1.61814100	-0.50254300
O	2.59707200	1.79905500	-0.67843900
C	-2.46543200	-0.58766600	0.13886900
H	-2.66282400	-1.55510700	-0.33934900
H	-2.75727400	-0.70652000	1.19189800
H	1.88676100	-2.17556500	0.93699800
C	-3.35917900	0.47069200	-0.52085500
H	-3.23218500	1.43695400	-0.02107700
H	-3.03378700	0.61758200	-1.55756000
C	-4.84263100	0.08979400	-0.50004600
H	-5.20478400	-0.03698800	0.52506100
H	-5.45775300	0.85784200	-0.97629200
H	-5.01661800	-0.85151700	-1.03037100

TS68

C	-0.92724500	1.38983400	0.66228200
C	0.57670300	1.12609100	0.66005600
C	0.96284500	-0.26492600	0.12598500
C	0.15334600	-1.35625500	0.85153600
C	-1.36677100	-1.17492700	0.67798400
C	-1.78256400	0.20502500	1.13787700
H	0.69439900	-0.30728600	-0.93417700
H	0.93049800	1.23185000	1.69383400
H	-1.17394800	2.27516300	1.25970900
H	0.41729400	-2.34559600	0.46687400
H	-1.91461800	-1.94082800	1.23657600
H	-2.75460200	0.69428800	0.32233200
H	-2.12746600	0.25243300	2.16977400
H	0.38485000	-1.35107100	1.92363800
O	-1.36593500	1.60790100	-0.69362300
O	-2.78092100	1.47926200	-0.60980800
H	1.05873100	1.92009600	0.08284500
C	2.47149700	-0.53906100	0.25256200
H	2.76766200	-0.45847000	1.30799900
H	2.65593500	-1.58179100	-0.03394300
O	-1.57465400	-1.40394000	-0.72335200
O	-2.99400800	-1.38458300	-1.00328400
H	-3.08542700	-0.49259100	-1.38079400
C	3.37218600	0.36441200	-0.59981700
H	3.25801700	1.40917500	-0.29177700
H	3.04139200	0.31554600	-1.64396700

C	4.85147900	-0.02299600	-0.51324900
H	5.01222100	-1.04967700	-0.85631500
H	5.47154900	0.63371900	-1.12910000
H	5.21879800	0.04273800	0.51574400

P68

C	-1.51246500	0.96435600	0.83630600
C	-0.00833500	1.25826100	0.86130300
C	0.84960400	0.18466400	0.16727000
C	0.52203200	-1.21343500	0.72739000
C	-0.97659700	-1.53997300	0.63933600
C	-1.80847800	-0.44524900	1.22100400
H	0.58551500	0.17863400	-0.89590800
H	0.29357700	1.34435700	1.91327000
H	-2.05256900	1.67033900	1.47319400
H	1.06935500	-1.98822400	0.18483300
H	-1.20236100	-2.49508900	1.12466900
H	-2.70594000	-0.67437300	1.78073700
H	0.82617700	-1.27701500	1.77937600
H	0.16410900	2.24096400	0.41129000
C	2.34405200	0.53093900	0.28794500
H	2.48017500	1.57951900	-0.00691000
H	2.64285100	0.47491900	1.34388800
O	-1.20020600	-1.68835100	-0.78717500
O	-2.62246600	-1.77721700	-1.04222200
H	-2.82180200	-0.84127800	-1.21245400
O	-2.07646600	1.09681700	-0.50501000
O	-2.10819700	2.51014800	-0.85255800
H	-1.45457800	2.53147900	-1.56499900
C	3.28790000	-0.33649100	-0.55479600
H	3.21792100	-1.38288200	-0.24024200
H	2.96028800	-0.31060500	-1.60094700
C	4.74840800	0.11606100	-0.46471300
H	5.11352100	0.07217400	0.56618700
H	5.39972300	-0.51613900	-1.07407300
H	4.86599200	1.14723700	-0.81246400

R69

C	-0.05906900	-1.31427000	-0.43350300
C	1.19135100	-2.08106800	0.02439800
C	2.36550900	-1.15069500	0.33594900
C	1.96489100	-0.03932900	1.31229700
C	0.69499900	0.69804800	0.89688400
C	-0.48353500	-0.22825500	0.57511300
H	0.96983300	-2.65531500	0.93148400

H	1.50007000	-2.79776500	-0.74084800
H	0.13556100	-0.84792200	-1.40334000
H	-0.87870300	-2.02193700	-0.57898800
H	1.78860300	-0.48285200	2.29814900
H	2.78107300	0.67348900	1.42656200
H	-0.71561300	-0.72513900	1.52886300
O	2.76042200	-0.63678700	-0.94432000
O	3.90047300	0.23464800	-0.77266400
H	3.47859800	1.10798700	-0.85724800
O	0.94423200	1.49121200	-0.33257900
O	1.93772100	2.34169400	-0.19900900
H	3.20631800	-1.71526600	0.75519900
H	0.41702700	1.43644300	1.65429600
C	-1.73428600	0.56200400	0.14559200
H	-1.88319100	1.38744800	0.85360000
H	-1.55053300	1.02726500	-0.82845700
C	-3.02256600	-0.26805000	0.08544900
H	-2.92426900	-1.06184700	-0.66350300
H	-3.17620000	-0.77278300	1.04875300
C	-4.26233100	0.57141400	-0.24569100
H	-4.37617400	1.36002300	0.50789400
H	-4.10356000	1.08551900	-1.20106300
C	-5.54923500	-0.25509700	-0.31841500
H	-5.75167500	-0.75773900	0.63261700
H	-6.41336500	0.37250600	-0.55206900
H	-5.48095000	-1.02647600	-1.09176900

TS69

C	0.63951200	-0.82224300	0.86723700
C	-0.51843800	0.15808100	0.63418900
C	-0.07320900	1.34354400	-0.24091100
C	1.18088900	2.03537600	0.31293200
C	2.37621700	1.06737500	0.40480900
C	1.98486400	-0.15859100	1.20245500
H	0.12585000	0.99307300	-1.25677200
H	-0.76234800	0.55099400	1.63261600
H	0.38593400	-1.54075100	1.65658100
H	1.47620600	2.87297600	-0.32477900
H	3.24045400	1.55732400	0.86505200
H	2.56060600	-1.26181200	0.65632900
H	2.23428600	-0.10459000	2.26127600
H	-0.88234200	2.07543000	-0.30459500
H	0.98570700	2.43908800	1.31273600
O	0.91543900	-1.54915800	-0.34647000
O	2.17886400	-2.15143200	-0.08853100

C	-1.76301600	-0.58245300	0.10575200
H	-1.93010400	-1.46787600	0.73170800
H	-1.55123800	-0.95930900	-0.90042800
C	-3.04615700	0.25696300	0.08865400
H	-2.92806900	1.11256700	-0.58576400
H	-3.22137300	0.67612900	1.08863700
O	2.69030500	0.79284000	-0.96742500
O	3.88358900	-0.02301000	-1.02938600
H	3.48859900	-0.89764700	-1.19008500
C	-4.27960400	-0.54539200	-0.34359200
H	-4.41198700	-1.39668300	0.33488700
H	-4.09936400	-0.97402200	-1.33652600
C	-5.56300300	0.28918600	-0.37267000
H	-5.47491500	1.12672900	-1.07170300
H	-6.42220000	-0.31172700	-0.68238600
H	-5.78761200	0.70510900	0.61458700

P69

C	-0.69548700	0.70843800	0.88211300
C	0.49408600	-0.23045200	0.55920100
C	0.05859000	-1.32011700	-0.44079100
C	-1.16106300	-2.11646200	0.04747000
C	-2.35131700	-1.21010900	0.39609400
C	-1.91381400	-0.06886700	1.25630700
H	-0.17508700	-0.85314200	-1.40086400
H	0.72641500	-0.71963900	1.51580700
H	-0.42103300	1.40946900	1.67834800
H	-1.48133300	-2.83326100	-0.71413900
H	-3.14660400	-1.77967200	0.88821700
H	-2.51261600	0.24266400	2.10222000
H	0.88766500	-2.01210400	-0.61111400
H	-0.90214200	-2.69068300	0.94405500
C	1.74376500	0.55488500	0.11847400
H	1.87904000	1.40313600	0.80152300
H	1.56918300	0.98781500	-0.87178500
C	3.03664300	-0.27047400	0.10114100
H	2.94856300	-1.09267600	-0.61796300
H	3.18380600	-0.73775100	1.08403200
O	-2.84471300	-0.76874500	-0.89080900
O	-3.93837300	0.15538700	-0.67479500
H	-3.45450500	1.00056100	-0.64135500
O	-0.88573900	1.47549600	-0.33380700
O	-2.06018400	2.32652200	-0.12456500
H	-1.80108900	3.08125000	-0.67022600
C	4.27573600	0.56189000	-0.25004300

H	4.37971500	1.37773500	0.47553200
H	4.12288200	1.04045000	-1.22482200
C	5.56721400	-0.26025100	-0.28236200
H	6.43075900	0.36252900	-0.53114200
H	5.76353900	-0.72754100	0.68776400
H	5.50878300	-1.05931400	-1.02790500

R70

C	-0.91826500	-1.25626900	-0.54744800
C	0.24978100	-2.06634500	0.03612900
C	1.42998500	-1.17912200	0.43910500
C	0.99140100	-0.03237800	1.35636100
C	-0.20278500	0.74864000	0.81546700
C	-1.38535500	-0.13496300	0.40129300
H	-0.07861400	-2.61298300	0.92777700
H	0.59584500	-2.81033900	-0.68588100
H	-0.61391300	-0.81661600	-1.50131800
H	-1.75173900	-1.93066300	-0.75852800
H	0.70816500	-0.44781000	2.32939600
H	1.82413100	0.64805300	1.53241900
H	-0.51690400	1.51196500	1.53315500
H	-1.72433200	-0.60363000	1.33728900
O	1.96050300	-0.70865600	-0.80844000
O	3.11764400	0.11718100	-0.54926100
H	2.74397900	1.00538600	-0.68782000
O	0.18947200	1.50783200	-0.39806800
O	1.20436000	2.31692800	-0.18891900
C	-2.55782900	0.69721300	-0.15229600
H	-2.73212700	1.54219700	0.52464200
H	-2.26801300	1.13306900	-1.11309000
H	2.20421800	-1.76973700	0.94234000
C	-3.86443200	-0.08687200	-0.31043900
H	-4.67184700	0.57214700	-0.63945200
H	-3.77672200	-0.88665000	-1.04974400
H	-4.17297200	-0.54009600	0.63728600

TS70

C	-0.27846900	-0.86692700	0.75916600
C	-1.43142100	0.08658800	0.41650500
C	-0.92776200	1.30121100	-0.38325500
C	0.23660200	2.01543400	0.31816300
C	1.44245400	1.07847800	0.52359100
C	1.00249800	-0.17388700	1.25176600
H	-0.60727500	0.97702000	-1.37652200
H	-1.79571800	0.45231600	1.38839100

H	-0.59597900	-1.60784300	1.50338100
H	0.57536900	2.87415000	-0.26774700
H	2.23659600	1.58209600	1.08439500
H	1.66623100	-1.24964000	0.75298500
H	1.13341600	-0.13542900	2.33232100
H	-1.74619700	2.01183800	-0.52382400
H	-0.07896500	2.39228400	1.29748100
O	0.14969200	-1.56121000	-0.42918500
O	1.39435800	-2.13396500	-0.04444000
C	-2.58939000	-0.67437300	-0.25967600
H	-2.80129200	-1.57408400	0.32938500
H	-2.25604000	-1.02637500	-1.24070700
C	-3.87771100	0.14143900	-0.40427700
H	-3.74828100	1.00053800	-1.06700700
H	-4.67746500	-0.47444400	-0.82344300
H	-4.22243400	0.51562600	0.56528100
O	1.91223200	0.84075400	-0.81043500
O	3.12847000	0.05899700	-0.75497800
H	2.77921200	-0.82243000	-0.97396600

P70

C	0.19638800	0.75585300	0.80780400
C	1.39457600	-0.13823100	0.40056200
C	0.92665700	-1.25825100	-0.54895400
C	-0.21280900	-2.09901700	0.04730300
C	-1.40857900	-1.23897400	0.48384700
C	-0.95217100	-0.06674200	1.29091400
H	0.59422000	-0.81423700	-1.49055100
H	1.72379200	-0.60398900	1.34036000
H	0.50378800	1.48101900	1.56970100
H	-0.56363100	-2.83999000	-0.67678600
H	-2.13426000	-1.83444800	1.04768600
H	-1.49220500	0.23254800	2.17972500
H	1.76815300	-1.91665200	-0.78084600
H	0.14419900	-2.64836800	0.92560300
C	2.57250500	0.68895500	-0.14876000
H	2.72977600	1.54881100	0.51304200
H	2.30019100	1.10234700	-1.12423100
C	3.88404700	-0.09546900	-0.26323700
H	3.81027200	-0.91550400	-0.98174200
H	4.69646200	0.55582500	-0.59620700
H	4.17676900	-0.52254100	0.70150400
O	-2.02647800	-0.83849900	-0.76188900
O	-3.13759800	0.04137900	-0.46494000
H	-2.68951700	0.90664100	-0.48060100

O	-0.12420100	1.49323200	-0.39890100
O	-1.31564600	2.29564400	-0.10818400
H	-1.14030700	3.04574500	-0.69221300

R71

C	-0.42916700	-1.35505100	-0.50081200
C	0.83614600	-2.07791200	-0.01322600
C	1.95897100	-1.10456100	0.35318800
C	1.47986100	-0.02826700	1.33324800
C	0.19407300	0.66256100	0.88808300
C	-0.93268600	-0.30666200	0.51106200
H	0.60893400	-2.67762300	0.87584600
H	1.20030500	-2.76707200	-0.77941600
H	-0.22093800	-0.86270500	-1.45476200
H	-1.21340000	-2.09269900	-0.68734100
H	1.29055100	-0.49595100	2.30537300
H	2.26096100	0.71599700	1.48623100
H	-0.14018700	1.37439300	1.64828100
H	-1.17432200	-0.83011600	1.44809500
O	2.37316400	-0.55285100	-0.90505600
O	3.46908000	0.36231300	-0.68257900
H	3.01348900	1.21843100	-0.76803500
O	0.44844400	1.48826400	-0.31870100
O	1.40179700	2.37550000	-0.13901800
C	-2.20073100	0.43883000	0.05396900
H	-2.39923800	1.25408800	0.76184700
H	-2.01124200	0.91719400	-0.91272700
H	2.80881700	-1.64086500	0.79101400
C	-3.45635000	-0.43695100	-0.04286100
H	-3.30681400	-1.22570300	-0.78719600
H	-3.61736300	-0.94589000	0.91578700
C	-4.70798300	0.36595000	-0.40998900
H	-5.58883200	-0.27812300	-0.47556700
H	-4.91578700	1.13910500	0.33616900
H	-4.58762100	0.86409500	-1.37674900

TS71

C	0.13042300	-0.77834300	0.86233300
C	-0.97460000	0.24519200	0.56577400
C	-0.44749300	1.38903700	-0.31899400
C	0.81520600	2.03952000	0.26443200
C	1.96314500	1.02359900	0.41986600
C	1.49139300	-0.16527400	1.23004400
H	-0.22797100	1.00607400	-1.31874900
H	-1.23585200	0.67196700	1.54578300

H	-0.18161500	-1.46595300	1.65820500
H	1.16876400	2.84873200	-0.38026000
H	2.83167000	1.48669400	0.89943700
H	2.03665900	-1.30470300	0.72944300
H	1.70648800	-0.09711500	2.29556400
H	-1.22112700	2.15318400	-0.42862400
H	0.60302300	2.47428100	1.24762200
O	0.41589300	-1.54538400	-0.32413300
O	1.64221500	-2.19484400	-0.00823100
C	-2.23117600	-0.45345500	0.00969300
H	-2.45702300	-1.31842600	0.64588100
H	-2.00334900	-0.85927900	-0.98175500
C	-3.47724700	0.43749400	-0.06674300
H	-3.29738500	1.27664700	-0.74650000
H	-3.66954700	0.87695700	0.92014600
O	2.31189300	0.70343600	-0.93398400
O	3.46978100	-0.16411200	-0.93554600
H	3.04273400	-1.02456400	-1.09073000
C	-4.71905000	-0.32739600	-0.53487200
H	-4.95719300	-1.15073700	0.14554500
H	-5.59361200	0.32664000	-0.58481800
H	-4.56652500	-0.75540900	-1.53022800

P71

C	-0.19725800	0.67713700	0.87388500
C	0.94193000	-0.30669000	0.50560900
C	0.43270100	-1.36036100	-0.49715300
C	-0.80495500	-2.11358800	0.01404300
C	-1.94577700	-1.16381900	0.40933600
C	-1.43651900	-0.05419000	1.27154500
H	0.19098500	-0.86743200	-1.44204400
H	1.17888500	-0.81920800	1.44880000
H	0.12783500	1.35505700	1.67110800
H	-1.17598600	-2.80477200	-0.74822400
H	-2.74987100	-1.70681500	0.91699100
H	-1.99873100	0.27102200	2.13721400
H	1.22688200	-2.08298400	-0.70313300
H	-0.54548000	-2.71185600	0.89462400
C	2.21189000	0.43098300	0.04113100
H	2.39580000	1.27125200	0.72286100
H	2.03502700	0.87400200	-0.94444800
O	-2.45691800	-0.68340100	-0.85642300
O	-3.50421600	0.28206100	-0.59567200
H	-2.98453600	1.10583300	-0.56458700
O	-0.38723800	1.46805400	-0.32678900

O	-1.51934100	2.36400600	-0.07571200
H	-1.24591400	3.11225200	-0.62331200
C	3.47167600	-0.44359600	-0.00507600
H	3.33168800	-1.26554100	-0.71454200
H	3.62533400	-0.90851600	0.97674300
C	4.72433300	0.34654300	-0.39621600
H	4.92169900	1.15353000	0.31623200
H	5.60845900	-0.29599600	-0.42428900
H	4.61150300	0.79989900	-1.38583500

R72

C	-1.00016600	-0.25336100	1.25086700
C	-2.11803700	-1.13124100	0.68529800
O	-0.15900900	0.71528200	-0.85579000
O	-0.74612500	1.86150200	-0.59552100
O	-2.62004400	-0.70976500	-0.57755200
O	-3.30247000	0.55189000	-0.38431900
H	-2.94003600	-1.18143700	1.40787700
H	-1.77771400	-2.15113500	0.48506700
H	-2.64105700	1.17938100	-0.72711000
H	-1.40605800	0.73203700	1.48823100
H	-0.67410300	-0.69446100	2.19893700
C	0.24539400	-0.05085300	0.37593500
C	1.30098300	0.78515100	1.11503900
C	0.82859100	-1.34151900	-0.20907800
C	2.59084900	0.99130700	0.31025700
H	1.53149000	0.26407500	2.05143900
H	0.85060800	1.74394600	1.38429400
C	2.13200000	-1.12094900	-0.99320600
H	1.01122900	-2.02505800	0.62881300
H	0.08121500	-1.81234800	-0.85175500
C	3.16948800	-0.34341800	-0.17513500
H	3.31911700	1.52474600	0.92836900
H	2.38243900	1.63349800	-0.55196700
H	2.53314500	-2.09120900	-1.30104800
H	1.90722800	-0.56952600	-1.91188900
H	4.06871600	-0.17096100	-0.77408900
H	3.48035900	-0.94352200	0.69042300

TS72

C	-0.13966100	0.13494600	0.33249200
C	-0.51057400	-1.34482400	0.16409200
C	-1.84670500	-1.57185500	-0.55415500
C	-2.98095800	-0.77942600	0.10109600
C	-2.66488200	0.73224000	0.06253700

C	-1.33905500	1.02916500	0.72681900
H	-1.75350800	-1.27195200	-1.60271300
H	-0.55541400	-1.78596800	1.16716100
H	-3.92846800	-0.96053800	-0.41499500
H	-3.46062800	1.31129300	0.53749700
H	-0.77415900	2.01692900	0.03342800
H	-1.38953700	1.29743700	1.78183500
H	-2.07692500	-2.64151600	-0.55057200
H	-3.11655300	-1.10710400	1.13924900
H	-2.62263500	1.03648700	-0.98875700
O	0.15798400	0.65218800	-1.00703500
O	0.09298300	2.06898400	-0.85763000
C	1.03881900	0.37439300	1.29366500
H	1.28443900	1.43931700	1.26340000
H	0.30463800	-1.84544900	-0.36388600
H	0.68395700	0.15692500	2.30862200
C	2.33148000	-0.42731000	1.08784900
H	2.16020300	-1.50810100	1.08159000
H	3.00885400	-0.19833200	1.91700900
O	3.09006900	-0.06551800	-0.05820200
O	2.53877400	-0.74576900	-1.21362100
H	1.86647200	-0.10248500	-1.50334600

P72

C	-0.24578300	0.20199600	0.39772900
C	-0.73096600	-1.26289600	0.37577800
C	-1.99447500	-1.46165600	-0.47425000
C	-3.12949700	-0.53940500	-0.01688100
C	-2.68876600	0.93677500	-0.04281600
C	-1.37879000	1.14642800	0.63937700
H	-1.76004000	-1.26302500	-1.52464500
H	-0.93766000	-1.55252700	1.41226500
H	-4.01074000	-0.67612000	-0.65056400
H	-3.45561200	1.57798500	0.40200400
H	-1.15987400	2.10358200	1.09931400
H	-2.30827200	-2.50830700	-0.41175900
H	-3.42728500	-0.80779900	1.00405100
H	-2.59888500	1.25635100	-1.09505500
C	0.89967700	0.45424900	1.39452800
H	1.11748300	1.52596800	1.39198400
H	0.07637000	-1.90729500	0.01831200
H	0.52539300	0.22170200	2.39779700
C	2.21266500	-0.30877200	1.19432500
H	2.09887400	-1.39093200	1.27460700
H	2.93126200	0.01587800	1.95337200

O	2.85420300	-0.02472200	-0.06157600
O	2.54045000	-1.08327200	-1.00222300
H	1.67864500	-0.76368000	-1.33861000
O	0.24607100	0.37805300	-0.99644700
O	0.83464000	1.68993600	-1.16445500
H	1.76304800	1.48793700	-0.94221500

R73

C	-0.87635900	-0.44122400	0.91683000
C	-2.00700300	-0.92204000	-0.00322300
O	0.29477100	1.09952100	-0.62074400
O	-0.19096200	2.14970200	0.00238100
O	-2.30536900	0.01409800	-1.05032300
O	-2.84830200	1.22152400	-0.46157600
H	-1.67295800	-1.78289900	-0.59169200
H	-2.07873500	1.81634900	-0.51091200
H	-1.21705800	0.43928900	1.46669300
H	-0.69082900	-1.22177600	1.66149600
C	0.47448300	-0.10054700	0.26854200
C	1.51058700	0.26968800	1.34149900
C	1.00063000	-1.16111500	-0.70511800
C	2.90001400	0.58073300	0.77006800
H	1.57967000	-0.57791800	2.03292800
H	1.12391200	1.11808000	1.91175500
C	2.40282500	-0.84680400	-1.25052300
H	1.01724000	-2.11701400	-0.16793700
H	0.29492300	-1.26839100	-1.53181900
C	3.40475400	-0.55558100	-0.12757600
H	3.59650100	0.75843600	1.59492300
H	2.85598200	1.51001100	0.19232600
H	2.74473200	-1.68644200	-1.86312900
H	2.34116100	0.02078000	-1.91540000
H	4.38071700	-0.29945200	-0.55070700
H	3.55329700	-1.46033600	0.47684000
C	-3.26073300	-1.30851400	0.77816000
H	-4.03702300	-1.64519500	0.08847600
H	-3.04442600	-2.11761500	1.48124400
H	-3.64340400	-0.45223800	1.33506200

TS73

C	0.37142500	0.10266100	-0.23115600
C	0.75455200	-1.34122400	0.12277800
C	2.19247000	-1.49666300	0.63337900
C	3.20383100	-0.86806900	-0.32867000
C	2.91172000	0.63980200	-0.49512700

C	1.49418400	0.87228000	-0.96825900
H	2.28443200	-1.02297000	1.61584700
H	0.62147100	-1.94490300	-0.78305300
H	4.22453400	-0.99635200	0.04368000
H	3.61896500	1.10155700	-1.18850400
H	1.06543600	1.98272400	-0.36890200
H	1.36546100	0.95842000	-2.04688700
H	2.40873000	-2.56004900	0.77364200
H	3.15550100	-1.36831900	-1.30381300
H	3.05387000	1.11577400	0.48107800
O	0.31500500	0.84770800	1.03032100
O	0.36453000	2.21575400	0.63226900
C	-0.94902300	0.22051700	-1.01482200
H	-1.17946400	1.28422200	-1.12430200
H	0.03851100	-1.71548600	0.85811000
H	-0.75765900	-0.17053500	-2.02098300
C	-2.20855800	-0.48666900	-0.47911300
H	-2.01240600	-1.54430600	-0.27679800
O	-2.71478200	0.10086100	0.72516700
O	-1.98051100	-0.41872600	1.85997600
H	-1.25886500	0.23293800	1.92390200
C	-3.36219400	-0.36320000	-1.47352700
H	-3.09543400	-0.81692200	-2.43108000
H	-4.24766300	-0.86462800	-1.08080600
H	-3.60981100	0.68744700	-1.64589800

P73

C	0.45927800	0.32326900	0.13299200
C	0.95732700	0.08672500	-1.30921300
C	2.34865800	-0.56061100	-1.36447800
C	3.37994600	0.25872000	-0.58196400
C	2.93483600	0.45870200	0.87955800
C	1.52621800	0.94002600	0.97992400
H	2.29388700	-1.57299500	-0.95246600
H	0.98678200	1.06227500	-1.80747000
H	4.35757500	-0.23161800	-0.60964200
H	3.60908200	1.14626400	1.39897400
H	1.22356900	1.54255700	1.82900300
H	2.65744300	-0.65982500	-2.40981300
H	3.50233400	1.23932700	-1.05783000
H	3.02141700	-0.50686500	1.40638400
C	-0.83801400	1.14993900	0.20520900
H	-1.06957600	1.32344100	1.26073400
H	0.22965200	-0.52540800	-1.84784800
H	-0.61790600	2.13299000	-0.22429400

C	-2.10054700	0.60024200	-0.47628200
H	-1.93796500	0.42701500	-1.54157000
O	-2.49353200	-0.67785200	0.08890800
O	-2.02906400	-1.75023100	-0.76782100
H	-1.12308000	-1.86718300	-0.41617000
O	0.21382500	-1.06769100	0.59925300
O	-0.35225700	-1.06292700	1.93129100
H	-1.30197500	-1.03693000	1.70781600
C	-3.29469500	1.52874100	-0.27748800
H	-3.08071300	2.52143600	-0.68187800
H	-4.17313300	1.12417500	-0.78205300
H	-3.52720500	1.63643700	0.78552300

R74

C	-0.57374700	-0.20470300	0.94049300
C	-1.76705000	-0.56256900	0.04498400
O	0.76573000	1.13156700	-0.65132200
O	0.42199500	2.25198400	-0.05700500
O	-1.95719700	0.38066100	-1.02171000
O	-2.33793800	1.66020400	-0.45706000
H	-1.55287400	-1.47249400	-0.52708200
H	-1.49907700	2.15076800	-0.52353000
H	-0.79290100	0.72638300	1.46841700
H	-0.47889800	-0.98189900	1.70537300
C	0.80216300	-0.05556600	0.27269300
C	1.88717200	0.21198400	1.32741600
C	1.18163400	-1.20209200	-0.67128200
C	3.29963100	0.32793100	0.73974800
H	1.85509200	-0.61694100	2.04372900
H	1.61622300	1.11853000	1.87450300
C	2.60726400	-1.08384400	-1.23357400
H	1.08248800	-2.13574900	-0.10466400
H	0.46093100	-1.24522200	-1.49097800
C	3.64862000	-0.88883900	-0.12587400
H	4.02008600	0.43985100	1.55554500
H	3.36859900	1.23819200	0.13467300
H	2.83452000	-1.97784000	-1.82211900
H	2.64957000	-0.23567500	-1.92460700
H	4.64497400	-0.77055500	-0.56227700
H	3.68771100	-1.78714100	0.50456800
C	-3.05046600	-0.77961700	0.85535300
H	-2.85009900	-1.54811400	1.61081000
H	-3.27837600	0.14407300	1.39386900
C	-4.24491200	-1.18927100	-0.00867900

H	-5.14169100	-1.31583100	0.60308400
H	-4.45153500	-0.43022400	-0.76465500
H	-4.05731100	-2.13592400	-0.52555300

TS74

C	0.71529400	0.03077900	-0.24523400
C	0.99517000	-1.16894800	0.67102300
C	2.44599800	-1.25366600	1.16142400
C	3.43919500	-1.19173500	-0.00190100
C	3.26893600	0.13490700	-0.77489600
C	1.84796200	0.30264200	-1.26471300
H	2.64668400	-0.42942500	1.85313500
H	0.74712200	-2.07551500	0.10609300
H	4.46815200	-1.26471000	0.36259700
H	3.96454900	0.19028900	-1.61587500
H	1.55834800	1.60053300	-1.17244300
H	1.65370500	-0.05001200	-2.27731000
H	2.57813600	-2.18066100	1.72752300
H	3.27972400	-2.04133300	-0.67734900
H	3.51860100	0.95288000	-0.09054100
O	0.80845300	1.23140600	0.59078600
O	0.94883200	2.29921500	-0.34314400
C	-0.63970100	-0.04802000	-0.97328400
H	-0.78050400	0.88828900	-1.52085800
H	0.30234900	-1.12499500	1.51468000
H	-0.55257500	-0.84611900	-1.71971500
C	-1.91436200	-0.31858800	-0.15281100
H	-1.79869300	-1.20540800	0.47938800
O	-2.26904100	0.77957400	0.69620900
O	-1.52920700	0.69287300	1.93781400
H	-0.73879900	1.21622600	1.71173000
C	-3.12812400	-0.50864500	-1.07284900
H	-2.86891900	-1.25653600	-1.82982900
H	-3.30221900	0.43121700	-1.60854900
C	-4.39594600	-0.93062900	-0.32736200
H	-4.65282800	-0.19820600	0.43885300
H	-5.24054500	-1.02055000	-1.01538300
H	-4.26111600	-1.89909900	0.16413900

P74

C	0.79098100	-0.17538900	-0.29440400
C	1.19419400	-0.80255700	1.05831800
C	2.61995000	-0.43041700	1.48968600
C	3.64701700	-0.79937700	0.41422500
C	3.29975400	-0.13664700	-0.93264600
C	1.87248100	-0.34710500	-1.31287100

H	2.66856700	0.64425800	1.69080200
H	1.11586200	-1.89025200	0.94962000
H	4.65177500	-0.50135400	0.72833300
H	3.96169500	-0.49862900	-1.72497700
H	1.59345800	-0.36934900	-2.36033600
H	2.85535400	-0.94011700	2.42924400
H	3.66417000	-1.88851300	0.28555000
H	3.49093800	0.94668300	-0.84765500
C	-0.55318400	-0.69700600	-0.83514100
H	-0.71226500	-0.25767200	-1.82495700
H	0.47329500	-0.50573000	1.82441700
H	-0.43875400	-1.77429200	-0.99458800
C	-1.82203700	-0.46730600	-0.00034600
H	-1.73477400	-0.90941700	0.99480700
O	-2.06898400	0.94867000	0.20254200
O	-1.60684800	1.34352200	1.51803500
H	-0.66844000	1.53459600	1.31570700
O	0.68506100	1.26389800	0.06223500
O	0.22994500	2.03520800	-1.07469600
H	-0.73405300	1.99169600	-0.92865200
C	-3.06729700	-1.01787300	-0.70249600
H	-2.86135300	-2.05121500	-1.00220000
H	-3.21885000	-0.45185700	-1.62888200
C	-4.32764900	-0.95700700	0.16224500
H	-4.52326200	0.06704100	0.48442400
H	-5.19902200	-1.31442800	-0.39214800
H	-4.22296900	-1.57774900	1.05715800

R75

C	-0.93781600	-0.08791300	0.22952200
C	-1.57848600	-1.42173900	-0.17075600
C	-2.98308500	-1.26963200	-0.77486500
C	-3.90556900	-0.44994800	0.13545000
C	-3.29035800	0.92038200	0.44600500
C	-1.88906000	0.78666000	1.05752700
H	-2.90511300	-0.77881000	-1.75040400
H	-0.91932800	-1.94747800	-0.86640400
H	-1.63275500	-2.03390500	0.73725000
H	-4.88594900	-0.32635100	-0.33436700
H	-3.93091100	1.48243400	1.13213200
H	-1.42513200	1.76794700	1.18776000
H	-1.96129100	0.33322400	2.05303300
H	-3.40605000	-2.26233800	-0.95639900
H	-4.07465000	-0.99609600	1.07301800

H	-3.22855500	1.50802900	-0.47608200
O	-0.74163900	0.59977700	-1.10101700
O	-0.09270200	1.73717700	-1.02692300
C	0.42805700	-0.22770800	0.90765200
H	0.26569600	-0.74665600	1.85801300
H	0.77651900	0.77169400	1.16321300
C	1.50631500	-0.96278500	0.08453700
H	1.34663900	-0.80654600	-0.98656100
H	1.42848100	-2.04103800	0.25511200
C	2.95027500	-0.51993700	0.38013700
H	3.08084800	-0.30793600	1.44813700
O	3.31387700	0.66320300	-0.35169500
O	2.66936500	1.82194800	0.23677200
H	1.90682000	1.94460700	-0.35292500
C	3.97827200	-1.55210100	-0.07478000
H	4.98933600	-1.17765600	0.09178300
H	3.85105000	-2.48225700	0.48390300
H	3.86187600	-1.76939600	-1.13987500

TS75

C	-0.88539400	-0.21068800	0.17495000
C	-1.64110400	-1.47732500	-0.24854200
C	-3.04264200	-1.19637300	-0.80545200
C	-3.86815800	-0.34506200	0.16463600
C	-3.16910300	1.01036200	0.42759500
C	-1.76614200	0.79969600	0.94763900
H	-2.95765200	-0.67597400	-1.76427700
H	-1.71930200	-2.12153100	0.63455600
H	-4.86704500	-0.15734200	-0.24020100
H	-3.74772600	1.61679600	1.12857900
H	-1.00151600	1.76316600	0.42223500
H	-1.66038400	0.77274300	2.03228200
H	-3.55038700	-2.14556500	-1.00253500
H	-4.00452300	-0.88254300	1.11112800
H	-3.12963500	1.55332200	-0.52270100
O	-0.61010100	0.53139700	-1.04630800
O	-0.23934000	1.82369100	-0.57621800
C	0.41306300	-0.49914400	0.94958800
H	0.76501300	0.44124500	1.37797600
H	-1.03148000	-2.01918400	-0.97839400
H	0.17232000	-1.15538900	1.79245900
C	1.54563300	-1.08872200	0.09142900
H	1.54743600	-2.18224000	0.12704200
H	1.39755900	-0.80130100	-0.95177100
C	2.92975500	-0.58071000	0.52141900

H	3.11559400	-0.85316900	1.56832000
O	2.96089300	0.85442300	0.61682800
O	2.69283300	1.41973000	-0.69551200
H	1.76168200	1.69194800	-0.59433100
C	4.05926900	-1.10713500	-0.35696100
H	3.92251500	-0.77234700	-1.38651900
H	5.02105500	-0.73528000	0.00191300
H	4.08112600	-2.20068600	-0.34387800

P75

C	0.83580300	-0.15139300	-0.41140600
C	1.31530600	-1.27012900	0.53963500
C	2.60421900	-0.90795000	1.29211600
C	3.73272900	-0.51783400	0.33236600
C	3.29789900	0.63974100	-0.58681300
C	1.97643000	0.37625100	-1.22616600
H	2.39977400	-0.07663800	1.97363100
H	1.47719000	-2.16847900	-0.06639400
H	4.62837400	-0.23419300	0.89335000
H	4.05756900	0.83522800	-1.34943800
H	1.76113000	0.78379500	-2.20721700
H	2.90953700	-1.75829500	1.91025000
H	4.00409400	-1.38377400	-0.28377800
H	3.22791200	1.55970800	0.01793700
C	-0.32537000	-0.58114300	-1.32969800
H	-0.58290600	0.27950700	-1.95559600
H	0.52185100	-1.50753700	1.25398200
H	0.09472300	-1.32658600	-2.01197900
C	-1.60535300	-1.15594300	-0.68238700
H	-1.97346100	-1.97477700	-1.30820300
H	-1.39744800	-1.60224000	0.29271300
C	-2.77563600	-0.17626700	-0.52874300
H	-2.97507000	0.30100500	-1.49647000
O	-2.44679700	0.98033300	0.27098800
O	-2.12961300	0.55288400	1.62320600
H	-1.15324000	0.59642100	1.57454700
O	0.39655700	0.88460600	0.54785700
O	0.05175100	2.10196600	-0.16019200
H	-0.92122000	2.00928500	-0.17202500
C	-4.04832600	-0.83736500	-0.01133700
H	-4.84859200	-0.09910500	0.06754500
H	-4.37215000	-1.63107600	-0.68978300
H	-3.87947900	-1.26837100	0.97665600

R76

C	0.64197300	0.14896100	0.22522200
C	1.45126400	1.37459000	-0.21344200
C	2.82478700	1.02119900	-0.80460900
C	3.63129800	0.11887200	0.13712800
C	2.84243500	-1.14883300	0.48847000
C	1.46954400	-0.81382300	1.08724400
H	2.68456800	0.51269300	-1.76396300
H	0.86722700	1.95910500	-0.92907000
H	1.58357600	2.00343400	0.67493100
H	4.58794000	-0.14662600	-0.32269900
H	3.40327800	-1.76639500	1.19629200
H	0.88168600	-1.72156600	1.24691200
H	1.59879800	-0.34069700	2.06769900
H	3.37369800	1.94383400	-1.01602700
H	3.86845400	0.66891400	1.05748400
H	2.70601600	-1.75365700	-0.41426700
O	0.36017500	-0.55077000	-1.08390700
O	-0.42599000	-1.59490500	-0.97528400
C	-0.69625200	0.48680600	0.88779200
H	-0.47220200	1.00739900	1.82443300
H	-1.17372400	-0.45106600	1.16806000
C	-1.66700400	1.33026800	0.03366600
H	-1.49718900	1.15206200	-1.03206500
H	-1.48270800	2.39657000	0.20168800
C	-3.15021300	1.05002800	0.29884400
H	-3.36702300	0.94491000	1.36812300
O	-3.66407000	-0.07897100	-0.40465200
O	-3.19940400	-1.29265300	0.24533200
H	-2.45283600	-1.54481300	-0.32330300
H	-3.76224800	1.86588700	-0.09803900

TS76

C	0.58600200	0.26683200	0.13850700
C	1.48165900	1.40758300	-0.36269900
C	2.86004100	0.93642700	-0.84392700
C	3.56508000	0.09320800	0.22350200
C	2.72049700	-1.15498800	0.57524800
C	1.33414300	-0.75664600	1.02513300
H	2.74609300	0.34539600	-1.75773700
H	1.60398500	2.11487300	0.46536800
H	4.54903500	-0.23087200	-0.12820200
H	3.21321900	-1.75333000	1.34540600
H	0.48639800	-1.67887200	0.55572800
H	1.20301900	-0.62328600	2.09909800
H	3.47009200	1.80745300	-1.10225600

H	3.73208400	0.69488000	1.12539100
H	2.64954900	-1.77269700	-0.32630400
O	0.26872100	-0.54740000	-1.02502000
O	-0.24950700	-1.74844700	-0.46197100
C	-0.69635700	0.75403400	0.83784200
H	-1.14818500	-0.10238300	1.34217200
H	0.95187400	1.94335400	-1.15664600
H	-0.41414000	1.46563300	1.62059800
C	-1.74508800	1.35611700	-0.11439500
H	-1.66436200	2.44615900	-0.17215700
H	-1.58177600	0.96821600	-1.12134500
C	-3.17437400	0.99259900	0.29733100
H	-3.43888200	1.42388300	1.26922700
O	-3.35742000	-0.40353200	0.51986800
O	-3.13836800	-1.09013900	-0.74078600
H	-2.24173900	-1.44931600	-0.60431200
H	-3.89328400	1.34605800	-0.44999700

P76

C	-0.64685000	-0.17208000	0.20742100
C	-1.43789800	-1.24931000	-0.56525600
C	-2.80402700	-0.75902400	-1.06467600
C	-3.65497700	-0.20301600	0.08216200
C	-2.91633200	0.93050500	0.82101700
C	-1.52004600	0.54649200	1.18625100
H	-2.65609900	0.01594800	-1.82394700
H	-1.57633300	-2.09564500	0.11688400
H	-4.61544200	0.16179800	-0.29430800
H	-3.47198400	1.23825000	1.71177200
H	-1.07000800	0.93362600	2.09370000
H	-3.32701800	-1.58539900	-1.55628500
H	-3.87692400	-1.00742900	0.79367100
H	-2.89864900	1.81418000	0.15858200
C	0.62449100	-0.71910100	0.87557300
H	1.09690400	0.11486600	1.39984600
H	-0.82832400	-1.60548700	-1.39961000
H	0.31851500	-1.43891500	1.64193900
C	1.65044900	-1.34159700	-0.08753400
H	1.50062300	-2.42136200	-0.18945900
H	1.52907200	-0.91028400	-1.08285200
C	3.09462100	-1.09171600	0.34951200
H	3.29536600	-1.50783000	1.34315300
O	3.39860800	0.29048700	0.52405100
O	3.30524100	0.93448100	-0.77483800
H	2.43275800	1.36290000	-0.70472500

H	3.79781800	-1.53509000	-0.36426400
O	-0.26536200	0.74018900	-0.89702300
O	0.56104400	1.81402100	-0.36490400
H	0.01688400	2.58552400	-0.56920100

R77

C	2.42172000	-1.05183200	-0.43829900
C	0.94258800	-1.45688100	-0.51874500
C	0.12739000	-0.85996700	0.64336200
C	0.29620000	0.66596300	0.70142700
C	1.76627100	1.08437500	0.77103500
C	2.59312700	0.47224500	-0.37015600
H	0.50884100	-1.12235000	-1.46600000
H	0.84988800	-2.54751300	-0.49885300
H	2.87202500	-1.50822400	0.45311400
H	2.96567000	-1.45106400	-1.29944500
H	-0.29447200	1.11237200	1.50229000
H	1.82568800	2.17623200	0.75760900
H	2.15786900	0.75444800	1.74064500
H	2.27623000	0.91706400	-1.31904500
H	3.64738600	0.73229100	-0.23819000
H	0.56665600	-1.22687100	1.58380200
C	-1.32907500	-1.34612900	0.66816600
H	-1.85791800	-0.98549900	1.55284400
O	-2.09103000	-0.98168900	-0.48633900
O	-3.04060500	0.04276300	-0.11371100
H	-2.62758100	0.84238000	-0.48511700
O	-0.25315300	1.24274700	-0.54438900
O	-1.17385600	2.15895900	-0.31982700
H	-1.33045400	-2.44214600	0.68972300

TS77

C	-2.08046400	1.19035900	-0.54973900
C	-0.56436100	1.48742400	-0.56618200
C	0.18746400	0.89190900	0.64327700
C	-0.21788200	-0.56426600	0.95587400
C	-1.74388600	-0.68088700	1.12829100
C	-2.37845900	-0.24548700	-0.16932300
H	-0.12275900	1.08676300	-1.47829900
H	-0.40893300	2.57098100	-0.58006000
H	-2.57525800	1.84712500	0.18325600
H	-2.51794400	1.43357900	-1.52183700
H	0.31306900	-0.91628100	1.84435600
H	-2.00617300	-1.70909100	1.38618700
H	-2.06041000	-0.03387100	1.95892800

H	-1.56778800	-0.95734200	-0.97043100
H	-3.38692300	-0.60725800	-0.36662000
H	-0.13494300	1.44256700	1.54124500
C	1.70283800	1.14756300	0.56225700
H	2.20455400	0.85391500	1.48973600
O	0.15064700	-1.57400700	-0.01673100
O	-0.45825800	-1.33012900	-1.28097500
O	2.37128700	0.53241900	-0.53100400
O	2.80438500	-0.78707900	-0.11828500
H	2.02097500	-1.32030200	-0.34377600
H	1.86656000	2.21989000	0.40654900

P77

C	2.14022900	-1.39462300	-0.57333100
C	0.60746400	-1.56215700	-0.62007600
C	-0.07132200	-0.93028400	0.60882900
C	0.30458900	0.55958700	0.73291500
C	1.82660500	0.73589500	0.84582500
C	2.55902300	-0.00393100	-0.22576400
H	0.20691800	-1.10128600	-1.52535900
H	0.35410100	-2.62590000	-0.66701600
H	2.53806400	-2.09664100	0.18417500
H	2.58464700	-1.70612600	-1.52345500
H	-0.20281300	1.01406700	1.59013300
H	2.06813000	1.80111700	0.84003300
H	2.11789500	0.37070900	1.84763600
H	3.51442300	0.37223900	-0.57401900
H	0.36024800	-1.39263500	1.50972400
C	-1.57340400	-1.25473200	0.69376300
H	-2.01880000	-0.83493800	1.60134300
O	-2.35586500	-0.86050900	-0.42564600
O	-2.82555600	0.49226900	-0.20754100
H	-2.05267500	0.99991700	-0.51691000
H	-1.68669300	-2.34440100	0.72513600
O	-0.19234800	1.20576000	-0.46207100
O	-0.01954800	2.64124200	-0.30925600
H	0.60399700	2.81525600	-1.02827200

R78

C	2.42172000	-1.05183200	-0.43829900
C	0.94258800	-1.45688100	-0.51874500
C	0.12739000	-0.85996700	0.64336200
C	0.29620000	0.66596300	0.70142700
C	1.76627100	1.08437500	0.77103500
C	2.59312700	0.47224500	-0.37015600

H	0.50884100	-1.12235000	-1.46600000
H	0.84988800	-2.54751300	-0.49885300
H	2.87202500	-1.50822400	0.45311400
H	2.96567000	-1.45106400	-1.29944500
H	-0.29447200	1.11237200	1.50229000
H	1.82568800	2.17623200	0.75760900
H	2.15786900	0.75444800	1.74064500
H	2.27623000	0.91706400	-1.31904500
H	3.64738600	0.73229100	-0.23819000
H	0.56665600	-1.22687100	1.58380200
C	-1.32907500	-1.34612900	0.66816600
H	-1.85791800	-0.98549900	1.55284400
O	-2.09103000	-0.98168900	-0.48633900
O	-3.04060500	0.04276300	-0.11371100
H	-2.62758100	0.84238000	-0.48511700
O	-0.25315300	1.24274700	-0.54438900
O	-1.17385600	2.15895900	-0.31982700
H	-1.33045400	-2.44214600	0.68972300

TS78

C	-2.08054900	1.21551800	-0.50985400
C	-0.57115400	1.20979300	-0.64877600
C	0.14562900	0.81442100	0.62361500
C	-0.24210700	-0.66432000	0.88966000
C	-1.76006200	-0.79822300	1.07969400
C	-2.61977000	-0.15125600	-0.02725400
H	-0.31923600	0.08118100	-1.35097100
H	-0.12274100	2.01254900	-1.23353700
H	-2.35316800	1.99977700	0.21341200
H	-2.55957600	1.48965900	-1.45363400
H	0.28591200	-1.06075600	1.76121700
H	-2.01013500	-1.85823600	1.17448900
H	-1.98860200	-0.33378100	2.04675500
H	-2.65741600	-0.82936800	-0.87907200
H	-3.64471700	-0.03247800	0.33600100
H	-0.29373900	1.38238100	1.46108500
C	1.64537500	1.15521800	0.64859700
O	0.28990900	-1.47932400	-0.17736200
O	-0.29071100	-1.12376300	-1.42449100
H	2.09734900	0.86545500	1.60243000
O	2.42069800	0.61856100	-0.41218100
O	2.90083400	-0.69012800	-0.01833800
H	2.14493200	-1.24596800	-0.28620300
H	1.75112600	2.23991000	0.53290600

P78

C	-2.27936700	1.10734800	-0.51627300
C	-0.82460200	1.43336200	-0.46179000
C	-0.00020200	0.90239400	0.66996500
C	-0.27769000	-0.60466600	0.92692000
C	-1.77811900	-0.91898100	0.93868700
C	-2.54358900	-0.39574800	-0.28527600
H	-0.39927300	2.17734400	-1.12577600
H	-2.81431800	1.67641800	0.26732200
H	-2.71315600	1.42758900	-1.46741500
H	0.15672600	-0.88585300	1.89356100
H	-1.89768700	-2.00146500	1.03596100
H	-2.19591100	-0.47063400	1.84985700
H	-2.23365100	-0.95347500	-1.16938700
H	-3.61530300	-0.56679800	-0.14372300
H	-0.34284500	1.39122900	1.60441400
C	1.48670300	1.27329200	0.56028000
H	2.03177200	1.05577500	1.48034900
O	2.17458600	0.61366900	-0.51709600
O	2.99032200	-0.45480200	0.02645900
H	2.32063900	-1.16329000	0.10329400
H	1.57320700	2.34419200	0.34963100
O	0.46715300	-1.48787100	0.05823800
O	0.19169600	-1.21033300	-1.33866000
H	0.84094200	-0.49833800	-1.50468200

R79

C	2.28433000	-1.38460300	-0.62618900
C	0.75156300	-1.35651000	-0.69813000
C	0.12746500	-0.67537600	0.53648100
C	0.74414300	0.71409200	0.79564600
C	2.27168800	0.70849600	0.79274000
C	2.86017500	0.02530300	-0.44893100
H	0.43365900	-0.81903100	-1.59514800
H	0.36222400	-2.37424900	-0.78843700
H	2.60358000	-2.01702300	0.21295000
H	2.68763500	-1.84482700	-1.53309300
H	0.34875800	1.14240200	1.71822400
H	2.63062200	1.73718000	0.88666600
H	2.59436400	0.17594500	1.69546400
H	2.63274400	0.62687600	-1.33549500
H	3.95026800	-0.01086800	-0.36430400
H	0.39638000	-1.26539700	1.42421800
C	-1.41518100	-0.61827700	0.49724800
H	-1.75670900	0.14222200	1.20455700

O	-1.78416500	-0.16457200	-0.81980400
O	-2.92600900	0.71234700	-0.70877900
H	-2.47300600	1.57366300	-0.70099500
O	0.32095500	1.65762200	-0.26129900
O	-0.69147400	2.40969200	0.11358800
C	-2.10695300	-1.94240900	0.80700400
H	-1.88104500	-2.27585200	1.82447000
H	-1.80006800	-2.72285300	0.10709800
H	-3.18724700	-1.81319800	0.71896700

TS79

C	-1.89991600	1.53591500	-0.64212900
C	-0.36459000	1.36855500	-0.71470900
C	0.23333600	0.68662500	0.53532400
C	-0.57262200	-0.55148300	0.98716700
C	-2.05524600	-0.19431400	1.20100500
C	-2.59373300	0.29552200	-0.11972500
H	-0.10210900	0.77505500	-1.59024200
H	0.08738000	2.35516400	-0.84228400
H	-2.14488900	2.37254800	0.03142800
H	-2.28830200	1.81307200	-1.62603100
H	-0.12958100	-0.97023800	1.89488000
H	-2.59542600	-1.07185500	1.56261000
H	-2.12772000	0.58709300	1.97099900
H	-2.06918100	-0.69197900	-0.86468000
H	-3.67233200	0.23759800	-0.26180500
H	0.11430600	1.37973100	1.38275700
C	1.76117800	0.44572500	0.43932900
H	2.09922800	-0.02203100	1.36992900
O	-0.56666900	-1.70555700	0.10941500
O	-1.13538300	-1.40256100	-1.16036400
C	2.55883400	1.72741600	0.20055100
H	3.62415800	1.51888800	0.30713200
H	2.39201500	2.11146600	-0.80763900
H	2.27851400	2.50321600	0.91838500
O	2.13137600	-0.43603000	-0.62900300
O	2.17969800	-1.79399100	-0.13164900
H	1.25831000	-2.07638800	-0.27350200

P79

C	1.61751900	-1.97737500	-0.70475500
C	0.17120600	-1.45082900	-0.81953700
C	-0.26267500	-0.70587200	0.45716000
C	0.71273100	0.45207000	0.75543500
C	2.14635300	-0.06736200	0.94259900
C	2.57070700	-0.95428100	-0.18183800

H	0.08446900	-0.77475300	-1.67238800
H	-0.50097300	-2.29061300	-1.00989800
H	1.60956000	-2.85091800	-0.02507100
H	1.95617000	-2.36233500	-1.67155400
H	0.38584600	1.00987600	1.63930000
H	2.82446700	0.77963500	1.06891200
H	2.16744400	-0.61095400	1.90481400
H	3.61997500	-1.01042600	-0.44942600
H	-0.12768000	-1.38708500	1.31010700
C	-1.76359400	-0.31963500	0.48966800
H	-1.94935600	0.29385300	1.37743100
O	-2.18009600	0.44808000	-0.64758400
O	-2.04094900	1.85789100	-0.35306100
H	-1.09612000	1.98802900	-0.55585400
O	0.64572600	1.34977200	-0.37662100
O	1.41140000	2.54375700	-0.05816700
H	2.11029000	2.48065300	-0.72425900
C	-2.69010600	-1.53558200	0.51575800
H	-2.40963300	-2.22363000	1.31771300
H	-3.71742100	-1.20708600	0.67845400
H	-2.65853100	-2.07705300	-0.43175900

R80

C	2.28433000	-1.38460300	-0.62618900
C	0.75156300	-1.35651000	-0.69813000
C	0.12746500	-0.67537600	0.53648100
C	0.74414300	0.71409200	0.79564600
C	2.27168800	0.70849600	0.79274000
C	2.86017500	0.02530300	-0.44893100
H	0.43365900	-0.81903100	-1.59514800
H	0.36222400	-2.37424900	-0.78843700
H	2.60358000	-2.01702300	0.21295000
H	2.68763500	-1.84482700	-1.53309300
H	0.34875800	1.14240200	1.71822400
H	2.63062200	1.73718000	0.88666600
H	2.59436400	0.17594500	1.69546400
H	2.63274400	0.62687600	-1.33549500
H	3.95026800	-0.01086800	-0.36430400
H	0.39638000	-1.26539700	1.42421800
C	-1.41518100	-0.61827700	0.49724800
H	-1.75670900	0.14222200	1.20455700
O	-1.78416500	-0.16457200	-0.81980400
O	-2.92600900	0.71234700	-0.70877900
H	-2.47300600	1.57366300	-0.70099500

O	0.32095500	1.65762200	-0.26129900
O	-0.69147400	2.40969200	0.11358800
C	-2.10695300	-1.94240900	0.80700400
H	-1.88104500	-2.27585200	1.82447000
H	-1.80006800	-2.72285300	0.10709800
H	-3.18724700	-1.81319800	0.71896700

TS80

C	-1.94102000	1.42187600	-0.70805600
C	-0.49146300	0.99905000	-0.84737500
C	0.15532800	0.64722000	0.47474500
C	-0.58460000	-0.62206000	0.97860300
C	-2.07694200	-0.32761400	1.19063500
C	-2.79146300	0.33812800	-0.00483700
H	-0.57233900	-0.25301700	-1.34926600
H	0.11402000	1.54258100	-1.57008600
H	-1.97032900	2.35503600	-0.12451300
H	-2.37546000	1.65638300	-1.68357700
H	-0.13794200	-0.99582900	1.90432400
H	-2.58440100	-1.25866500	1.45691400
H	-2.13703900	0.32542700	2.06983500
H	-3.04121200	-0.43259000	-0.73318600
H	-3.73364400	0.77652800	0.33689500
H	-0.10411100	1.42911200	1.20697200
C	1.70084400	0.57589300	0.46719700
O	-0.32523600	-1.70752300	0.06336300
O	-0.85624400	-1.42106600	-1.22306800
H	2.04141900	0.17062400	1.42552900
O	2.22397600	-0.27120400	-0.56170700
O	2.40519200	-1.60757300	-0.03793900
H	1.50984400	-1.97393500	-0.16479900
C	2.34280300	1.94446300	0.24062900
H	1.98948900	2.66681500	0.98117500
H	3.42671200	1.85710400	0.32389900
H	2.11257000	2.33160900	-0.75456800

P80

C	-2.12569000	1.42639900	-0.63868700
C	-0.63715200	1.32093800	-0.65379100
C	0.03978400	0.75043000	0.55301000
C	-0.61747400	-0.59559300	0.98638200
C	-2.14828800	-0.50554800	1.01754300
C	-2.78161400	0.07882200	-0.25369500
H	-0.07161800	1.54542800	-1.54880800
H	-2.43658000	2.18188200	0.10400200
H	-2.50446500	1.76968600	-1.60446700

H	-0.25449800	-0.85753700	1.98794800
H	-2.54312000	-1.50442800	1.22259600
H	-2.41610100	0.12303100	1.87714000
H	-2.65911700	-0.62596200	-1.07663300
H	-3.85529600	0.22076000	-0.09330800
H	-0.16738500	1.41841800	1.40985500
C	1.57674500	0.67448000	0.45616300
H	1.97276300	0.23266700	1.37315400
O	1.97886000	-0.21542400	-0.62190400
O	2.58082600	-1.40950700	-0.06569400
H	1.77691500	-1.91882400	0.15736000
O	-0.13816300	-1.73887400	0.24735200
O	-0.39669200	-1.58864200	-1.17286700
H	0.39625000	-1.07996000	-1.43512300
C	2.24585600	2.02258700	0.21186500
H	1.97630100	2.73233600	0.99893600
H	3.32998400	1.89902800	0.20653000
H	1.94431100	2.44795700	-0.74701200

R81

C	2.27339400	-1.73912000	-0.63406600
C	0.79252800	-1.36716800	-0.79100700
C	0.24527200	-0.63906500	0.45337100
C	1.12644500	0.56709000	0.83120400
C	2.61153500	0.22016000	0.92959900
C	3.13054800	-0.50812200	-0.31803900
H	0.66176100	-0.71971100	-1.66216400
H	0.20116300	-2.26729800	-0.97791700
H	2.38383400	-2.47651100	0.17218400
H	2.63166700	-2.22197900	-1.54806600
H	0.76434800	1.04128300	1.74474100
H	3.17846100	1.13600500	1.11862000
H	2.73699100	-0.41916200	1.81180200
H	3.11211400	0.17883900	-1.17079000
H	4.17555200	-0.79407300	-0.16665200
H	0.33132300	-1.32042600	1.31186200
C	-1.24928500	-0.26097100	0.34152500
H	-1.49018900	0.48388800	1.10531300
O	-1.45169500	0.37392400	-0.94031500
O	-2.21725500	1.58602900	-0.76830500
H	-1.49985100	2.24450800	-0.72293400
O	1.00062700	1.61311900	-0.20545700
O	0.24495800	2.61916500	0.18353400
C	-2.19991400	-1.45483900	0.48396700

H	-1.97701000	-1.96363100	1.42973100
H	-1.99126000	-2.17173800	-0.31654900
C	-3.67620100	-1.05381200	0.44104400
H	-3.90946000	-0.53870300	-0.49214900
H	-4.31950700	-1.93434100	0.51696600
H	-3.92895300	-0.37977000	1.26476000

TS81

C	1.98199700	-1.76193400	-0.64854000
C	0.50035700	-1.33573400	-0.76377500
C	-0.02027500	-0.59324100	0.48669100
C	0.96668900	0.47683700	1.00435100
C	2.35951300	-0.13160800	1.25206400
C	2.85458700	-0.66999000	-0.06672900
H	0.37365100	-0.68455900	-1.62841800
H	-0.10666700	-2.22779500	-0.93601400
H	2.05909000	-2.64483600	0.00587500
H	2.35333200	-2.07568200	-1.62806300
H	0.56805500	0.93897500	1.91163600
H	3.02610900	0.63225500	1.65805500
H	2.27229600	-0.93388100	1.99866100
H	2.52815100	0.41064000	-0.79572400
H	3.93205600	-0.78960600	-0.17480100
H	-0.05474200	-1.31774300	1.31522200
C	-1.47769300	-0.08927600	0.34445500
H	-1.76945200	0.40804600	1.27648100
C	-2.48158400	-1.20887200	0.03964600
H	-2.32862100	-1.54628200	-0.98992300
H	-2.26232100	-2.06492800	0.68802000
C	-3.93379100	-0.76231700	0.22230900
H	-4.14098700	0.11497000	-0.39282500
H	-4.62763100	-1.55582300	-0.06697400
O	1.18660400	1.63914600	0.16633900
O	1.74067800	1.27910100	-1.09508500
H	-4.14023400	-0.50068600	1.26461700
O	-1.63996200	0.87057500	-0.70899000
O	-1.49345400	2.20606700	-0.17091000
H	-0.53182500	2.33464300	-0.25949100

P81

C	1.70832300	-2.14716800	-0.69428500
C	0.36446400	-1.41364200	-0.89091500
C	-0.04648900	-0.63529800	0.37326500
C	1.06338700	0.35976800	0.77319400
C	2.38976600	-0.36775200	1.03961100
C	2.75984700	-1.28223800	-0.08183900

H	0.43525300	-0.71782300	-1.72932800
H	-0.40758900	-2.14288400	-1.14693500
H	1.52686000	-3.02079100	-0.03936800
H	2.05445100	-2.56157800	-1.64608400
H	0.75876400	0.93925300	1.65114700
H	3.17237400	0.36790700	1.23853500
H	2.26320000	-0.92973100	1.98294800
H	3.80619700	-1.48193400	-0.28415700
H	-0.07651800	-1.34414600	1.21373300
C	-1.47012800	-0.02733700	0.31067900
H	-1.62810200	0.59364900	1.19970000
C	-2.57648500	-1.08896100	0.24129100
H	-2.54980600	-1.56367800	-0.74431000
H	-2.35738400	-1.87294600	0.97485200
C	-3.96896100	-0.50479300	0.48999400
H	-4.17544900	0.29892500	-0.21867500
H	-4.74145600	-1.26983300	0.37655500
H	-4.05111100	-0.09324300	1.50063800
O	-1.67396900	0.81497000	-0.83275900
O	-1.36912100	2.18637300	-0.48631300
H	-0.40133800	2.18431800	-0.60593600
O	1.20749000	1.28337800	-0.33025800
O	2.10972200	2.34707100	0.08090400
H	2.83633200	2.20472400	-0.54186900

R82

C	2.27339400	-1.73912000	-0.63406600
C	0.79252800	-1.36716800	-0.79100700
C	0.24527200	-0.63906500	0.45337100
C	1.12644500	0.56709000	0.83120400
C	2.61153500	0.22016000	0.92959900
C	3.13054800	-0.50812200	-0.31803900
H	0.66176100	-0.71971100	-1.66216400
H	0.20116300	-2.26729800	-0.97791700
H	2.38383400	-2.47651100	0.17218400
H	2.63166700	-2.22197900	-1.54806600
H	0.76434800	1.04128300	1.74474100
H	3.17846100	1.13600500	1.11862000
H	2.73699100	-0.41916200	1.81180200
H	3.11211400	0.17883900	-1.17079000
H	4.17555200	-0.79407300	-0.16665200
H	0.33132300	-1.32042600	1.31186200
C	-1.24928500	-0.26097100	0.34152500
H	-1.49018900	0.48388800	1.10531300

O	-1.45169500	0.37392400	-0.94031500
O	-2.21725500	1.58602900	-0.76830500
H	-1.49985100	2.24450800	-0.72293400
O	1.00062700	1.61311900	-0.20545700
O	0.24495800	2.61916500	0.18353400
C	-2.19991400	-1.45483900	0.48396700
H	-1.97701000	-1.96363100	1.42973100
H	-1.99126000	-2.17173800	-0.31654900
C	-3.67620100	-1.05381200	0.44104400
H	-3.90946000	-0.53870300	-0.49214900
H	-4.31950700	-1.93434100	0.51696600
H	-3.92895300	-0.37977000	1.26476000

TS82

C	-2.19829800	-1.71917900	0.40693300
C	-0.83518300	-1.18095400	0.79047300
C	-0.07429400	-0.54167100	-0.34764900
C	-0.91339400	0.68083000	-0.79697100
C	-2.32630600	0.26516500	-1.22458400
C	-3.08632900	-0.62010800	-0.21764400
H	-1.10988700	-0.02005800	1.43879800
H	-0.25260800	-1.77662300	1.49382600
H	-2.05497100	-2.53650000	-0.31684000
H	-2.70135200	-2.15942000	1.27210700
H	-0.40934200	1.21041700	-1.60674200
H	-2.90391600	1.16743400	-1.44288800
H	-2.21116700	-0.27066200	-2.17491600
H	-3.47533700	0.01040000	0.58198300
H	-3.94599800	-1.07962100	-0.71391500
H	-0.06934900	-1.22660200	-1.21069300
C	1.39642900	-0.21117400	0.02585400
H	1.46239500	0.01161400	1.09428000
C	2.34223600	-1.36184500	-0.32780800
H	2.36506500	-1.45469300	-1.42016600
H	1.91237300	-2.29578700	0.05340000
C	3.75961000	-1.16741900	0.21196700
H	4.17597800	-0.22353500	-0.14372800
H	3.76726500	-1.14770500	1.30584000
O	-0.86832100	1.65394700	0.26801000
O	-1.45443100	1.13735300	1.45550500
H	4.41732500	-1.97747100	-0.11321600
O	1.86986400	0.95649900	-0.66667100
O	1.91327200	2.08001300	0.24785000
H	0.96364000	2.28445400	0.34188200

P82

C	1.63074700	-2.15496600	-0.72031500
C	0.33016400	-1.42449900	-0.79317300
C	-0.07668000	-0.63174700	0.40939500
C	1.06712900	0.34674600	0.80120600
C	2.38747800	-0.41029200	0.98303200
C	2.77454100	-1.21112500	-0.26917100
H	-0.17669300	-1.26356100	-1.73568800
H	1.55812100	-2.97206000	0.01655900
H	1.88038500	-2.61880000	-1.67776000
H	0.79899900	0.89767800	1.70894500
H	3.17257300	0.30386300	1.24410500
H	2.27884700	-1.08110800	1.84348600
H	3.00019300	-0.51858100	-1.08602400
H	3.68532500	-1.78681700	-0.07705100
H	-0.10622200	-1.31054100	1.27959600
C	-1.49594100	-0.02169300	0.32539500
C	-2.58192600	-1.10588500	0.30600100
H	-2.38610300	-1.80216700	1.12895200
H	-2.48308000	-1.68424300	-0.61823200
C	-3.99717400	-0.53637800	0.42055200
H	-4.13744800	-0.01393700	1.37197100
H	-4.18947900	0.17494700	-0.38397900
H	-4.74438200	-1.33218500	0.36305800
O	1.18055500	1.30822600	-0.27281200
O	2.04736300	2.38392400	0.18587200
H	2.76369100	2.31677300	-0.46080700
O	-1.69817300	0.75909000	-0.85977600
O	-1.41068400	2.14912000	-0.58129400
H	-0.43820600	2.14542600	-0.65253700
H	-1.65905000	0.64146700	1.18195200

R83

C	-1.00016600	-0.25336100	1.25086700
C	-2.11803700	-1.13124100	0.68529800
O	-0.15900900	0.71528200	-0.85579000
O	-0.74612500	1.86150200	-0.59552100
O	-2.62004400	-0.70976500	-0.57755200
O	-3.30247000	0.55189000	-0.38431900
H	-2.94003600	-1.18143700	1.40787700
H	-1.77771400	-2.15113500	0.48506700
H	-2.64105700	1.17938100	-0.72711000
H	-1.40605800	0.73203700	1.48823100
H	-0.67410300	-0.69446100	2.19893700
C	0.24539400	-0.05085300	0.37593500

C	1.30098300	0.78515100	1.11503900
C	0.82859100	-1.34151900	-0.20907800
C	2.59084900	0.99130700	0.31025700
H	1.53149000	0.26407500	2.05143900
H	0.85060800	1.74394600	1.38429400
C	2.13200000	-1.12094900	-0.99320600
H	1.01122900	-2.02505800	0.62881300
H	0.08121500	-1.81234800	-0.85175500
C	3.16948800	-0.34341800	-0.17513500
H	3.31911700	1.52474600	0.92836900
H	2.38243900	1.63349800	-0.55196700
H	2.53314500	-2.09120900	-1.30104800
H	1.90722800	-0.56952600	-1.91188900
H	4.06871600	-0.17096100	-0.77408900
H	3.48035900	-0.94352200	0.69042300

TS83

C	-2.92135500	-0.08514600	0.63079900
C	-2.46321500	0.40931900	-0.72458100
C	-1.27151700	1.32746300	-0.65389300
C	-0.06461900	0.52949700	-0.09709100
C	-0.41666000	-0.07901600	1.27601000
C	-1.77689300	-0.80195700	1.37915800
H	-1.80578800	-0.65079200	-1.25119800
H	-3.23586900	0.66392100	-1.44905100
H	-3.26262800	0.78316500	1.21603200
H	-3.78261500	-0.75205400	0.53766600
H	-0.39777500	0.75602900	1.98773800
H	-1.67194700	-1.80732700	0.97289000
H	-2.04018600	-0.90927100	2.43556100
H	-1.47560300	2.16844700	0.02468400
O	0.22469300	-0.46067100	-1.12979700
O	-0.84564400	-1.37906500	-1.28696600
H	-1.00992100	1.73924800	-1.63127400
H	0.38109300	-0.76264300	1.57093800
C	1.19717200	1.41041500	-0.06921700
H	0.93356900	2.32980400	0.46587200
H	1.42390200	1.69802700	-1.10066200
C	2.48098000	0.86609900	0.57171600
H	3.22281000	1.67145600	0.57894600
H	2.32841100	0.53871600	1.60411900
O	3.12872500	-0.16538300	-0.15999200
O	2.49547700	-1.42942400	0.15987300
H	1.78318300	-1.45041900	-0.50461100

P83

C	3.12878000	0.51908400	-0.10206700
C	2.64557400	-0.89104300	-0.15268700
C	1.39901000	-1.26036100	0.57876500
C	0.25288600	-0.24060600	0.38799400
C	0.76569900	1.20398000	0.53622200
C	1.97734500	1.51253000	-0.35489700
H	3.25180900	-1.66670100	-0.60352000
H	3.55690700	0.73686700	0.89464800
H	3.93761000	0.68208100	-0.81955300
H	1.03682800	1.34539500	1.58947300
H	1.67880500	1.45643400	-1.40468100
H	2.31348000	2.53696300	-0.16720200
H	1.59429000	-1.32432600	1.66475800
H	1.03644000	-2.24379900	0.27249300
H	-0.04732000	1.90239000	0.32159700
C	-0.90913700	-0.56721600	1.34677600
H	-0.55636700	-0.40734100	2.37167700
H	-1.12810700	-1.63569400	1.26464800
C	-2.21817700	0.21204100	1.18194300
H	-2.95725200	-0.17786200	1.88874600
H	-2.10670000	1.28220500	1.36226300
O	-2.82568900	0.04621100	-0.11220700
O	-2.49143500	1.18957200	-0.93989700
H	-1.62169200	0.90442200	-1.28488600
O	-0.19847900	-0.28497600	-0.99948900
O	-0.79829000	-1.56913600	-1.29892600
H	-1.72946300	-1.37486400	-1.07860600

R84

C	-0.87635900	-0.44122400	0.91683000
C	-2.00700300	-0.92204000	-0.00322300
O	0.29477100	1.09952100	-0.62074400
O	-0.19096200	2.14970200	0.00238100
O	-2.30536900	0.01409800	-1.05032300
O	-2.84830200	1.22152400	-0.46157600
H	-1.67295800	-1.78289900	-0.59169200
H	-2.07873500	1.81634900	-0.51091200
H	-1.21705800	0.43928900	1.46669300
H	-0.69082900	-1.22177600	1.66149600
C	0.47448300	-0.10054700	0.26854200
C	1.51058700	0.26968800	1.34149900
C	1.00063000	-1.16111500	-0.70511800
C	2.90001400	0.58073300	0.77006800
H	1.57967000	-0.57791800	2.03292800

H	1.12391200	1.11808000	1.91175500
C	2.40282500	-0.84680400	-1.25052300
H	1.01724000	-2.11701400	-0.16793700
H	0.29492300	-1.26839100	-1.53181900
C	3.40475400	-0.55558100	-0.12757600
H	3.59650100	0.75843600	1.59492300
H	2.85598200	1.51001100	0.19232600
H	2.74473200	-1.68644200	-1.86312900
H	2.34116100	0.02078000	-1.91540000
H	4.38071700	-0.29945200	-0.55070700
H	3.55329700	-1.46033600	0.47684000
C	-3.26073300	-1.30851400	0.77816000
H	-4.03702300	-1.64519500	0.08847600
H	-3.04442600	-2.11761500	1.48124400
H	-3.64340400	-0.45223800	1.33506200

TS84

C	-3.18142100	-0.32152900	0.70603000
C	-2.06625000	0.51161900	1.30082900
C	-0.71035400	-0.14505500	1.25053200
C	-0.32441100	-0.35726600	-0.23305300
C	-1.41358800	-1.18611700	-0.95043700
C	-2.86909100	-0.71795300	-0.75331800
H	-1.80948900	1.43471000	0.34564500
H	-2.29792800	1.07679800	2.20278700
H	-3.29828900	-1.22881900	1.31981700
H	-4.13718500	0.20733900	0.75425800
H	-1.31583000	-2.20911700	-0.56774500
H	-3.05936800	0.13828900	-1.39904300
H	-3.54540200	-1.51806200	-1.06924500
H	-0.73944400	-1.12731000	1.74609400
O	-0.17324000	0.97785500	-0.80269600
O	-1.36830500	1.73554600	-0.73430500
H	0.05007100	0.46333000	1.73945700
H	-1.17459900	-1.22604000	-2.01721700
C	1.06172400	-0.99232500	-0.45908900
H	1.36023900	-0.76209700	-1.48589000
H	0.94957000	-2.07914300	-0.40676800
C	2.20378800	-0.61024000	0.50046000
H	1.97726300	-0.98916600	1.50390300
O	2.29298400	0.79564800	0.76066700
O	2.53835500	1.49390700	-0.48672200
H	1.62034300	1.71118200	-0.73156300
C	3.54366300	-1.17707400	0.03777100
H	4.31852400	-0.94562500	0.77095200

H	3.49046300	-2.26291000	-0.08271000
H	3.82834300	-0.73136500	-0.91662200

P84

C	-3.40463000	0.04371900	-0.53496800
C	-2.89523700	-0.70689500	0.64864100
C	-1.53366200	-1.31363500	0.59377400
C	-0.46502600	-0.36575700	-0.00027900
C	-0.98809800	0.32804300	-1.27267300
C	-2.34227800	1.02342000	-1.07215900
H	-3.54062100	-0.91117200	1.49363300
H	-3.66297500	-0.66069400	-1.34797400
H	-4.32785200	0.57741900	-0.29342500
H	-1.08028600	-0.44248100	-2.04761700
H	-2.22426200	1.84819600	-0.36496100
H	-2.67205700	1.45504900	-2.02230100
H	-1.54254900	-2.22337700	-0.03367800
H	-1.19930900	-1.62616100	1.58517900
H	-0.24560200	1.04734900	-1.62764200
C	0.84822400	-1.14011800	-0.23285300
H	0.65154900	-1.89162300	-1.00465000
H	1.08167900	-1.69676800	0.68041200
C	2.10359200	-0.35691300	-0.64965800
H	1.95114400	0.16960900	-1.59362100
O	2.45104200	0.65796100	0.32872100
O	1.95221000	1.94519800	-0.11168300
H	1.04432700	1.90808900	0.25063600
O	-0.26007300	0.74904300	0.91822000
O	0.32323100	0.28418100	2.15971100
H	1.27058800	0.37293600	1.93969800
C	3.32347300	-1.26710900	-0.75337600
H	3.14355300	-2.07080900	-1.47203900
H	4.19406700	-0.69429800	-1.07560400
H	3.54918500	-1.72085600	0.21551900

R85

C	-0.57374700	-0.20470300	0.94049300
C	-1.76705000	-0.56256900	0.04498400
O	0.76573000	1.13156700	-0.65132200
O	0.42199500	2.25198400	-0.05700500
O	-1.95719700	0.38066100	-1.02171000
O	-2.33793800	1.66020400	-0.45706000
H	-1.55287400	-1.47249400	-0.52708200
H	-1.49907700	2.15076800	-0.52353000
H	-0.79290100	0.72638300	1.46841700

H	-0.47889800	-0.98189900	1.70537300
C	0.80216300	-0.05556600	0.27269300
C	1.88717200	0.21198400	1.32741600
C	1.18163400	-1.20209200	-0.67128200
C	3.29963100	0.32793100	0.73974800
H	1.85509200	-0.61694100	2.04372900
H	1.61622300	1.11853000	1.87450300
C	2.60726400	-1.08384400	-1.23357400
H	1.08248800	-2.13574900	-0.10466400
H	0.46093100	-1.24522200	-1.49097800
C	3.64862000	-0.88883900	-0.12587400
H	4.02008600	0.43985100	1.55554500
H	3.36859900	1.23819200	0.13467300
H	2.83452000	-1.97784000	-1.82211900
H	2.64957000	-0.23567500	-1.92460700
H	4.64497400	-0.77055500	-0.56227700
H	3.68771100	-1.78714100	0.50456800
C	-3.05046600	-0.77961700	0.85535300
H	-2.85009900	-1.54811400	1.61081000
H	-3.27837600	0.14407300	1.39386900
C	-4.24491200	-1.18927100	-0.00867900
H	-5.14169100	-1.31583100	0.60308400
H	-4.45153500	-0.43022400	-0.76465500
H	-4.05731100	-2.13592400	-0.52555300

TS85

C	-3.45160400	-0.60121800	0.79084400
C	-2.35182800	0.21426600	1.43664100
C	-0.96411800	-0.32649100	1.20411800
C	-0.67226400	-0.28785500	-0.31515900
C	-1.75521100	-1.07907500	-1.08278500
C	-3.21968100	-0.75338300	-0.72816300
H	-2.22289200	1.28487200	0.61990400
H	-2.55391200	0.62110700	2.42673800
H	-3.46652000	-1.59542500	1.26505700
H	-4.43308700	-0.15692000	0.97740100
H	-1.56581300	-2.13749700	-0.86746300
H	-3.50962800	0.17258700	-1.22298100
H	-3.86467900	-1.54547800	-1.12053000
H	-0.89563300	-1.37085800	1.54444700
O	-0.64743800	1.12423800	-0.68306100
O	-1.87989400	1.77408600	-0.42655400
H	-0.21074800	0.25645000	1.73320000
H	-1.59094400	-0.94293500	-2.15573200
C	0.73156600	-0.77986000	-0.71937500

H	0.93931100	-0.38236000	-1.71701400
H	0.69129400	-1.86791500	-0.82307800
C	1.91376600	-0.46018400	0.21333000
H	1.78903600	-1.00039800	1.16022600
O	1.92767300	0.89485500	0.68117300
O	2.03605600	1.78905000	-0.45619100
H	1.09005100	1.96632900	-0.61076700
C	3.25352800	-0.85660500	-0.41618300
H	3.18042400	-1.89224700	-0.76825300
H	3.40666300	-0.22976700	-1.29898200
C	4.43511100	-0.70780900	0.54426600
H	5.37701400	-0.95102800	0.04609700
H	4.49987200	0.31630400	0.91580900
H	4.33343900	-1.37337900	1.40777300

P85

C	3.68641400	-0.59896500	0.51441000
C	3.26694000	-0.22564000	-0.86737800
C	1.88717300	-0.56087000	-1.32387500
C	0.79903600	-0.23262000	-0.27451700
C	1.22099000	-0.71420900	1.12653400
C	2.60967300	-0.21236900	1.54812500
H	3.98344500	0.17559100	-1.57304900
H	3.84743700	-1.69163900	0.57943000
H	4.64396400	-0.13773100	0.77121700
H	1.21528900	-1.81066200	1.10783000
H	2.58546100	0.87532800	1.65128000
H	2.86174300	-0.62625500	2.52940300
H	1.80628800	-1.64090200	-1.54394400
H	1.63822500	-0.03751000	-2.24934100
H	0.47122400	-0.40531300	1.85956900
C	-0.55570700	-0.81470600	-0.72776900
H	-0.45921300	-1.90531800	-0.72408200
H	-0.71794700	-0.52730300	-1.77147500
C	-1.81650800	-0.45166600	0.07232000
H	-1.73381600	-0.76744700	1.11471900
O	-2.03104800	0.98381700	0.09879800
O	-1.55091600	1.52690300	1.35372300
H	-0.61014400	1.66925600	1.12628900
O	0.71995100	1.21453500	-0.11073500
O	0.25179900	1.83971100	-1.33039200
H	-0.71005800	1.83075100	-1.16116100
C	-3.07748600	-1.05716600	-0.55196600
H	-2.89632500	-2.12441400	-0.71999100
H	-3.22073800	-0.60871500	-1.54189700

C	-4.33195100	-0.85884700	0.30060800
H	-4.50104900	0.20169600	0.49401000
H	-5.21423900	-1.26012300	-0.20430100
H	-4.23927900	-1.36668200	1.26539000

R86

C	0.64197300	0.14896100	0.22522200
C	1.45126400	1.37459000	-0.21344200
C	2.82478700	1.02119900	-0.80460900
C	3.63129800	0.11887200	0.13712800
C	2.84243500	-1.14883300	0.48847000
C	1.46954400	-0.81382300	1.08724400
H	2.68456800	0.51269300	-1.76396300
H	0.86722700	1.95910500	-0.92907000
H	1.58357600	2.00343400	0.67493100
H	4.58794000	-0.14662600	-0.32269900
H	3.40327800	-1.76639500	1.19629200
H	0.88168600	-1.72156600	1.24691200
H	1.59879800	-0.34069700	2.06769900
H	3.37369800	1.94383400	-1.01602700
H	3.86845400	0.66891400	1.05748400
H	2.70601600	-1.75365700	-0.41426700
O	0.36017500	-0.55077000	-1.08390700
O	-0.42599000	-1.59490500	-0.97528400
C	-0.69625200	0.48680600	0.88779200
H	-0.47220200	1.00739900	1.82443300
H	-1.17372400	-0.45106600	1.16806000
C	-1.66700400	1.33026800	0.03366600
H	-1.49718900	1.15206200	-1.03206500
H	-1.48270800	2.39657000	0.20168800
C	-3.15021300	1.05002800	0.29884400
H	-3.36702300	0.94491000	1.36812300
O	-3.66407000	-0.07897100	-0.40465200
O	-3.19940400	-1.29265300	0.24533200
H	-2.45283600	-1.54481300	-0.32330300
H	-3.76224800	1.86588700	-0.09803900

TS86

C	3.40778100	-0.08873800	-0.21525500
C	2.36766400	0.16002400	-1.28679300
C	1.16538500	-0.74234900	-1.18451100
C	0.44367100	-0.44910400	0.15403500
C	1.42112000	-0.64205200	1.33250300
C	2.79912400	0.03821600	1.19859600
H	1.73838800	1.28659700	-0.86838600

H	2.73168000	0.37956900	-2.28974600
H	3.81094200	-1.10408100	-0.35661700
H	4.25395100	0.59552700	-0.32080700
H	1.56315700	-1.72486900	1.43335300
H	2.69709200	1.09518900	1.44162100
H	3.48399400	-0.39678000	1.93261700
H	1.47414800	-1.79824600	-1.18224400
O	-0.05314700	0.91536500	0.04464600
O	0.99284600	1.85768000	-0.11771800
H	0.47860500	-0.59828700	-2.02016400
H	0.92826200	-0.30398800	2.24821900
C	-0.83052000	-1.28167200	0.36890800
H	-1.23606700	-0.98901000	1.34159500
H	-0.52381900	-2.32754600	0.45492800
C	-1.93193400	-1.12526800	-0.70561800
H	-1.65933600	-0.35213100	-1.42528300
H	-2.05926200	-2.05096500	-1.27524300
C	-3.28640000	-0.73751900	-0.10854500
H	-4.03232600	-0.59481900	-0.89811600
H	-3.65177400	-1.50232400	0.58579400
O	-3.22069900	0.42586000	0.70889000
O	-2.89444200	1.55081900	-0.14779300
H	-1.92944300	1.59913700	-0.02563400

P86

C	3.65703600	-0.13044700	-0.07385100
C	2.78523200	-0.90378600	-1.00507400
C	1.42582700	-1.32564100	-0.55369200
C	0.65359600	-0.21900300	0.20433900
C	1.56310500	0.47366200	1.23375100
C	2.87107500	0.99505700	0.62709600
H	3.18835000	-1.30749900	-1.92597700
H	4.06484900	-0.80038000	0.70655300
H	4.52480100	0.27709800	-0.60053700
H	1.78494100	-0.25073400	2.02661100
H	2.64569200	1.77780300	-0.10378800
H	3.48462300	1.45383100	1.40848900
H	1.49781900	-2.17507000	0.14910700
H	0.82314600	-1.67608100	-1.39479300
H	1.00386400	1.28793000	1.70112400
C	-0.63561000	-0.74115900	0.86072700
H	-1.09577700	0.09711900	1.38917600
H	-0.35694600	-1.47626000	1.62282500
C	-1.66564800	-1.33170700	-0.11884100
H	-1.52413500	-0.89197900	-1.10791100

H	-1.53742200	-2.41315000	-0.23183600
C	-3.10891700	-1.05622500	0.30529300
H	-3.81315300	-1.46967500	-0.42523400
H	-3.33188900	-1.48519700	1.28859800
O	-3.38176500	0.32967900	0.50125400
O	-3.24868700	0.99581600	-0.78275900
H	-2.35978300	1.38615600	-0.69441500
O	0.32162900	0.69596200	-0.87768400
O	-0.47656500	1.79744100	-0.33912300
H	0.00995600	2.55039500	-0.69998600

R87

C	-0.93781600	-0.08791300	0.22952200
C	-1.57848600	-1.42173900	-0.17075600
C	-2.98308500	-1.26963200	-0.77486500
C	-3.90556900	-0.44994800	0.13545000
C	-3.29035800	0.92038200	0.44600500
C	-1.88906000	0.78666000	1.05752700
H	-2.90511300	-0.77881000	-1.75040400
H	-0.91932800	-1.94747800	-0.86640400
H	-1.63275500	-2.03390500	0.73725000
H	-4.88594900	-0.32635100	-0.33436700
H	-3.93091100	1.48243400	1.13213200
H	-1.42513200	1.76794700	1.18776000
H	-1.96129100	0.33322400	2.05303300
H	-3.40605000	-2.26233800	-0.95639900
H	-4.07465000	-0.99609600	1.07301800
H	-3.22855500	1.50802900	-0.47608200
O	-0.74163900	0.59977700	-1.10101700
O	-0.09270200	1.73717700	-1.02692300
C	0.42805700	-0.22770800	0.90765200
H	0.26569600	-0.74665600	1.85801300
H	0.77651900	0.77169400	1.16321300
C	1.50631500	-0.96278500	0.08453700
H	1.34663900	-0.80654600	-0.98656100
H	1.42848100	-2.04103800	0.25511200
C	2.95027500	-0.51993700	0.38013700
H	3.08084800	-0.30793600	1.44813700
O	3.31387700	0.66320300	-0.35169500
O	2.66936500	1.82194800	0.23677200
H	1.90682000	1.94460700	-0.35292500
C	3.97827200	-1.55210100	-0.07478000
H	4.98933600	-1.17765600	0.09178300
H	3.85105000	-2.48225700	0.48390300

H	3.86187600	-1.76939600	-1.13987500

TS87

C	-3.41547400	-0.41590700	0.70165500
C	-3.09640400	0.40941600	-0.52621500
C	-1.99386700	1.41333700	-0.31724600
C	-0.68010500	0.65752200	0.01473400
C	-0.90328100	-0.23828500	1.25366200
C	-2.15707900	-1.13568400	1.23160700
H	-2.37775600	-0.42672200	-1.29821300
H	-3.93644400	0.72880300	-1.14232900
H	-3.81051900	0.25991700	1.47672600
H	-4.20646400	-1.14153600	0.49324000
H	-0.97916500	0.45267800	2.10262400
H	-1.96056200	-2.00704100	0.60772800
H	-2.34474500	-1.50337200	2.24491400
H	-2.22771000	2.08232600	0.52403800
O	-0.35428000	-0.07240500	-1.20462000
O	-1.35387500	-1.02527100	-1.53961700
H	-1.83651000	2.03276900	-1.20346800
H	-0.01251600	-0.84622100	1.41314500
C	0.49023100	1.65865200	0.18882800
H	0.49625700	1.97454900	1.23730900
H	0.24068000	2.55020700	-0.39484300
C	1.91654100	1.25167600	-0.23543300
H	1.92043100	0.93597900	-1.28064700
H	2.51981000	2.16509700	-0.19625100
C	2.65451300	0.19678900	0.59667500
H	2.53144700	0.41698100	1.66496700
O	2.05002500	-1.10628200	0.51923700
O	2.09470500	-1.57148000	-0.85384300
H	1.21174400	-1.29299700	-1.15649200
C	4.14209700	0.12307400	0.25966900
H	4.63474900	1.07883700	0.45837400
H	4.62140700	-0.64891600	0.86475700
H	4.28023700	-0.13138000	-0.79244400

P87

C	3.93664400	-0.43290300	-0.11696900
C	2.95546300	-1.18792900	-0.94881100
C	1.57249700	-1.40789100	-0.43059700
C	0.95211500	-0.15534900	0.23482600
C	1.96609800	0.52388200	1.17101800
C	3.30212600	0.83555100	0.48665700
H	3.28202300	-1.72102700	-1.83353100
H	4.29240200	-1.06712800	0.71704400

H	4.82700400	-0.17830400	-0.69920600
H	2.13424100	-0.14339500	2.02493400
H	3.13959000	1.56579600	-0.31226700
H	3.98760500	1.29512800	1.20531700
H	1.57070000	-2.19194000	0.34780800
H	0.90853700	-1.76384700	-1.22177400
H	1.51577100	1.43605700	1.57000900
C	-0.36357200	-0.46597000	0.96751400
H	-0.72035300	0.46631900	1.41143700
H	-0.13845600	-1.14051100	1.80025200
C	-1.47821600	-1.05043700	0.08228500
H	-1.34382300	-0.71832500	-0.94945600
H	-1.43976400	-2.14398400	0.07117200
C	-2.88044800	-0.61481500	0.52744100
H	-3.02805400	-0.86600400	1.58565100
O	-2.99526100	0.81860400	0.58101700
O	-2.80635600	1.35645800	-0.75708200
H	-1.88080700	1.65693500	-0.70247500
O	0.68531200	0.68654600	-0.92141500
O	0.03570900	1.91703500	-0.46772000
H	0.57040400	2.57110500	-0.93688600
C	-3.99660200	-1.23282300	-0.30801600
H	-3.96497300	-2.32448100	-0.24748100
H	-4.96934300	-0.89206000	0.05248500
H	-3.89455500	-0.93737600	-1.35351900

R88

C	1.18666700	-1.32685700	-0.66523800
C	1.74657400	-0.24452300	0.27610700
C	0.62480700	0.71280100	0.72203300
C	-0.55113100	-0.03784500	1.35741800
C	-1.07929400	-1.17593100	0.49042200
C	0.01381300	-2.09701000	-0.03889700
H	2.08927900	-0.73818400	1.19626100
H	0.85977900	-0.85638800	-1.59629500
H	1.98282900	-2.03050500	-0.92672500
H	-0.22292400	-0.46758300	2.30992100
H	-1.35999400	0.65585800	1.58543200
H	-1.85175000	-1.73670500	1.02334400
H	0.36824700	-2.69741400	0.80716900
H	-0.42083100	-2.79565000	-0.75949500
O	0.23250000	1.41255400	-0.46644900
O	-0.75760400	2.40664500	-0.12187700
H	-1.57117600	1.96813900	-0.42851800

O	-1.76412800	-0.64981400	-0.71900600
O	-2.69759100	0.23242300	-0.44273400
C	2.93809200	0.49884400	-0.33769600
H	3.33164500	1.24953200	0.35370800
H	3.74715600	-0.19867000	-0.57193800
H	2.64859300	1.01181600	-1.25628000
H	1.01656100	1.44440200	1.43905600

TS88

C	-1.27915000	-0.96178500	0.28309100
C	-0.85591700	0.40769300	0.87884500
C	0.59607500	0.40733200	1.40486500
C	1.65395900	-0.40463200	0.63745800
C	1.10786500	-1.78464100	0.21194300
C	-0.15188600	-1.52398900	-0.56870400
H	0.93091700	1.43793100	1.52014800
H	-1.38490100	-1.63550200	1.14944000
H	2.55543500	-0.50146300	1.24768200
H	1.86575500	-2.30813800	-0.37341600
H	0.89934600	-2.37950300	1.11265500
H	0.35097600	-0.46926800	-1.28746200
H	-0.43119700	-2.25844600	-1.32336300
H	0.55564900	-0.02417400	2.41131600
O	2.16072400	0.20747500	-0.55672600
O	1.08416600	0.48701100	-1.43510900
O	-1.14945800	1.34712600	-0.17319500
O	-0.33214800	2.53059300	-0.09546600
H	0.35995900	2.26474200	-0.72772900
C	-2.63271900	-0.91395600	-0.43580200
H	-2.58753700	-0.27800000	-1.31996000
H	-2.93115100	-1.91999000	-0.74339300
H	-3.41095200	-0.51686400	0.22107600
H	-1.50792200	0.65489700	1.72679000

P88

C	-1.81454500	-0.28739800	0.25109600
C	-0.70343400	0.68809400	0.72059600
C	0.47672200	-0.06717200	1.34509000
C	1.06514500	-1.16842700	0.45747200
C	-0.02859300	-2.11971300	-0.07203800
C	-1.22445400	-1.38380400	-0.57925500
H	1.25656700	0.64004200	1.62689600
H	-2.19331300	-0.72808300	1.19140500
H	1.82158100	-1.73066500	1.01934400
H	0.39962200	-2.77136900	-0.83733900
H	-0.29909700	-2.76994600	0.77716700

H	-1.61693500	-1.59153100	-1.56646300
H	0.13252300	-0.53601900	2.27440100
O	-0.33432600	1.42613400	-0.44711600
O	0.68430700	2.39183500	-0.08924800
H	1.48635100	1.89964600	-0.34116300
C	-2.98100500	0.43232300	-0.43579800
H	-2.65201300	0.90425400	-1.36238400
H	-3.78103500	-0.27560000	-0.66844900
H	-3.39469600	1.21192400	0.20951000
H	-1.11404900	1.38814800	1.46030600
O	1.67951300	-0.66519600	-0.73745200
O	2.70005300	0.30930800	-0.34012600
H	3.37911200	0.09912800	-0.99467100

R89

C	-1.17823600	-1.33558100	-0.65978600
C	-0.00260400	-2.08634900	-0.01653000
C	1.09824900	-1.13612300	0.46275600
C	0.54309700	-0.02584200	1.36218900
C	-0.65982500	0.69482000	0.75916800
C	-1.75997400	-0.25265800	0.26796800
H	-0.34991600	-2.66115700	0.84988500
H	0.42834100	-2.80045300	-0.72276000
H	-0.84619700	-0.87129100	-1.59228100
H	-1.96904600	-2.04506100	-0.92196900
H	0.22795100	-0.46721700	2.31375400
H	1.32510600	0.69734500	1.59205600
H	-1.05903000	1.42997500	1.46384400
H	-2.12553400	-0.74982000	1.17745900
O	1.67258700	-0.62193100	-0.74759000
O	2.76355000	0.26496100	-0.41376400
H	2.34894100	1.13211400	-0.56820600
O	-0.24171300	1.48894500	-0.42224300
O	0.71205700	2.35097700	-0.14707900
C	-2.93805900	0.49413800	-0.36898000
H	-3.73672400	-0.20508600	-0.62983600
H	-3.35518400	1.23773200	0.31663900
H	-2.63108200	1.01183200	-1.28001200
H	1.87464200	-1.68696300	1.00612000

TS89

C	0.36869600	1.85398100	-0.29643900
C	1.30705100	0.94355600	0.51798400
C	0.55314000	-0.04860700	1.42739600
C	-0.79223900	-0.61343600	0.93952400

C	-1.67788600	0.48924400	0.29410900
C	-0.83404900	1.08789800	-0.80536800
H	1.22316600	-0.86668800	1.69210900
H	0.02787900	2.68192300	0.34242300
H	0.94598700	2.29711700	-1.11082900
H	-1.31230900	-1.08963400	1.77560600
H	-1.83156200	1.24867000	1.07745300
H	-0.33673800	-0.09854800	-1.27417400
H	-1.36613800	1.51618600	-1.65431600
H	0.33604200	0.49175500	2.35558300
O	-0.69950300	-1.68436400	-0.00706900
O	0.03325300	-1.24687300	-1.13957600
O	2.13466100	0.36572500	-0.51483100
O	2.67685400	-0.91260600	-0.13088900
H	2.01055100	-1.48227100	-0.55534000
C	-3.03646200	-0.03346300	-0.17542400
H	-3.58562900	-0.49970700	0.64722500
H	-3.64656300	0.78540400	-0.56573400
H	-2.91682900	-0.77941700	-0.96269500
H	1.96051200	1.54511400	1.16093300

P89

C	0.02785200	-2.14140900	-0.08767800
C	-1.07993100	-1.20055600	0.42611500
C	-0.51151900	-0.10383400	1.33579200
C	0.67052000	0.66673700	0.73814000
C	1.79245400	-0.29398700	0.25976600
C	1.21912100	-1.39042500	-0.58244000
H	-1.30612000	0.58806200	1.61465300
H	0.30247800	-2.79525000	0.75731400
H	-0.38888900	-2.78807600	-0.86278400
H	1.07245000	1.36520800	1.48406500
H	2.16327200	-0.73460100	1.20308800
H	1.62457800	-1.58449200	-1.56719900
H	-0.16841300	-0.57245200	2.26566200
O	-1.68325300	-0.68960400	-0.76750500
O	-2.74105200	0.23170400	-0.40466600
H	-2.26510600	1.07875700	-0.47047400
C	2.96356200	0.43717800	-0.40702100
H	3.35878400	1.22441100	0.24120700
H	3.77482900	-0.26374200	-0.61943400
H	2.65313500	0.89635500	-1.34701700
H	-1.83564800	-1.77022300	0.98140300
O	0.31161500	1.41757200	-0.42873100
O	-0.76046200	2.34054600	-0.05030000

H	-0.49249700	3.11207700	-0.56690900

R90

C	-0.42916700	-1.35505100	-0.50081200
C	0.83614600	-2.07791200	-0.01322600
C	1.95897100	-1.10456100	0.35318800
C	1.47986100	-0.02826700	1.33324800
C	0.19407300	0.66256100	0.88808300
C	-0.93268600	-0.30666200	0.51106200
H	0.60893400	-2.67762300	0.87584600
H	1.20030500	-2.76707200	-0.77941600
H	-0.22093800	-0.86270500	-1.45476200
H	-1.21340000	-2.09269900	-0.68734100
H	1.29055100	-0.49595100	2.30537300
H	2.26096100	0.71599700	1.48623100
H	-0.14018700	1.37439300	1.64828100
H	-1.17432200	-0.83011600	1.44809500
O	2.37316400	-0.55285100	-0.90505600
O	3.46908000	0.36231300	-0.68257900
H	3.01348900	1.21843100	-0.76803500
O	0.44844400	1.48826400	-0.31870100
O	1.40179700	2.37550000	-0.13901800
C	-2.20073100	0.43883000	0.05396900
H	-2.39923800	1.25408800	0.76184700
H	-2.01124200	0.91719400	-0.91272700
H	2.80881700	-1.64086500	0.79101400
C	-3.45635000	-0.43695100	-0.04286100
H	-3.30681400	-1.22570300	-0.78719600
H	-3.61736300	-0.94589000	0.91578700
C	-4.70798300	0.36595000	-0.40998900
H	-5.58883200	-0.27812300	-0.47556700
H	-4.91578700	1.13910500	0.33616900
H	-4.58762100	0.86409500	-1.37674900

TS90

C	-1.37118200	1.84293600	-0.22626400
C	-0.12831900	1.24571600	-0.85100800
C	0.86552700	0.71613800	0.15520900
C	0.16530900	-0.50709900	0.81388800
C	-1.19782900	-0.12560800	1.41800300
C	-2.13187200	0.79652100	0.60887700
H	-0.51400900	0.02662800	-1.33609800
H	0.28296800	1.76486500	-1.71636400
H	-1.08339100	2.68189800	0.42421000
H	-2.05517300	2.24280000	-0.97808700

H	0.78854200	-0.94834900	1.59485700
H	-1.74280100	-1.02822000	1.69494600
H	-0.97689300	0.40562000	2.35060300
H	-2.80404800	1.28939200	1.32136000
H	0.99205900	1.46133100	0.95806800
C	2.23961400	0.40187900	-0.45537700
H	2.60663500	1.31773700	-0.93482000
H	2.11470300	-0.33761500	-1.25224800
C	3.29637300	-0.09208600	0.53954300
H	2.98442900	-1.05034600	0.96743800
H	3.36870500	0.61372400	1.37679700
O	0.13053900	-1.54814000	-0.16944400
O	-0.72901100	-1.16455700	-1.23054400
O	-2.95725300	0.15798700	-0.38937900
O	-3.31008500	-1.19098900	-0.02673900
H	-2.61161000	-1.65724700	-0.52019100
C	4.67595200	-0.26168600	-0.10391800
H	5.04328300	0.68609900	-0.50950900
H	5.41149900	-0.62076900	0.62058000
H	4.64013800	-0.98222600	-0.92640500

P90

C	1.40815000	-2.09643800	-0.09332400
C	0.03343100	-1.77203700	-0.57490000
C	-0.86101600	-0.90802500	0.25953700
C	-0.09967300	0.35935100	0.73828800
C	1.27218800	0.00592400	1.32321200
C	2.15702800	-0.84620600	0.40652600
H	-0.30225400	-2.11086600	-1.54684000
H	1.36943900	-2.80051300	0.75521500
H	2.00207700	-2.57782800	-0.87329500
H	-0.68814800	0.89071200	1.49562300
H	1.80252900	0.91631300	1.60243600
H	1.10336400	-0.55118700	2.25228500
H	3.06426300	-1.13674800	0.95095900
H	-1.08396200	-1.43906400	1.20394600
C	-2.20434600	-0.61312700	-0.43382800
H	-2.65221800	-1.57753800	-0.70196300
H	-2.01077600	-0.08962600	-1.37519100
C	-3.21024900	0.18780400	0.39965200
H	-2.80690800	1.18432600	0.60845200
H	-3.34977000	-0.29946500	1.37281500
O	2.54544200	-0.16851200	-0.79348700
O	3.25497300	1.04450500	-0.44056900
H	2.52939800	1.69232600	-0.48923200

O	-0.01316700	1.19319800	-0.42478900
O	0.71152100	2.40896700	-0.04572600
H	0.22208400	3.05339800	-0.57406600
C	-4.56783700	0.33399800	-0.29456000
H	-5.02396200	-0.64297700	-0.48211700
H	-5.26631000	0.91555200	0.31320600
H	-4.46505800	0.84045500	-1.25928400

R91

C	-0.05906900	-1.31427000	-0.43350300
C	1.19135100	-2.08106800	0.02439800
C	2.36550900	-1.15069500	0.33594900
C	1.96489100	-0.03932900	1.31229700
C	0.69499900	0.69804800	0.89688400
C	-0.48353500	-0.22825500	0.57511300
H	0.96983300	-2.65531500	0.93148400
H	1.50007000	-2.79776500	-0.74084800
H	0.13556100	-0.84792200	-1.40334000
H	-0.87870300	-2.02193700	-0.57898800
H	1.78860300	-0.48285200	2.29814900
H	2.78107300	0.67348900	1.42656200
H	-0.71561300	-0.72513900	1.52886300
O	2.76042200	-0.63678700	-0.94432000
O	3.90047300	0.23464800	-0.77266400
H	3.47859800	1.10798700	-0.85724800
O	0.94423200	1.49121200	-0.33257900
O	1.93772100	2.34169400	-0.19900900
H	3.20631800	-1.71526600	0.75519900
H	0.41702700	1.43644300	1.65429600
C	-1.73428600	0.56200400	0.14559200
H	-1.88319100	1.38744800	0.85360000
H	-1.55053300	1.02726500	-0.82845700
C	-3.02256600	-0.26805000	0.08544900
H	-2.92426900	-1.06184700	-0.66350300
H	-3.17620000	-0.77278300	1.04875300
C	-4.26233100	0.57141400	-0.24569100
H	-4.37617400	1.36002300	0.50789400
H	-4.10356000	1.08551900	-1.20106300
C	-5.54923500	-0.25509700	-0.31841500
H	-5.75167500	-0.75773900	0.63261700
H	-6.41336500	0.37250600	-0.55206900
H	-5.48095000	-1.02647600	-1.09176900

TS91

C	-1.90363700	1.81939500	-0.02847300

C	-0.67028700	1.35054400	-0.77052300
C	0.40612800	0.79009500	0.12823000
C	-0.19140700	-0.52392500	0.70813700
C	-1.53447600	-0.27432100	1.41705100
C	-2.55987900	0.66223600	0.74720700
H	-1.02103900	0.15985300	-1.34429000
H	-0.33705000	1.96663600	-1.60534500
H	-1.62190000	2.61259400	0.67951500
H	-2.64981300	2.24487500	-0.70313200
H	0.49813200	-0.99710000	1.41073500
H	-2.01576300	-1.22692000	1.63912200
H	-1.28648400	0.18371700	2.38114100
H	-3.21338200	1.05177300	1.53686200
H	0.54120800	1.46667800	0.98837200
C	1.75654000	0.60854100	-0.58131000
H	2.04913800	1.58359300	-0.98971200
H	1.62117000	-0.05946700	-1.43725900
C	2.89077500	0.08223600	0.30537900
H	2.65571200	-0.93119600	0.64971700
H	2.97324800	0.70521700	1.20638600
O	-0.23053000	-1.47392300	-0.36324900
O	-1.16861500	-1.04624600	-1.33745800
O	-3.40899800	0.06936000	-0.25946100
O	-3.66957900	-1.32448200	-0.00444200
H	-2.97780700	-1.70431800	-0.57538400
C	4.24628500	0.05416000	-0.41127200
H	4.49531700	1.06654000	-0.75162500
H	4.16018400	-0.55870800	-1.31617900
C	5.38121200	-0.48398200	0.46418600
H	6.33205300	-0.49010600	-0.07533100
H	5.51337200	0.12782100	1.36223400
H	5.17795000	-1.50902100	0.78937200

P91

C	2.01049300	-2.01932800	-0.01534600
C	0.63035300	-1.83219400	-0.55109700
C	-0.36698800	-1.03296900	0.22938500
C	0.26054700	0.30948600	0.69656200
C	1.63728400	0.09721100	1.33589200
C	2.62696800	-0.69510600	0.47403900
H	0.36139200	-2.22190500	-1.52468400
H	2.00454800	-2.70308100	0.85048800
H	2.67235500	-2.46412100	-0.76162800
H	-0.39963700	0.80468600	1.41841700
H	2.07399400	1.05867600	1.60620300

H	1.48671000	-0.44848500	2.27484100
H	3.53596600	-0.88964500	1.05693500
H	-0.57571400	-1.55818500	1.18014400
C	-1.70518700	-0.87574300	-0.51784000
H	-2.05661900	-1.88232400	-0.77381200
H	-1.52260900	-0.36110400	-1.46608100
C	-2.80692500	-0.14542300	0.25698500
H	-2.49836100	0.88681900	0.45891500
H	-2.94792500	-0.62260900	1.23614500
O	2.99736800	-0.01547900	-0.73057700
O	3.58276700	1.26486200	-0.38786000
H	2.80515200	1.84358800	-0.48169200
O	0.31157300	1.11600700	-0.48738800
O	0.91119800	2.40224400	-0.12288300
H	0.38323200	2.98500800	-0.68466200
C	-4.14750800	-0.12230400	-0.48739100
H	-4.46802600	-1.15219700	-0.68540600
H	-4.00472900	0.34467900	-1.46931600
C	-5.25033500	0.61781500	0.27451300
H	-6.19023000	0.61900500	-0.28403800
H	-5.44229300	0.15105100	1.24574700
H	-4.97266200	1.66040200	0.45892900

R92

C	0.52174000	1.88594500	-0.90543000
C	-0.90467600	1.65797300	-0.38764500
C	-1.12111300	0.24393800	0.18042600
C	-0.04092200	-0.10271200	1.22234900
C	1.38741900	0.18944800	0.77060000
C	1.57145300	1.57780500	0.17094800
H	-1.13559700	2.37433200	0.40950800
H	-1.63110800	1.82418300	-1.18763500
H	0.69569800	1.24889100	-1.77521300
H	0.63275900	2.92148000	-1.23962700
H	-0.22210900	0.48531500	2.12807100
H	-0.12374000	-1.15223800	1.50610100
H	2.08652700	0.00991900	1.59159500
H	1.49408100	2.29994900	0.99226300
H	2.58381100	1.66590200	-0.23315200
C	-2.52387900	0.07566900	0.76323500
H	-3.27158800	0.32472300	0.00697200
H	-2.68162200	-0.95770300	1.07424200
H	-2.66946100	0.72906600	1.62777400
O	-0.97772900	-0.58836000	-0.99747700

O	-1.13076500	-1.98131300	-0.64244300
H	-0.20300100	-2.27689200	-0.64656600
O	1.81432600	-0.75070100	-0.29752500
O	1.68232100	-2.01279200	0.04479000

TS92

C	-0.45981300	-1.42534900	-0.88778800
C	-1.10665900	-0.41714600	0.09197600
C	-0.17288800	-0.05996400	1.27852900
C	1.35035500	-0.05144300	1.06060200
C	1.80704100	-1.23952400	0.18742000
C	1.01585900	-1.15616600	-1.08903900
H	-0.48966000	0.89733100	1.69307700
H	-0.58980300	-2.44289100	-0.49114500
H	-1.01307400	-1.37695500	-1.82868400
H	1.85552500	-0.05138800	2.02980200
H	2.88380000	-1.17471900	0.02238000
H	1.59771400	-2.17674900	0.72345400
H	1.12768700	0.20256700	-1.23595200
H	1.47219400	-1.56560600	-1.98861500
H	-0.35535200	-0.81130700	2.05384200
C	-2.45587300	-0.90693200	0.62425600
H	-3.12024500	-1.14011100	-0.21045200
H	-2.34698800	-1.79906200	1.24782300
O	1.88455100	1.12746100	0.44264200
O	1.24980400	1.33016900	-0.80745900
H	-2.92445000	-0.11982200	1.21973400
O	-1.37663000	0.68973700	-0.81518900
O	-1.43736200	1.95774000	-0.13426600
H	-0.52838100	2.25700400	-0.31682600

P92

C	1.30934100	1.44470300	-0.44035300
C	1.18618900	0.02124400	0.15991000
C	0.03913300	-0.03784300	1.18766200
C	-1.30262800	0.53222900	0.71281600
C	-1.14545800	1.94315600	0.10993900
C	-0.00561200	2.01581100	-0.84940900
H	-0.09451000	-1.06784600	1.52057400
H	1.78196100	2.07057300	0.33524200
H	2.00598100	1.40534700	-1.28090600
H	-2.00638100	0.55269100	1.55434400
H	-2.08712900	2.24568400	-0.35381100
H	-0.98377500	2.62017300	0.96590600
H	-0.15401800	2.39942300	-1.84937100
H	0.33862700	0.53638900	2.07173200

C	2.50403000	-0.44662400	0.77792900
H	3.30983300	-0.35882400	0.04521000
H	2.76429300	0.14892900	1.65752400
H	2.42435200	-1.49358000	1.07382500
O	0.89068900	-0.77463800	-1.00963500
O	0.65360400	-2.15151300	-0.62237900
H	-0.31849300	-2.15666200	-0.56502900
O	-1.90514000	-0.22091900	-0.34925800
O	-2.08236100	-1.59677200	0.12395100
H	-2.94505400	-1.78762100	-0.26763300

R93

C	-0.91826500	-1.25626900	-0.54744800
C	0.24978100	-2.06634500	0.03612900
C	1.42998500	-1.17912200	0.43910500
C	0.99140100	-0.03237800	1.35636100
C	-0.20278500	0.74864000	0.81546700
C	-1.38535500	-0.13496300	0.40129300
H	-0.07861400	-2.61298300	0.92777700
H	0.59584500	-2.81033900	-0.68588100
H	-0.61391300	-0.81661600	-1.50131800
H	-1.75173900	-1.93066300	-0.75852800
H	0.70816500	-0.44781000	2.32939600
H	1.82413100	0.64805300	1.53241900
H	-0.51690400	1.51196500	1.53315500
H	-1.72433200	-0.60363000	1.33728900
O	1.96050300	-0.70865600	-0.80844000
O	3.11764400	0.11718100	-0.54926100
H	2.74397900	1.00538600	-0.68782000
O	0.18947200	1.50783200	-0.39806800
O	1.20436000	2.31692800	-0.18891900
C	-2.55782900	0.69721300	-0.15229600
H	-2.73212700	1.54219700	0.52464200
H	-2.26801300	1.13306900	-1.11309000
H	2.20421800	-1.76973700	0.94234000
C	-3.86443200	-0.08687200	-0.31043900
H	-4.67184700	0.57214700	-0.63945200
H	-3.77672200	-0.88665000	-1.04974400
H	-4.17297200	-0.54009600	0.63728600

TS93

C	-0.88710700	1.86407400	-0.18695200
C	0.31285500	1.22551500	-0.85329300
C	1.30663100	0.63508200	0.11801500
C	0.57084300	-0.56870700	0.77301500

C	-0.75890700	-0.14007100	1.41848800
C	-1.67155100	0.83628300	0.64951200
H	-0.13785700	0.03315400	-1.34940200
H	0.72500400	1.74228600	-1.71970500
H	-0.54737900	2.67816700	0.47001200
H	-1.57159800	2.30628500	-0.91419300
H	1.19385300	-1.05065700	1.52983900
H	-1.33569100	-1.02324900	1.69365500
H	-0.49191500	0.36388600	2.35416300
H	-2.30357200	1.34466400	1.38737800
H	1.48604300	1.35883800	0.93053500
C	2.65110100	0.27205600	-0.53119100
H	3.04814100	1.18087100	-0.99727700
H	2.47255800	-0.44094900	-1.34065200
C	3.69549700	-0.29444800	0.43484100
H	3.39662200	-1.26738600	0.83249500
H	3.86615000	0.37935600	1.28099800
O	0.46647900	-1.58884100	-0.22722600
O	-0.40198600	-1.14909900	-1.25879100
O	-2.54874400	0.25239700	-0.33802700
O	-2.95108800	-1.08646600	0.01026600
H	-2.28678100	-1.57350100	-0.50958800
H	4.65296100	-0.43437900	-0.07264600

P93

C	0.84760300	-2.13866800	-0.04303400
C	-0.46912400	-1.71544800	-0.60335800
C	-1.33602500	-0.77283600	0.17209700
C	-0.50562500	0.44212700	0.66892000
C	0.80230800	-0.00420500	1.33222300
C	1.66522600	-0.94232800	0.48036100
H	-0.77838900	-2.04306200	-1.58782600
H	0.70714900	-2.82213500	0.81159900
H	1.44236400	-2.67882100	-0.78279000
H	-1.08913800	1.03371600	1.38443800
H	1.38769900	0.86781100	1.62375100
H	0.54244800	-0.52604000	2.26083200
H	2.51371600	-1.29331900	1.08104100
H	-1.64484400	-1.26817200	1.11195100
C	-2.61821200	-0.39028700	-0.59125300
H	-3.12364900	-1.32247000	-0.86601700
H	-2.33911700	0.09898400	-1.52856800
C	-3.58958200	0.50042600	0.18725200
H	-3.16228200	1.48614700	0.38868400
H	-3.86523600	0.05095500	1.14688100

H	-4.51102300	0.65510500	-0.38010100
O	2.17460900	-0.32213200	-0.70529900
O	2.95754200	0.83809400	-0.33103500
H	2.28964300	1.53967600	-0.43174900
O	-0.29467900	1.24088800	-0.50248400
O	0.50608700	2.40294100	-0.10780900
H	0.10276800	3.07061800	-0.67836500

R94

C	-1.70248000	-0.03179100	-0.41076000
C	-1.23004800	-1.27695900	0.36141200
C	0.27292300	-1.25803200	0.65349300
C	0.70395200	0.03079800	1.36137000
C	0.20139400	1.29303300	0.66952800
C	-1.28297900	1.25335600	0.32605400
H	-1.76117600	-1.34555900	1.31928500
H	-1.46952700	-2.18265100	-0.20266600
H	-1.19965400	-0.03683700	-1.38267500
H	0.30013600	0.02521900	2.37956500
H	1.78917600	0.06707700	1.45159800
H	0.45134400	2.17867700	1.25966400
H	-1.83918400	1.33781500	1.26836200
H	-1.53900500	2.13505600	-0.26948200
O	0.88642800	-1.40122800	-0.63599000
O	2.32115000	-1.45436800	-0.47204600
H	2.57038800	-0.55427200	-0.74656200
O	0.89552400	1.49813800	-0.62889300
O	2.20489000	1.48422200	-0.52304700
C	-3.21440800	-0.06171400	-0.64972500
H	-3.54196300	0.80904800	-1.22496800
H	-3.50655200	-0.95672100	-1.20583200
H	-3.76496400	-0.06283500	0.29763300
H	0.55691800	-2.12027100	1.26765800

TS94

C	0.55280300	-1.48573900	0.59402500
C	-0.87619500	-0.97219600	0.84296100
C	-0.92713400	0.49932700	1.30242300
C	0.13579300	1.46700500	0.75640600
C	1.53194600	0.81614200	0.72150600
C	1.42242500	-0.44855900	-0.10179300
H	-1.92474400	0.89652600	1.11549500
H	1.01633900	-1.75190600	1.55591300
H	0.48364700	-2.40693800	0.00979100
H	0.13823900	2.38273300	1.35299300

H	2.24732800	1.52323500	0.29557800
H	1.85082600	0.58548800	1.74851600
H	0.61685500	0.08471600	-1.04502900
H	-0.79392400	0.48461600	2.38997100
O	-0.10927200	1.96738700	-0.56683800
O	-0.23629100	0.87390400	-1.45942500
O	-1.52632100	-1.27736900	-0.41090400
O	-2.64416800	-0.40840800	-0.67487800
H	-2.18408400	0.24797800	-1.22886700
C	2.66853900	-0.95202500	-0.78892000
H	3.38771800	-1.33341300	-0.04941200
H	2.44268200	-1.77098300	-1.47620300
H	-1.36711900	-1.57335200	1.61764500
H	3.16559000	-0.15653700	-1.34968200

P94

C	1.58273900	-0.73501400	0.67787900
C	0.19549000	-1.37748500	0.84950100
C	-0.85096000	-0.37974500	1.38226600
C	-0.87570700	0.98333100	0.69076800
C	0.52745900	1.59019300	0.53173000
C	1.54306100	0.61281100	0.02831500
H	-1.84300900	-0.83317300	1.33080500
H	2.02755200	-0.65760900	1.68834600
H	2.21608000	-1.42255800	0.11226200
H	-1.52286300	1.67044000	1.24543600
H	0.46494400	2.46719000	-0.11923600
H	0.80686900	1.97218200	1.53264600
H	-0.63591700	-0.20112700	2.44327600
O	-0.24434400	-2.08515700	-0.31911100
O	0.02173600	-1.32951000	-1.52438900
H	-0.65367200	-0.62679900	-1.48131000
C	2.68235500	1.06232000	-0.82060800
H	3.57734400	1.31497900	-0.22528300
H	2.98404100	0.27384600	-1.51836300
H	2.42788200	1.95235300	-1.40351700
H	0.26369600	-2.20139200	1.56789600
O	-1.41652600	0.90396000	-0.65183900
O	-2.82543600	0.55813300	-0.54600000
H	-3.22301500	1.37379000	-0.88135700

R95

C	-0.95425900	-0.31378700	0.06000300
C	-0.15355000	-1.53541800	0.54969600
C	1.36023800	-1.31185800	0.51490500

C	1.77005600	-0.04729100	1.27592700
C	0.96034500	1.17982800	0.87495900
C	-0.54595400	0.94522100	0.84903400
H	-0.43626600	-1.77799300	1.58188400
H	-0.39476700	-2.40950100	-0.06146400
H	-0.69267500	-0.14915200	-0.99068500
H	1.61382800	-0.21470500	2.34708000
H	2.83269700	0.15011600	1.13805900
H	1.21819600	2.03234300	1.50898000
H	-0.88035500	0.85194700	1.89036300
H	-1.03171400	1.83520600	0.44131800
O	1.67508300	-1.23122600	-0.88306500
O	3.10350000	-1.07561900	-1.03636000
H	3.16374600	-0.12484200	-1.23653700
O	1.30784200	1.61814100	-0.50254300
O	2.59707200	1.79905500	-0.67843900
C	-2.46543200	-0.58766600	0.13886900
H	-2.66282400	-1.55510700	-0.33934900
H	-2.75727400	-0.70652000	1.19189800
H	1.88676100	-2.17556500	0.93699800
C	-3.35917900	0.47069200	-0.52085500
H	-3.23218500	1.43695400	-0.02107700
H	-3.03378700	0.61758200	-1.55756000
C	-4.84263100	0.08979400	-0.50004600
H	-5.20478400	-0.03698800	0.52506100
H	-5.45775300	0.85784200	-0.97629200
H	-5.01661800	-0.85151700	-1.03037100

TS95

C	-0.30813600	-1.31891700	1.06171200
C	-1.76425800	-0.95148500	0.72647600
C	-2.07798100	0.54509600	0.92433100
C	-0.95835400	1.56072200	0.64627900
C	0.40146000	1.07366100	1.18504800
C	0.68032800	-0.27585400	0.55697800
H	-2.96785200	0.80279600	0.35017600
H	-0.20129900	-1.41573400	2.15246200
H	-0.10236900	-2.30355100	0.63405200
H	-1.22950400	2.52977300	1.07302300
H	1.16239500	1.81702700	0.94439200
H	0.34584500	0.98495100	2.27985600
H	0.21919300	0.05548600	-0.66854800
H	-2.34080900	0.66501300	1.98135600
O	-0.75232800	1.88693900	-0.73680000
O	-0.47743300	0.69816900	-1.45922500

O	-1.90119200	-1.46484000	-0.61751000
O	-2.90860100	-0.76458800	-1.37219200
H	-2.32796200	-0.12280700	-1.82004800
C	2.11659300	-0.73608400	0.39346800
H	2.54468900	-0.89770100	1.39723600
H	2.11029200	-1.72132500	-0.08672700
H	-2.45563100	-1.51792600	1.36188700
C	3.03916400	0.20072700	-0.40010400
H	3.11517900	1.16881800	0.10535700
H	2.58829100	0.40035500	-1.37770700
C	4.44366200	-0.38061900	-0.58464100
H	5.08173500	0.30175600	-1.15177800
H	4.92834700	-0.56475400	0.37936000
H	4.41102400	-1.33110000	-1.12582000

P95

C	-0.43386200	1.27877600	0.80860600
C	1.08731600	1.50487500	0.71636300
C	1.89028100	0.29818100	1.24022300
C	1.42826400	-1.07187500	0.74275800
C	-0.09724000	-1.25043200	0.84247400
C	-0.86086300	-0.07187900	0.32353200
H	2.94722300	0.43536800	1.00231100
H	-0.70687100	1.41246700	1.87143200
H	-0.93698400	2.07368300	0.25302400
H	1.93809700	-1.86249000	1.30303100
H	-0.38353700	-2.17024800	0.32447100
H	-0.30585100	-1.42134300	1.91493000
H	1.80805000	0.29008000	2.33432100
O	1.51314100	1.94669500	-0.58046400
O	0.87489800	1.18629900	-1.63392900
H	1.33683600	0.32856400	-1.58092800
C	-2.10526800	-0.23841100	-0.48525300
H	-2.15486700	0.56377300	-1.23298800
H	1.36224300	2.37491800	1.32239900
O	1.75253100	-1.28336600	-0.65377100
O	3.20074700	-1.36010400	-0.76938900
H	3.29818600	-2.28554700	-1.03379600
C	-3.41748500	-0.22174800	0.33881800
H	-3.47641700	0.71773000	0.89968100
H	-3.38471600	-1.02424300	1.08475900
C	-4.66581800	-0.37883400	-0.53335700
H	-4.64865400	-1.32626300	-1.08123700
H	-5.57874500	-0.35932500	0.06829500
H	-4.73516100	0.42687100	-1.27064400

H	-2.06130800	-1.18211300	-1.04361900

R96

C	2.42172000	-1.05183200	-0.43829900
C	0.94258800	-1.45688100	-0.51874500
C	0.12739000	-0.85996700	0.64336200
C	0.29620000	0.66596300	0.70142700
C	1.76627100	1.08437500	0.77103500
C	2.59312700	0.47224500	-0.37015600
H	0.50884100	-1.12235000	-1.46600000
H	0.84988800	-2.54751300	-0.49885300
H	2.87202500	-1.50822400	0.45311400
H	2.96567000	-1.45106400	-1.29944500
H	-0.29447200	1.11237200	1.50229000
H	1.82568800	2.17623200	0.75760900
H	2.15786900	0.75444800	1.74064500
H	2.27623000	0.91706400	-1.31904500
H	3.64738600	0.73229100	-0.23819000
H	0.56665600	-1.22687100	1.58380200
C	-1.32907500	-1.34612900	0.66816600
H	-1.85791800	-0.98549900	1.55284400
O	-2.09103000	-0.98168900	-0.48633900
O	-3.04060500	0.04276300	-0.11371100
H	-2.62758100	0.84238000	-0.48511700
O	-0.25315300	1.24274700	-0.54438900
O	-1.17385600	2.15895900	-0.31982700
H	-1.33045400	-2.44214600	0.68972300

TS96

C	0.25490000	0.42913400	0.95677300
C	-0.28927900	-0.94198300	0.51180100
C	0.31810800	-1.32534900	-0.86096500
C	1.68369800	-0.70028600	-1.05909300
C	2.50995600	-0.53384300	0.20184200
C	1.75750400	0.33840900	1.24936700
H	0.38845500	-2.41907400	-0.94544900
H	-0.28629900	0.79691200	1.83166400
H	2.22029300	-1.01480000	-1.95414600
H	1.37852100	0.58818400	-1.28118700
H	2.71985300	-1.52903700	0.61982600
H	3.47953400	-0.08836500	-0.03048300
H	1.87631500	-0.08584600	2.24979200
H	2.16948900	1.34734200	1.27404400
H	-0.36070100	-1.00230200	-1.65619700
O	-0.04827700	1.43937900	-0.06388000

O	1.03815900	1.68689200	-0.93470100
H	0.07314600	-1.65060600	1.27014200
C	-1.81840000	-1.10641500	0.52226600
H	-2.23661900	-0.85522600	1.50264600
O	-2.51967100	-0.38569500	-0.48087100
O	-2.71512200	0.97280000	-0.01693000
H	-1.90068900	1.39988100	-0.34221600
H	-2.06093300	-2.15207600	0.30067200

P96

C	0.33861400	-0.69633800	-0.75657900
C	0.17820600	0.83072600	-0.53583700
C	0.97211300	1.35100400	0.68215600
C	2.41826600	0.98718200	0.56259100
C	2.81800400	-0.13490900	-0.34082700
C	1.73165300	-1.22621800	-0.36526400
H	0.85538600	2.44065000	0.75191800
H	0.12538800	-0.90947700	-1.81019600
H	3.16065900	1.52235600	1.14256700
H	2.99986800	0.22694000	-1.36888200
H	3.76881700	-0.57036100	-0.01865000
H	2.01212100	-2.04437000	-1.03322000
H	1.64123600	-1.64228100	0.63992600
H	0.54100200	0.94950900	1.61125900
H	0.65307100	1.28696600	-1.41586300
C	-1.26921500	1.34059600	-0.59070500
H	-1.72139200	1.16409800	-1.56818800
O	-2.14628500	0.75945400	0.39448200
O	-3.06209700	-0.15127600	-0.26483400
H	-2.48500000	-0.93450000	-0.36668100
H	-1.27862400	2.41841100	-0.39766200
O	-0.68856800	-1.50172900	-0.13267400
O	-0.62587200	-1.36035600	1.31212200
H	-1.21579500	-0.58894500	1.42769900

R97

C	2.28433000	-1.38460300	-0.62618900
C	0.75156300	-1.35651000	-0.69813000
C	0.12746500	-0.67537600	0.53648100
C	0.74414300	0.71409200	0.79564600
C	2.27168800	0.70849600	0.79274000
C	2.86017500	0.02530300	-0.44893100
H	0.43365900	-0.81903100	-1.59514800
H	0.36222400	-2.37424900	-0.78843700
H	2.60358000	-2.01702300	0.21295000

H	2.68763500	-1.84482700	-1.53309300
H	0.34875800	1.14240200	1.71822400
H	2.63062200	1.73718000	0.88666600
H	2.59436400	0.17594500	1.69546400
H	2.63274400	0.62687600	-1.33549500
H	3.95026800	-0.01086800	-0.36430400
H	0.39638000	-1.26539700	1.42421800
C	-1.41518100	-0.61827700	0.49724800
H	-1.75670900	0.14222200	1.20455700
O	-1.78416500	-0.16457200	-0.81980400
O	-2.92600900	0.71234700	-0.70877900
H	-2.47300600	1.57366300	-0.70099500
O	0.32095500	1.65762200	-0.26129900
O	-0.69147400	2.40969200	0.11358800
C	-2.10695300	-1.94240900	0.80700400
H	-1.88104500	-2.27585200	1.82447000
H	-1.80006800	-2.72285300	0.10709800
H	-3.18724700	-1.81319800	0.71896700

TS97

C	0.58391300	0.43808300	0.97482700
C	-0.30701200	-0.69595700	0.42928900
C	0.21418300	-1.14017000	-0.96121300
C	1.70472500	-0.90481400	-1.09803000
C	2.50690000	-1.06092500	0.17941700
C	1.99180700	-0.08777200	1.28006200
H	-0.00628500	-2.20298700	-1.12741900
H	0.13909500	0.88436200	1.86748400
H	2.15943400	-1.29659600	-2.00796500
H	1.77915200	0.42807600	-1.23106400
H	2.42168000	-2.10143900	0.52468600
H	3.56823100	-0.88659300	-0.00970100
H	1.95477400	-0.59584700	2.24724000
H	2.66639500	0.76200300	1.38569300
H	-0.32509900	-0.59094500	-1.73922600
O	0.60987700	1.55973600	0.02844200
O	1.75037700	1.55172700	-0.80776200
H	-0.16554000	-1.52260600	1.13933000
C	-1.82999000	-0.41811000	0.43755900
H	-2.11344600	-0.02605800	1.42058900
C	-2.65554800	-1.66696400	0.12910700
H	-3.71728500	-1.44240600	0.23848200
H	-2.48802700	-2.00891100	-0.89460600
H	-2.39575600	-2.47951200	0.81256900
O	-2.24252100	0.54292900	-0.53988600

O	-2.06544900	1.87473200	-0.00259300
H	-1.14945400	2.06624000	-0.27781500

P97

C	-0.64157500	-0.58693800	0.96020000
C	0.03729200	0.74998500	0.57489300
C	-0.61766400	1.45962600	-0.63869000
C	-2.11072600	1.40792200	-0.61670900
C	-2.81014200	0.15275400	-0.21586000
C	-2.16766900	-0.48153200	1.03176600
H	-0.28640500	2.50202400	-0.66430400
H	-0.26683000	-0.88389200	1.94793700
H	-2.66217100	2.18408200	-1.13624900
H	-3.87065600	0.34592200	-0.02620300
H	-2.76809000	-0.57802000	-1.03767600
H	-2.41235800	0.13054000	1.90781000
H	-2.57167700	-1.48098700	1.21300300
H	-0.24028000	1.00671000	-1.56926600
H	-0.14660300	1.39559400	1.44465900
C	1.57374100	0.63340700	0.47050000
H	1.95759600	0.18709900	1.39080200
O	1.96156900	-0.27327500	-0.59990600
O	2.50713000	-1.48947600	-0.03185000
H	1.67937100	-1.97057300	0.16748900
O	-0.20969600	-1.73620400	0.19556300
O	-0.45239400	-1.54135900	-1.22372500
H	0.38950000	-1.11403100	-1.47546400
C	2.28997500	1.95555800	0.21265600
H	2.00089700	2.70590700	0.95405400
H	3.36887700	1.80386600	0.27060000
H	2.05657600	2.34358500	-0.78038500

R98

C	2.27339400	-1.73912000	-0.63406600
C	0.79252800	-1.36716800	-0.79100700
C	0.24527200	-0.63906500	0.45337100
C	1.12644500	0.56709000	0.83120400
C	2.61153500	0.22016000	0.92959900
C	3.13054800	-0.50812200	-0.31803900
H	0.66176100	-0.71971100	-1.66216400
H	0.20116300	-2.26729800	-0.97791700
H	2.38383400	-2.47651100	0.17218400
H	2.63166700	-2.22197900	-1.54806600
H	0.76434800	1.04128300	1.74474100
H	3.17846100	1.13600500	1.11862000

H	2.73699100	-0.41916200	1.81180200
H	3.11211400	0.17883900	-1.17079000
H	4.17555200	-0.79407300	-0.16665200
H	0.33132300	-1.32042600	1.31186200
C	-1.24928500	-0.26097100	0.34152500
H	-1.49018900	0.48388800	1.10531300
O	-1.45169500	0.37392400	-0.94031500
O	-2.21725500	1.58602900	-0.76830500
H	-1.49985100	2.24450800	-0.72293400
O	1.00062700	1.61311900	-0.20545700
O	0.24495800	2.61916500	0.18353400
C	-2.19991400	-1.45483900	0.48396700
H	-1.97701000	-1.96363100	1.42973100
H	-1.99126000	-2.17173800	-0.31654900
C	-3.67620100	-1.05381200	0.44104400
H	-3.90946000	-0.53870300	-0.49214900
H	-4.31950700	-1.93434100	0.51696600
H	-3.92895300	-0.37977000	1.26476000

TS98

C	-0.68427000	0.00861800	-0.97591500
C	-0.01299700	-0.78732800	0.16092500
C	-0.86892600	-0.66250200	1.44673200
C	-2.33730200	-0.47164100	1.12712200
C	-2.81633200	-1.15161500	-0.14139000
C	-2.00886300	-0.65419900	-1.37555500
H	-0.73384600	-1.55853400	2.06909300
H	-0.02959300	0.09708500	-1.84425400
H	-3.01388700	-0.50238700	1.98116300
H	-2.37405200	0.80308800	0.70976300
H	-2.69790000	-2.23860200	-0.02523600
H	-3.88308200	-0.97424800	-0.29395100
H	-1.77391100	-1.49254200	-2.03664600
H	-2.59489500	0.06035800	-1.95369900
H	-0.50101400	0.17834400	2.04266600
O	-0.87219200	1.40553700	-0.56174300
O	-2.17897300	1.67473100	-0.09409500
H	-0.04116500	-1.83338100	-0.17294800
C	1.48093900	-0.51050000	0.46031400
H	1.74379700	-1.15824400	1.30832200
C	2.43538100	-0.82527200	-0.69530900
H	2.21585900	-1.83998100	-1.04730400
H	2.23320400	-0.14535800	-1.52531500
C	3.90894200	-0.71409900	-0.29639300
H	4.15580700	-1.41002000	0.51168700

H	4.55954900	-0.94284600	-1.14434100
H	4.13804200	0.29533800	0.04775100
O	1.72358900	0.77825500	1.04091200
O	1.73157200	1.79652400	0.00724500
H	0.78569700	2.03278500	-0.02847800

P98

C	2.50199000	1.41136400	-0.57807800
C	1.04039300	1.51061400	-0.28054800
C	0.26674300	0.25168000	-0.74063300
C	0.95244800	-1.02667600	-0.19281400
C	2.41085700	-1.09680900	-0.66606900
C	3.22434300	0.14561700	-0.25382800
H	0.88336300	1.63189500	0.80164800
H	0.60892400	2.40200400	-0.74601400
H	3.06685500	2.31899500	-0.76137900
H	0.42426900	-1.92537000	-0.52378900
H	2.86431200	-2.00432200	-0.26013900
H	2.40663700	-1.18845200	-1.75750100
H	3.40903200	0.08657000	0.83204500
H	4.20959200	0.12304400	-0.72975700
H	0.37563200	0.18772100	-1.83284700
C	-1.25605700	0.37616200	-0.52876900
H	-1.59519100	1.27891600	-1.04799800
C	-2.07186800	-0.82773300	-1.01589600
H	-1.89221600	-1.66797900	-0.34054300
H	-1.68066600	-1.12331000	-1.99515600
C	-3.57253500	-0.54414400	-1.11659800
H	-4.11504600	-1.43222100	-1.44976100
H	-3.98010900	-0.25015600	-0.14685100
H	-3.77976000	0.25942800	-1.83030500
O	-1.64615500	0.55062600	0.86101000
O	-1.52443700	1.94807600	1.23518300
H	-2.45493900	2.15803700	1.39848000
O	1.02082800	-1.06782600	1.23965300
O	-0.22187300	-1.59075900	1.76491400
H	-0.75546800	-0.77619300	1.82318600

R99

C	-1.00016600	-0.25336100	1.25086700
C	-2.11803700	-1.13124100	0.68529800
O	-0.15900900	0.71528200	-0.85579000
O	-0.74612500	1.86150200	-0.59552100
O	-2.62004400	-0.70976500	-0.57755200
O	-3.30247000	0.55189000	-0.38431900

H	-2.94003600	-1.18143700	1.40787700
H	-1.77771400	-2.15113500	0.48506700
H	-2.64105700	1.17938100	-0.72711000
H	-1.40605800	0.73203700	1.48823100
H	-0.67410300	-0.69446100	2.19893700
C	0.24539400	-0.05085300	0.37593500
C	1.30098300	0.78515100	1.11503900
C	0.82859100	-1.34151900	-0.20907800
C	2.59084900	0.99130700	0.31025700
H	1.53149000	0.26407500	2.05143900
H	0.85060800	1.74394600	1.38429400
C	2.13200000	-1.12094900	-0.99320600
H	1.01122900	-2.02505800	0.62881300
H	0.08121500	-1.81234800	-0.85175500
C	3.16948800	-0.34341800	-0.17513500
H	3.31911700	1.52474600	0.92836900
H	2.38243900	1.63349800	-0.55196700
H	2.53314500	-2.09120900	-1.30104800
H	1.90722800	-0.56952600	-1.91188900
H	4.06871600	-0.17096100	-0.77408900
H	3.48035900	-0.94352200	0.69042300

TS99

C	-0.10430600	-0.41596300	-0.34393200
C	-0.49202700	-0.98766600	1.02844600
C	-1.67622300	-0.20153200	1.64399800
C	-2.57025300	0.39811600	0.57864400
C	-2.66732800	-0.40824700	-0.69937900
C	-1.26509700	-0.60774700	-1.34137400
H	-2.26583200	-0.86223500	2.29458600
H	0.36149900	-0.94385100	1.70447900
H	-3.49002500	0.86025600	0.93709500
H	-1.82428800	1.42371800	0.11620700
H	-3.11366400	-1.38599800	-0.46600800
H	-3.34035500	0.07426600	-1.41157900
H	-1.18817600	-1.61956100	-1.74613100
H	-1.12556400	0.08511800	-2.17139600
H	-1.29494600	0.59356800	2.29144300
O	0.16242400	1.03014200	-0.11217500
O	-0.90301100	1.86777300	-0.50477500
C	1.20510200	-0.97475100	-0.92957500
H	1.01692800	-1.98642300	-1.30566300
H	1.46138200	-0.35982100	-1.79592800
C	2.41773900	-1.06851400	0.00585100
H	2.28495200	-1.85973800	0.75044800

H	-0.75525200	-2.04405500	0.89961100
H	3.31005900	-1.30474300	-0.58574300
O	2.65641300	0.07713500	0.80905800
O	2.93818700	1.19348900	-0.07131700
H	2.04554900	1.57901100	-0.13382200

P99

C	-0.26993100	-0.37510200	0.16037800
C	-1.02984800	0.49337900	1.17039300
C	-2.20680100	1.24270700	0.52310800
C	-3.01291900	0.34679200	-0.35762900
C	-2.72004200	-1.11385400	-0.36043200
C	-1.20751300	-1.41991400	-0.50164000
H	-2.83115000	1.68069000	1.31887300
H	-0.35511700	1.20564900	1.64621200
H	-3.84736100	0.74736900	-0.92039300
H	-3.07059100	-1.56849700	0.58371600
H	-3.27081800	-1.62653000	-1.15301800
H	-0.99834800	-2.40977000	-0.08540500
H	-0.93521300	-1.45923000	-1.56023500
H	-1.81932300	2.09390500	-0.04774400
C	0.94363200	-1.11375800	0.77217500
H	0.60503900	-1.64027300	1.67176300
H	1.25944400	-1.88960600	0.06954500
C	2.18752800	-0.31850800	1.16452300
H	1.98190400	0.44742300	1.91761300
H	-1.39664700	-0.17735800	1.95580800
H	2.94384000	-1.00321400	1.56100200
O	2.77676600	0.44728300	0.09847600
O	2.99864200	-0.41129600	-1.04632900
H	2.15055700	-0.29721700	-1.51431400
O	0.16065000	0.42553300	-0.99567000
O	0.56066400	1.76665900	-0.61423400
H	1.50537400	1.62077800	-0.40662000

R100

C	-0.87635900	-0.44122400	0.91683000
C	-2.00700300	-0.92204000	-0.00322300
O	0.29477100	1.09952100	-0.62074400
O	-0.19096200	2.14970200	0.00238100
O	-2.30536900	0.01409800	-1.05032300
O	-2.84830200	1.22152400	-0.46157600
H	-1.67295800	-1.78289900	-0.59169200
H	-2.07873500	1.81634900	-0.51091200
H	-1.21705800	0.43928900	1.46669300

H	-0.69082900	-1.22177600	1.66149600
C	0.47448300	-0.10054700	0.26854200
C	1.51058700	0.26968800	1.34149900
C	1.00063000	-1.16111500	-0.70511800
C	2.90001400	0.58073300	0.77006800
H	1.57967000	-0.57791800	2.03292800
H	1.12391200	1.11808000	1.91175500
C	2.40282500	-0.84680400	-1.25052300
H	1.01724000	-2.11701400	-0.16793700
H	0.29492300	-1.26839100	-1.53181900
C	3.40475400	-0.55558100	-0.12757600
H	3.59650100	0.75843600	1.59492300
H	2.85598200	1.51001100	0.19232600
H	2.74473200	-1.68644200	-1.86312900
H	2.34116100	0.02078000	-1.91540000
H	4.38071700	-0.29945200	-0.55070700
H	3.55329700	-1.46033600	0.47684000
C	-3.26073300	-1.30851400	0.77816000
H	-4.03702300	-1.64519500	0.08847600
H	-3.04442600	-2.11761500	1.48124400
H	-3.64340400	-0.45223800	1.33506200

TS100

C	-0.33878300	-0.40650300	-0.19844200
C	-0.73718300	-0.63315600	1.26821200
C	-2.04990800	0.11537100	1.61042700
C	-2.94469300	0.27027500	0.39819500
C	-2.85500700	-0.85865000	-0.60642600
C	-1.40092300	-1.02439100	-1.13201500
H	-2.58949400	-0.42018900	2.40385900
H	0.05835400	-0.28739300	1.92730500
H	-3.93727500	0.67763200	0.59036900
H	-2.30880300	1.22636700	-0.31169500
H	-3.18477600	-1.78886100	-0.12096700
H	-3.53842200	-0.68960200	-1.44158800
H	-1.16920500	-2.08673900	-1.23700800
H	-1.30034000	-0.57144200	-2.11856800
H	-1.81598700	1.10193300	2.02139100
O	-0.27886400	1.07032100	-0.37001600
O	-1.41535800	1.60964900	-1.00858000
C	1.06409000	-0.91971900	-0.57554600
H	1.01311000	-2.00894700	-0.67567700
H	1.30095300	-0.52478700	-1.56759700
C	2.22878100	-0.61780500	0.38454800
H	2.07328900	-1.16080900	1.32391400

H	-0.85455000	-1.71113300	1.42956200
O	2.24689400	0.73300700	0.85858000
O	2.41965400	1.62805800	-0.27083200
H	1.48564200	1.83894500	-0.45098200
C	3.57552800	-1.02091500	-0.21118200
H	4.37006000	-0.85392300	0.51858100
H	3.57878400	-2.07730000	-0.49481900
H	3.78864000	-0.41907500	-1.09591300

P100

C	-0.49060300	-0.32588300	-0.05681200
C	-1.20763800	-0.09221500	1.27896700
C	-2.52185600	0.68826700	1.10438200
C	-3.33054700	0.15827800	-0.03270400
C	-2.90078100	-1.10486800	-0.69575100
C	-1.39100100	-1.11453800	-1.04492900
H	-3.09014900	0.64424200	2.04790900
H	-0.55529100	0.43364700	1.97676500
H	-4.25840500	0.64335500	-0.31074500
H	-3.09901000	-1.96365900	-0.02929700
H	-3.48502900	-1.29620100	-1.59933400
H	-1.04045200	-2.14897200	-1.10645600
H	-1.23618500	-0.67055400	-2.03258700
H	-2.29167800	1.74836100	0.94985700
C	0.85567100	-1.07525800	0.09102500
H	0.67207500	-1.96635800	0.70064600
H	1.15361400	-1.44116600	-0.89580200
C	2.06755300	-0.35622000	0.69476800
H	1.83087700	0.04005400	1.68828300
H	-1.41023200	-1.07980000	1.70894100
O	2.40841100	0.86735300	-0.00894000
O	2.51999300	0.61114500	-1.43107700
H	1.60122900	0.79085700	-1.70602000
O	-0.29603100	0.94392900	-0.77051500
O	0.01631300	2.03676300	0.13121700
H	0.99025600	1.95447400	0.17711500
C	3.29139600	-1.26194400	0.77503800
H	4.13214800	-0.71679600	1.20764100
H	3.08718100	-2.13778600	1.39661700
H	3.57639700	-1.60093700	-0.22291900

R101

C	-0.57374700	-0.20470300	0.94049300
C	-1.76705000	-0.56256900	0.04498400
O	0.76573000	1.13156700	-0.65132200

O	0.42199500	2.25198400	-0.05700500
O	-1.95719700	0.38066100	-1.02171000
O	-2.33793800	1.66020400	-0.45706000
H	-1.55287400	-1.47249400	-0.52708200
H	-1.49907700	2.15076800	-0.52353000
H	-0.79290100	0.72638300	1.46841700
H	-0.47889800	-0.98189900	1.70537300
C	0.80216300	-0.05556600	0.27269300
C	1.88717200	0.21198400	1.32741600
C	1.18163400	-1.20209200	-0.67128200
C	3.29963100	0.32793100	0.73974800
H	1.85509200	-0.61694100	2.04372900
H	1.61622300	1.11853000	1.87450300
C	2.60726400	-1.08384400	-1.23357400
H	1.08248800	-2.13574900	-0.10466400
H	0.46093100	-1.24522200	-1.49097800
C	3.64862000	-0.88883900	-0.12587400
H	4.02008600	0.43985100	1.55554500
H	3.36859900	1.23819200	0.13467300
H	2.83452000	-1.97784000	-1.82211900
H	2.64957000	-0.23567500	-1.92460700
H	4.64497400	-0.77055500	-0.56227700
H	3.68771100	-1.78714100	0.50456800
C	-3.05046600	-0.77961700	0.85535300
H	-2.85009900	-1.54811400	1.61081000
H	-3.27837600	0.14407300	1.39386900
C	-4.24491200	-1.18927100	-0.00867900
H	-5.14169100	-1.31583100	0.60308400
H	-4.45153500	-0.43022400	-0.76465500
H	-4.05731100	-2.13592400	-0.52555300

TS101

C	-0.68225800	-0.35107900	-0.27361600
C	-0.98387600	-0.80154600	1.16427900
C	-2.31759200	-0.19592000	1.66922000
C	-3.28624000	0.06297800	0.53412500
C	-3.18622600	-0.90987800	-0.62152800
C	-1.75638800	-0.90570100	-1.23295300
H	-2.77921600	-0.86950900	2.40453700
H	-0.17455800	-0.49718400	1.82700700
H	-4.28899000	0.37327800	0.82794000
H	-2.74955900	1.14689900	-0.06465600
H	-3.43295100	-1.91736900	-0.25577200
H	-3.92398300	-0.67396300	-1.39170300
H	-1.46819800	-1.92617900	-1.49536800

H	-1.73810200	-0.31740600	-2.15063600
H	-2.12026200	0.73871300	2.20256500
O	-0.72044500	1.13595600	-0.24060200
O	-1.92100600	1.68035400	-0.74314100
C	0.72526200	-0.71420700	-0.78428100
H	0.73301400	-1.78163400	-1.02791300
H	0.88020800	-0.17728700	-1.72449900
C	1.92318300	-0.46574700	0.14928700
H	1.86003900	-1.14200800	1.01089400
H	-1.02781900	-1.89684200	1.17867900
O	1.88228000	0.80587400	0.80813300
O	1.93510100	1.85868700	-0.19014500
H	0.98073200	2.02352500	-0.29807200
C	3.25876500	-0.69467500	-0.56662000
H	3.22690600	-1.67425000	-1.05781000
H	3.34803100	0.05569700	-1.35684300
C	4.46406300	-0.61340300	0.37213500
H	5.39943400	-0.73307300	-0.18044900
H	4.48877400	0.35211800	0.88027400
H	4.42690400	-1.39543600	1.13759400

P101

C	-0.82558200	-0.28936800	-0.15784000
C	-1.40612000	-0.36159700	1.26028300
C	-2.76392300	0.35352000	1.37179300
C	-3.66728100	0.00202300	0.23674100
C	-3.25528800	-1.07955200	-0.70085200
C	-1.79502100	-0.91919100	-1.19383700
H	-3.22472100	0.09327200	2.33881800
H	-0.70639100	0.06106800	1.98183900
H	-4.63866800	0.47357700	0.15007400
H	-3.33698000	-2.06091400	-0.19968600
H	-3.92617700	-1.13479500	-1.56183700
H	-1.40923900	-1.89465500	-1.50412100
H	-1.77125000	-0.27555500	-2.07793400
H	-2.59717200	1.43587200	1.40838400
C	0.55515400	-0.97619000	-0.29103500
H	0.46336400	-1.98297700	0.12987500
H	0.76769500	-1.11241100	-1.35531700
C	1.79183400	-0.33195800	0.34499000
H	1.63886200	-0.16558900	1.41790400
H	-1.51795500	-1.42411500	1.50473800
O	2.01206300	1.03030500	-0.10857000
O	2.00489300	1.08130800	-1.55748900
H	1.05806800	1.25757000	-1.71449400

O	-0.75207000	1.10528100	-0.61811400
O	-0.40070300	2.01914000	0.45230800
H	0.57584000	1.97853200	0.40693700
C	3.05457600	-1.16986100	0.12852600
H	2.85086500	-2.19197100	0.46640100
H	3.24350300	-1.22418700	-0.94792800
C	4.28266100	-0.61253900	0.85153900
H	5.16738900	-1.21774600	0.63930300
H	4.48679800	0.41134000	0.53365900
H	4.13663300	-0.60630600	1.93646500

R102

C	0.64197300	0.14896100	0.22522200
C	1.45126400	1.37459000	-0.21344200
C	2.82478700	1.02119900	-0.80460900
C	3.63129800	0.11887200	0.13712800
C	2.84243500	-1.14883300	0.48847000
C	1.46954400	-0.81382300	1.08724400
H	2.68456800	0.51269300	-1.76396300
H	0.86722700	1.95910500	-0.92907000
H	1.58357600	2.00343400	0.67493100
H	4.58794000	-0.14662600	-0.32269900
H	3.40327800	-1.76639500	1.19629200
H	0.88168600	-1.72156600	1.24691200
H	1.59879800	-0.34069700	2.06769900
H	3.37369800	1.94383400	-1.01602700
H	3.86845400	0.66891400	1.05748400
H	2.70601600	-1.75365700	-0.41426700
O	0.36017500	-0.55077000	-1.08390700
O	-0.42599000	-1.59490500	-0.97528400
C	-0.69625200	0.48680600	0.88779200
H	-0.47220200	1.00739900	1.82443300
H	-1.17372400	-0.45106600	1.16806000
C	-1.66700400	1.33026800	0.03366600
H	-1.49718900	1.15206200	-1.03206500
H	-1.48270800	2.39657000	0.20168800
C	-3.15021300	1.05002800	0.29884400
H	-3.36702300	0.94491000	1.36812300
O	-3.66407000	-0.07897100	-0.40465200
O	-3.19940400	-1.29265300	0.24533200
H	-2.45283600	-1.54481300	-0.32330300
H	-3.76224800	1.86588700	-0.09803900

TS102

C	-0.48646500	-0.51500800	0.22927400

C	-1.44662500	-0.63054300	1.42404400
C	-2.63807000	0.34725800	1.28652800
C	-2.96482800	0.61289100	-0.16914600
C	-2.69689900	-0.54900200	-1.10408400
C	-1.20051400	-0.97374200	-1.05568700
H	-3.52049900	-0.05963800	1.79920900
H	-0.90529700	-0.43724000	2.35287300
H	-3.87081700	1.19015500	-0.35391200
H	-1.95967100	1.42129900	-0.54177700
H	-3.33601000	-1.39334900	-0.80711300
H	-2.98114200	-0.29479900	-2.12751200
H	-1.12273700	-2.06337000	-1.10142800
H	-0.67137500	-0.57545000	-1.92028500
H	-2.40793100	1.29166400	1.78866400
O	-0.10022500	0.92062100	0.15772100
O	-0.81436200	1.64175200	-0.82698000
C	0.83842700	-1.23971100	0.49144700
H	1.26063000	-0.80450600	1.40216600
H	0.61615300	-2.28719000	0.71787400
C	1.87787800	-1.12263800	-0.64394500
H	1.55458500	-0.37932700	-1.37458100
H	1.98637100	-2.06710800	-1.18586200
C	3.25470600	-0.69118900	-0.13384300
H	3.67735900	-1.42817800	0.55807300
H	3.95174900	-0.55059100	-0.96728500
H	-1.80457400	-1.66498700	1.47276000
O	3.20487500	0.49140200	0.65880900
O	2.77147500	1.58195700	-0.19495100
H	1.81346100	1.58687200	-0.01618500

P102

C	-0.68773000	-0.01029500	0.00533600
C	-1.57063600	0.71842900	1.03746900
C	-2.97145800	1.05804800	0.50632100
C	-3.67846700	-0.18762700	0.07794500
C	-2.85488800	-1.37461800	-0.30340800
C	-1.51308400	-0.93920600	-0.92276100
H	-3.54702700	1.59319200	1.27022200
H	-1.03785200	1.61345300	1.36545700
H	-4.76062600	-0.23037900	0.04605200
H	-2.65744300	-2.01820700	0.57236400
H	-3.39219000	-2.01263600	-1.01248100
H	-0.91680200	-1.80873700	-1.20515200
H	-1.72144900	-0.38915200	-1.84538400
H	-2.87433500	1.76588600	-0.33490400

C	0.47920500	-0.72878300	0.70535100
H	0.96469600	-0.00537000	1.36521100
H	0.05510000	-1.50513100	1.35101300
C	1.53800600	-1.32889600	-0.23758700
H	1.53950600	-0.78890200	-1.18648700
H	1.31727000	-2.37529700	-0.47206800
C	2.95212000	-1.25867300	0.34072600
H	3.02529600	-1.79120500	1.29556100
H	3.67784900	-1.69001100	-0.35759200
H	-1.67320500	0.07717900	1.91894800
O	3.35323600	0.06428900	0.68902500
O	3.44074000	0.83907800	-0.53596300
H	2.58738000	1.30971100	-0.51442800
O	-0.17283600	0.94990400	-0.96856100
O	0.73927400	1.87740700	-0.29897700
H	0.37211600	2.71991300	-0.59688100

R103

C	-0.93781600	-0.08791300	0.22952200
C	-1.57848600	-1.42173900	-0.17075600
C	-2.98308500	-1.26963200	-0.77486500
C	-3.90556900	-0.44994800	0.13545000
C	-3.29035800	0.92038200	0.44600500
C	-1.88906000	0.78666000	1.05752700
H	-2.90511300	-0.77881000	-1.75040400
H	-0.91932800	-1.94747800	-0.86640400
H	-1.63275500	-2.03390500	0.73725000
H	-4.88594900	-0.32635100	-0.33436700
H	-3.93091100	1.48243400	1.13213200
H	-1.42513200	1.76794700	1.18776000
H	-1.96129100	0.33322400	2.05303300
H	-3.40605000	-2.26233800	-0.95639900
H	-4.07465000	-0.99609600	1.07301800
H	-3.22855500	1.50802900	-0.47608200
O	-0.74163900	0.59977700	-1.10101700
O	-0.09270200	1.73717700	-1.02692300
C	0.42805700	-0.22770800	0.90765200
H	0.26569600	-0.74665600	1.85801300
H	0.77651900	0.77169400	1.16321300
C	1.50631500	-0.96278500	0.08453700
H	1.34663900	-0.80654600	-0.98656100
H	1.42848100	-2.04103800	0.25511200
C	2.95027500	-0.51993700	0.38013700
H	3.08084800	-0.30793600	1.44813700

O	3.31387700	0.66320300	-0.35169500
O	2.66936500	1.82194800	0.23677200
H	1.90682000	1.94460700	-0.35292500
C	3.97827200	-1.55210100	-0.07478000
H	4.98933600	-1.17765600	0.09178300
H	3.85105000	-2.48225700	0.48390300
H	3.86187600	-1.76939600	-1.13987500

TS103

C	-0.80998500	-0.43464000	0.35378800
C	-1.85784400	-0.38106500	1.47726400
C	-3.07831200	0.47819400	1.06916000
C	-3.29836500	0.44186900	-0.42980700
C	-2.90061700	-0.85743500	-1.10027200
C	-1.39470100	-1.17170900	-0.86505300
H	-3.97944300	0.12312300	1.58779900
H	-1.40346000	0.01695000	2.38736700
H	-4.21243300	0.91656100	-0.78660500
H	-2.30450500	1.22603500	-0.88011400
H	-3.52105900	-1.66805300	-0.69093000
H	-3.11268300	-0.82034400	-2.17102100
H	-1.26198100	-2.24330000	-0.69323600
H	-0.81621600	-0.91250800	-1.75071300
H	-2.93340200	1.51307900	1.39326800
O	-0.48789100	0.98280900	0.03343400
O	-1.15169000	1.45943300	-1.12036800
C	0.52240000	-1.01068200	0.84462200
H	0.85531700	-0.37624100	1.67144200
H	0.33076500	-2.00172100	1.26811100
C	1.63992000	-1.06835000	-0.21798600
H	1.35967200	-0.47999100	-1.09425100
H	1.79931800	-2.09264300	-0.56685600
C	2.97380100	-0.50999300	0.29772900
H	3.28205600	-1.06333200	1.19409900
H	-2.16996900	-1.40768000	1.69911000
O	2.80661400	0.80877900	0.84588000
O	2.36787200	1.70570200	-0.20974500
H	1.40162600	1.67840900	-0.08620400
C	4.08806000	-0.55075600	-0.74193200
H	5.01426700	-0.15999400	-0.31582600
H	4.26475300	-1.57619600	-1.07912000
H	3.82199200	0.06147900	-1.60501000

P103

C	-0.99166300	0.02194500	0.02213200
C	-1.98514900	0.70532700	0.98133500

C	-3.39645600	0.84578100	0.39245900
C	-3.94210900	-0.49710300	0.02713800
C	-2.97662600	-1.60705100	-0.23345200
C	-1.67031800	-1.06925400	-0.84889000
H	-4.05516300	1.35700400	1.10399300
H	-1.57021800	1.67563700	1.26157700
H	-5.01044600	-0.66281500	-0.04440400
H	-2.73884800	-2.15213400	0.69743700
H	-3.41186100	-2.35645200	-0.90225600
H	-0.96886800	-1.88336400	-1.03914500
H	-1.90441700	-0.62356400	-1.82047100
H	-3.35201700	1.50658900	-0.49043600
C	0.23797400	-0.49033200	0.79254900
H	0.61771800	0.33600800	1.39850000
H	-0.10385100	-1.25895200	1.49416800
C	1.38244100	-1.03308400	-0.08166400
H	1.36087600	-0.55615400	-1.06406300
H	1.27055700	-2.10818400	-0.25181900
C	2.76827500	-0.78435600	0.52873900
H	2.79778000	-1.18083700	1.55176900
H	-2.04126700	0.11360700	1.90082500
O	2.99099300	0.61401800	0.78318900
O	2.98662000	1.32855600	-0.48304300
H	2.07842400	1.68220200	-0.48779000
O	-0.57536300	0.96882800	-1.01011200
O	0.18976000	2.05148000	-0.38907900
H	-0.25136700	2.81584100	-0.78213200
C	3.90382500	-1.39029500	-0.28951900
H	4.86326300	-1.18604800	0.19014400
H	3.78130000	-2.47360400	-0.37755900
H	3.92115200	-0.95731100	-1.29095000

R104

C	-1.70248000	-0.03179100	-0.41076000
C	-1.23004800	-1.27695900	0.36141200
C	0.27292300	-1.25803200	0.65349300
C	0.70395200	0.03079800	1.36137000
C	0.20139400	1.29303300	0.66952800
C	-1.28297900	1.25335600	0.32605400
H	-1.76117600	-1.34555900	1.31928500
H	-1.46952700	-2.18265100	-0.20266600
H	-1.19965400	-0.03683700	-1.38267500
H	0.30013600	0.02521900	2.37956500
H	1.78917600	0.06707700	1.45159800

H	0.45134400	2.17867700	1.25966400
H	-1.83918400	1.33781500	1.26836200
H	-1.53900500	2.13505600	-0.26948200
O	0.88642800	-1.40122800	-0.63599000
O	2.32115000	-1.45436800	-0.47204600
H	2.57038800	-0.55427200	-0.74656200
O	0.89552400	1.49813800	-0.62889300
O	2.20489000	1.48422200	-0.52304700
C	-3.21440800	-0.06171400	-0.64972500
H	-3.54196300	0.80904800	-1.22496800
H	-3.50655200	-0.95672100	-1.20583200
H	-3.76496400	-0.06283500	0.29763300
H	0.55691800	-2.12027100	1.26765800

TS104

C	0.74025300	-1.53207500	-0.38967400
C	-0.52490000	-1.17976300	-1.18030700
C	-1.11983200	0.18581700	-0.76792200
C	-0.11538700	1.01320700	0.00060300
C	1.29283400	0.98485000	-0.56666900
C	1.83940900	-0.47889100	-0.57667800
H	-1.28484100	-1.94856400	-1.02800900
H	1.10330300	-2.51629300	-0.69732600
H	-0.48657700	1.97732700	0.34476700
H	0.07413200	0.27796500	1.14250900
H	1.22127600	1.32491000	-1.61239800
H	2.34410600	-0.67917800	-1.52604100
H	2.57922600	-0.60788500	0.21522000
O	0.38232500	-1.74425600	1.00434700
O	0.57583000	-0.57281000	1.77734800
H	-0.28334900	-1.16810300	-2.24781900
O	-2.28777600	-0.13134200	0.00009100
O	-2.97018600	1.12093800	0.29168600
H	-2.96071600	1.09357100	1.25819200
H	-1.45259400	0.74549600	-1.65492400
C	2.23837900	1.93830200	0.17061200
H	3.23947100	1.91341500	-0.26846700
H	1.87721000	2.96951600	0.12523600
H	2.32327500	1.64889300	1.22148900

P104

C	0.20728300	1.33300400	0.80823900
C	-0.62985600	0.17130700	1.37417500
C	-0.70304400	-1.06720900	0.47778800
C	0.60414700	-1.37246500	-0.15885400
C	1.83144100	-0.64044500	0.27824900

C	1.59555800	0.89487400	0.28647600
H	-1.63967800	0.51874600	1.60059000
H	0.32232600	2.07108700	1.60792600
H	0.65300500	-2.16973300	-0.89180800
H	2.04245300	-0.93613100	1.32218300
H	2.38368900	1.36814900	0.87904700
H	1.68673500	1.26167800	-0.73623200
H	-0.17907600	-0.13740900	2.32515800
O	-1.65677400	-0.90299600	-0.61743100
O	-3.00053600	-0.89396400	-0.04911800
H	-3.34756300	-1.71312600	-0.42876300
H	-1.08049800	-1.92152400	1.05935100
C	3.06206900	-1.00832500	-0.55786600
H	3.94997700	-0.48023600	-0.20019500
H	3.26709600	-2.08144500	-0.51020300
H	2.90900400	-0.73704200	-1.60632600
O	-0.51927100	2.11812700	-0.15315400
O	-0.52022100	1.46837900	-1.44766200
H	-1.19865500	0.77616300	-1.33104500

R105

C	-1.17823600	-1.33558100	-0.65978600
C	-0.00260400	-2.08634900	-0.01653000
C	1.09824900	-1.13612300	0.46275600
C	0.54309700	-0.02584200	1.36218900
C	-0.65982500	0.69482000	0.75916800
C	-1.75997400	-0.25265800	0.26796800
H	-0.34991600	-2.66115700	0.84988500
H	0.42834100	-2.80045300	-0.72276000
H	-0.84619700	-0.87129100	-1.59228100
H	-1.96904600	-2.04506100	-0.92196900
H	0.22795100	-0.46721700	2.31375400
H	1.32510600	0.69734500	1.59205600
H	-1.05903000	1.42997500	1.46384400
H	-2.12553400	-0.74982000	1.17745900
O	1.67258700	-0.62193100	-0.74759000
O	2.76355000	0.26496100	-0.41376400
H	2.34894100	1.13211400	-0.56820600
O	-0.24171300	1.48894500	-0.42224300
O	0.71205700	2.35097700	-0.14707900
C	-2.93805900	0.49413800	-0.36898000
H	-3.73672400	-0.20508600	-0.62983600
H	-3.35518400	1.23773200	0.31663900
H	-2.63108200	1.01183200	-1.28001200

H	1.87464200	-1.68696300	1.00612000

TS105

C	-1.13860900	0.66231700	0.74949300
C	-1.59349200	-0.77659300	0.43951100
C	-0.78047200	-1.32030300	-0.76689100
C	0.58319100	-0.66941800	-0.86591700
C	1.20064200	-0.32000000	0.47093800
C	0.31438600	0.68740900	1.24304200
H	-0.68039500	-2.41185700	-0.68878500
H	-1.79408700	1.11678400	1.49785900
H	1.27149800	-1.07116500	-1.60642500
H	0.27672100	0.59228700	-1.29515500
H	1.33643200	-1.24421000	1.05480500
H	0.32920200	0.45599300	2.31030400
H	0.72902100	1.68700400	1.10897400
H	-1.33373100	-1.13640700	-1.69393100
O	-1.36106400	1.47915700	-0.43801500
O	-0.16753900	1.67704900	-1.17328000
H	-1.34422400	-1.37433600	1.32666500
C	-3.10136000	-0.87727000	0.20051200
H	-3.39045800	-1.90085700	-0.05473100
H	-3.39966200	-0.22314500	-0.62227900
O	2.47307600	0.31444500	0.35432300
O	3.39402000	-0.66153800	-0.21160800
H	4.05128500	-0.69664800	0.49634000
H	-3.66525900	-0.58319000	1.09087600

P105

C	1.00523900	0.65919500	0.63762000
C	-0.24470000	0.36377500	1.47386200
C	-1.23095900	-0.58073200	0.77911000
C	-0.55758800	-1.72574700	0.10344800
C	0.79013200	-1.57687400	-0.52391500
C	1.73961400	-0.63940600	0.25921500
H	-0.75399400	1.29212400	1.73639700
H	1.67150300	1.31943400	1.20805600
H	-1.12903100	-2.63087100	-0.06986000
H	1.25940800	-2.55890300	-0.64562200
H	0.68078400	-1.16797800	-1.54152500
H	1.98557600	-1.12805400	1.21044900
H	0.07668900	-0.10289700	2.41010700
O	-1.94766100	0.28780500	-0.16752000
O	-3.08850700	-0.42993800	-0.68439200
H	-2.75512000	-0.71662000	-1.54645200
H	-1.98676900	-0.93337100	1.48616200

C	3.04402200	-0.38200400	-0.50182300
H	3.72429600	0.23952900	0.08730900
H	3.55629900	-1.32324700	-0.72131300
H	2.85176300	0.13357900	-1.44448000
O	0.70381100	1.27912100	-0.61913800
O	-0.10131200	2.46105900	-0.40252200
H	-0.98661400	2.08364700	-0.54575600

R106

C	0.52174000	1.88594500	-0.90543000
C	-0.90467600	1.65797300	-0.38764500
C	-1.12111300	0.24393800	0.18042600
C	-0.04092200	-0.10271200	1.22234900
C	1.38741900	0.18944800	0.77060000
C	1.57145300	1.57780500	0.17094800
H	-1.13559700	2.37433200	0.40950800
H	-1.63110800	1.82418300	-1.18763500
H	0.69569800	1.24889100	-1.77521300
H	0.63275900	2.92148000	-1.23962700
H	-0.22210900	0.48531500	2.12807100
H	-0.12374000	-1.15223800	1.50610100
H	2.08652700	0.00991900	1.59159500
H	1.49408100	2.29994900	0.99226300
H	2.58381100	1.66590200	-0.23315200
C	-2.52387900	0.07566900	0.76323500
H	-3.27158800	0.32472300	0.00697200
H	-2.68162200	-0.95770300	1.07424200
H	-2.66946100	0.72906600	1.62777400
O	-0.97772900	-0.58836000	-0.99747700
O	-1.13076500	-1.98131300	-0.64244300
H	-0.20300100	-2.27689200	-0.64656600
O	1.81432600	-0.75070100	-0.29752500
O	1.68232100	-2.01279200	0.04479000

TS106

C	-1.52833500	0.02481900	0.95481800
C	-0.13467500	0.50911200	1.37301400
C	0.91185600	0.41735100	0.23364800
C	0.20835400	0.33483800	-1.11025200
C	-1.01833600	1.21147600	-1.26082300
C	-2.09609900	0.87543500	-0.18893700
H	0.22992800	-0.07850500	2.21838900
H	-2.19966100	0.05512200	1.81722700
H	0.87274300	0.26543900	-1.96933000
H	-0.41461000	-0.88570800	-1.03680100

H	-0.72031400	2.26626700	-1.17702500
H	-1.43480100	1.09475300	-2.26350800
H	-2.49561500	1.79623400	0.24442300
H	-2.93208400	0.33999700	-0.63965300
O	-1.47464400	-1.39306400	0.64300800
O	-1.28574200	-1.61367100	-0.74253500
H	-0.21539300	1.54819100	1.70954100
C	1.92642300	1.56658400	0.28008300
H	2.36442800	1.62577000	1.28008100
H	1.45079800	2.52360400	0.04950300
H	2.72652700	1.38905300	-0.43961300
O	1.59390100	-0.82467600	0.54202000
O	2.54281100	-1.12441200	-0.51894100
H	2.14231900	-1.93133000	-0.87095500

P106

C	1.60486700	-0.09654600	0.62723900
C	0.24988400	0.00959600	1.33750900
C	-0.97180400	-0.26890000	0.44254300
C	-0.73230400	-1.41654800	-0.48654000
C	0.61628200	-1.70732200	-1.06011400
C	1.76298600	-1.45256300	-0.06178000
H	0.14011800	0.99553900	1.79298800
H	2.40158000	0.05913900	1.36555900
H	-1.59638100	-1.95503300	-0.86069300
H	0.65474500	-2.73825600	-1.42537400
H	0.79079700	-1.06915000	-1.94071000
H	1.77098900	-2.22892700	0.71006400
H	2.72686300	-1.49180400	-0.57510400
H	0.24515500	-0.71965600	2.15355000
C	-2.24736300	-0.44492300	1.26336400
H	-2.37703100	0.39824600	1.94577700
H	-2.20287100	-1.36799400	1.84697300
H	-3.11365200	-0.49104200	0.60206600
O	-1.08143400	1.01043400	-0.30714300
O	-2.17455900	0.95068600	-1.24892200
H	-1.70566800	0.71982900	-2.06330700
O	1.77508700	0.84649400	-0.43988300
O	1.48416900	2.18168200	0.03203600
H	0.54396200	2.23798100	-0.21382500

R107

C	-0.91826500	-1.25626900	-0.54744800
C	0.24978100	-2.06634500	0.03612900
C	1.42998500	-1.17912200	0.43910500

C	0.99140100	-0.03237800	1.35636100
C	-0.20278500	0.74864000	0.81546700
C	-1.38535500	-0.13496300	0.40129300
H	-0.07861400	-2.61298300	0.92777700
H	0.59584500	-2.81033900	-0.68588100
H	-0.61391300	-0.81661600	-1.50131800
H	-1.75173900	-1.93066300	-0.75852800
H	0.70816500	-0.44781000	2.32939600
H	1.82413100	0.64805300	1.53241900
H	-0.51690400	1.51196500	1.53315500
H	-1.72433200	-0.60363000	1.33728900
O	1.96050300	-0.70865600	-0.80844000
O	3.11764400	0.11718100	-0.54926100
H	2.74397900	1.00538600	-0.68782000
O	0.18947200	1.50783200	-0.39806800
O	1.20436000	2.31692800	-0.18891900
C	-2.55782900	0.69721300	-0.15229600
H	-2.73212700	1.54219700	0.52464200
H	-2.26801300	1.13306900	-1.11309000
H	2.20421800	-1.76973700	0.94234000
C	-3.86443200	-0.08687200	-0.31043900
H	-4.67184700	0.57214700	-0.63945200
H	-3.77672200	-0.88665000	-1.04974400
H	-4.17297200	-0.54009600	0.63728600

TS107

C	-0.81910200	0.65633500	0.65527400
C	-1.32304900	-0.65330300	0.01737700
C	-0.39689300	-1.02022500	-1.17433100
C	1.00770200	-0.48627700	-0.98813700
C	1.47179100	-0.45374800	0.45149000
C	0.55810400	0.45930100	1.30388500
H	-0.37834100	-2.11076100	-1.30859800
H	-1.52490400	1.02442100	1.40334000
H	1.75631800	-0.79968800	-1.71265500
H	0.84140300	0.85552900	-1.18944700
H	1.47563100	-1.48085000	0.84971400
H	0.42100300	0.02787500	2.29780400
H	1.04792000	1.42661400	1.42075100
H	-0.81505400	-0.61221000	-2.10070400
O	-0.83559500	1.69756400	-0.36679800
O	0.45242000	1.93110100	-0.90562400
H	-1.22588300	-1.43038900	0.78879000
C	-2.79569700	-0.59953600	-0.41917900
H	-3.00229700	-1.48151100	-1.03662500

H	-2.92928300	0.27036800	-1.07042200
O	2.78559300	0.08040500	0.60742400
O	3.70846800	-0.84292000	-0.03809200
H	4.26671600	-1.07199000	0.71707800
C	-3.80403400	-0.55456500	0.73305000
H	-3.68572800	0.34073200	1.34960200
H	-4.82846600	-0.54933200	0.35201400
H	-3.69906200	-1.42591500	1.38750400

P107

C	-0.69950500	0.59713800	0.58584900
C	-1.36489400	-0.73581000	0.19638300
C	-0.35033400	-1.62387100	-0.56048100
C	0.99309500	-1.70493900	0.08396500
C	1.59164700	-0.54382500	0.79508500
C	0.54469900	0.36129400	1.45145900
H	-0.76498600	-2.63005500	-0.68655600
H	-1.39530700	1.23098500	1.14856800
H	1.64443500	-2.53621300	-0.15874500
H	2.32177600	-0.88321900	1.53908500
H	0.21886400	-0.11221800	2.38278700
H	1.00116900	1.31515900	1.72016800
H	-0.23561400	-1.21767700	-1.57851700
H	-1.61414500	-1.23278200	1.14378400
C	-2.66530600	-0.57048300	-0.61026300
H	-3.00776500	-1.57083300	-0.90033800
H	-2.44120700	-0.03713100	-1.53821900
O	2.32606100	0.36465800	-0.10219200
O	3.40317500	-0.36961900	-0.72853500
H	4.16017400	-0.02920100	-0.23096600
C	-3.79481900	0.14689000	0.13499400
H	-3.54172300	1.18741000	0.35305500
H	-4.70838700	0.15551200	-0.46529200
H	-4.02759200	-0.34937100	1.08305400
O	-0.41000000	1.22510900	-0.66893300
O	0.38039600	2.41768000	-0.45700000
H	1.26575200	2.04864800	-0.62054400

R108

C	-0.95425900	-0.31378700	0.06000300
C	-0.15355000	-1.53541800	0.54969600
C	1.36023800	-1.31185800	0.51490500
C	1.77005600	-0.04729100	1.27592700
C	0.96034500	1.17982800	0.87495900
C	-0.54595400	0.94522100	0.84903400

H	-0.43626600	-1.77799300	1.58188400
H	-0.39476700	-2.40950100	-0.06146400
H	-0.69267500	-0.14915200	-0.99068500
H	1.61382800	-0.21470500	2.34708000
H	2.83269700	0.15011600	1.13805900
H	1.21819600	2.03234300	1.50898000
H	-0.88035500	0.85194700	1.89036300
H	-1.03171400	1.83520600	0.44131800
O	1.67508300	-1.23122600	-0.88306500
O	3.10350000	-1.07561900	-1.03636000
H	3.16374600	-0.12484200	-1.23653700
O	1.30784200	1.61814100	-0.50254300
O	2.59707200	1.79905500	-0.67843900
C	-2.46543200	-0.58766600	0.13886900
H	-2.66282400	-1.55510700	-0.33934900
H	-2.75727400	-0.70652000	1.19189800
H	1.88676100	-2.17556500	0.93699800
C	-3.35917900	0.47069200	-0.52085500
H	-3.23218500	1.43695400	-0.02107700
H	-3.03378700	0.61758200	-1.55756000
C	-4.84263100	0.08979400	-0.50004600
H	-5.20478400	-0.03698800	0.52506100
H	-5.45775300	0.85784200	-0.97629200
H	-5.01661800	-0.85151700	-1.03037100

TS108

C	0.78004500	1.79686600	-0.35148300
C	1.75892700	0.93625200	-1.15747000
C	1.69040700	-0.55740500	-0.76782100
C	0.42692700	-0.86715400	0.00105300
C	-0.82879200	-0.21610700	-0.55194200
C	-0.67181800	1.33942500	-0.54198300
H	2.78027500	1.28886900	-1.00157900
H	0.88580300	2.84485500	-0.64438600
H	0.33737700	-1.90106500	0.33091700
H	0.58427600	-0.14166700	1.15299500
H	-0.91951900	-0.54019100	-1.60246200
H	-1.03526100	1.75901900	-1.48375100
H	-1.27493400	1.77335900	0.25758900
O	1.19886900	1.81082800	1.04164700
O	0.51185800	0.83371300	1.80344000
H	1.53478000	1.04899700	-2.22288100
O	2.88145500	-0.80102300	-0.00818000
O	2.94154300	-2.23021100	0.26136200
H	2.94400700	-2.21664000	1.22820800

H	1.73769700	-1.19331600	-1.66454800
C	-2.09413000	-0.68666200	0.18390900
H	-2.09142700	-1.78309200	0.22137700
H	-2.04103700	-0.33886800	1.22250500
C	-3.40841800	-0.21861300	-0.45206900
H	-3.43720600	0.87590200	-0.48538100
H	-3.44439900	-0.55748300	-1.49500600
C	-4.64374700	-0.73160400	0.29377500
H	-5.56684900	-0.38626300	-0.17917700
H	-4.66890300	-1.82557100	0.31257400
H	-4.65088300	-0.38328200	1.33104700

P108

C	-1.23455800	-1.47171400	-0.69218600
C	-1.59105900	-0.11248400	-1.32090900
C	-1.05468500	1.10501500	-0.56239000
C	0.32114900	0.87893900	-0.04900300
C	1.09698500	-0.30963500	-0.51652900
C	0.26524900	-1.61243400	-0.34368200
H	-2.67370700	-0.02646100	-1.43097000
H	-1.52776800	-2.24801400	-1.40563700
H	0.73690200	1.59547000	0.64922100
H	1.27706500	-0.19119900	-1.60111100
H	0.71764200	-2.39803800	-0.95582100
H	0.32804800	-1.92907900	0.69783900
H	-1.16536800	-0.08622600	-2.33120400
O	-1.87072500	1.43331600	0.60369100
O	-3.15204700	1.94420100	0.13054700
H	-3.08740300	2.86139600	0.43083800
H	-1.10855600	1.99418000	-1.20888800
C	2.47221000	-0.43826900	0.15866900
H	2.90541300	-1.40726800	-0.11701800
H	2.32800800	-0.46681700	1.24590600
O	-2.09220600	-1.81078100	0.41111300
O	-1.67246500	-1.12102500	1.61389000
H	-2.03485800	-0.22546000	1.47448800
C	3.46802600	0.66998100	-0.20389200
H	3.04876500	1.64765500	0.05870200
H	3.60834000	0.68333100	-1.29178700
C	4.82599300	0.50113200	0.48379800
H	5.51701000	1.30056400	0.20325400
H	4.72261100	0.51835200	1.57310600
H	5.29154800	-0.45125200	0.21189100

R109

C	-0.42916700	-1.35505100	-0.50081200
C	0.83614600	-2.07791200	-0.01322600
C	1.95897100	-1.10456100	0.35318800
C	1.47986100	-0.02826700	1.33324800
C	0.19407300	0.66256100	0.88808300
C	-0.93268600	-0.30666200	0.51106200
H	0.60893400	-2.67762300	0.87584600
H	1.20030500	-2.76707200	-0.77941600
H	-0.22093800	-0.86270500	-1.45476200
H	-1.21340000	-2.09269900	-0.68734100
H	1.29055100	-0.49595100	2.30537300
H	2.26096100	0.71599700	1.48623100
H	-0.14018700	1.37439300	1.64828100
H	-1.17432200	-0.83011600	1.44809500
O	2.37316400	-0.55285100	-0.90505600
O	3.46908000	0.36231300	-0.68257900
H	3.01348900	1.21843100	-0.76803500
O	0.44844400	1.48826400	-0.31870100
O	1.40179700	2.37550000	-0.13901800
C	-2.20073100	0.43883000	0.05396900
H	-2.39923800	1.25408800	0.76184700
H	-2.01124200	0.91719400	-0.91272700
H	2.80881700	-1.64086500	0.79101400
C	-3.45635000	-0.43695100	-0.04286100
H	-3.30681400	-1.22570300	-0.78719600
H	-3.61736300	-0.94589000	0.91578700
C	-4.70798300	0.36595000	-0.40998900
H	-5.58883200	-0.27812300	-0.47556700
H	-4.91578700	1.13910500	0.33616900
H	-4.58762100	0.86409500	-1.37674900

TS109

C	-0.37386600	0.70439100	0.65302500
C	-0.92383300	-0.56989000	-0.01846700
C	0.00067000	-0.94802600	-1.20823300
C	1.42387400	-0.47754600	-0.99449400
C	1.87147400	-0.49531600	0.45059300
C	0.98615500	0.43661200	1.31244500
H	-0.02457700	-2.03508600	-1.36731300
H	-1.07291200	1.08580900	1.40071500
H	2.16757200	-0.80628100	-1.71729700
H	1.31589100	0.87383300	-1.16810600
H	1.82793000	-1.52995600	0.82632900
H	0.81938200	-0.00979400	2.29515400
H	1.51408400	1.38002900	1.45571700

H	-0.38907900	-0.50211100	-2.12946500
O	-0.33482800	1.76685900	-0.34638500
O	0.96817100	1.95841600	-0.86528100
H	-0.86567400	-1.36681300	0.73629300
C	-2.38754900	-0.44700700	-0.46959400
H	-2.61949300	-1.29657000	-1.12434600
H	-2.48388400	0.45240100	-1.08817900
O	3.20436100	-0.01993000	0.63293100
O	4.09593300	-0.96693500	-0.02215600
H	4.63393100	-1.23645500	0.73436100
C	-3.41759000	-0.41216800	0.66604200
H	-3.21890800	0.43731000	1.32895700
H	-3.30720800	-1.31307900	1.28236500
C	-4.85854600	-0.31782500	0.15515200
H	-5.10879100	-1.17193200	-0.48163400
H	-5.57424600	-0.29814600	0.98120900
H	-5.00784500	0.59021500	-0.43645200

P109

C	0.11862400	-0.40446200	0.67142700
C	0.92227300	0.84704900	0.26901700
C	0.06615500	1.78634000	-0.61663300
C	-1.30644600	2.03035900	-0.08784000
C	-2.08782200	0.90236800	0.49640000
C	-1.21076500	-0.03039800	1.33696400
H	0.59127700	2.73647300	-0.75805000
H	0.69836100	-1.01451600	1.37432100
H	-1.81980400	2.95737100	-0.31584300
H	-2.93241200	1.26405800	1.09414800
H	-0.98744600	0.48034900	2.28018500
H	-1.76656000	-0.93202000	1.59356500
H	-0.01258700	1.32936500	-1.61633800
H	1.13078200	1.37391100	1.20997400
C	2.26740800	0.53348700	-0.40908000
H	2.73846200	1.48805000	-0.67732000
H	2.07785600	0.00468800	-1.34823800
O	-2.63316700	0.23779600	-0.67803500
O	-3.36952300	-0.96435200	-0.23080900
H	-4.15319600	-0.88327500	-0.79024000
C	3.25101000	-0.28068300	0.43938400
H	2.83265900	-1.27257300	0.63684700
H	3.38105200	0.20313200	1.41582100
O	-0.05355200	-1.14936100	-0.53970900
O	-0.79497300	-2.35832400	-0.25086700
H	-1.70266800	-2.07216500	-0.45984600

C	4.61707100	-0.44103500	-0.23477800
H	5.08854700	0.53075600	-0.41282100
H	5.29875900	-1.03334300	0.38164800
H	4.52158400	-0.94448800	-1.20163000

R110

C	-0.05906900	-1.31427000	-0.43350300
C	1.19135100	-2.08106800	0.02439800
C	2.36550900	-1.15069500	0.33594900
C	1.96489100	-0.03932900	1.31229700
C	0.69499900	0.69804800	0.89688400
C	-0.48353500	-0.22825500	0.57511300
H	0.96983300	-2.65531500	0.93148400
H	1.50007000	-2.79776500	-0.74084800
H	0.13556100	-0.84792200	-1.40334000
H	-0.87870300	-2.02193700	-0.57898800
H	1.78860300	-0.48285200	2.29814900
H	2.78107300	0.67348900	1.42656200
H	-0.71561300	-0.72513900	1.52886300
O	2.76042200	-0.63678700	-0.94432000
O	3.90047300	0.23464800	-0.77266400
H	3.47859800	1.10798700	-0.85724800
O	0.94423200	1.49121200	-0.33257900
O	1.93772100	2.34169400	-0.19900900
H	3.20631800	-1.71526600	0.75519900
H	0.41702700	1.43644300	1.65429600
C	-1.73428600	0.56200400	0.14559200
H	-1.88319100	1.38744800	0.85360000
H	-1.55053300	1.02726500	-0.82845700
C	-3.02256600	-0.26805000	0.08544900
H	-2.92426900	-1.06184700	-0.66350300
H	-3.17620000	-0.77278300	1.04875300
C	-4.26233100	0.57141400	-0.24569100
H	-4.37617400	1.36002300	0.50789400
H	-4.10356000	1.08551900	-1.20106300
C	-5.54923500	-0.25509700	-0.31841500
H	-5.75167500	-0.75773900	0.63261700
H	-6.41336500	0.37250600	-0.55206900
H	-5.48095000	-1.02647600	-1.09176900

TS110

C	-0.03028900	0.65897000	-0.62829000
C	0.48693900	-0.52034500	0.21948400
C	-0.51809100	-0.78585300	1.37376000
C	-1.92851100	-0.37633600	1.00524100

C	-2.26541900	-0.56434400	-0.45761100
C	-1.33263900	0.28803100	-1.35116700
H	-0.48780400	-1.84754200	1.65579200
H	0.71709100	0.97215600	-1.36072100
H	-2.71927100	-0.64113100	1.70393700
H	-1.85511200	0.98817600	1.03400700
H	-2.17702600	-1.63276900	-0.71100700
H	-1.08562700	-0.25964900	-2.26321900
H	-1.86306000	1.19707400	-1.63675700
H	-0.20562300	-0.23092700	2.26474700
O	-0.16082400	1.82370000	0.24103200
O	-1.50203900	2.04065700	0.63823100
H	0.49698500	-1.39680300	-0.44365400
C	1.91109600	-0.31499300	0.75912400
H	2.10554300	-1.08290200	1.51839100
H	1.94653900	0.64818500	1.28033300
O	-3.58821400	-0.14456300	-0.78908600
O	-4.51139900	-1.03459600	-0.09884100
H	-4.98627100	-1.39943100	-0.85764800
C	3.02132100	-0.37979600	-0.29639200
H	2.86189500	0.38967700	-1.06144900
H	2.97096000	-1.34446700	-0.81875200
C	4.42500200	-0.20068400	0.29460300
H	4.59545700	-0.97052800	1.05698700
H	4.47354100	0.76131500	0.81830500
C	5.53704000	-0.26752200	-0.75578100
H	6.52311300	-0.13785600	-0.30165800
H	5.53480100	-1.23149300	-1.27444800
H	5.41497200	0.51456700	-1.51170500

P110

C	-0.23982300	-0.34727100	0.62099100
C	0.42615500	0.98829600	0.23834100
C	-0.54932100	1.87017900	-0.58046700
C	-1.91482300	1.96588400	0.01065300
C	-2.56465100	0.74771100	0.57398600
C	-1.57252700	-0.12810100	1.34571600
H	-0.12147100	2.86943500	-0.70947500
H	0.42175300	-0.92284200	1.27915300
H	-2.51914500	2.85009600	-0.15610300
H	-3.41642800	1.00496200	1.21414400
H	-1.36344700	0.36527000	2.30135400
H	-2.03088800	-1.08795900	1.58345900
H	-0.62807000	1.43745300	-1.59079200
H	0.62157600	1.50060200	1.18998400

C	1.76641900	0.82420100	-0.50050600
H	2.13633100	1.82637800	-0.75243900
H	1.58708400	0.30992500	-1.44946700
O	-3.08829600	0.07775500	-0.60738900
O	-3.68725700	-1.20664200	-0.18571500
H	-4.50212000	-1.17620600	-0.70411600
C	2.85382900	0.07816600	0.28102400
H	2.53095600	-0.95065900	0.47395200
H	2.99275100	0.55038100	1.26312800
O	-0.38529400	-1.06149200	-0.61186500
O	-0.99248900	-2.34808700	-0.34577900
H	-1.93062200	-2.14792400	-0.51644500
C	4.19960500	0.04156000	-0.45334100
H	4.53455000	1.06797600	-0.64723500
H	4.05811000	-0.42313800	-1.43629100
C	5.28723700	-0.71348200	0.31584100
H	4.99429300	-1.75248400	0.49559700
H	6.23150400	-0.72561100	-0.23542900
H	5.47737400	-0.25078900	1.28956800
